# Supplementary material for: Rescue of conformational dynamics in enzyme catalysis by directed evolution
Source: Nat Commun. 2018 Apr 3;9:1314. doi: 10.1038/s41467-018-03562-9 (PMC5883053; doi:10.1038/s41467-018-03562-9)
Supplement: Supplementary file 1 — Supplementary Information [file 41467_2018_3562_MOESM1_ESM.pdf]

Supplementary Information for

Rescue of Conformational Dynamics in Enzyme Catalysis by Directed Evolution

Otten *et al.*

This PDF file includes:

Figures 1 – 10
Tables 1 – 3
References

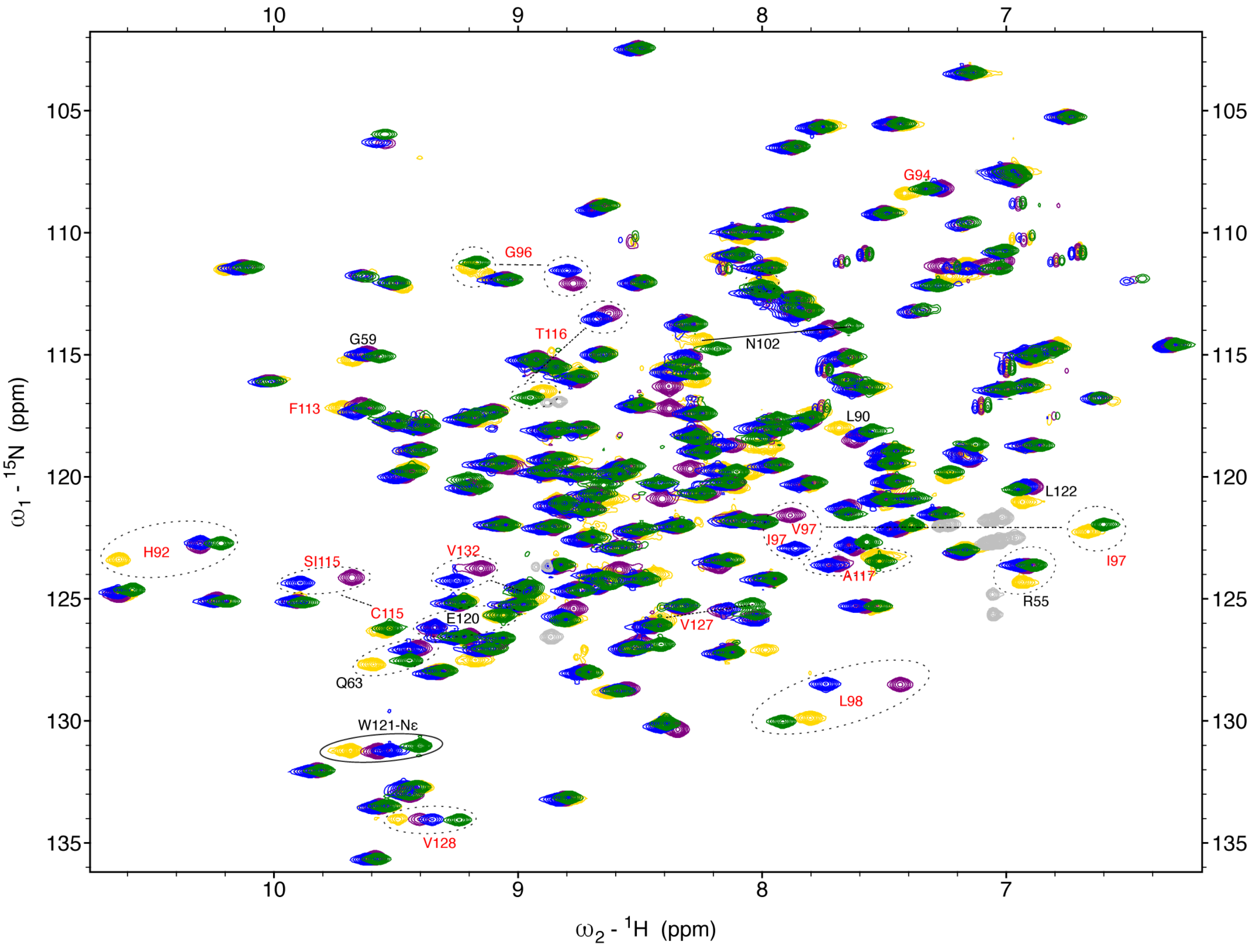

Supplementary Figure 1 | Overlay of ^{15}N-[TROSY]-HSQC spectra for wild-type CypA (yellow), S99T (green) and rescue mutants (S99T/C115S, blue; S99T/C115S/I97V, purple). All spectra were recorded on ~1 mM protein samples using a 600 MHz spectrometer at 10 °C. Sequence-specific assignments given in red indicate residues that have moved significantly due to proximity, in sequence and/or space, to the mutation site (<5 Å). Cross peaks connected with a solid line or in a solid circle show the population inversion upon the Ser99 mutation and the partial shift towards wild-type for the rescue mutants. Aliased Arg-Nε side chain signals are shown in grey for all spectra.

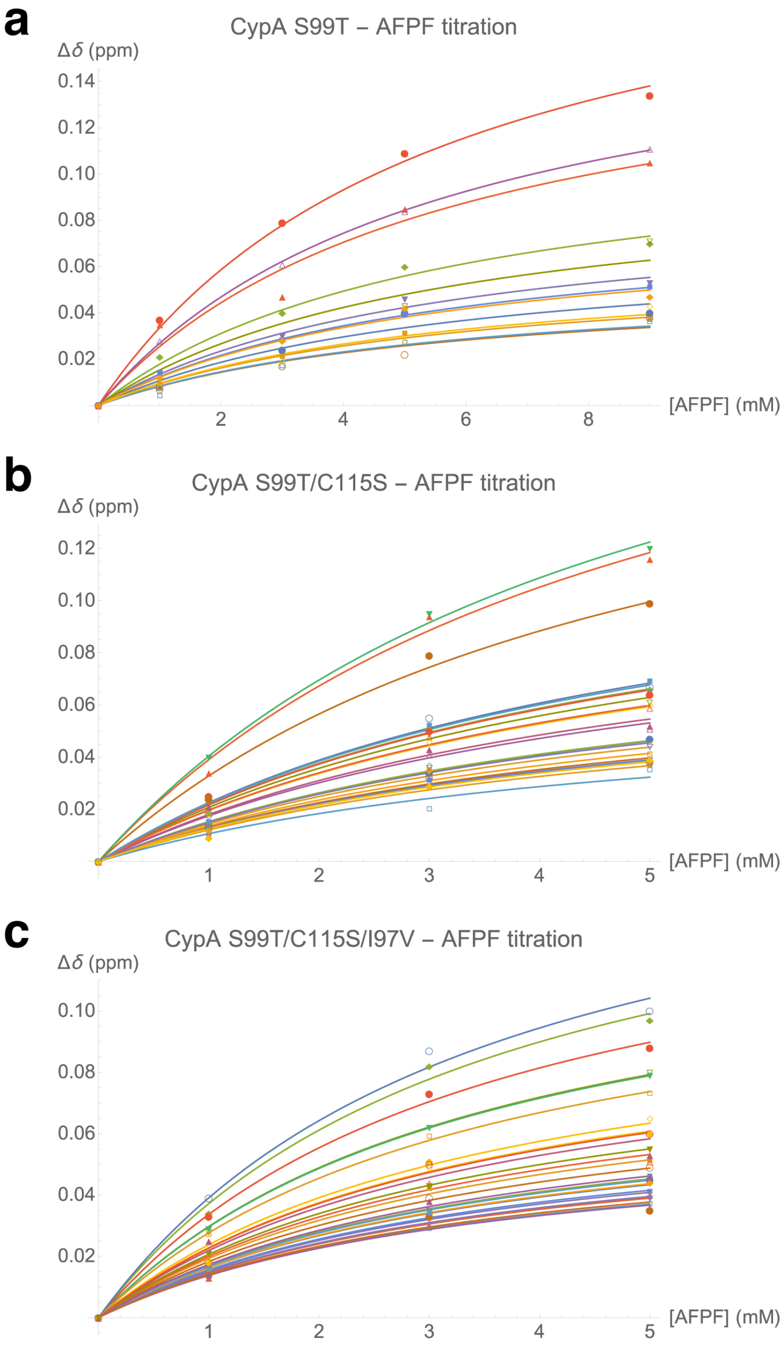

Supplementary Figure 2 | K_D determination for the three mutant forms of CypA for Suc-AFPF-pNA measured by NMR chemical shift analysis from peptide titrations. Resonances for which $\Delta\delta \geq 0.035$ ppm (thirteen for CypA S99T (**a**), twenty-three for S99T/C115S (**b**) and twenty-six for S99T/C115S/I97V (**c**), respectively) were fit simultaneously in Mathematica[1] (version 11.2) and standard errors are obtained from the global fit.

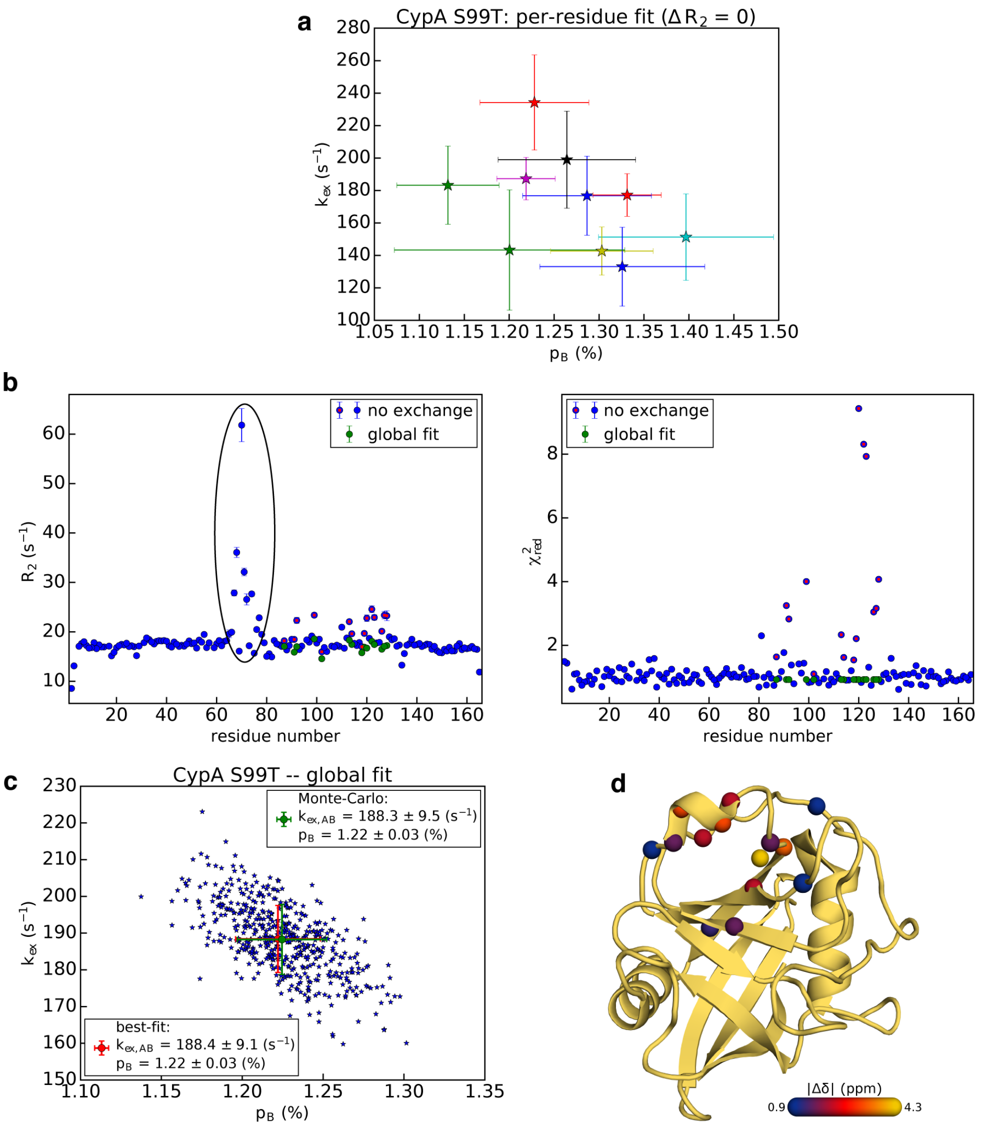

Supplementary Figure 3 | Analysis of the ^{15}N-CEST data for CypA S99T. (**a**) Per-residue fit of the CEST profiles for the initial 10 residues that clearly show slow exchange by visual inspection. Clustering of the k_{ex}/p_B values indicate the presence of one global exchange process. (**b**) Comparison of R_2 and χ^2_{red} values for no-exchange model (blue) and one global, slow-exchange process (green). For ease of comparison residues that are included in the global fit are also shown with a red marker for the no-exchange model. Fitting the data assuming no exchange results in elevated R_2 values (left) for the loop region (residues 65-80), whereas χ^2_{red} values (right) are close to one. This observation is consistent with line broadening of the ground-state signal due to dynamics on the millisecond timescale and the absence of a second dip or asymmetry that would indicate a slow-exchange process. In contrast, assuming a no-exchange model for residues that, in fact, do experience a slow-exchange process, results in large χ^2_{red} values in addition to a higher R_2 in an attempt to –unsuccessfully– explain the asymmetry in the CEST profiles. The data show that the data can be explained satisfactory by one, global exchange process as judged from the R_2 and χ^2_{red} values. (**c**) Uncertainties in the fitting parameters for the global fit were determined from 500 Monte-Carlo simulations and the resulting scatter plot for k_{ex} vs. p_B is shown. The values and their uncertainties obtained using this method and estimated from the covariance matrix are nearly identical. (**d**) The chemical shift difference between the states, $|\Delta\delta|$, for the 15 residues is plotted on the structure of CypA.

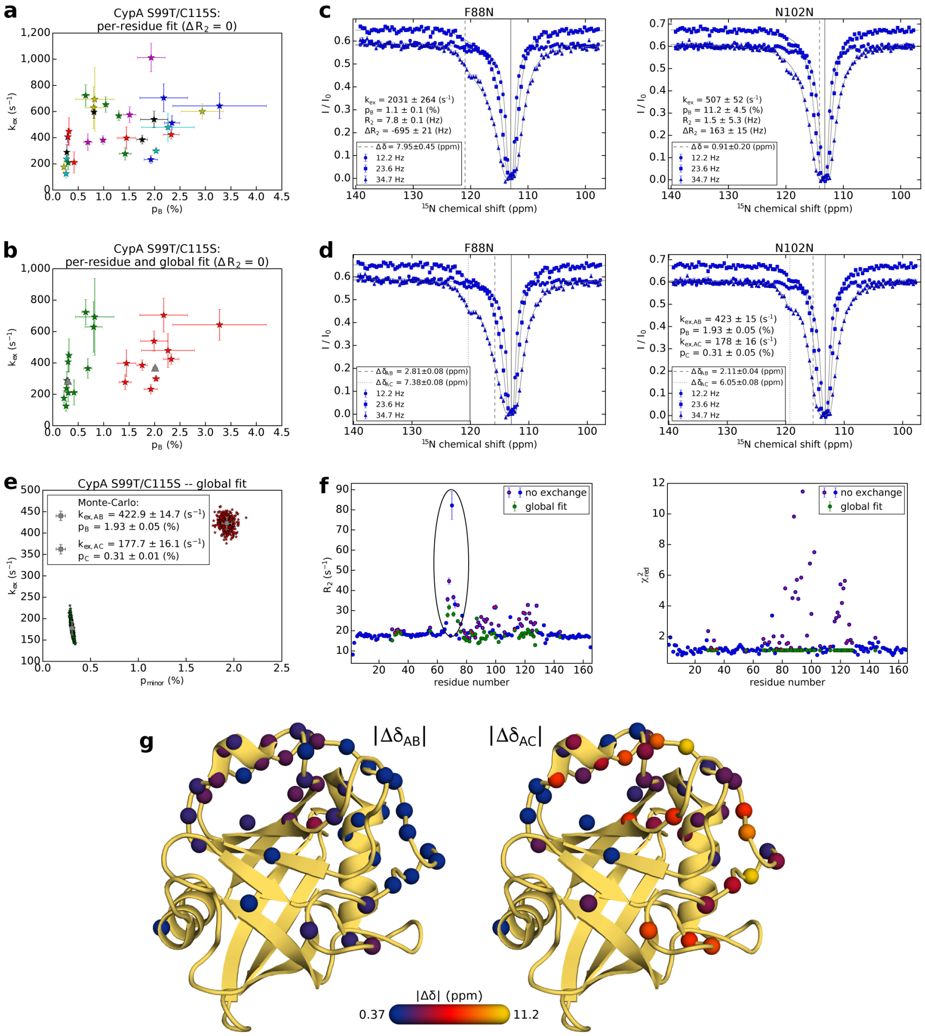

Supplementary Figure 4 | Analysis of the ^{15}N-CEST data for CypA S99T/S115S indicates the presence of more than one exchange process. (**a**) Per-residue fit of the CEST profiles for the initial 30 residues that clearly show slow exchange by visual inspection. Clustering of the k_{ex}/p_B values suggests the presence of two distinct exchange processes. (**b**) Residues that are well-described by a two-state exchange model were split into two clusters (*c.f.*, red (11 residues) and green (22 residues) spheres in Fig. 2e). Here, only the residues that are present in panel a and are used in the two-state global fit are shown together with their respective global k_{ex}/p_B values (grey triangles). (**c-d**) Several residues, including F88 and N102, do not fit to a two-site exchange process even if all fitting parameters are allowed to float (**c**). However, a three-site exchange model can explain the experimental data (**d**) and the vales of k_{ex}/p_{minor} correspond well to the two observed clusters assuming a single two-site exchange model (panel b). (**e**) Uncertainties in the fitting parameters for the three-state global fit that include all 46 residues were determined from 400 Monte-Carlo simulations and the resulting scatter plot for k_{ex} vs. p_{minor} is shown. The values and their uncertainties obtained using this method and from the covariance matrix are nearly identical. (**f**) Residue-specific values of R_2 and χ^2_{red} assuming there is no exchange (blue) or a global, three-site process (green). For ease of comparison, the 46 residues that experience exchange and are included in the global fit are also shown with a red marker for the no-exchange model. Fitting a no-exchange model shows significantly elevated R_2 and χ^2_{red} values for a large number of residues. After fitting an appropriate exchange model R_2 and χ^2_{red} drop to expected values, with the exception of the loop region (residues 65-80), where elevated R_2 values are still observed, consistent with line broadening of the ground-state signal due to dynamics on the millisecond timescale. (**g**) The chemical shift differences between the states, $|\Delta\delta_{AB}|$ (left panel) and $|\Delta\delta_{AC}|$ (right panel), for the 46 residues are plotted on the structure of CypA.

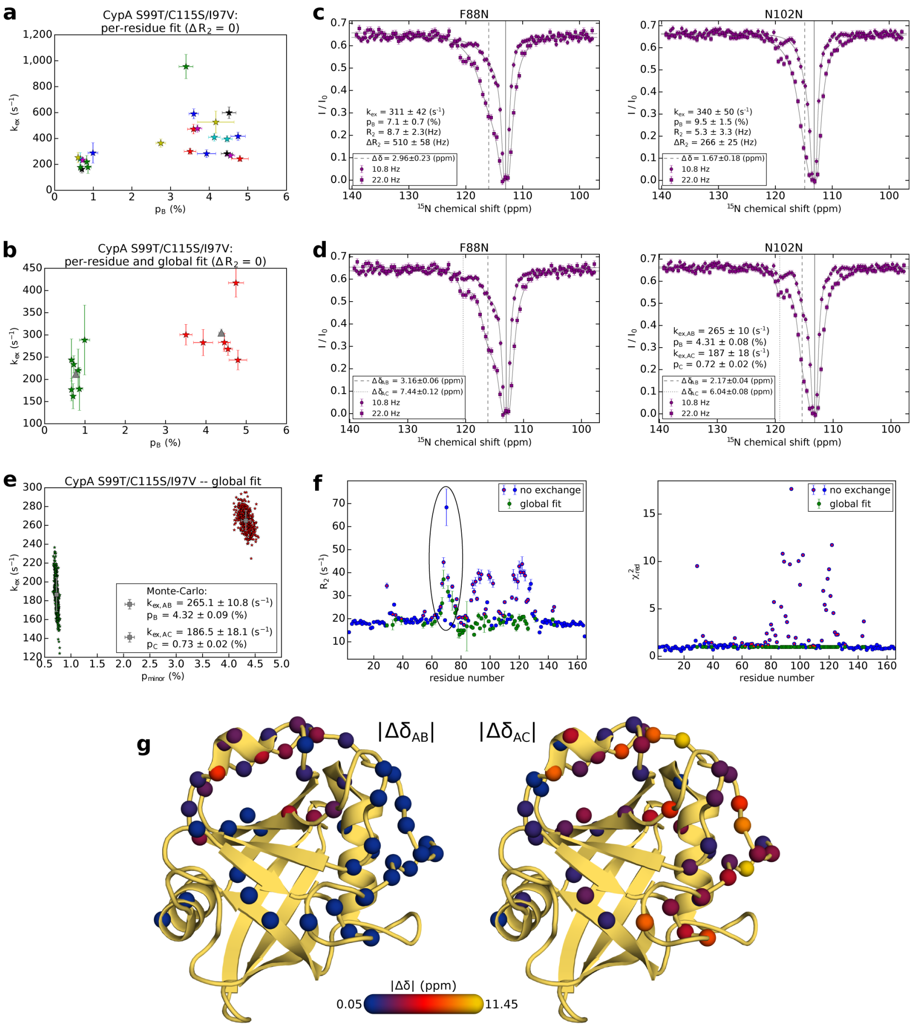

Supplementary Figure 5 | Analysis of the ^{15}N-CEST data for CypA S99T/S115S/I97V indicates the presence of more than one exchange process. (**a**) Per-residue fit of the CEST profiles for the initial 23 residues that clearly show slow exchange by visual inspection. Clustering of the k_{ex}/p_B values suggest the presence of two distinct exchange processes. (**b**) Residues that are well-described by a two-state exchange model were split into two clusters (*c.f.*, red (12 residues) and green (25 residues) spheres in Fig. 2g). Here, only the residues that are in panel a and are used in the two-state global fit are shown together with their respective global k_{ex}/p_B values (grey triangles). (**c-d**) Several residues, including F88 and N102, do not fit to a two-site exchange process even if all fitting parameters are allowed to float (**c**). However, a three-site exchange model can explain the experimental data (**d**) and the vales of k_{ex}/p_{minor} correspond well to the two observed clusters assuming a single two-site exchange model (panel b). (**e**) Uncertainties in the fitting parameters for the three-state global fit that include all 55 residues were determined from 425 Monte-Carlo simulations and the resulting scatter plot for k_{ex} vs. p_{minor} is shown. The values and their uncertainties obtained using this method and from the covariance matrix are nearly identical. (**f**) Residue-specific values of R_2 and χ^2_{red} assuming there is no exchange (blue) or a global, three-site process (green). For ease of comparison, the 55 residues that experience exchange and are included in the global fit are also shown with a red marker for the no-exchange model. Fitting a no-exchange model shows significantly elevated R_2 and χ^2_{red} values for a large number of residues. After fitting an appropriate exchange model R_2 and χ^2_{red} drop to expected values, with the exception of the loop region (residues 65-80), where elevated R_2 values are still observed, consistent with line broadening of the ground-state signal due to dynamics on the millisecond timescale. (**g**) The chemical shift differences between the states, $|\Delta\delta_{AB}|$ (left panel) and $|\Delta\delta_{AC}|$ (right panel), for the 55 residues are plotted on the structure of CypA.

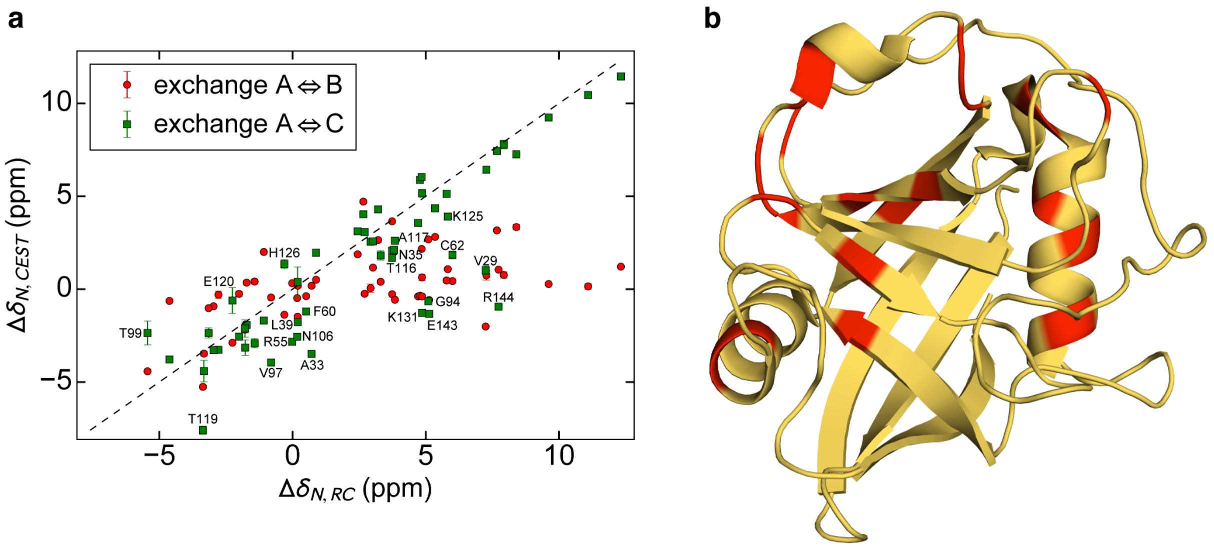

Supplementary Figure 6 | Correlation between ^{15}N Δδ values obtained from CEST and those between the major state and predicted random coil chemical shifts of CypA S99T/C115S/I97V. (**a**) ^{15}N chemical shift differences between state A↔B (red circles) and A↔C (green squares) were extracted from the 3-state global fit of the CEST profiles, and the random coil chemical shifts were predicted using the method of Tamiola *et al.*[2]. There is no clear correlation between $\Delta\delta_{N,AB}$ and $\Delta\delta_{N,RC}$ (pairwise rmsd = 4.2 ppm). On the contrary, the correlation between $\Delta\delta_{AC}$ and $\Delta\delta_{RC}$ suggests that minor state C corresponds to a more extended/unfolded conformation. Residues that are labeled with their assignment are not correlated ($|\Delta\delta_{N,AC}-\Delta\delta_{N,RC}| \geq 1.5$ ppm), indicating that these do not sample an extended conformation, and are color in red on the structure (**b**). The majority of these residues belong to group-I or are part of the dynamic network that is involved in the catalysis. The pairwise rmsd for $\Delta\delta_{N,AC}$ and $\Delta\delta_{N,RC}$ is 2.6 ppm when considering all residues and 0.9 ppm excluding the labeled ones that are part of the dynamic network.

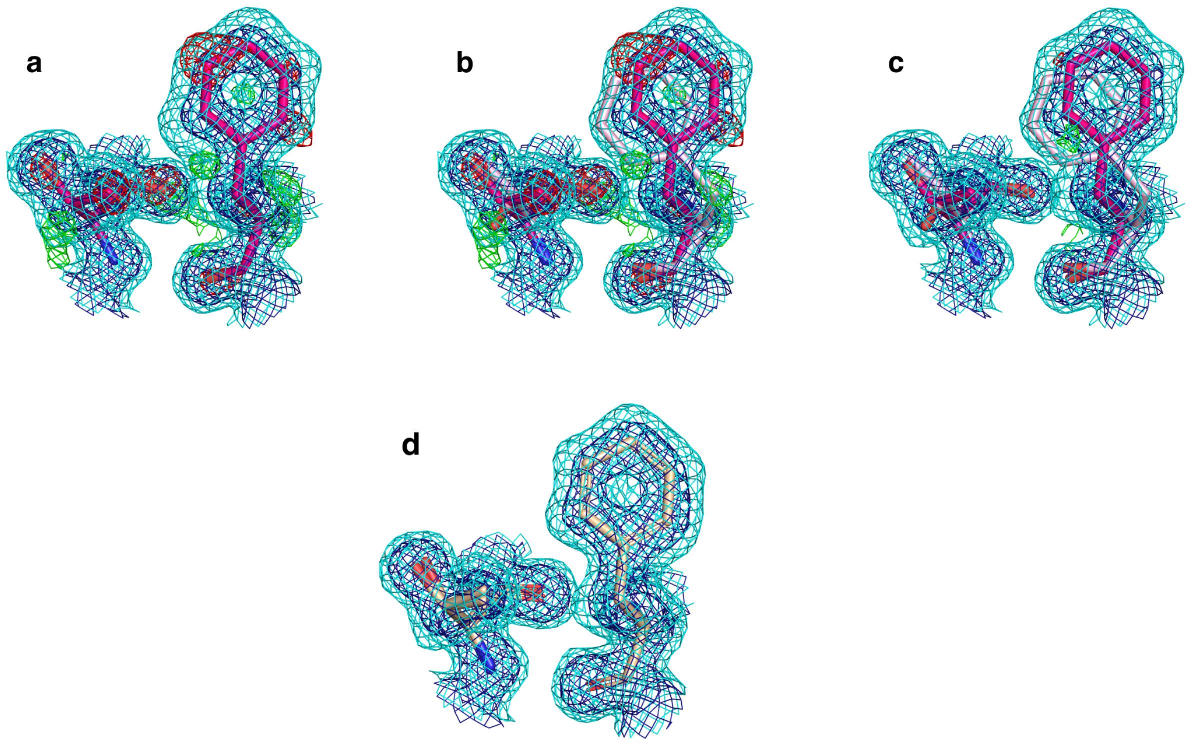

Supplementary Figure 7 | Difference electron density for alternative conformations of Thr99 and Phe113 in CypA S99T/C115S/I97V. (**a**) Negative and positive difference peaks in mFo-DFc electron density (±3.0σ in green and red) are contained within expanded 2mFo-DFc electron density (2.0σ in dark blue and 0.3σ in cyan), and not accounted for by a single conformer model (magenta) of CypA S99T/C115S/I97V. (**b**) The electron density maps as shown in (a) with the refined qFit multiconformer model (alternative conformations in magenta, ~0.8 occupancy, and light pink, ~0.2 occupancy) that explains the difference features. (**c**) The refined qFit models shown in (b) with the final 2mFo-DFc (2.0σ in dark blue and 0.3σ in cyan) and mFo-DFc maps (±3.0σ in green and red). (**d**) The electron density maps as shown in (a) for the refined S99T/C115S model lack the expanded density and difference peaks.

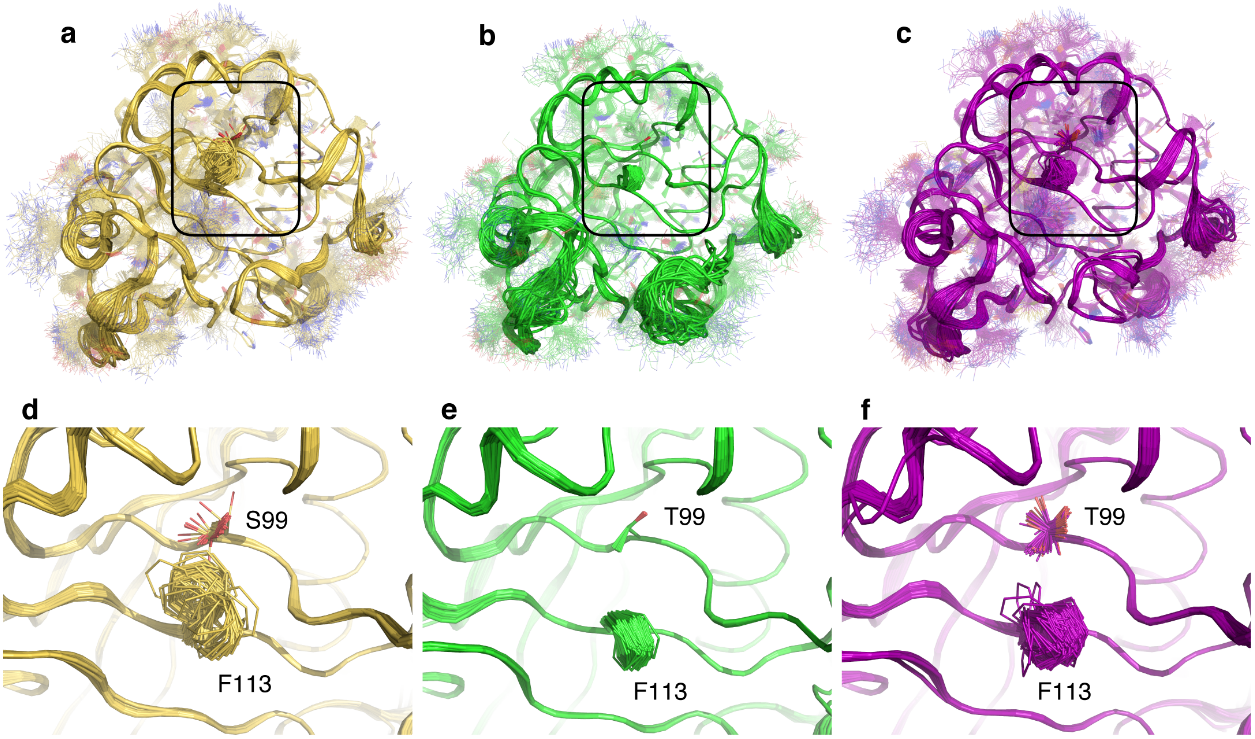

Supplementary Figure 8 | Time-averaged ensemble refinement for wild-type CypA (yellow), S99T (green), and S99T/C115S/I97V (purple). All refinements were performed using phenix.ensemble refinement[3] with parameters (pTLS, wxray, tx) selected based on lowest R_{free}. Overall view (**a-c**) and corresponding zoom-in (**d-f**) for wild-type CypA (**a,d**) (refined with pTLS 0.775, wxray 8.125, and tx 2.0) shows extensive side chain heterogeneity in the active site extending to the core through residues Phe113 and Ser99 (shown in sticks); S99T (**b,e**) (refined with pTLS 0.775, wxray 8.125, and tx 2.0) shows no heterogeneity in Phe113 and Thr99; and S99T/C115S/I97V (**c,f**) (refined with pTLS 0.55, wxray 8.125, and tx 2.0) shows increased heterogeneity in Phe113 and multiple conformations of Thr99 relative to the S99T.

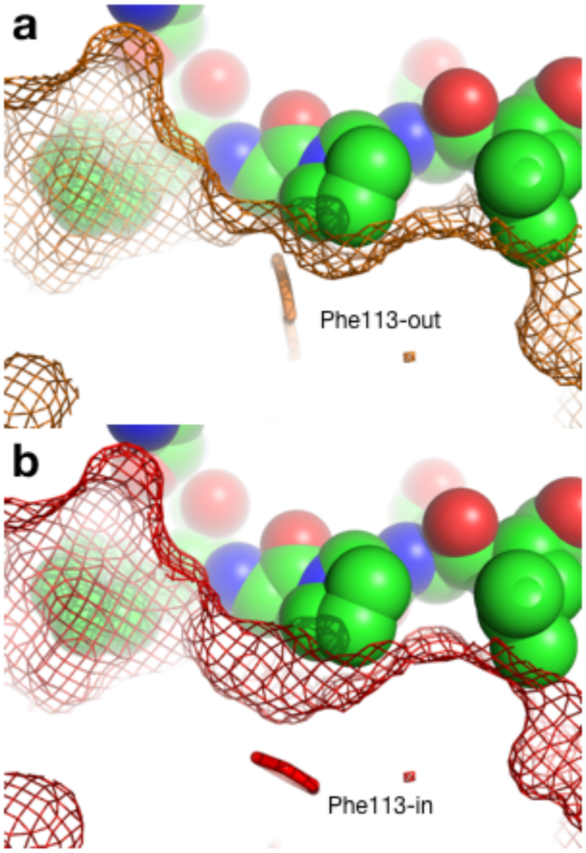

Supplementary Figure 9 | The triple mutant restores the alternative conformation of Phe113, which can make distinct complementary interactions with the trans-substrate. (**a**) The tight surface complementarity of CypA surface (shown in mesh) for the Phe-out conformation (orange) overlaid with the *trans*-substrate model from an NMR ensemble[4]. (**b**) In contrast, the Phe-in state (red) leaves a void and has poorer surface complementarity to the substrate. These modeled results suggest that the transition between different conformations of Phe113 may stabilize distinct substrate states.

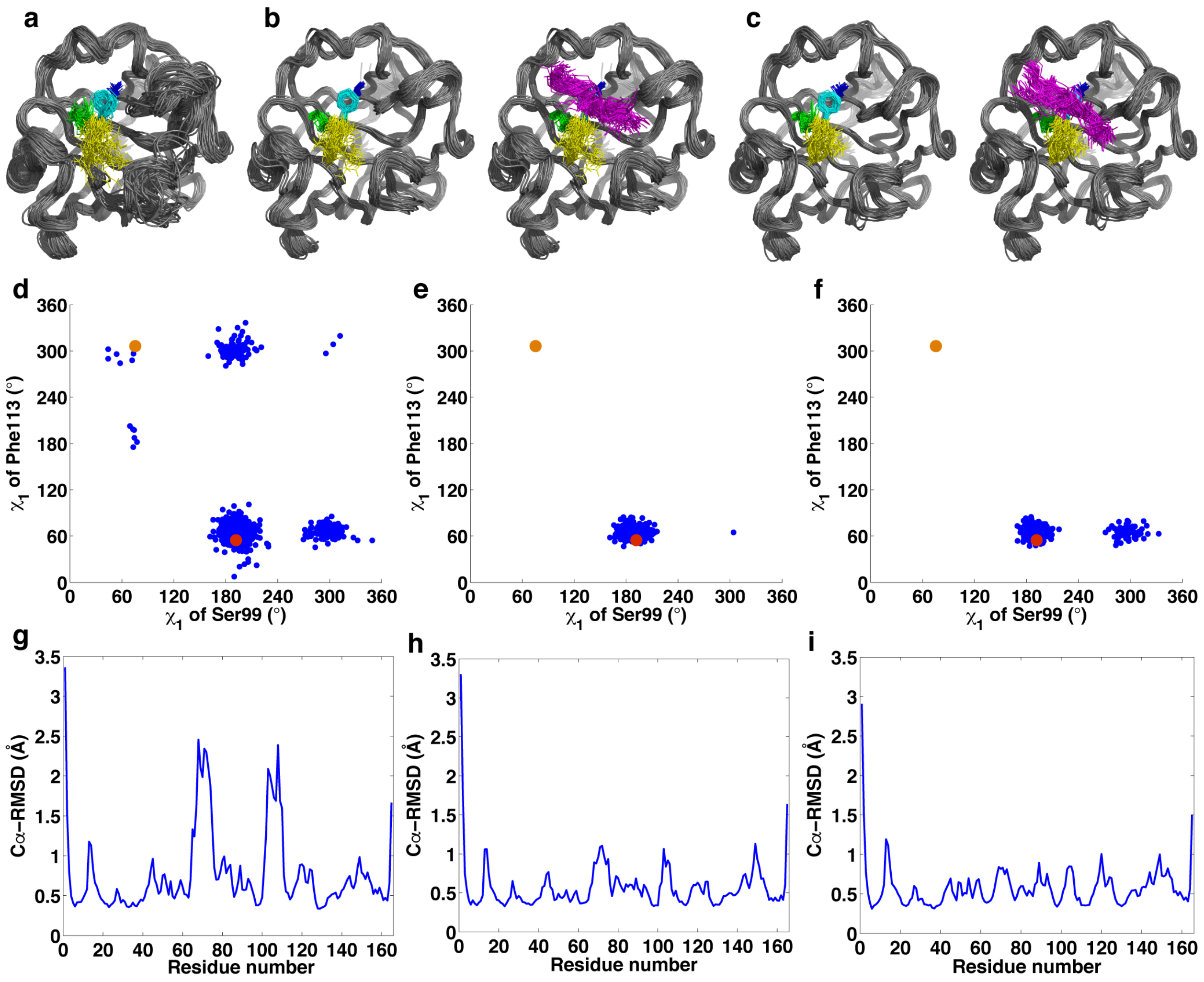

Supplementary Figure 10 | Analysis of conformational ensembles calculated by Camilloni *et al.* using DFT calculations to propose a catalytic mechanism[4]. Conformational heterogeneity is suppressed in the catalytic complexes, and alternate conformations seen in the X-ray structures[5] are not sampled in the simulation with the substrates bound. (**a-c**) A subset of 68 randomly selected ensemble members is shown in cartoon with residues Ser99 (blue), Phe113 (cyan), Met61 (green), and Arg55 (yellow) shown in sticks. The apo protein ensemble (**a**) shows more conformational heterogeneity than the peptide (GSFGPDLRAGD) substrate-bound forms with a Gly-Pro in cis- (**b**) and trans- (**c**) conformations, shown without (left) or with (right) the substrate backbone (magenta). Curiously, their cis- and trans-peptide complexes show only minimal protein conformational differences compared to the starting crystal structures, while the free CypA showed multiple substates in their simulations. This is reflected in the dihedral angle distribution for Ser99 and Phe113 for apo wild-type CypA (**d**) versus the cis- (**e**) and trans- (**f**) substrate bound ensembles. The two alternative (major and minor) conformations seen in the room-temperature X-ray structure of wild-type CypA (3K0N[5]) are shown in as red and orange dots for reference, respectively. Their result is in sharp contrast with our NMR dynamics for WT[6] and S99T during catalysis measured here, which clearly shows that conformational substates interconvert across the core catalytic network and that this rate is correlated to catalysis. Although no significant change in loop dynamics is detected by NMR during catalysis, the MD simulations indicate larger deviations in the C^{α} RMSDs in the apo protein (**g**) is specifically decreased in the loops surrounding residues 60-80 and 100-120 in the cis- (**h**) and trans- (**i**) substrate bound ensembles. New simulations that incorporate the side-chain dynamics characterized here may help bridging their DFT computational approach which emphasize a key role for electrostatics, with our experimental results that deliver the protein conformational substates.

Supplementary Table 1 | Data collection and refinement statistics for CypA S99T/C115S/I97V

The number of crystals for each structure is 1.
[*] Values in parentheses are for highest-resolution shell.

	CypA S99T/C115S/I97V (5WC7)
**Data collection**	
Space group	P212121
Cell dimensions	
a, *b*, *c* (Å)	43.0, 52.4, 89.2
α, β, γ (°)	90, 90, 90
Resolution (Å)	33.97 – 1.43 (1.48 - 1.43)[*]
R_{merge}	0.031 (0.482)
$I/\sigma(I)$	18.7 (1.1)
$CC_{1/2}$	1 (0.829)
Completeness (%)	0.99 (0.96)
Redundancy	3.81 (2.37)
**Refinement**	
Resolution (Å)	33.21 - 1.43
No. reflections	37777 (3735)
R_{work} / R_{free}	0.1031/0.1285
No. atoms	
Protein	2023
Water	229
B factors	
Protein	19.88
Water	43.71
R.m.s. deviations	
Bond lengths (Å)	0.010
Bond angles (°)	1.26

Supplementary Table 2 | Data collection and refinement statistics for CypA S99T/C115S

The number of crystals for each structure is 1.
[*] Values in parentheses are for highest-resolution shell.

	CypA S99T/C115S (6BTA)
**Data collection**	
Space group	P212121
Cell dimensions	
a, b, c (Å)	42.9, 52.5, 89.3
α, β, γ (°)	90, 90, 90
Resolution (Å)	25.9 – 1.48 (1.53 - 1.48)[*]
R_{merge}	0.042 (0.488)
$I/\sigma(I)$	13.6 (2.4)
$CC_{1/2}$	1 (0.795)
Completeness (%)	0.93 (0.96)
Redundancy	3.1 (3.0)
**Refinement**	
Resolution (Å)	25.9 – 1.48
No. reflections	32055 (3250)
R_{work} / R_{free}	0.1267/0.1492
No. atoms	
Protein	1987
Water	164
B factors	
Protein	20.18
Water	41.47
R.m.s. deviations	
Bond lengths (Å)	0.006
Bond angles (°)	1.02

Supplementary Table 3 | Primer sequences used for generating the CypA mutants

I97V mutation	
forward	5'– CTGGCGTCTTGACCATGGCAAATGCTGGAC –3'
reverse	5'– CCATGGTCAAGACGCCAGGACCCGTATGCTTTAGG –3'
C115S mutation	
forward	5'– GTTTTTCATCTCCACTGCCAAGACTGAGTGGTTGG –3'
reverse	5'– TCTTGGCAGTGGAGATGAAAAACTGGGAACCATTT –3'

Supplementary References

1 Mathematica (Wolfram Research, Inc., Champaign, Illinois, 2017).

2 Tamiola, K., Acar, B. & Mulder, F. A. Sequence-specific random coil chemical shifts of intrinsically disordered proteins. *J Am Chem Soc* **132**, 18000-18003, doi:10.1021/ja105656t (2010).

3 Burnley, B. T., Afonine, P. V., Adams, P. D. & Gros, P. Modelling dynamics in protein crystal structures by ensemble refinement. *Elife* **1**, e00311, doi:10.7554/eLife.00311 (2012).

4 Camilloni, C. *et al.* Cyclophilin A catalyzes proline isomerization by an electrostatic handle mechanism. *Proc Natl Acad Sci U S A* **111**, 10203-10208, doi:10.1073/pnas.1404220111 (2014).

5 Fraser, J. S. *et al.* Hidden alternative structures of proline isomerase essential for catalysis. *Nature* **462**, 669-673, doi:10.1038/nature08615 (2009).

6 Eisenmesser, E. Z. *et al.* Intrinsic dynamics of an enzyme underlies catalysis. *Nature* **438**, 117-121, doi:10.1038/nature04105 (2005).
