# Supplementary material for: Rescue of conformational dynamics in enzyme catalysis by directed evolution
Source: Nat Commun. 2018 Apr 3;9:1314. doi: 10.1038/s41467-018-03562-9 (PMC5883053; doi:10.1038/s41467-018-03562-9)
Supplement: Supplementary file 3 — Description of Additional Supplementary Files [file 41467_2018_3562_MOESM3_ESM.pdf]

Below are the legends for each Supplementary Data files:

Supplementary Data 1 | ^{15}N-CPMG relaxation dispersion profiles for CypA S99T/C115S recorded at 10 (blue) and 15 (green) °C on a 600 MHz spectrometer. The time scale of the exchange process experienced by each residue was determined based on the temperature dependence and/or shape of the profile and is color-coded on the structure in Figure 2e. Profiles are normalized to the lowest temperature using the average of the three highest ν_{CPMG} field strengths. Residues that are in the slow-exchange regime show different apparent R_{ex} values, suggesting that there are two different exchange processes.

Supplementary Data 2 | ^{15}N-CPMG relaxation dispersion profiles for CypA S99T/C115S/I97V recorded at 10 (blue), 15 (green) and 20 (red) °C on a 600 MHz spectrometer. The time scale of the exchange process experienced by each residue was determined based on the temperature dependence and/or shape of the profile and is color-coded on the structure in Figure 2g. Profiles are normalized to the lowest temperature using the average of the three highest ν_{CPMG} field strengths. Residues that are in the slow-exchange regime show different apparent R_{ex} values, suggesting that there are two different exchange processes.

Supplementary Data 3 | Comparison of the ^{15}N-CPMG relaxation dispersion profiles for CypA S99T (green circles), S99T/C115S (blue squares) and S99T/C115S/I97V (purple triangles) recorded at 10 (left) and 15 (right) °C on a 600 MHz spectrometer. Profiles are normalized to the first CypA mutant shown by using the average of the three highest ν_{CPMG} field strengths. Profiles for residues in the ligand-binding loop, which are in the fast-exchange regime, are nearly identical for all the mutants, indicating that their exchange parameters are very similar.

Supplementary Data 4 | ^{15}N-CEST profiles of the 15 residues in CypA S99T undergoing exchange and the results of the global fit. Data was recorded at 10 °C on a 500 MHz spectrometer with two different $\mathbf{B}_1$ field strengths (circles and squares). The solid lines represent the best-fit with global parameters k_{ex} = 188.4 ± 9.1 s^{-1} and p_B = 1.2 ± 0.03% (residue-specific $\Delta\delta$ values are given in the legend); vertical lines indicate the position of the major (solid) and minor (dashed) state peaks.

Supplementary Data 5 | ^{15}N-CEST profiles of the 46 residues in CypA S99T/C115S undergoing exchange and the results of the global fit. Data was recorded at 10 °C on a 500 MHz spectrometer with three different $\mathbf{B}_1$ field strengths (circles, squares and triangles). The solid lines represent the best-fit with global parameters $k_{ex,AB}$ = 414.0 ± 14.7 s^{-1}, p_B = 1.94 ± 0.05%, $k_{ex,AC}$ = 207.9 ± 20.1 s^{-1}, p_C = 0.29 ± 0.01% (residue-specific $\Delta\delta$ values are given in the legend); vertical lines indicate the position of the major (solid) and minor (dashed/dotted) state peaks.

Supplementary Data 6 | ^{15}N-CEST profiles of the 55 residues in CypA S99T/C115S/I97V undergoing exchange and the results of the global fit. Data was recorded at 10 °C on a 500 MHz spectrometer with two different $\mathbf{B}_1$ field strengths (circles and squares). The solid lines represent the best-fit with global parameters $k_{ex,AB}$ = 251.7 ± 9.6 s^{-1}, p_B = 4.33 ± 0.08%, $k_{ex,AC}$ = 239.9 ± 22.4 s^{-1}, p_C = 0.68 ± 0.02% (residue-specific $\Delta\delta$ values are given in the legend); vertical lines indicate the position of the major (solid) and minor (dashed/dotted) state peaks.

Supplementary Data 7 | ^{15}N-CPMG relaxation dispersion profiles for CypA S99T with Suc-AFPF-pNA recorded at 10 (blue) and 15 (green) °C on a 600 MHz spectrometer. The time scale of the exchange process experienced by each residue was determined based on the temperature dependence and/or shape of the profile and is color-coded on the structure in Figure 3c. Profiles are normalized to the lowest temperature using the average of the three highest ν_{CPMG} field strengths.

Supplementary Data 8 | ^{15}N-CEST profiles of the 7 residues in CypA S99T with Suc-AFPF-pNA undergoing exchange and the results of the global fit. Data was recorded at 10 °C on a 500 MHz spectrometer with two different $\mathbf{B}_1$ field strengths (circles and squares). The solid lines represent the best-fit with global parameters k_{ex} = 118.3 ± 37.0 s^{-1} and p_B = 2.5 ± 0.27% (residue-specific $\Delta\delta$ values are given in the legend); vertical lines indicate the position of the major (solid) and minor (dashed) state peaks.
