# Supplementary figures and images for: Rescue of conformational dynamics in enzyme catalysis by directed evolution
Source: Nat Commun. 2018 Apr 3;9:1314. doi: 10.1038/s41467-018-03562-9 (PMC5883053; doi:10.1038/s41467-018-03562-9)

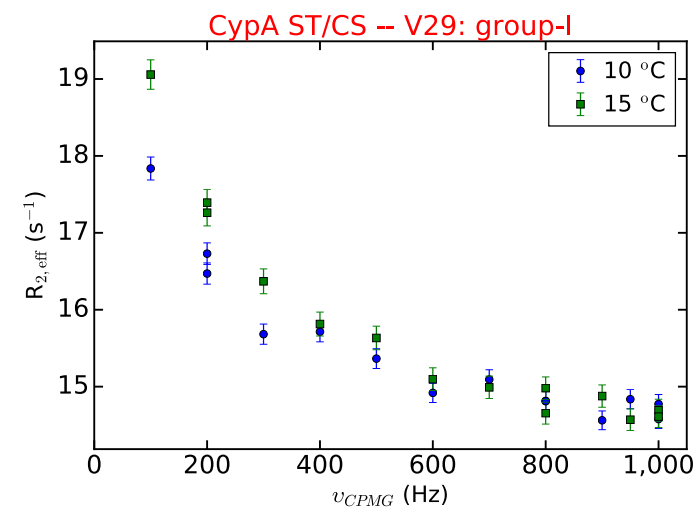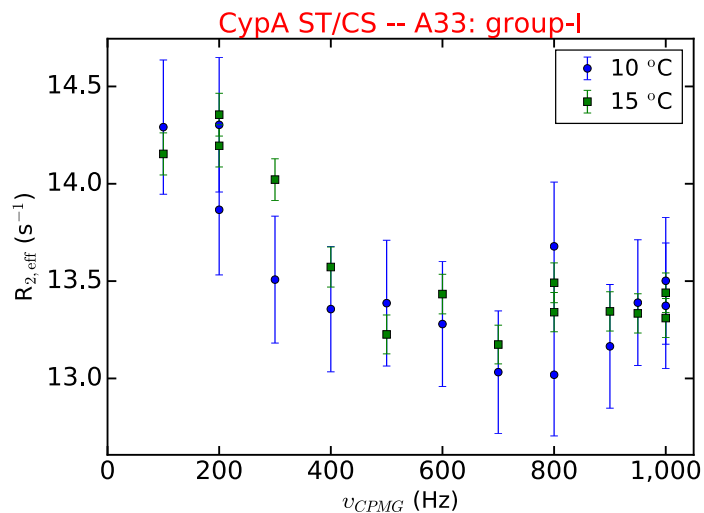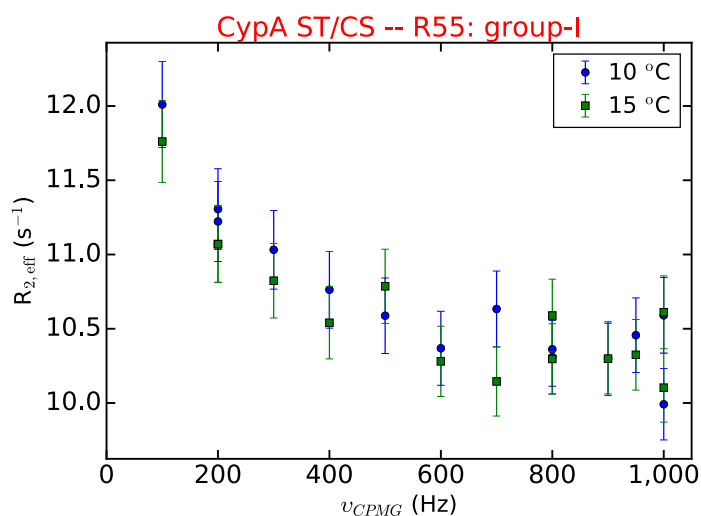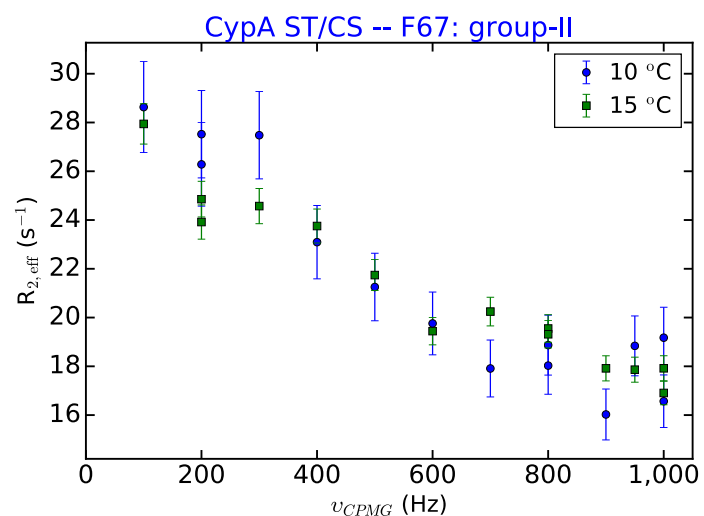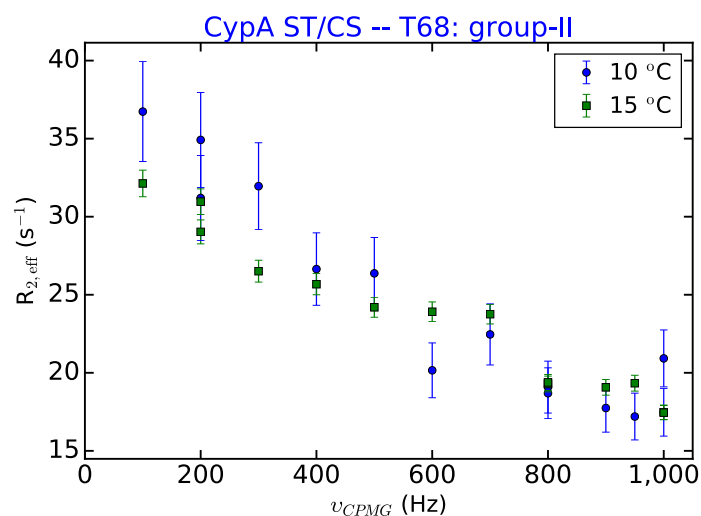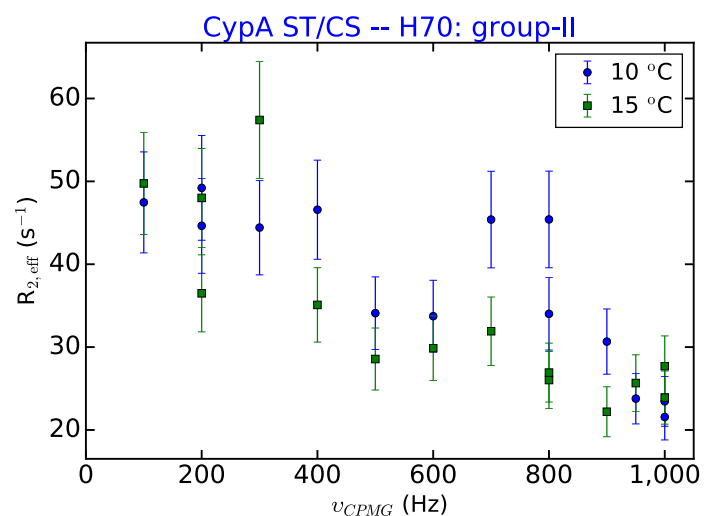

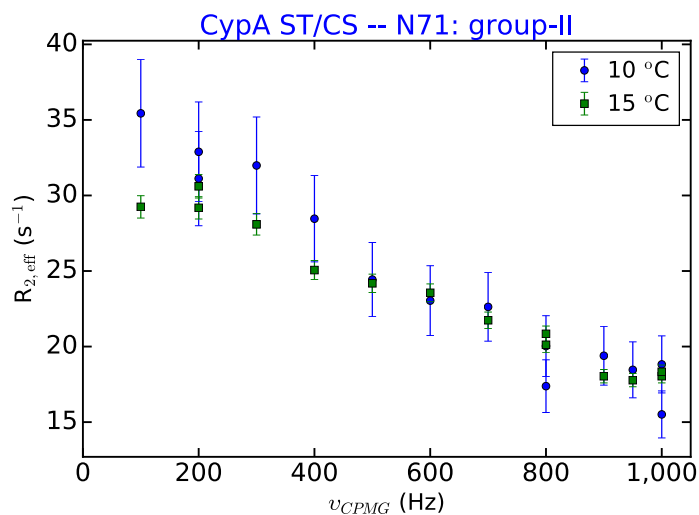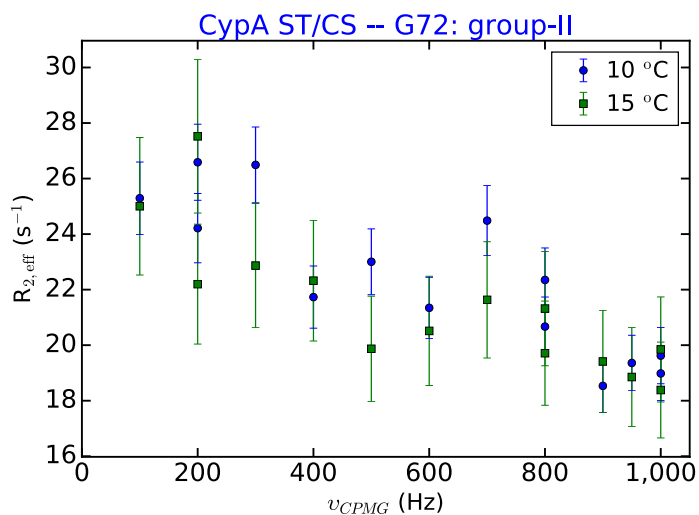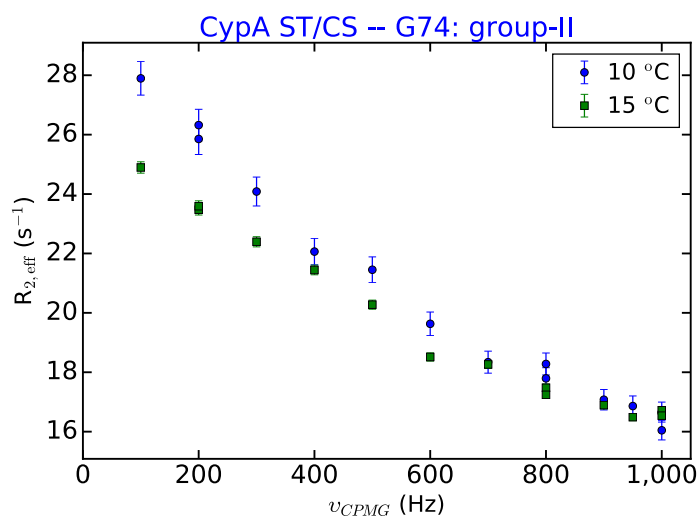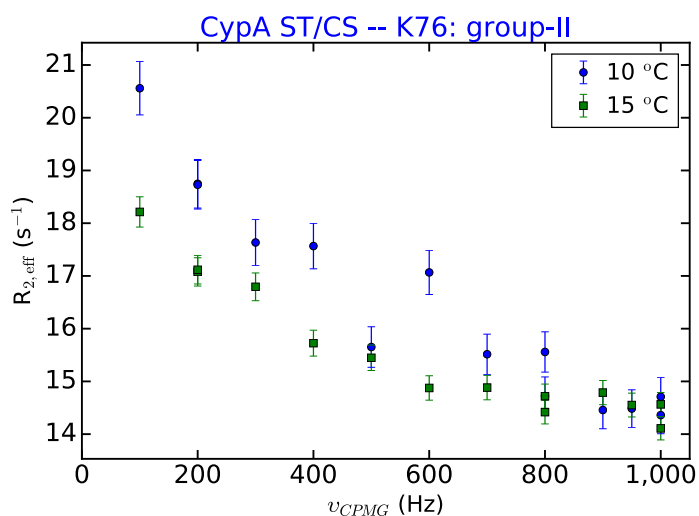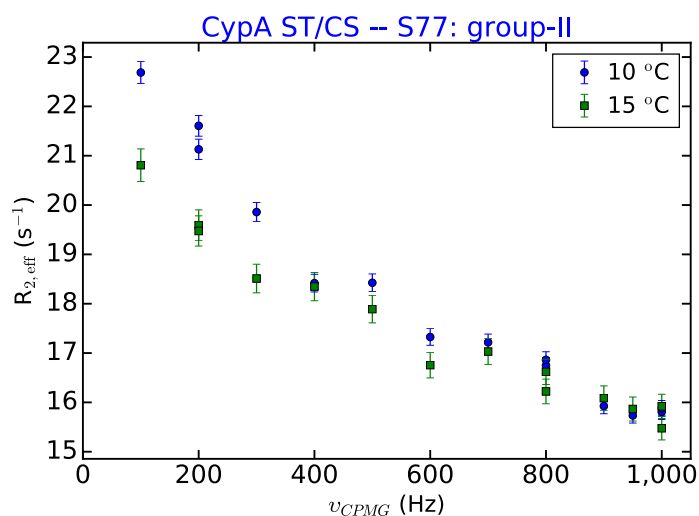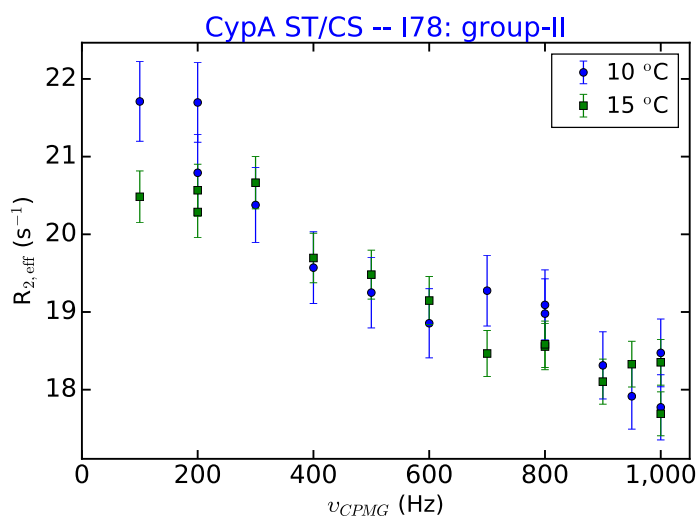

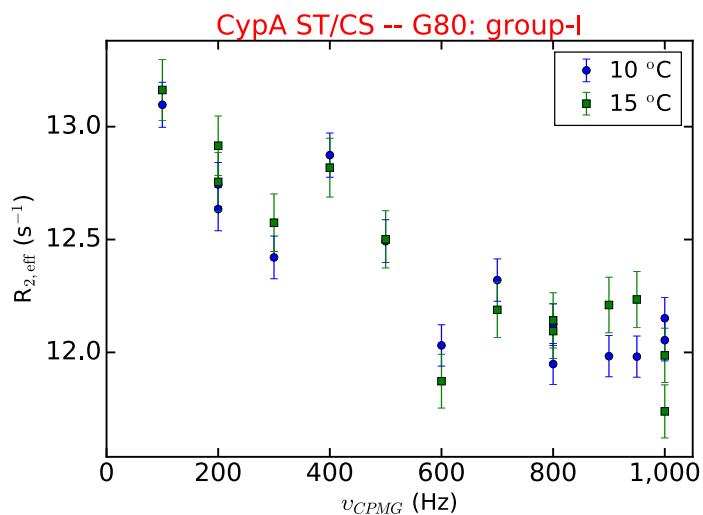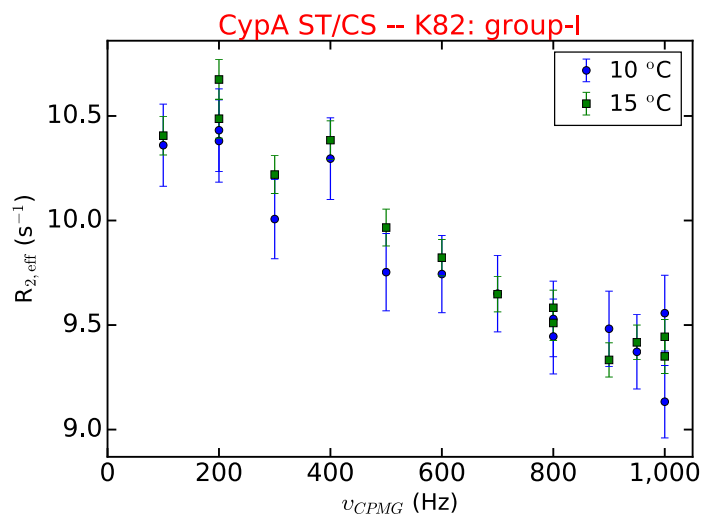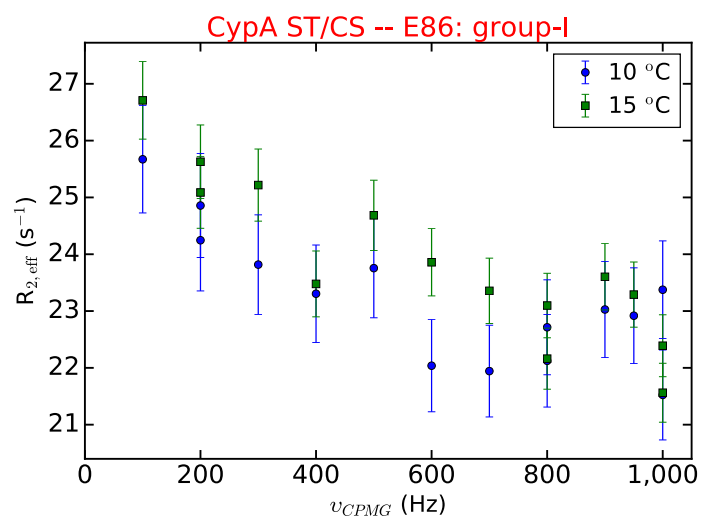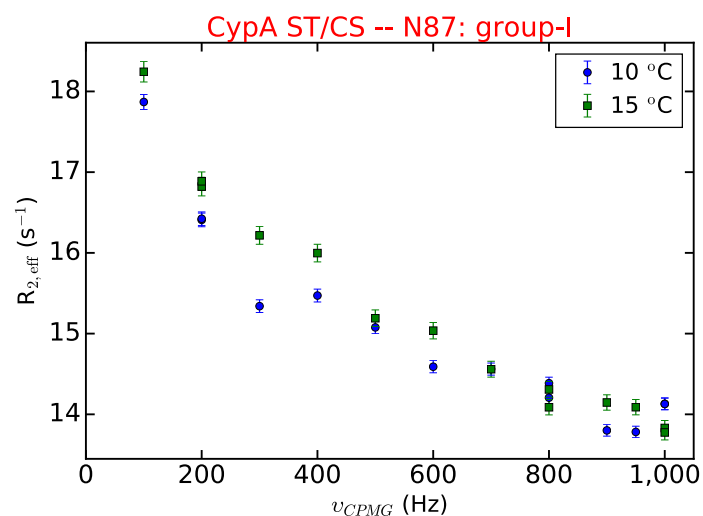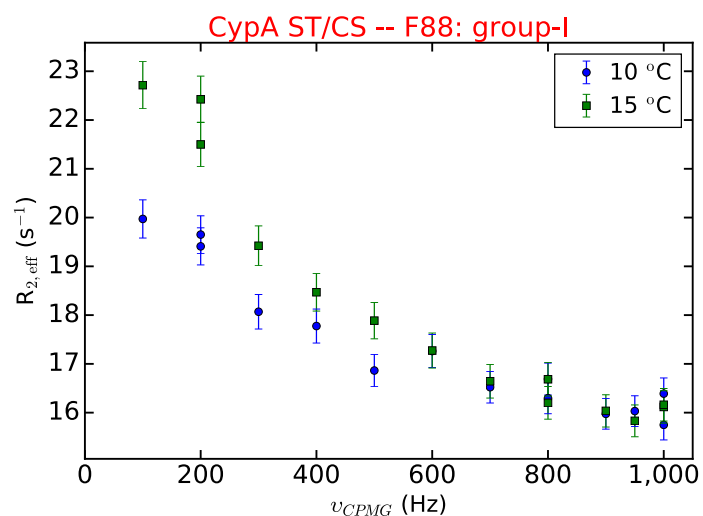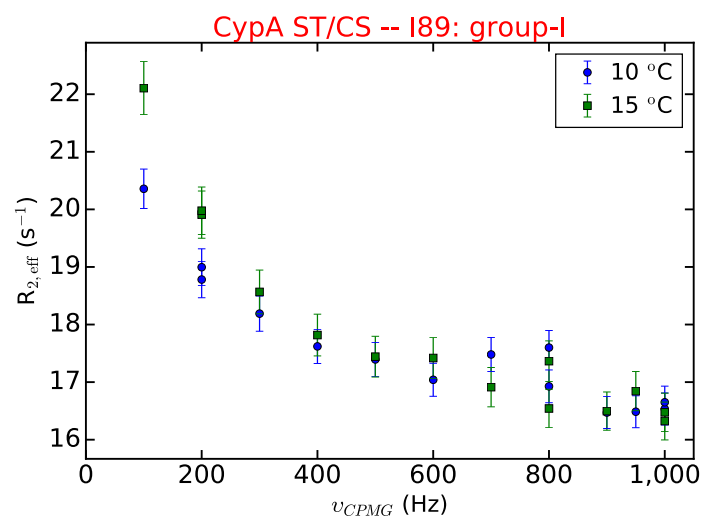

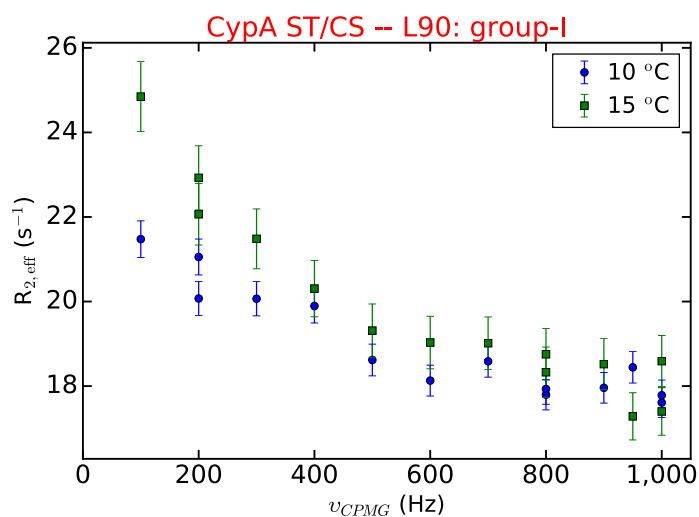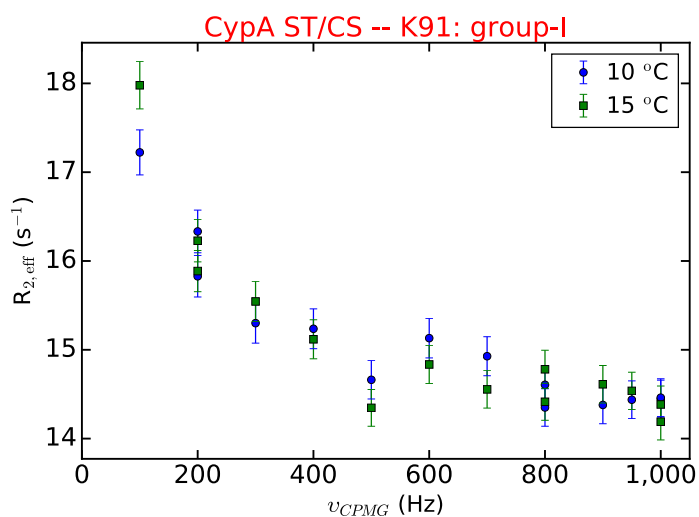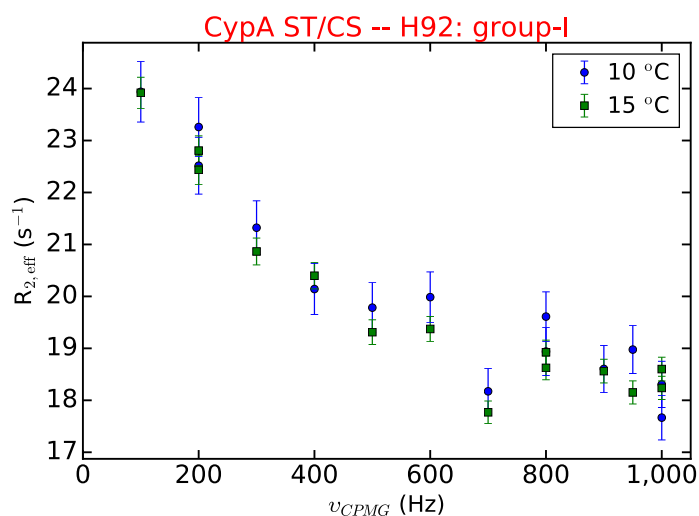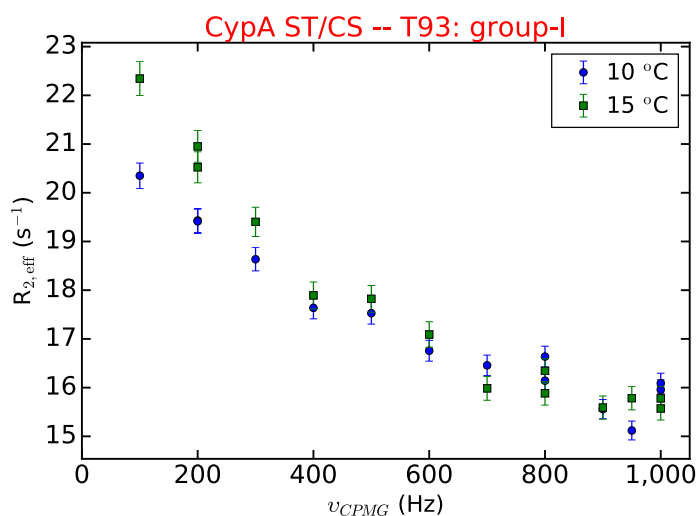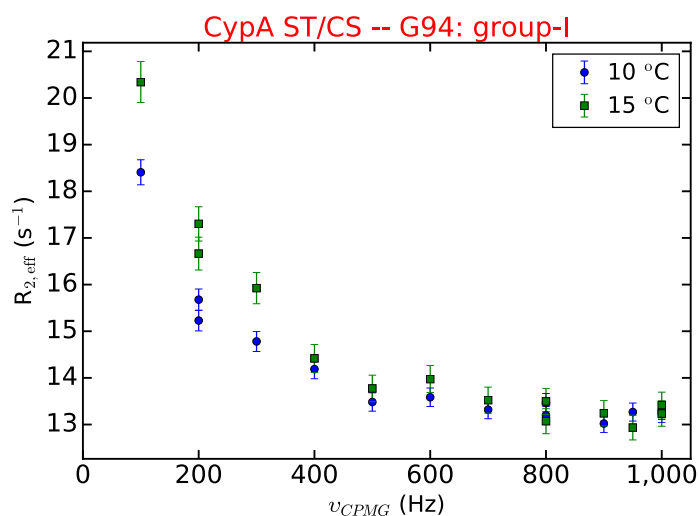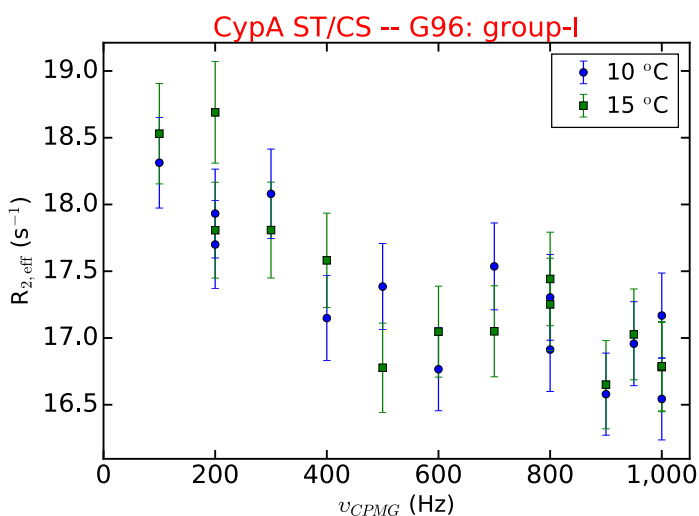

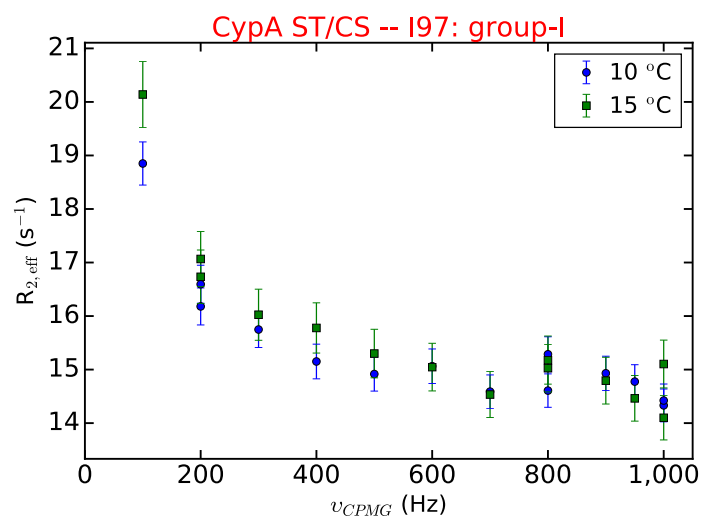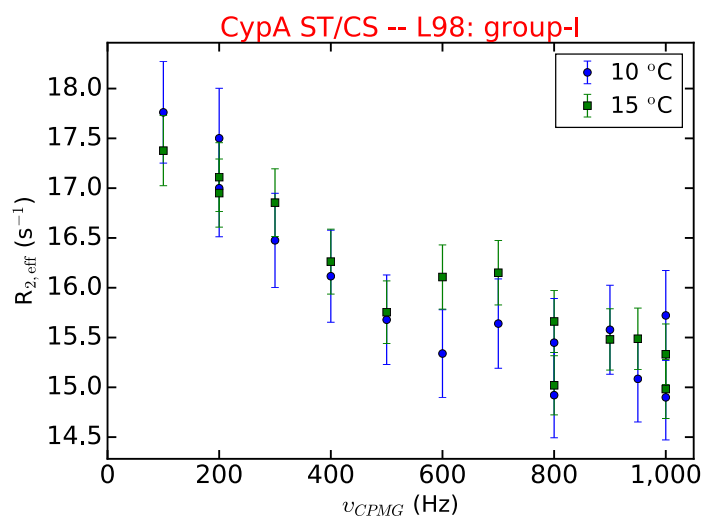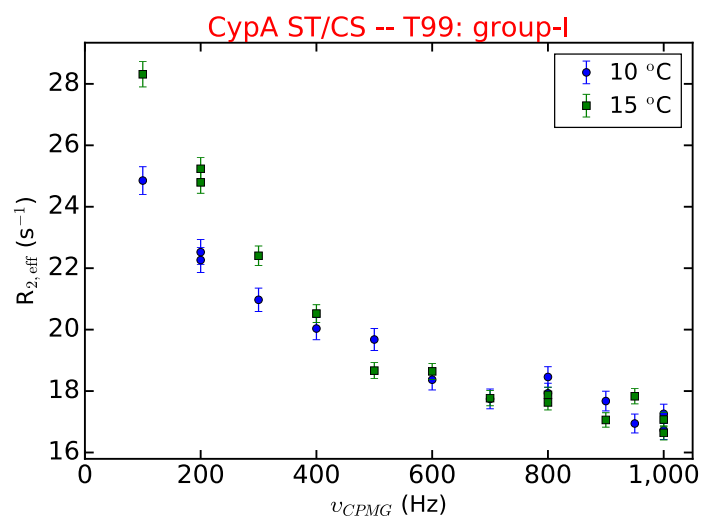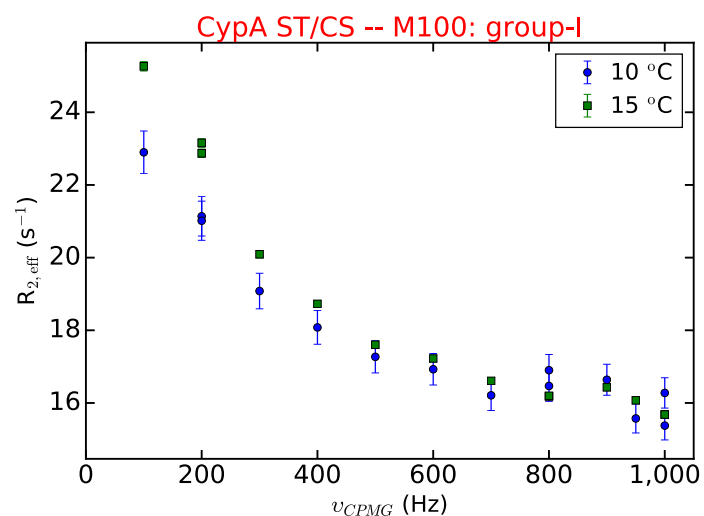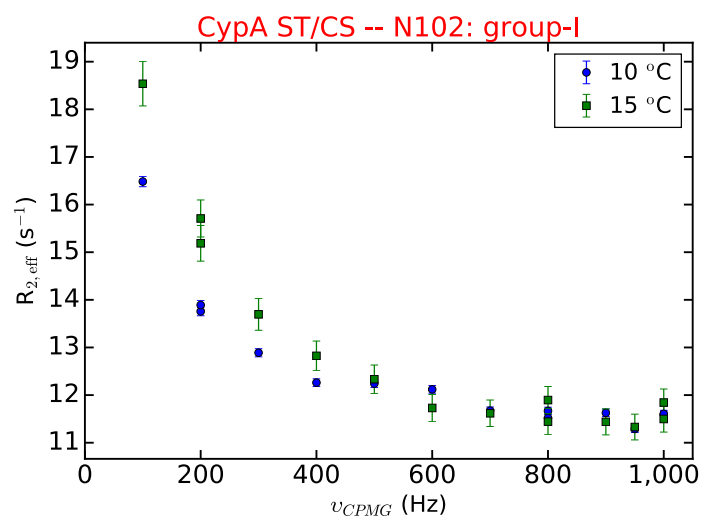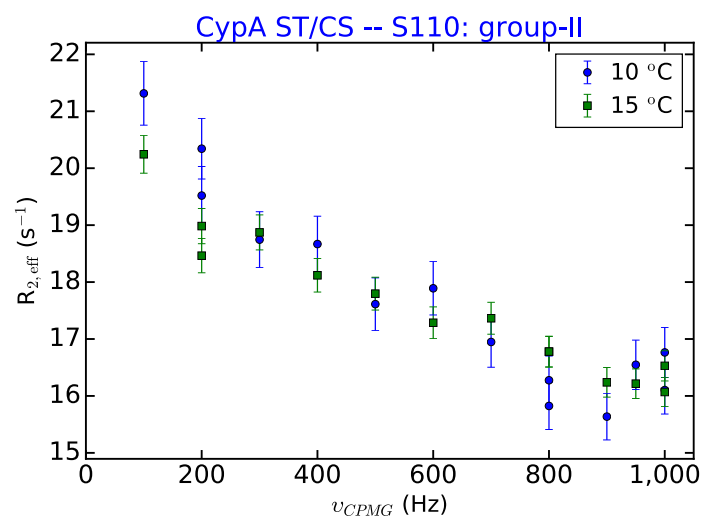

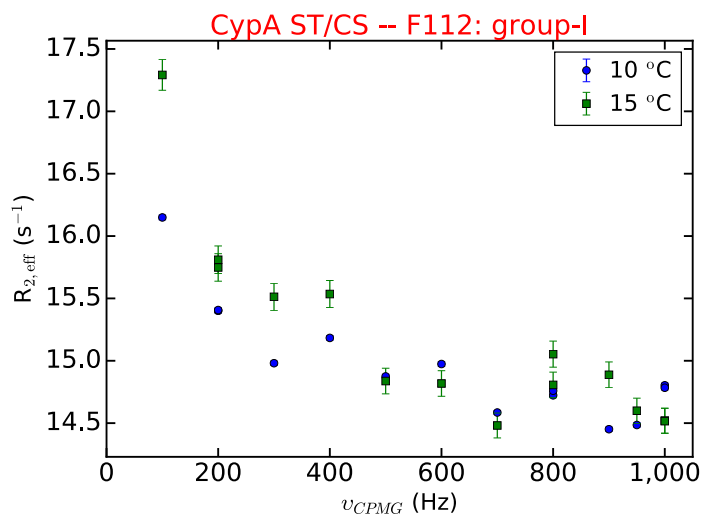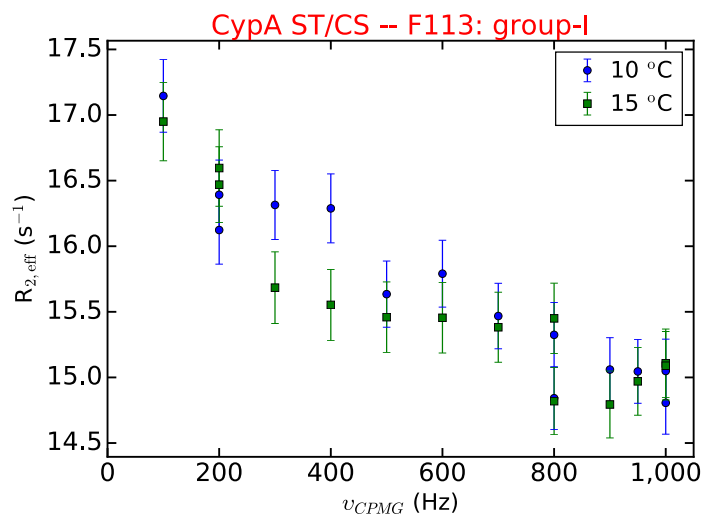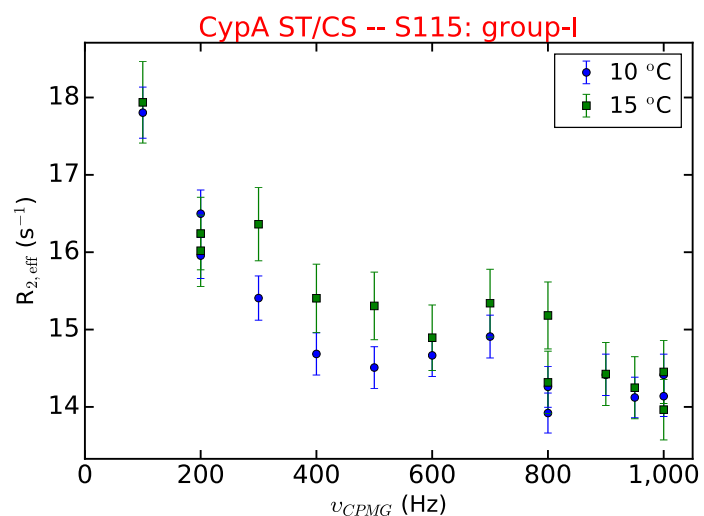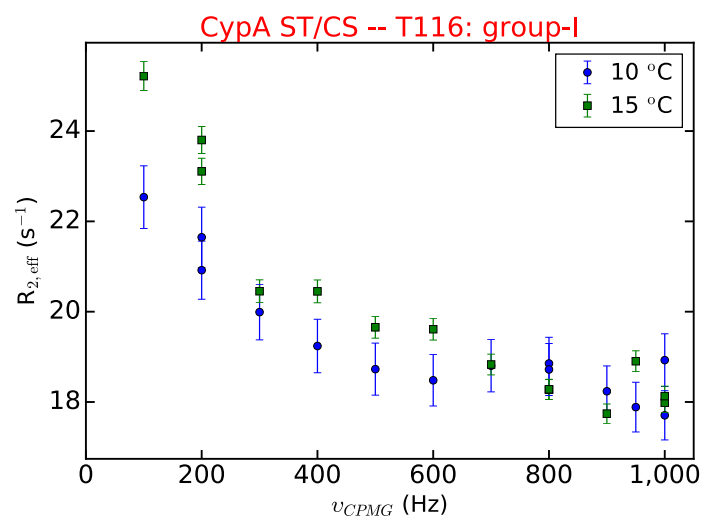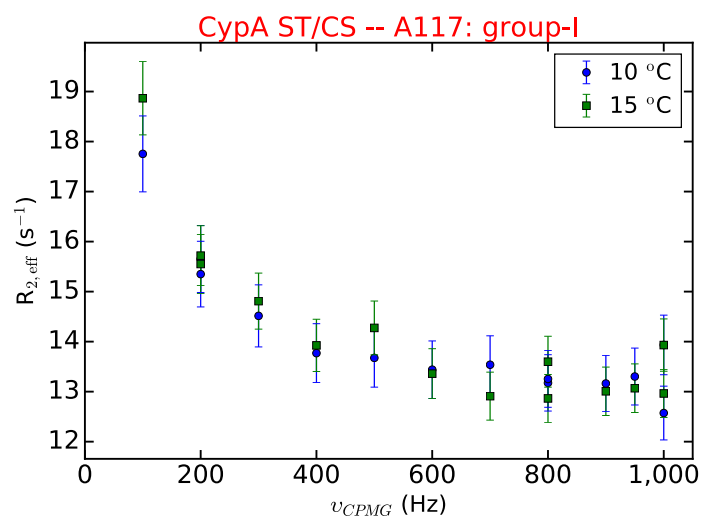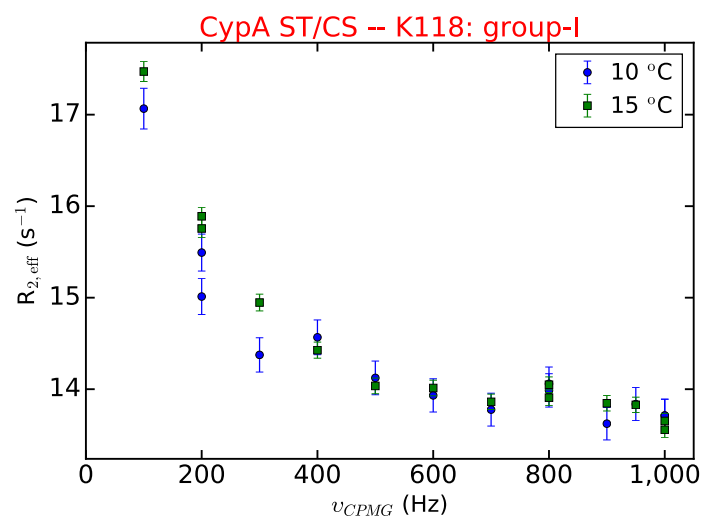

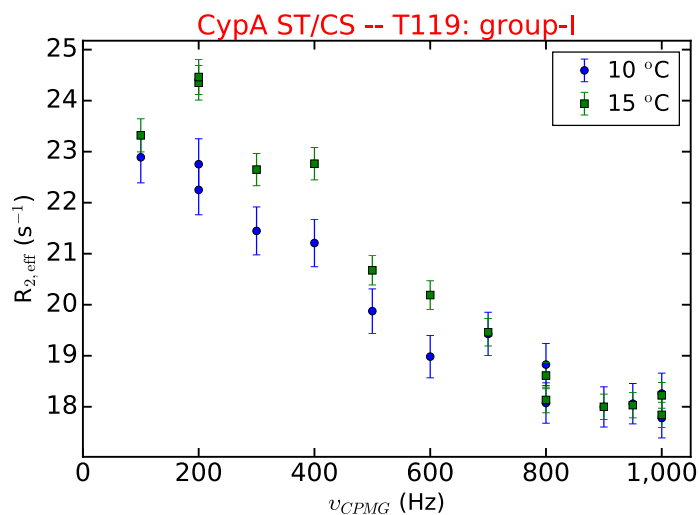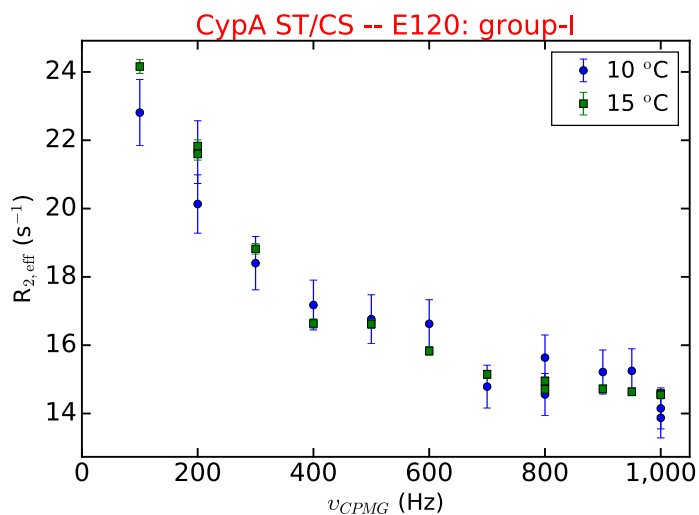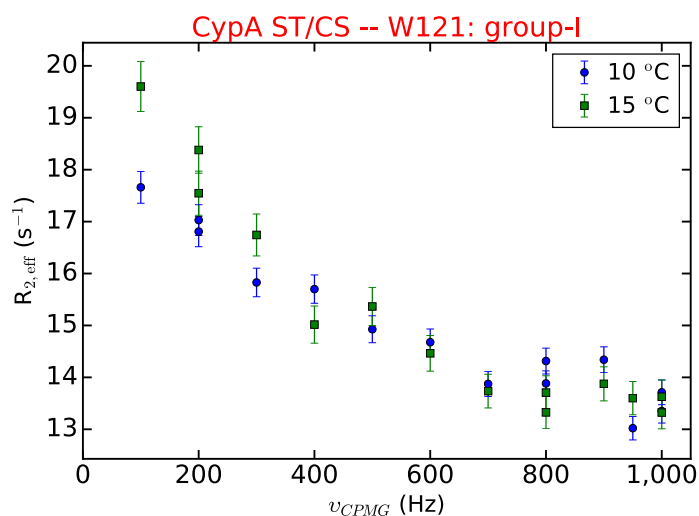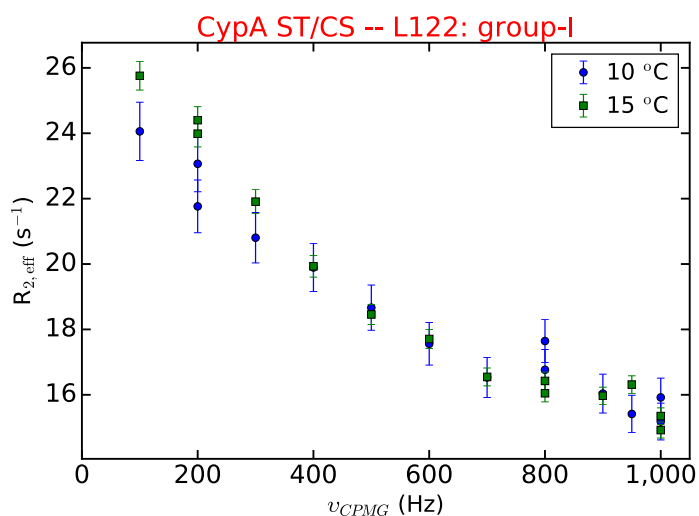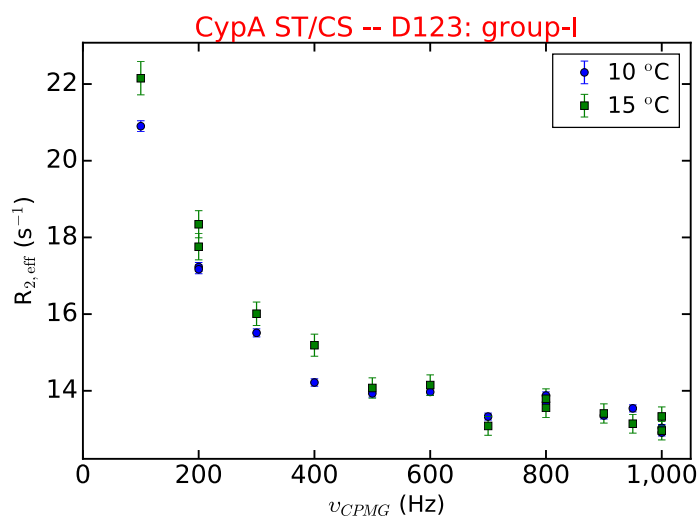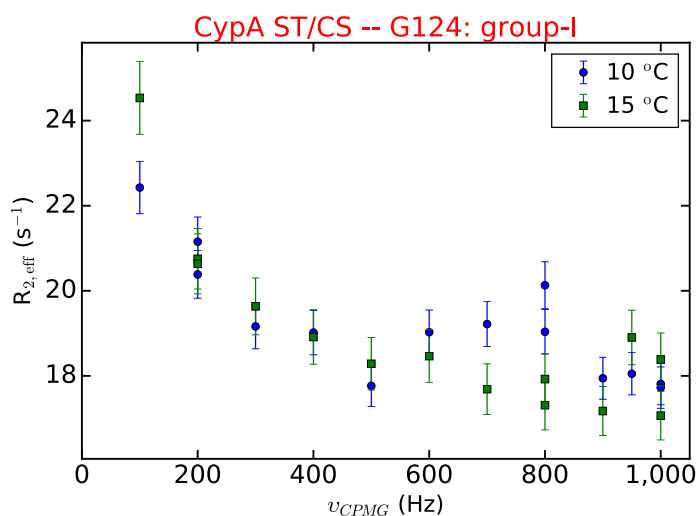

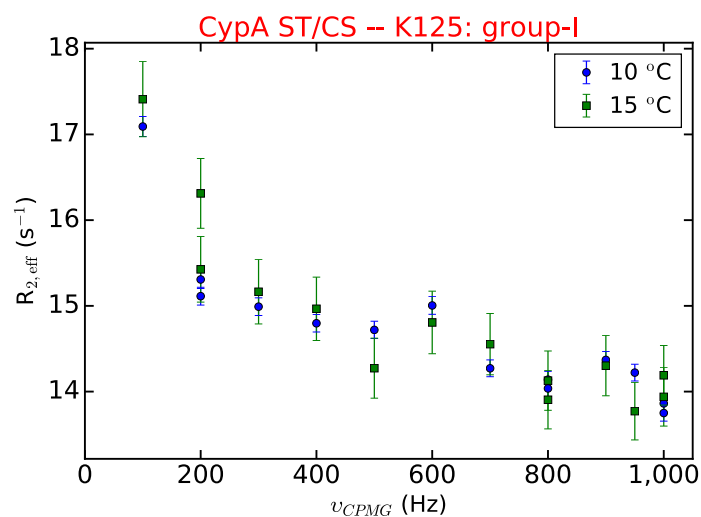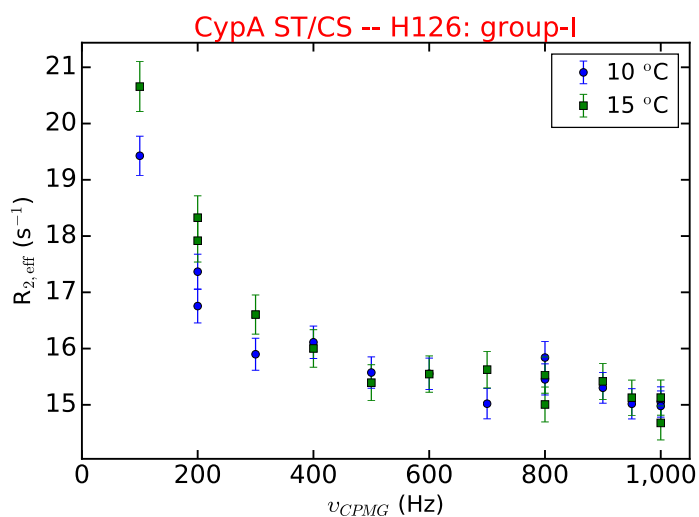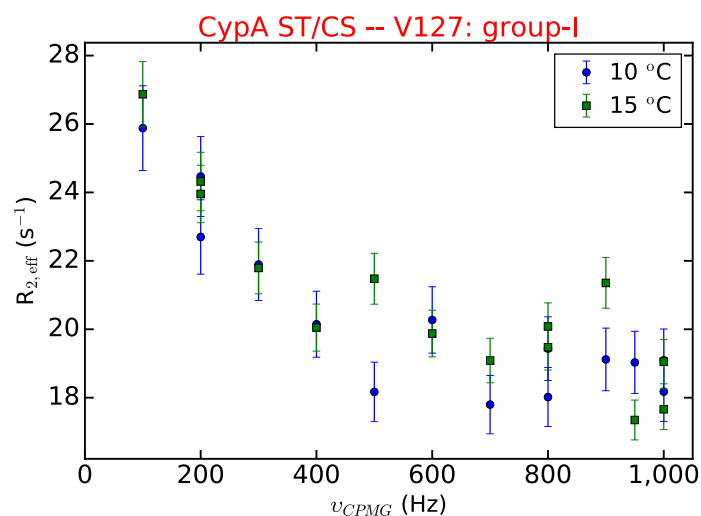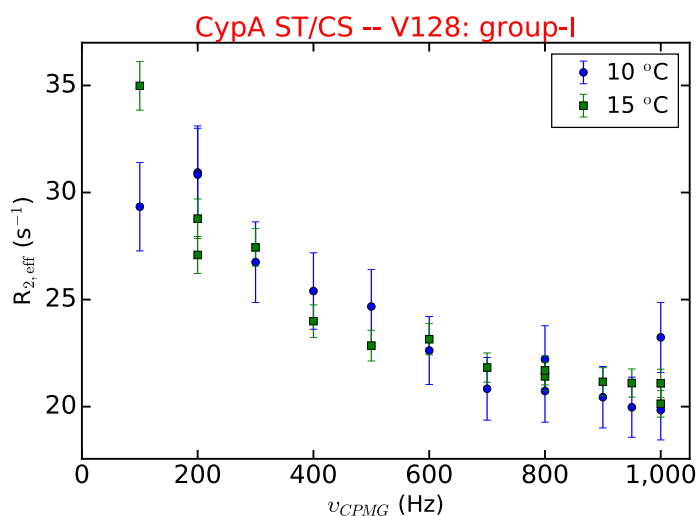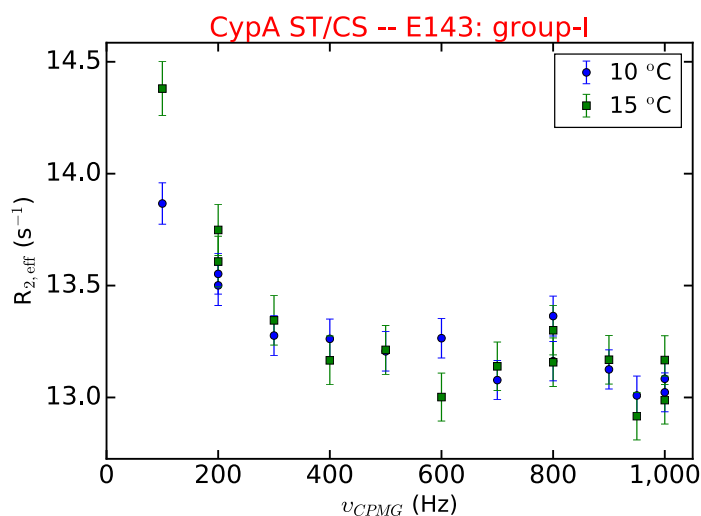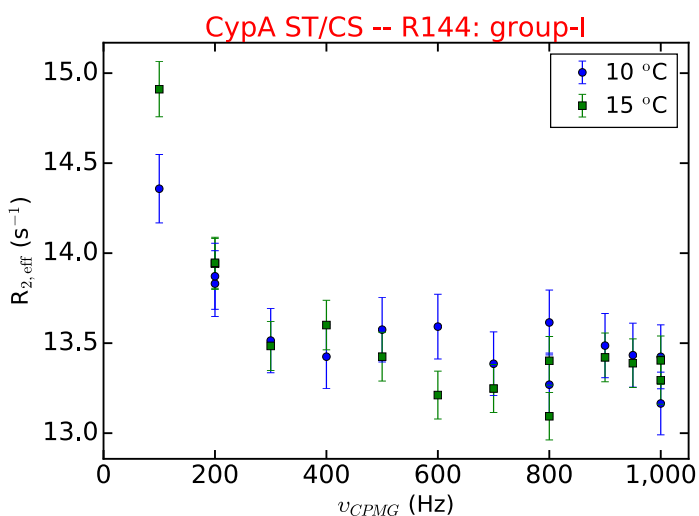

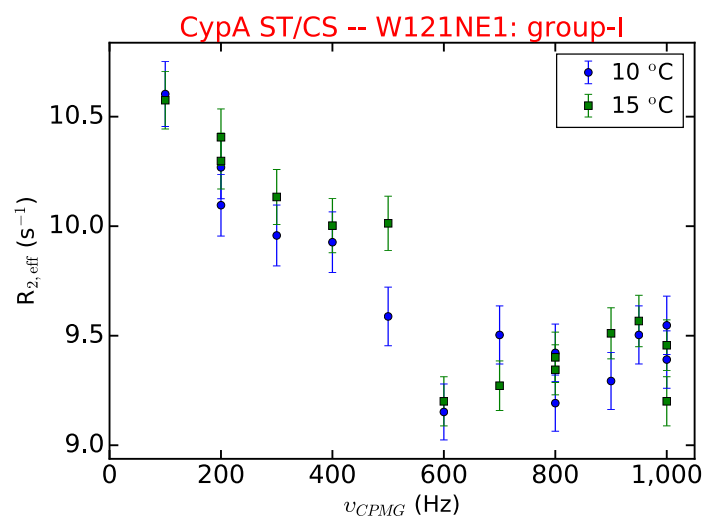

Supplement: Supplementary file 4 — Supplementary Data 1 [file 41467_2018_3562_MOESM4_ESM.pdf]

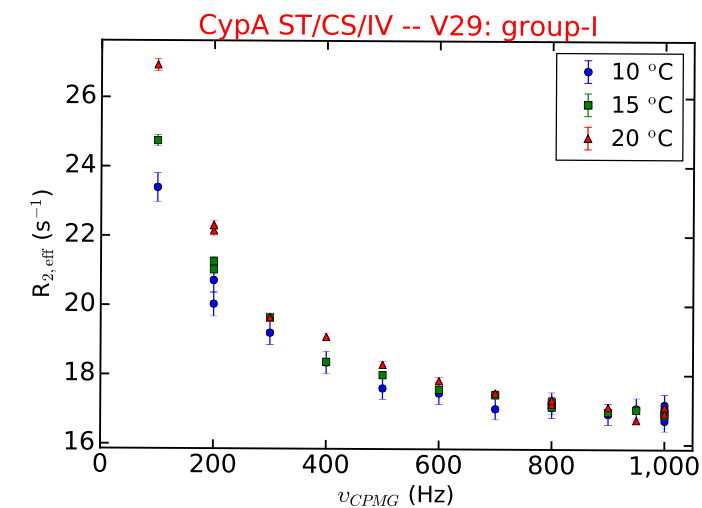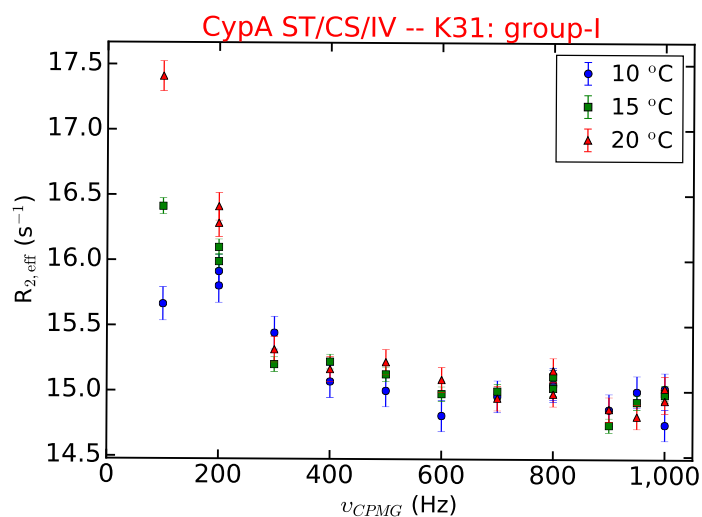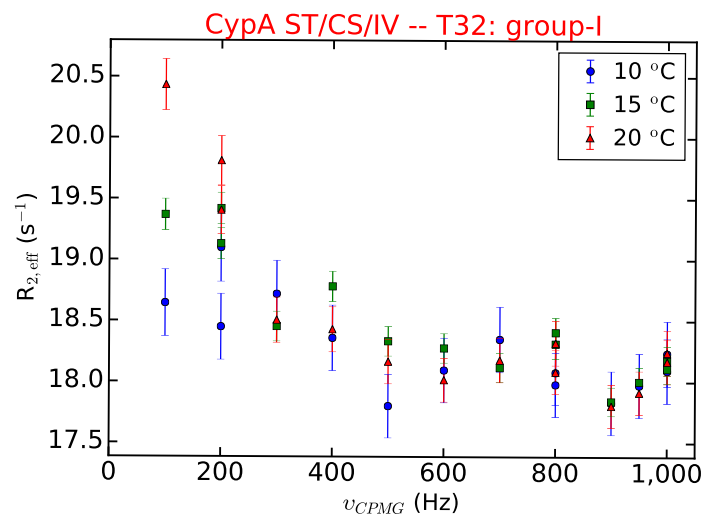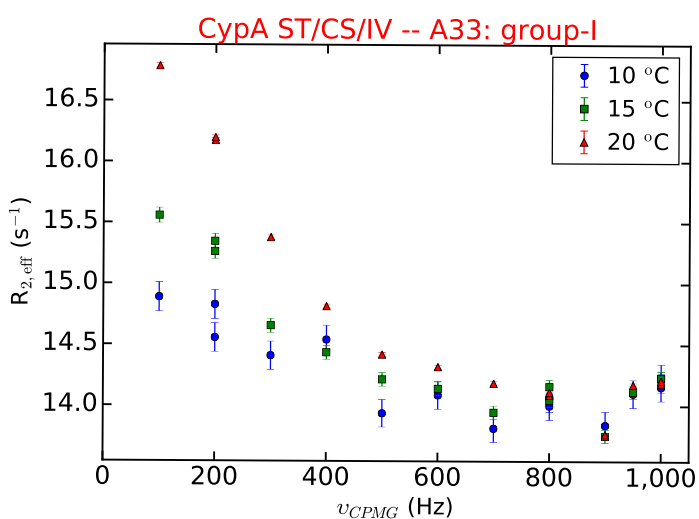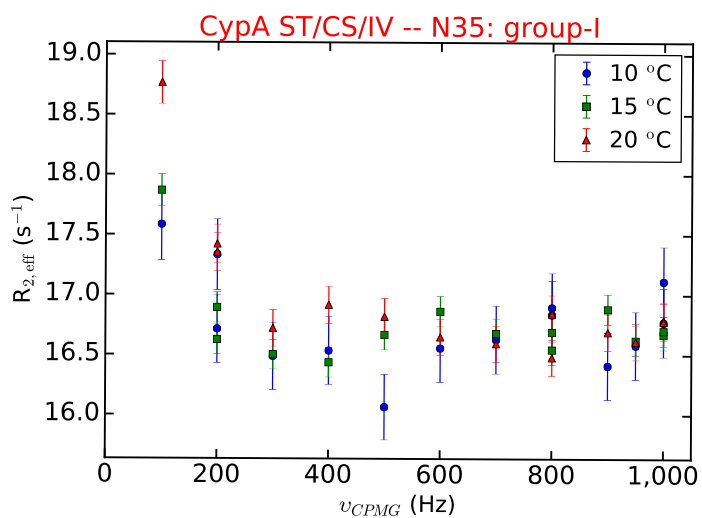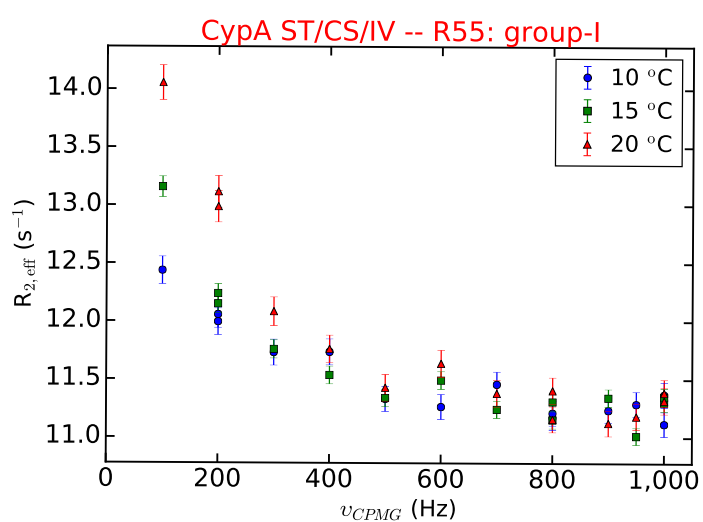

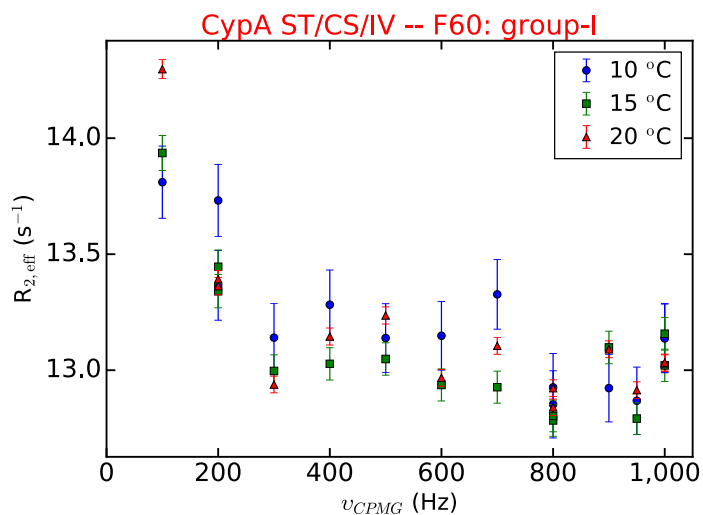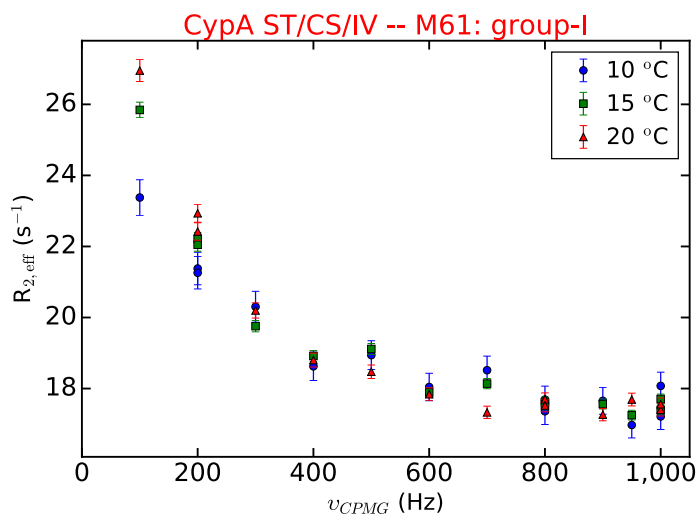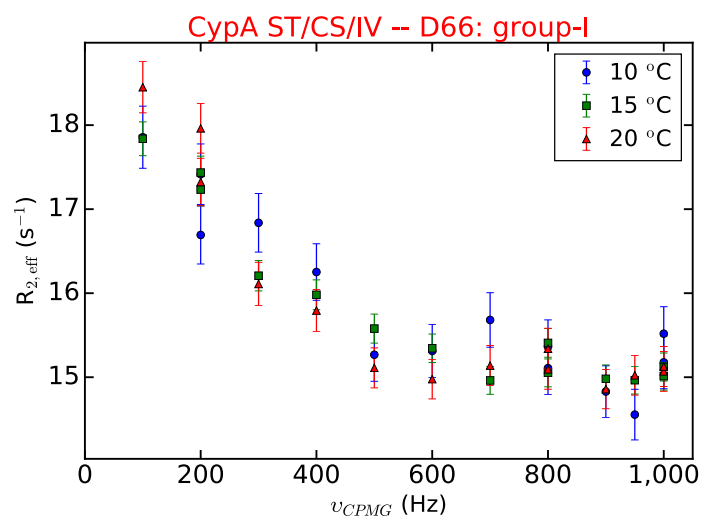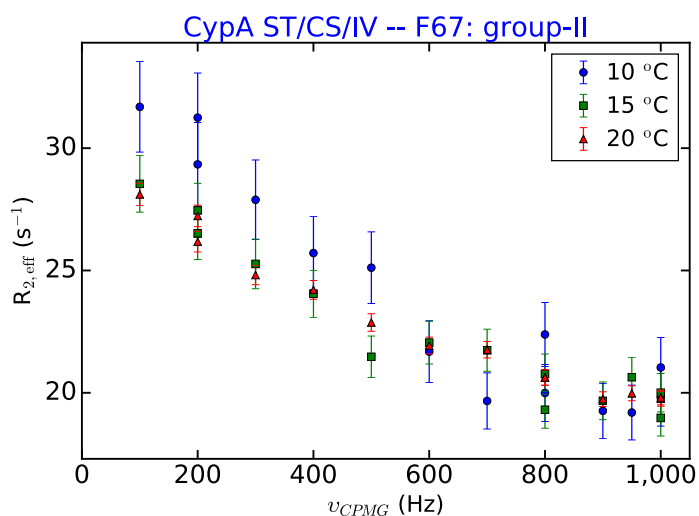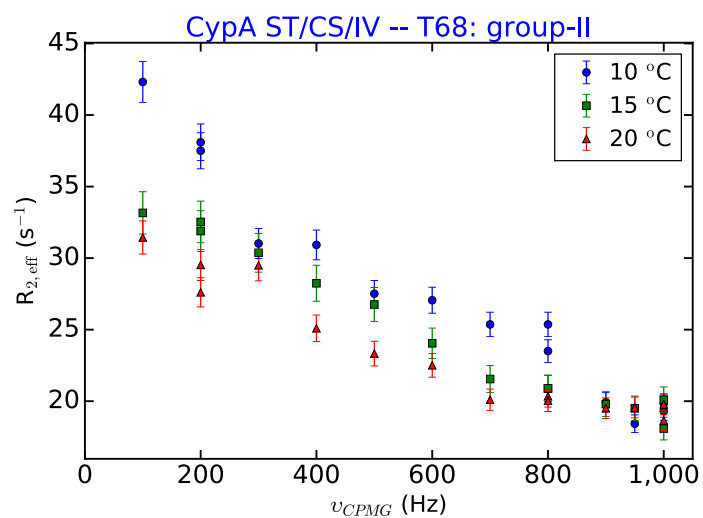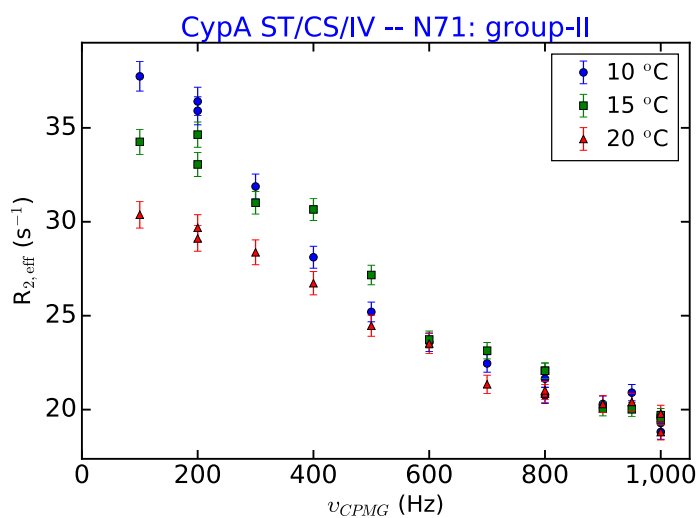

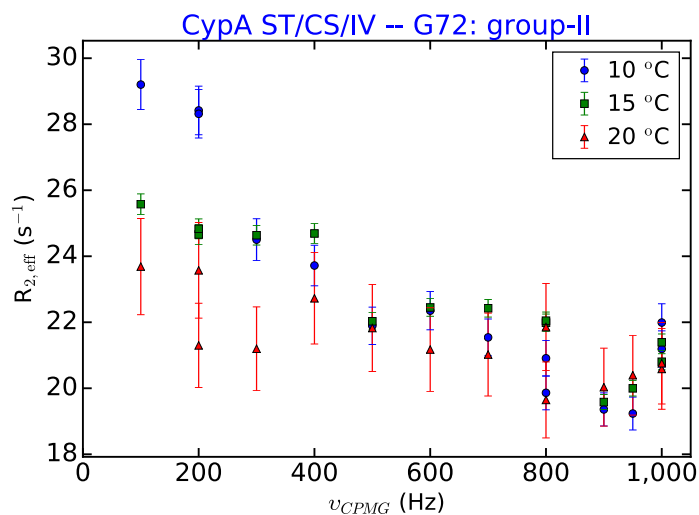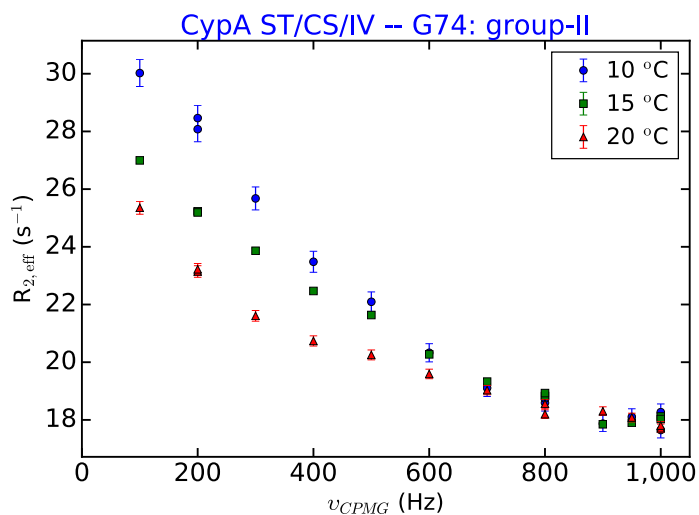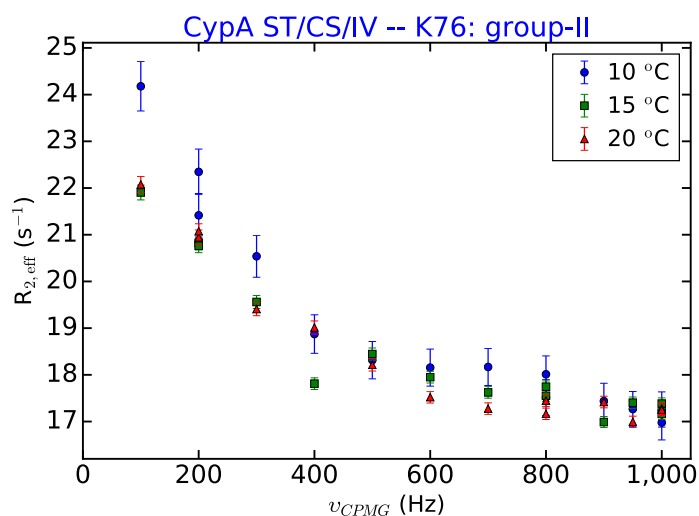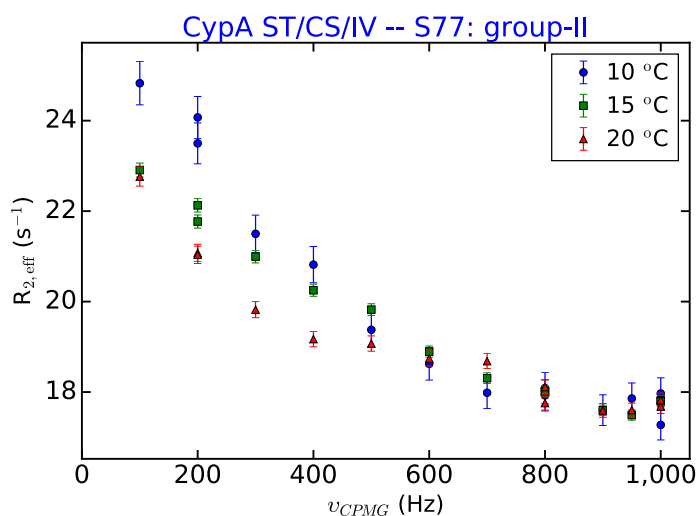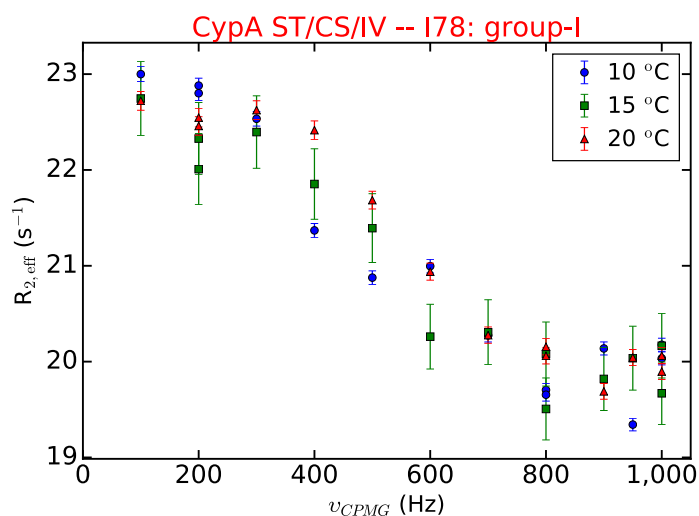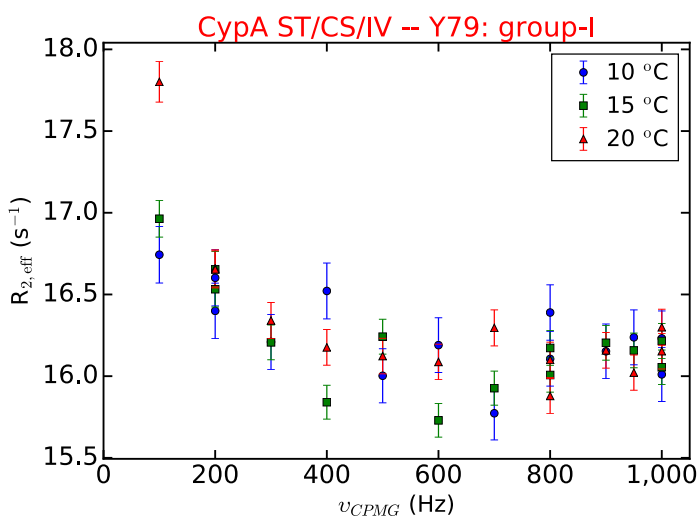

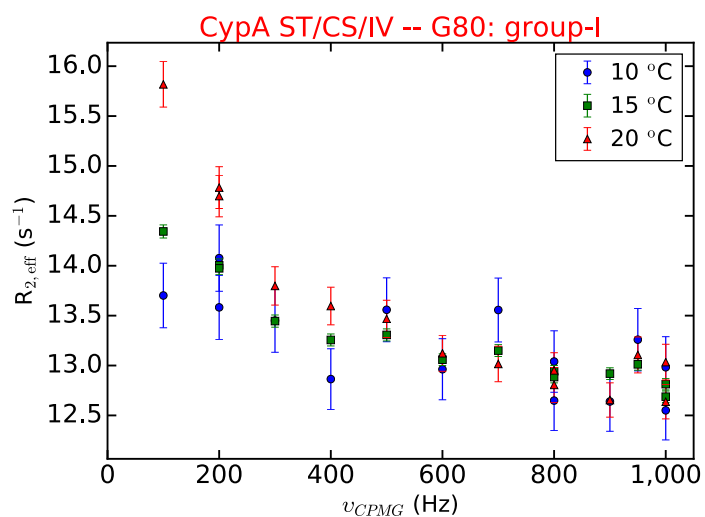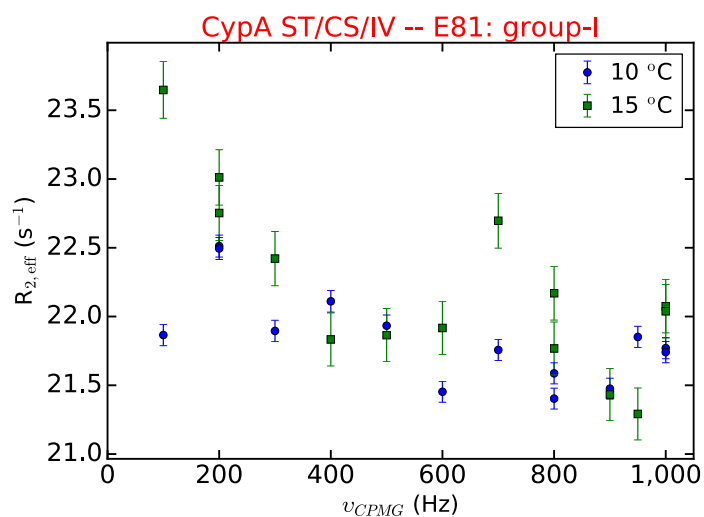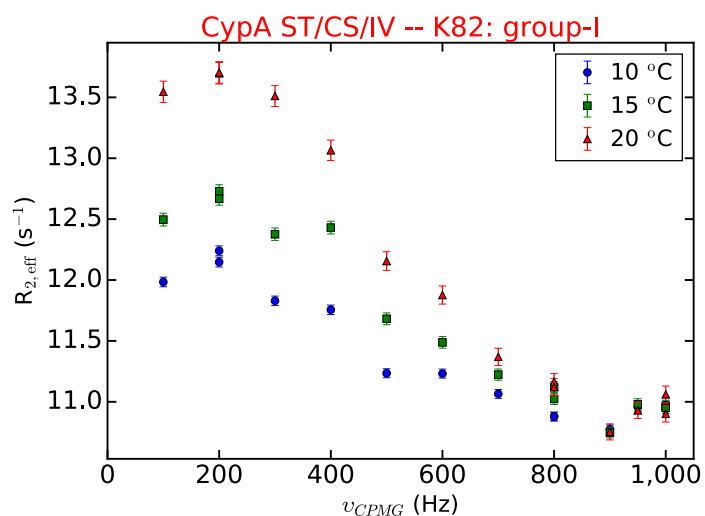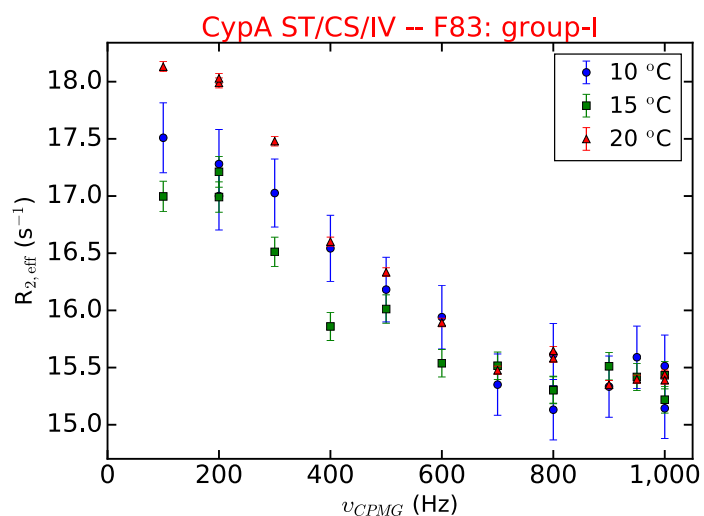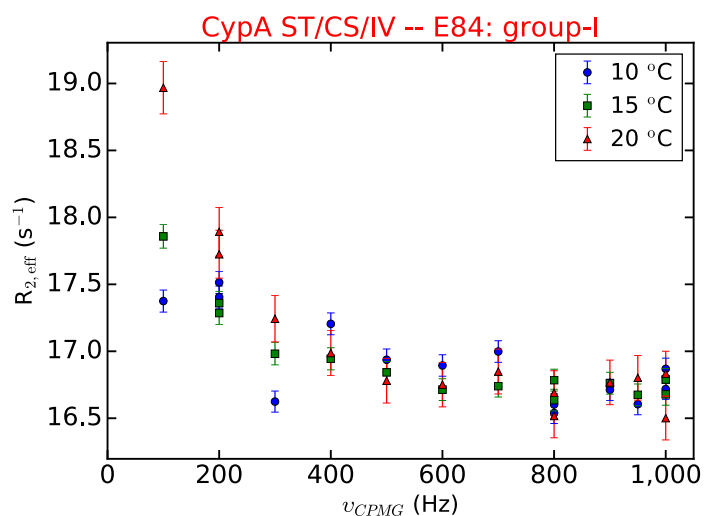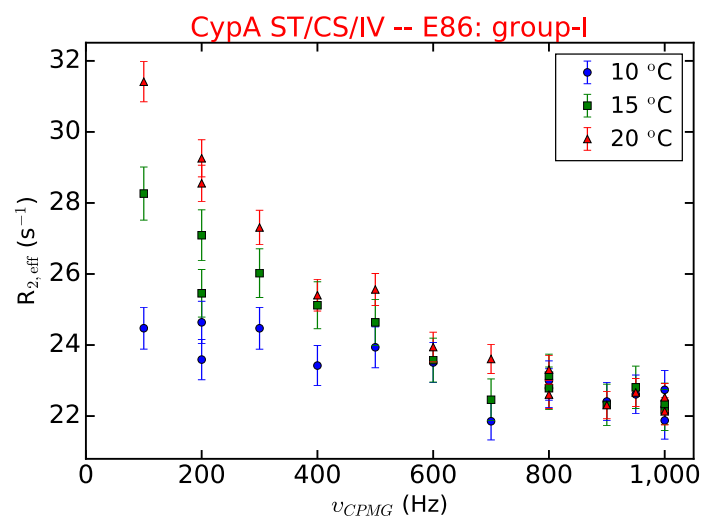

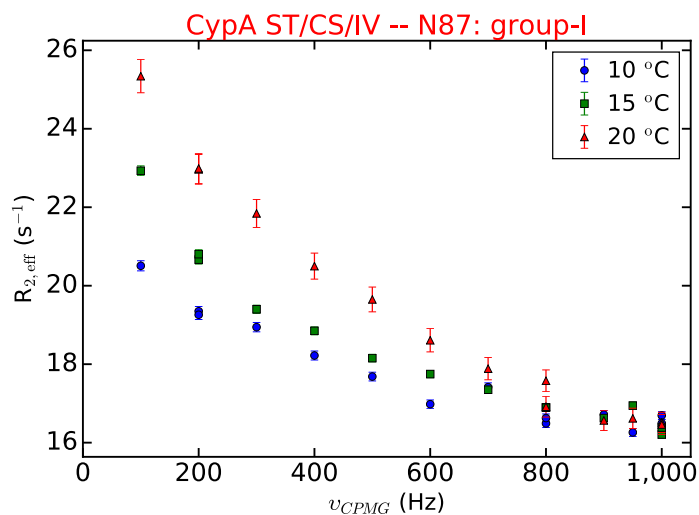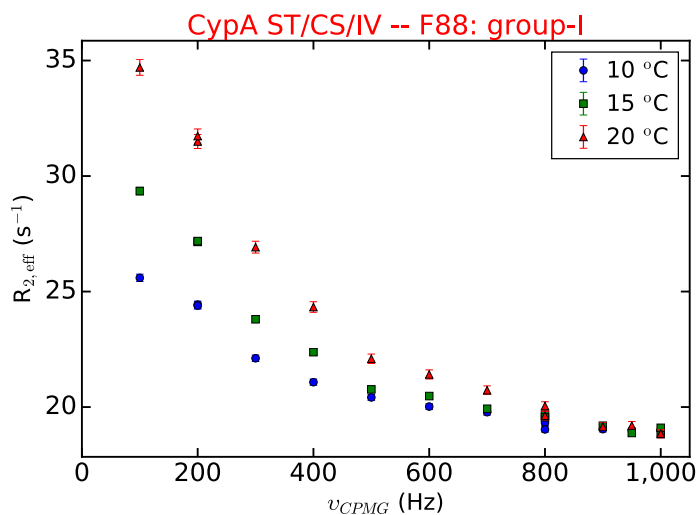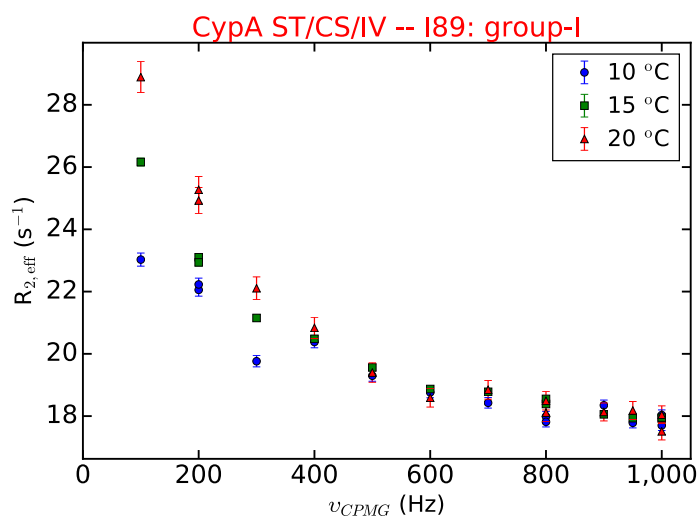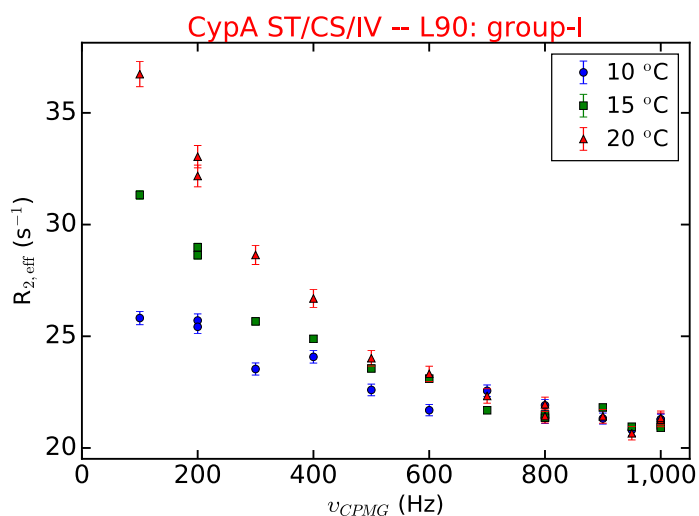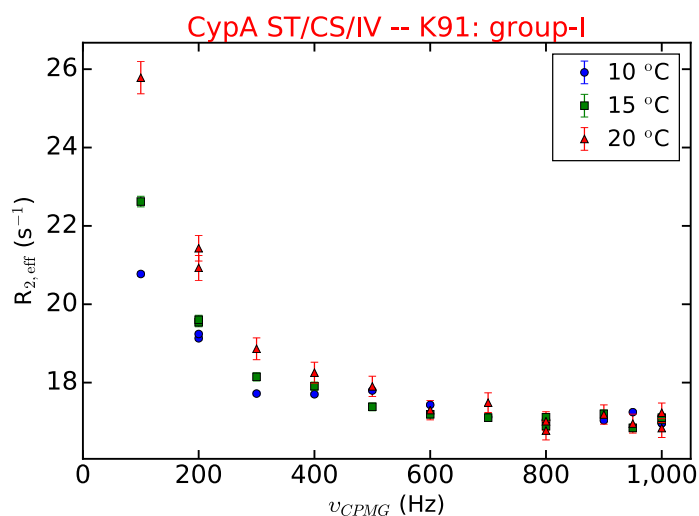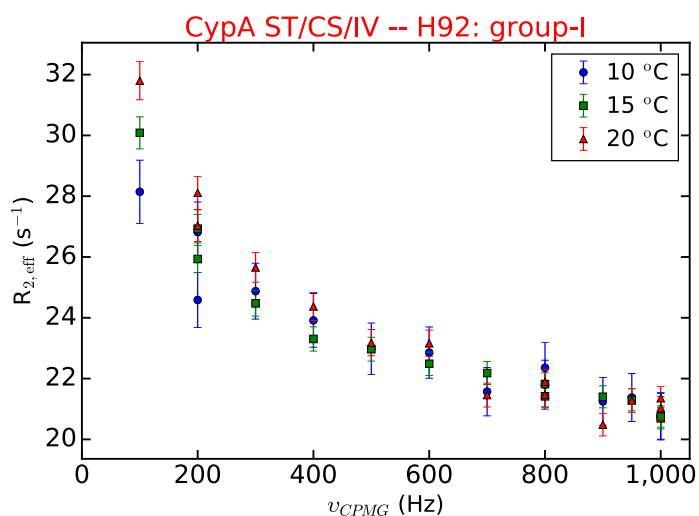

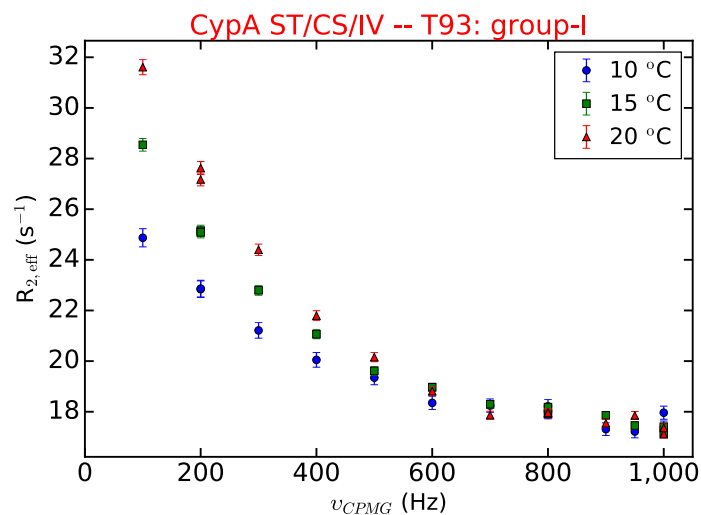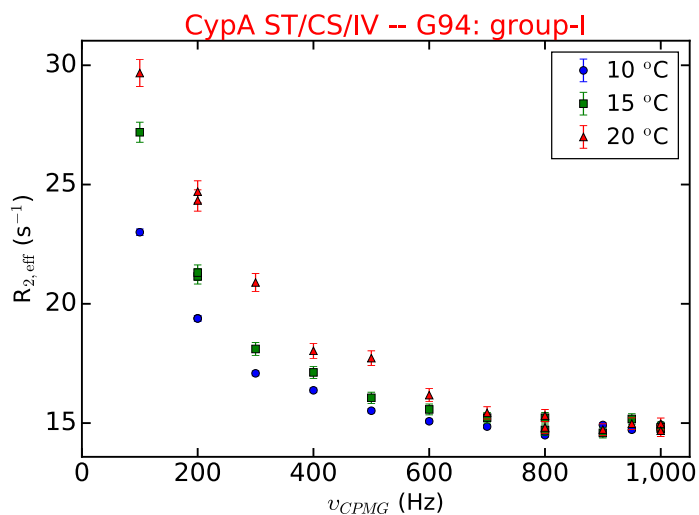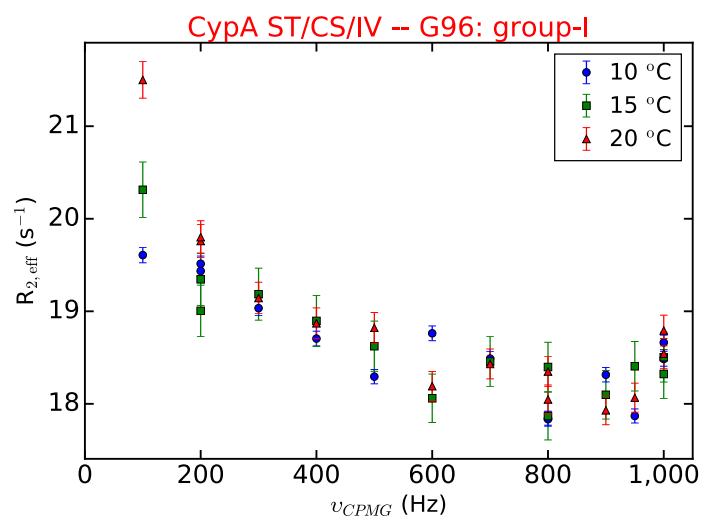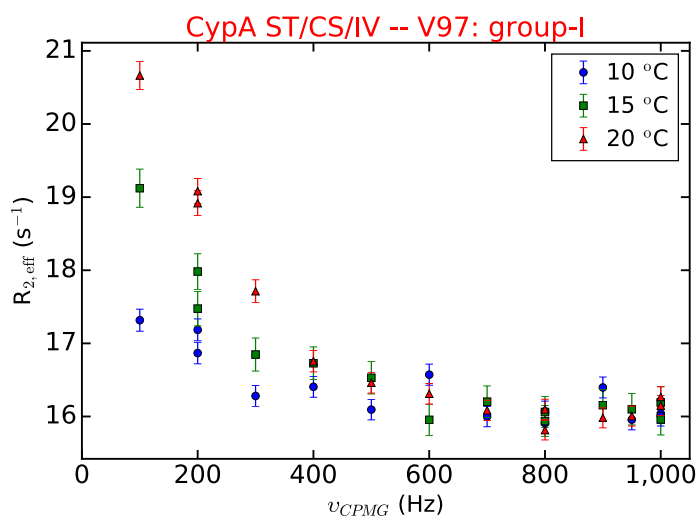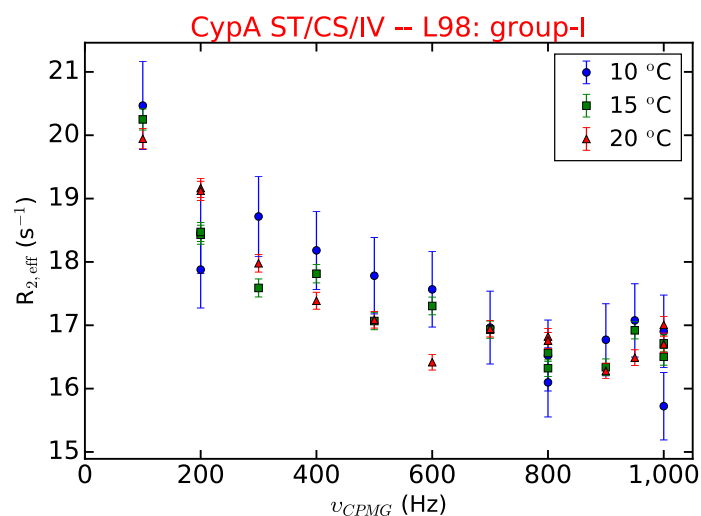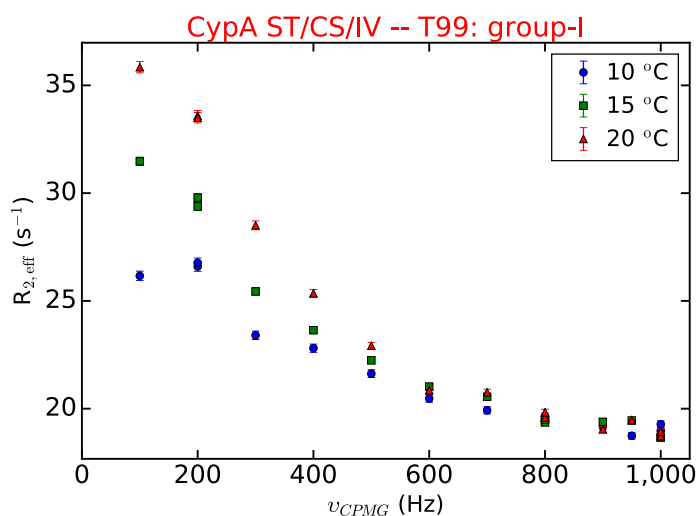

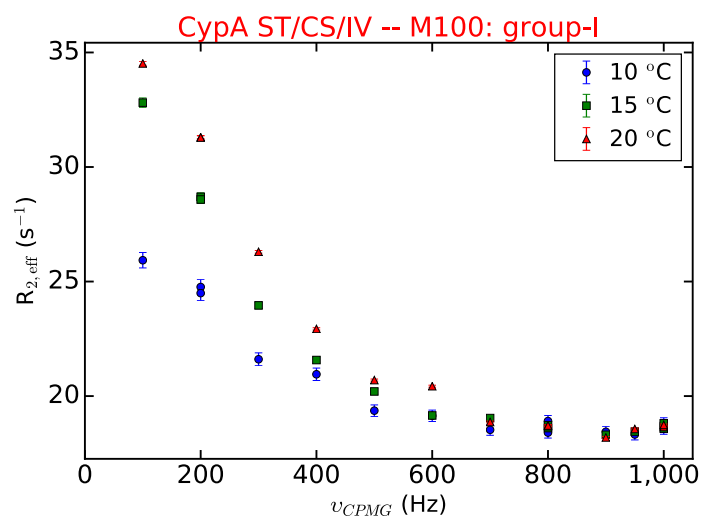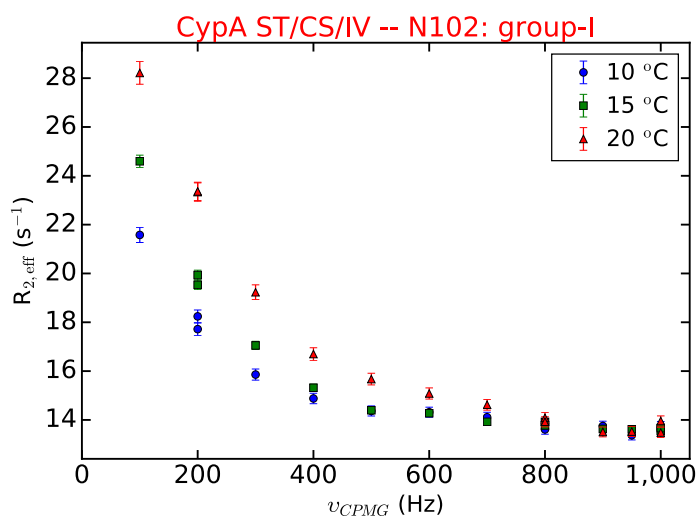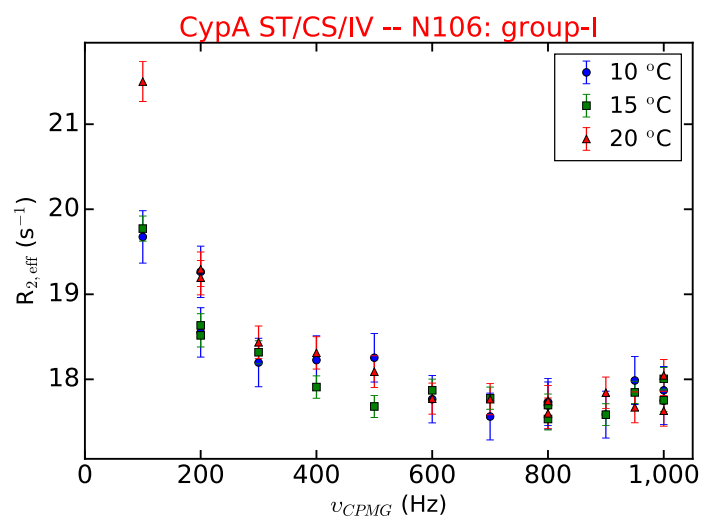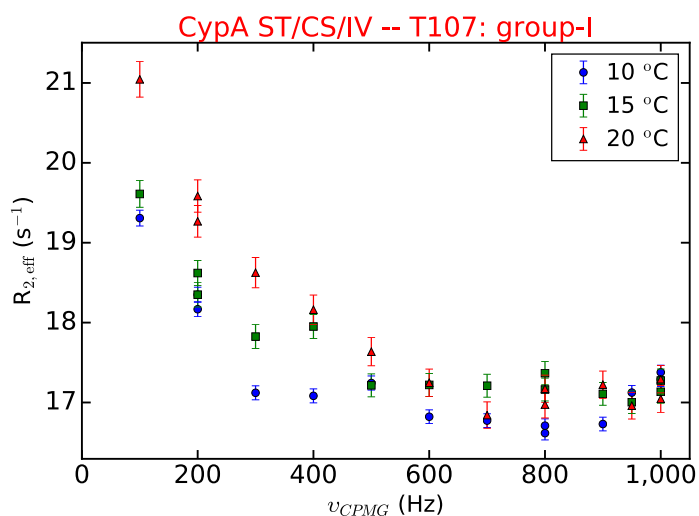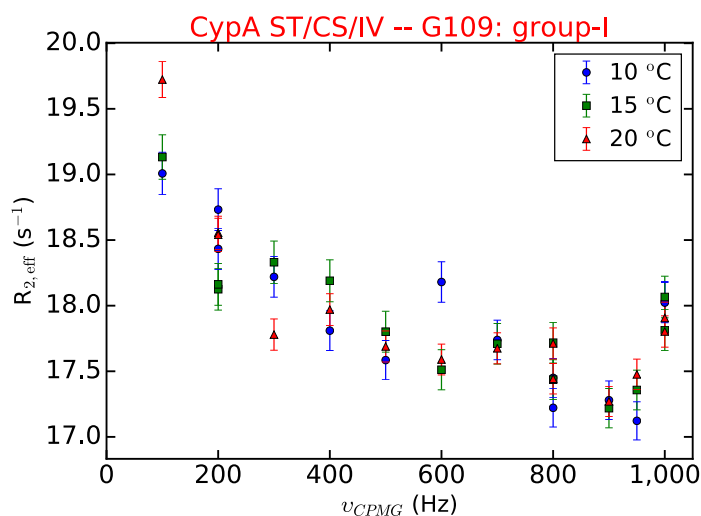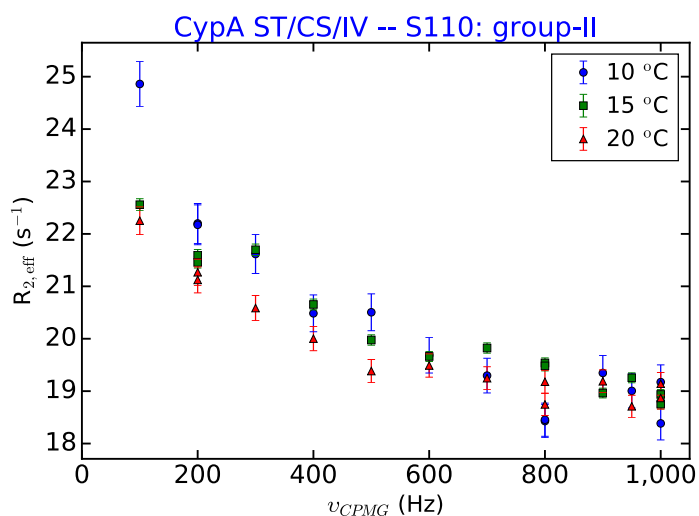

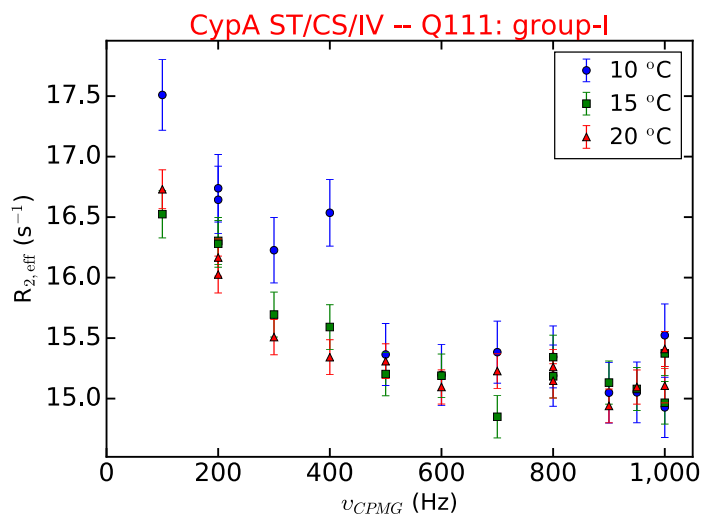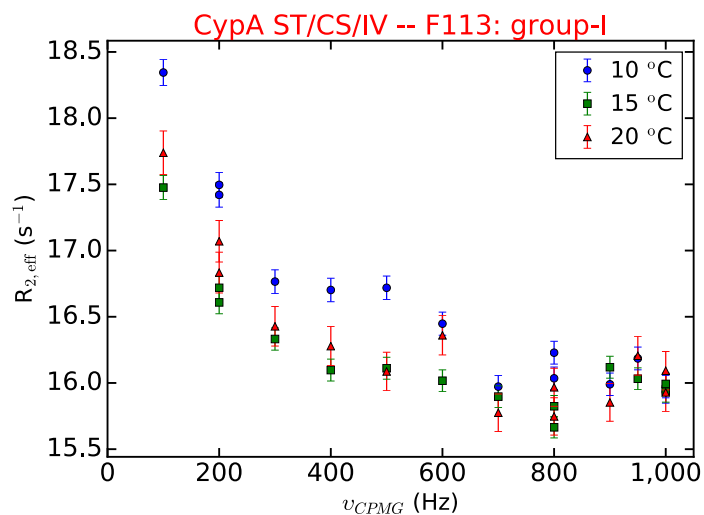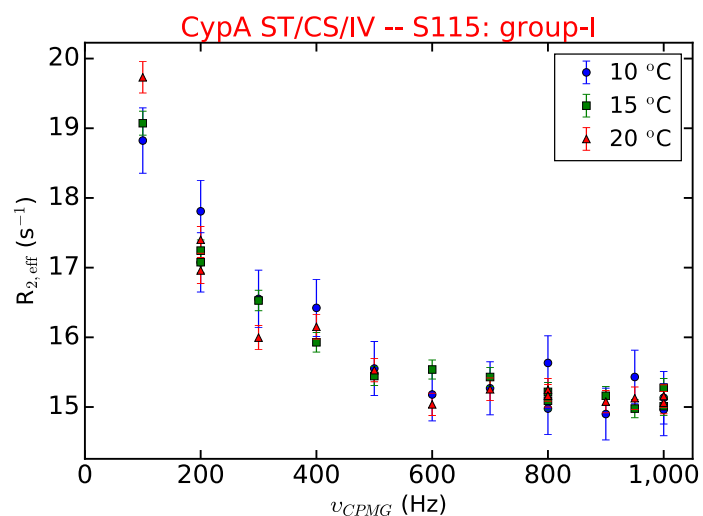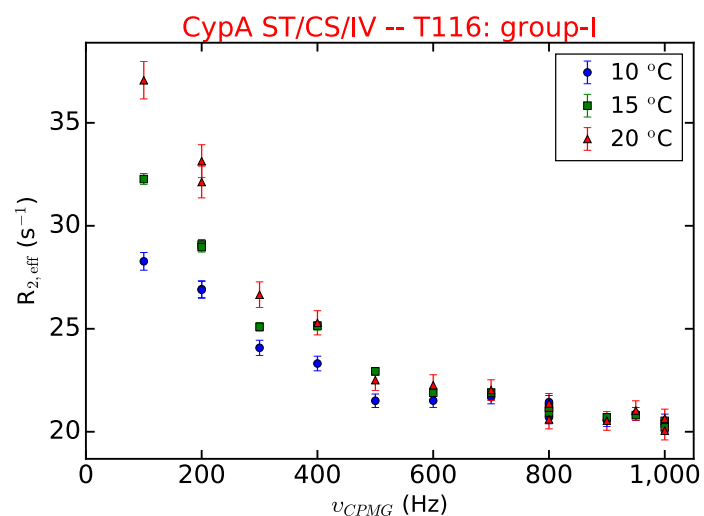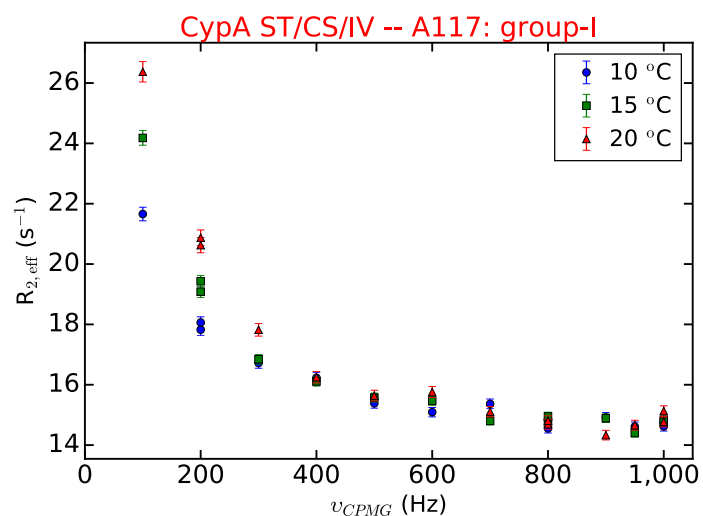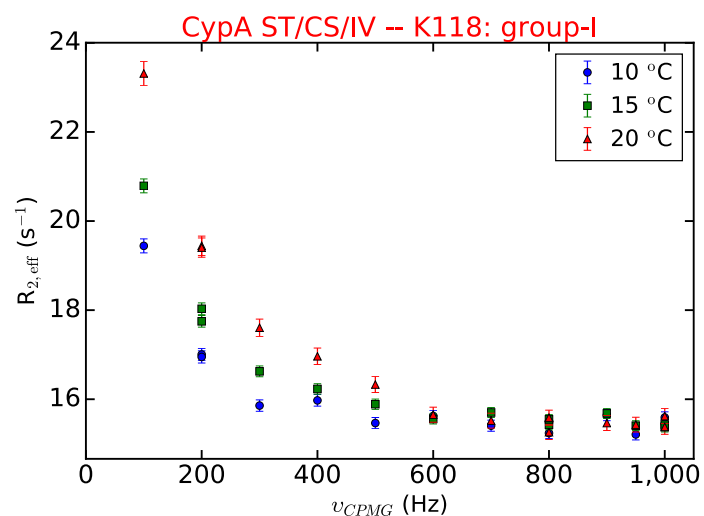

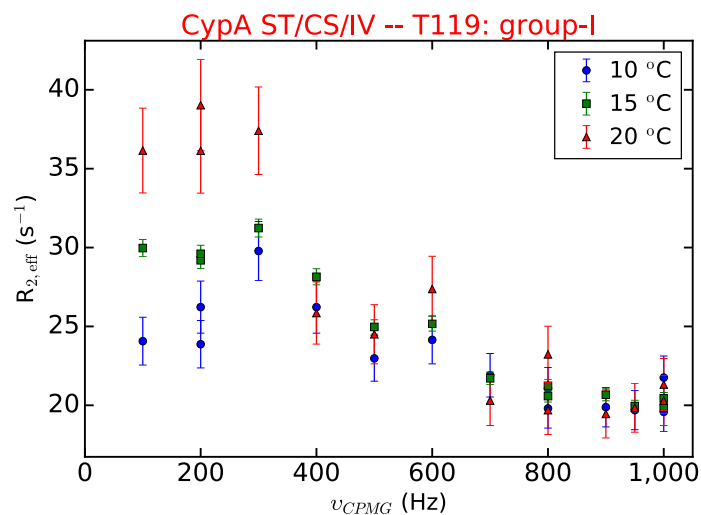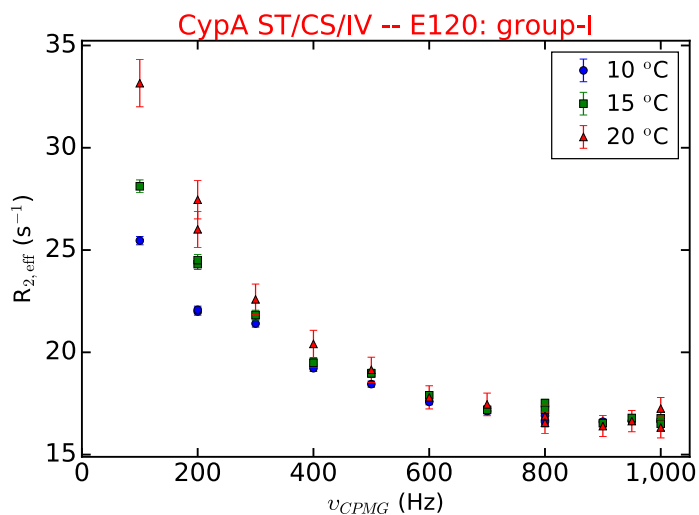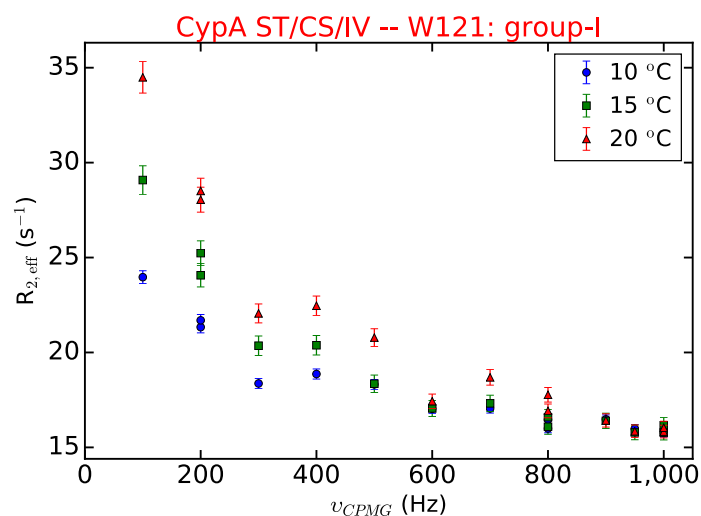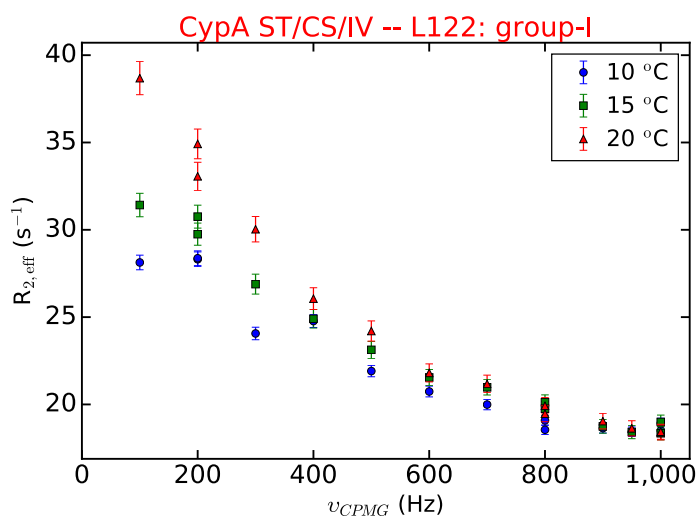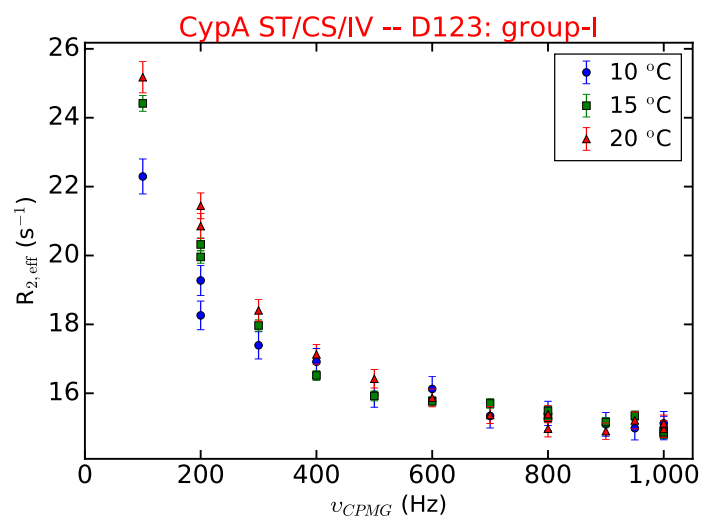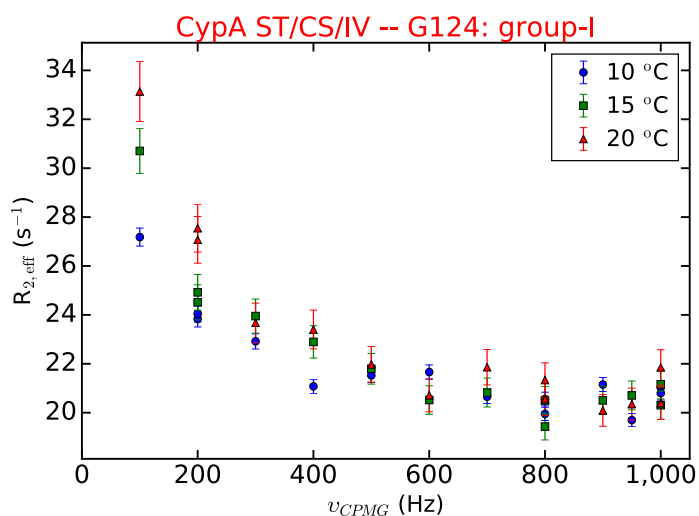

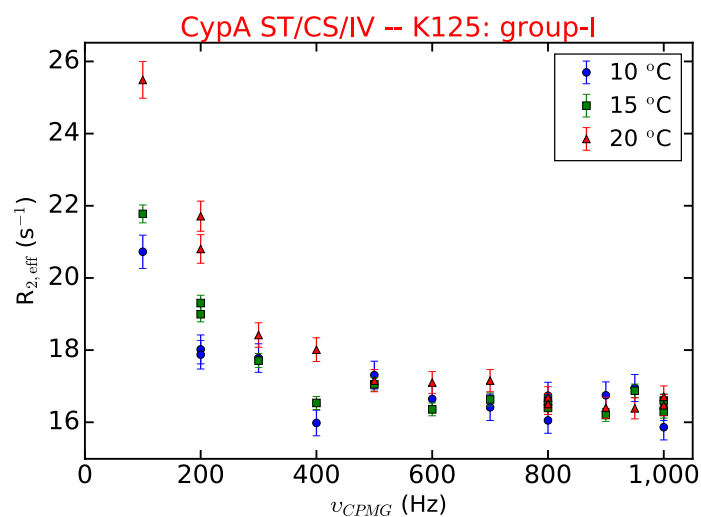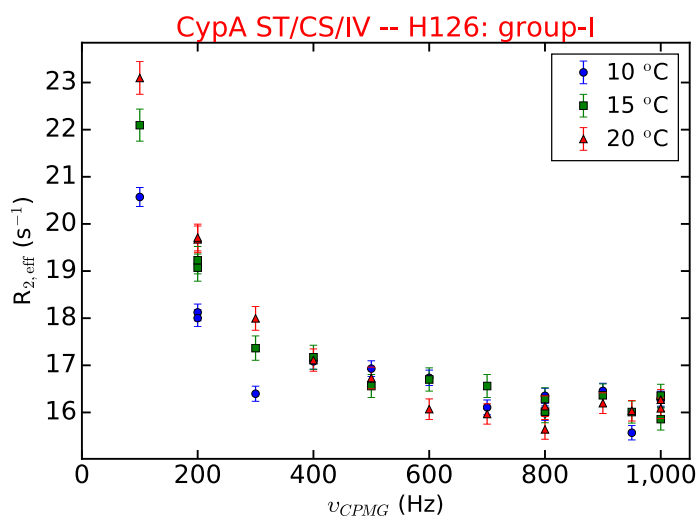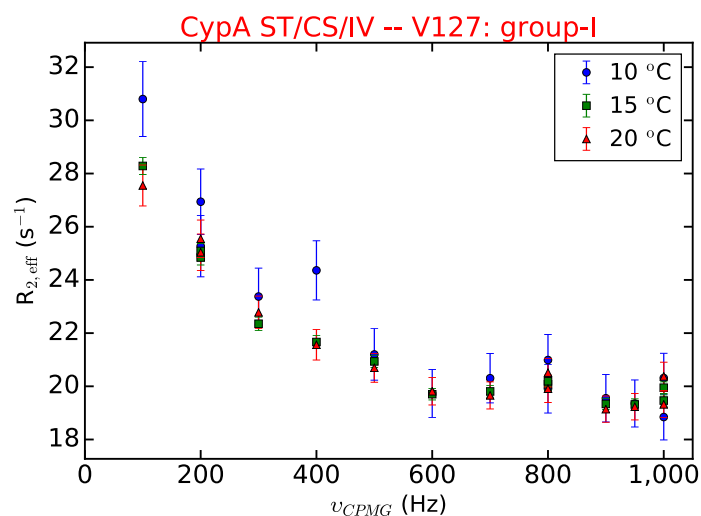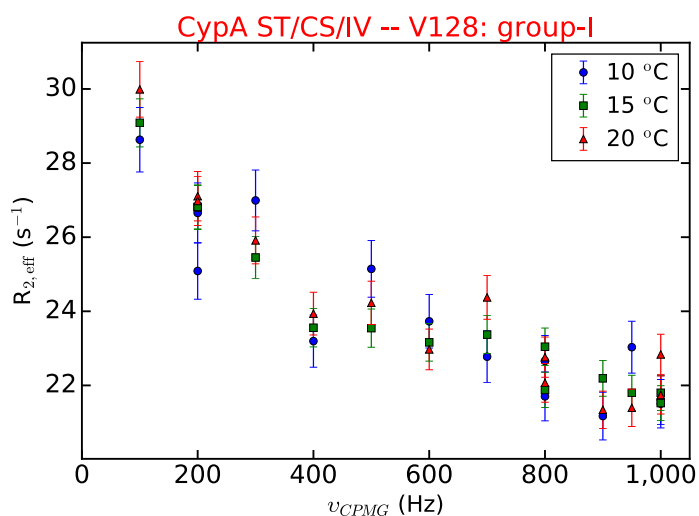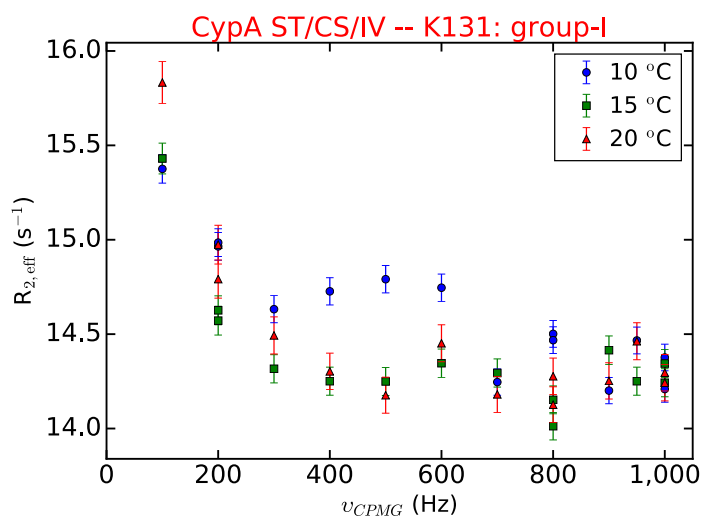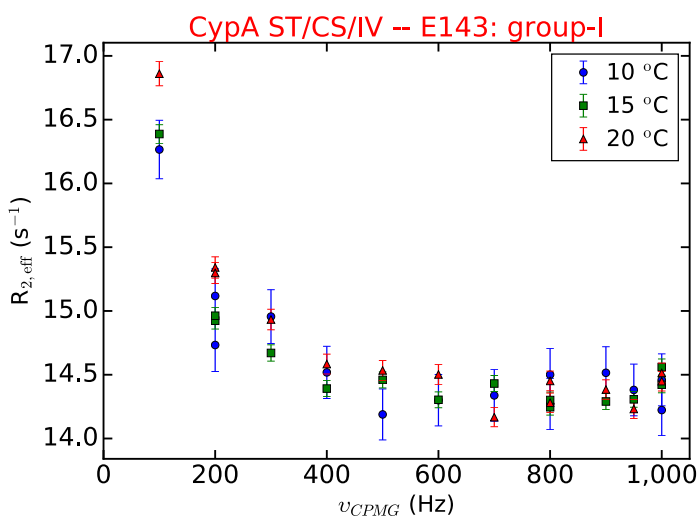

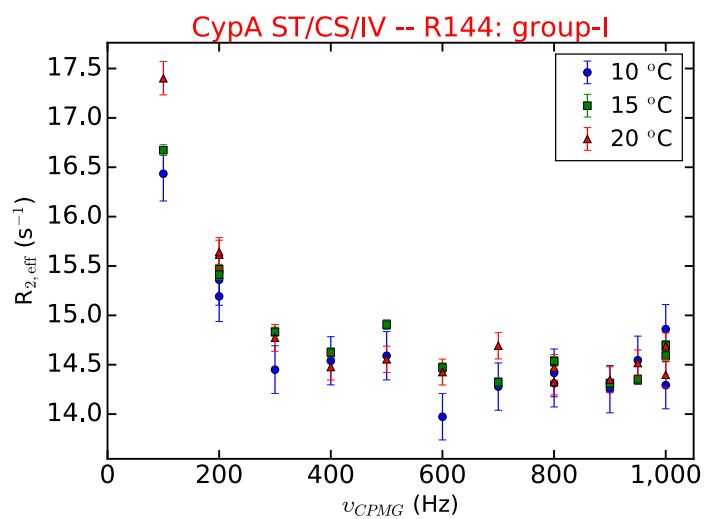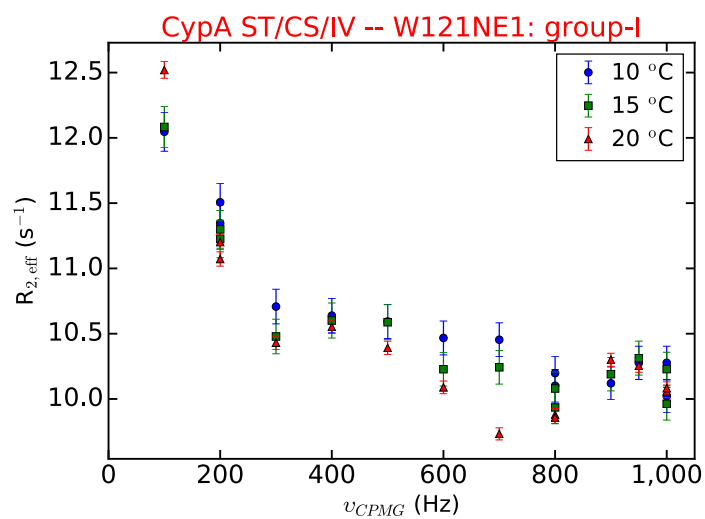

Supplement: Supplementary file 5 — Supplementary Data 2 [file 41467_2018_3562_MOESM5_ESM.pdf]

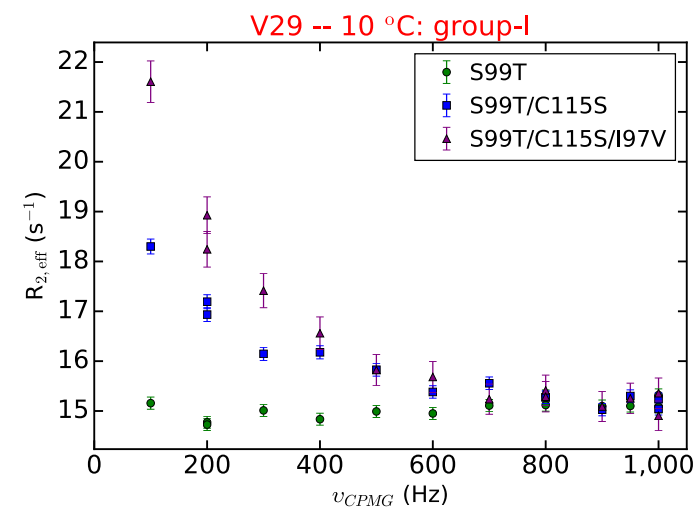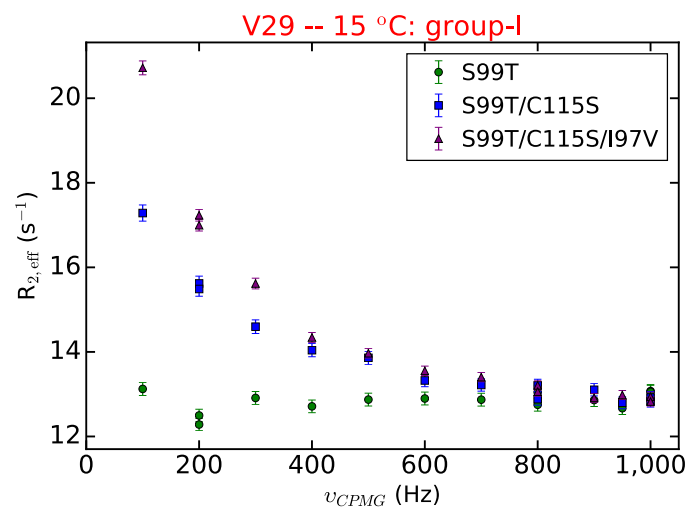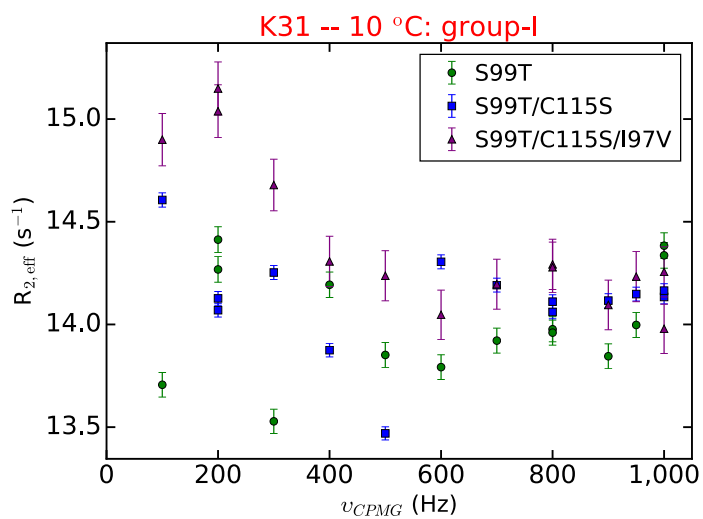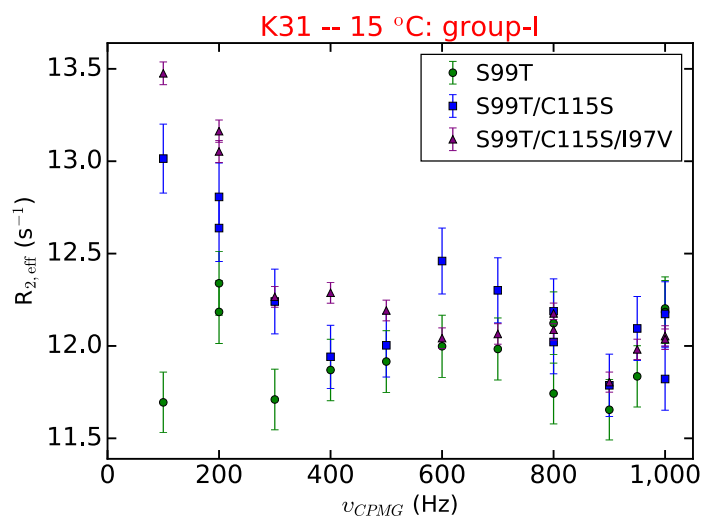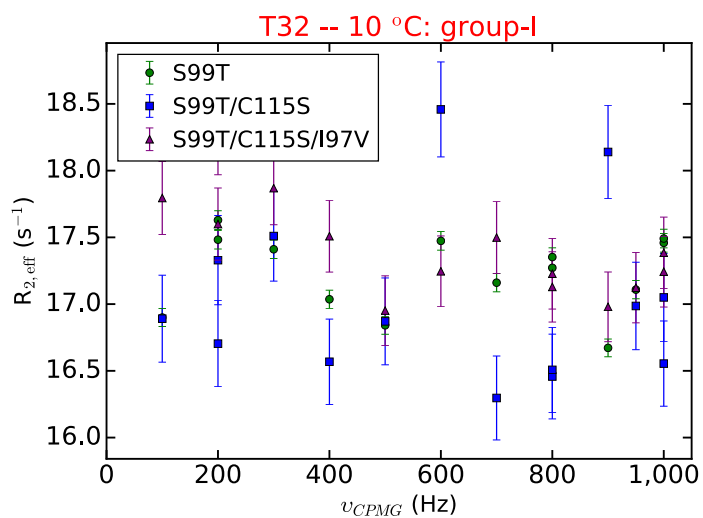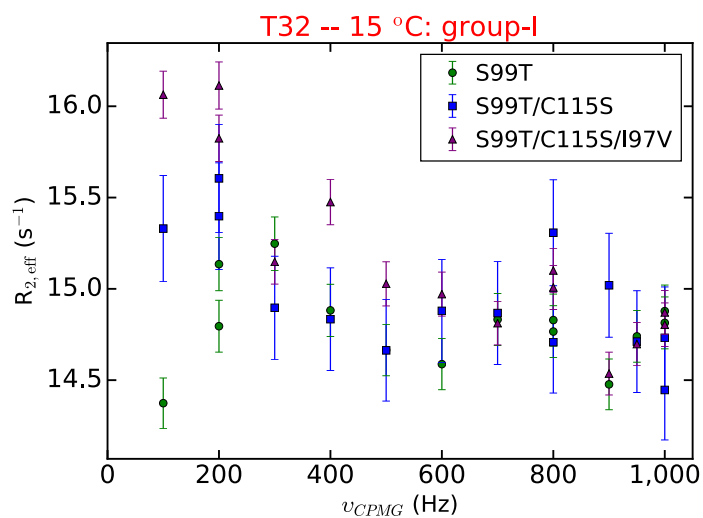

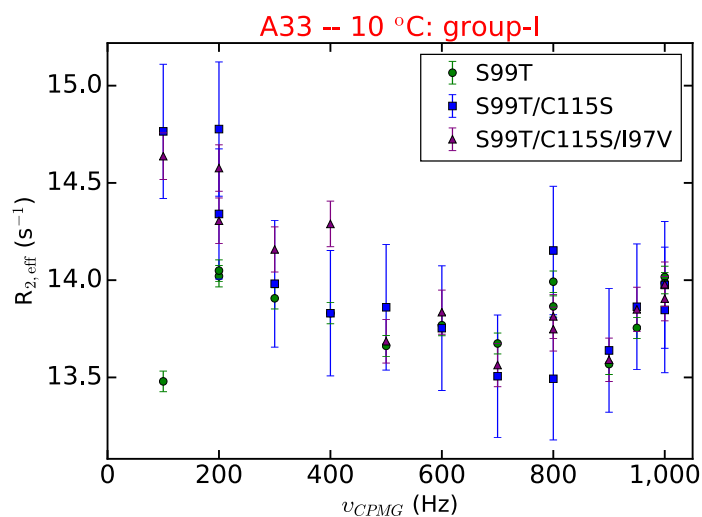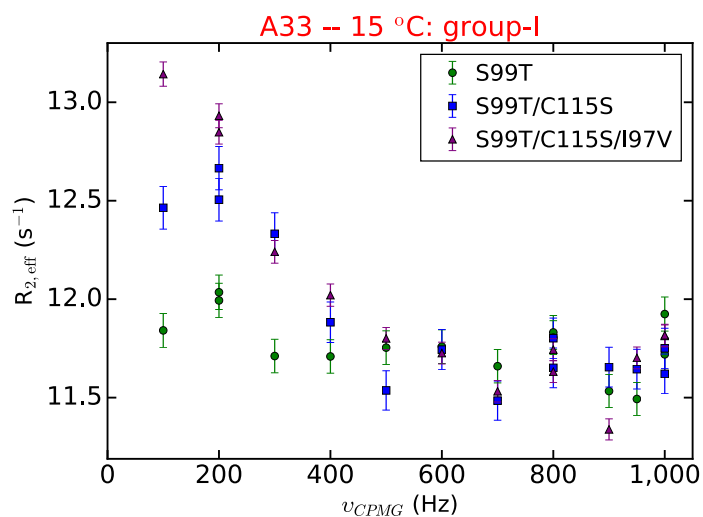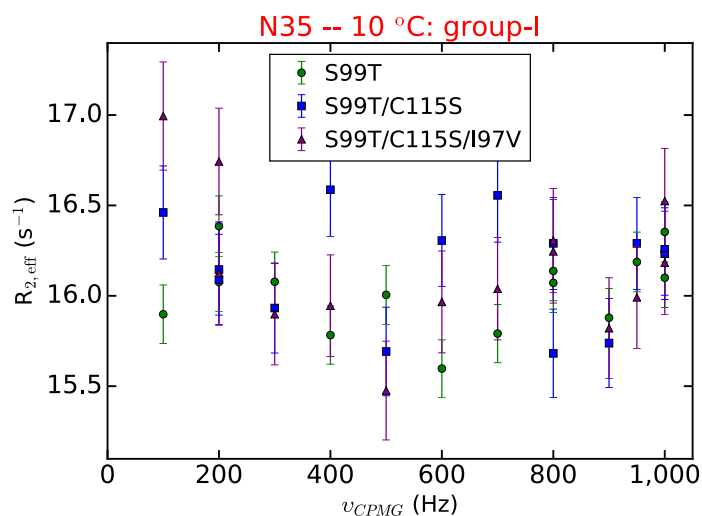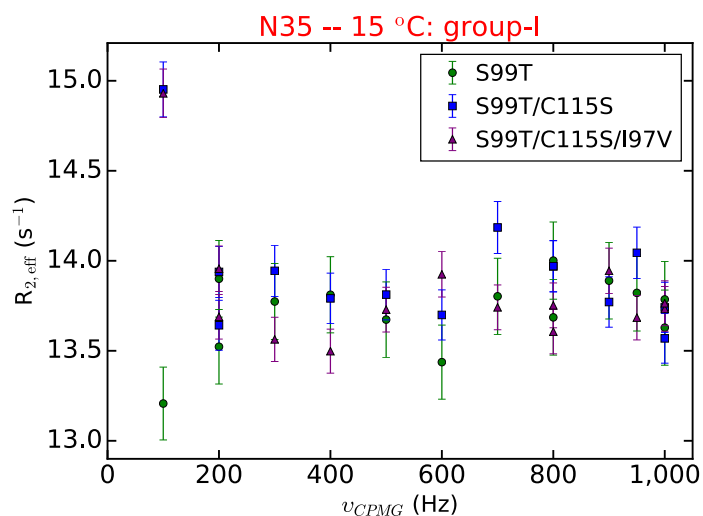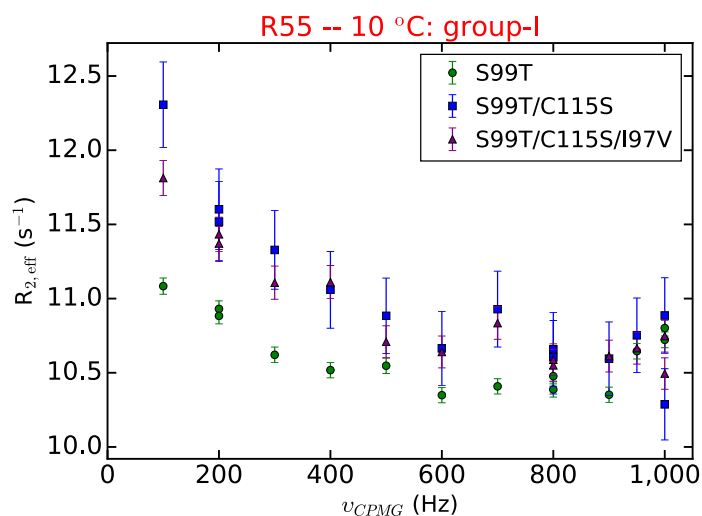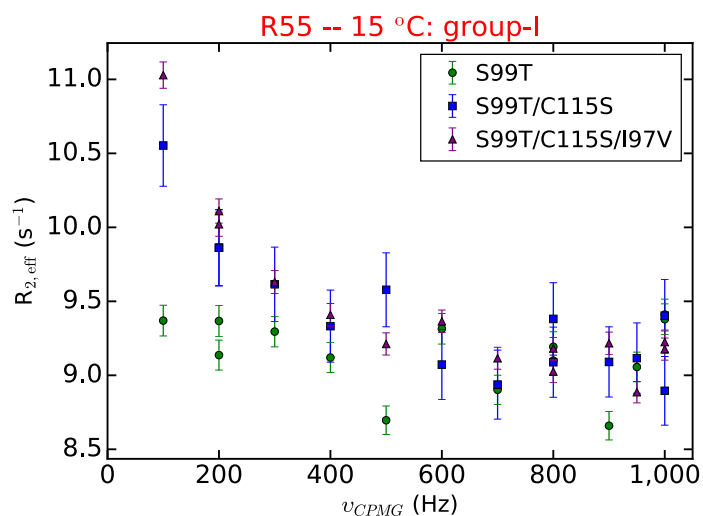

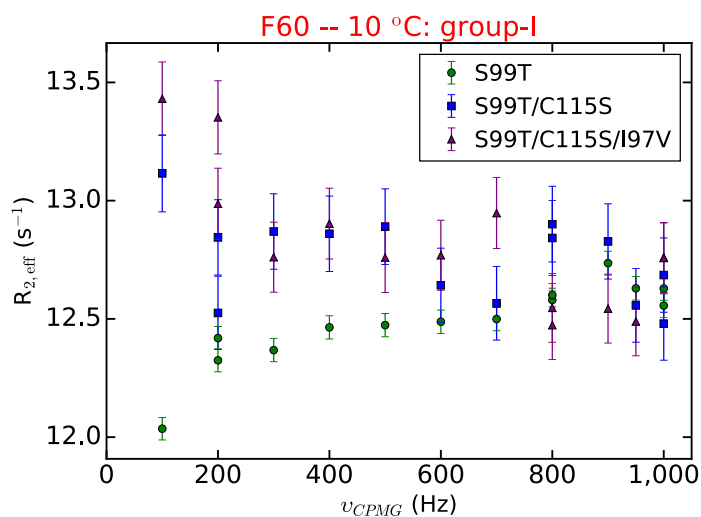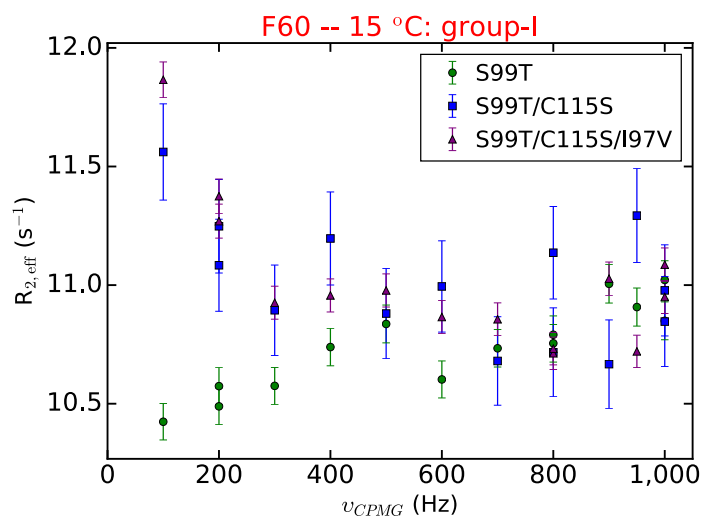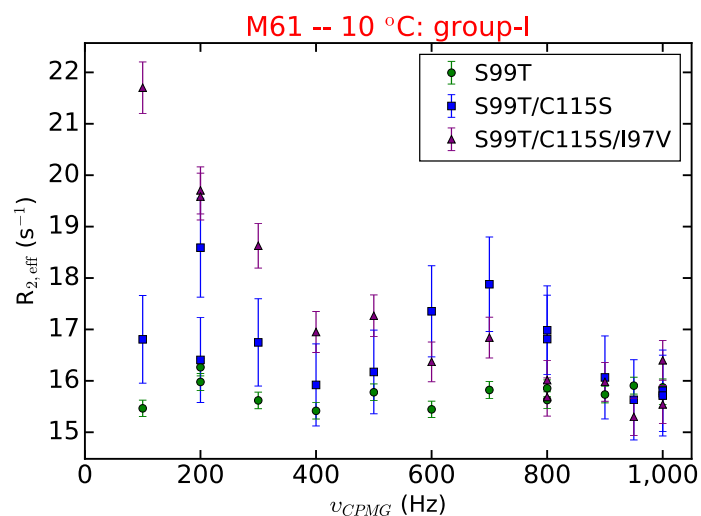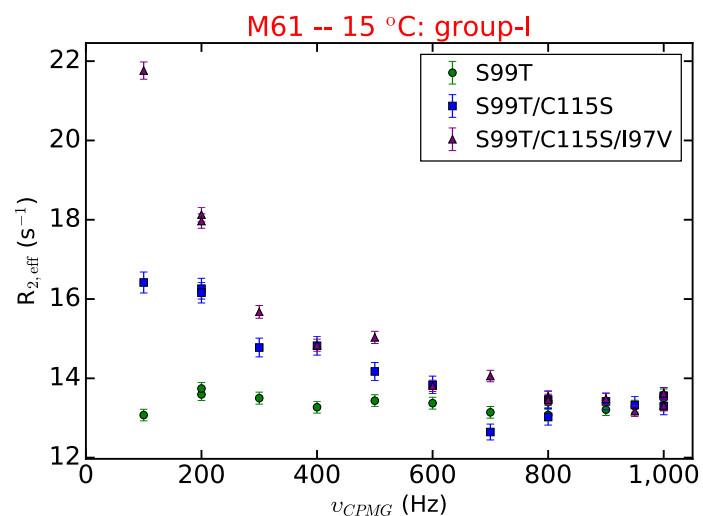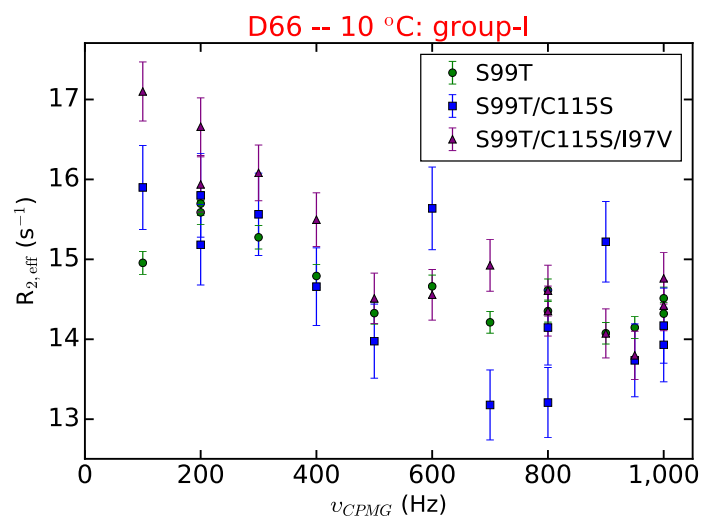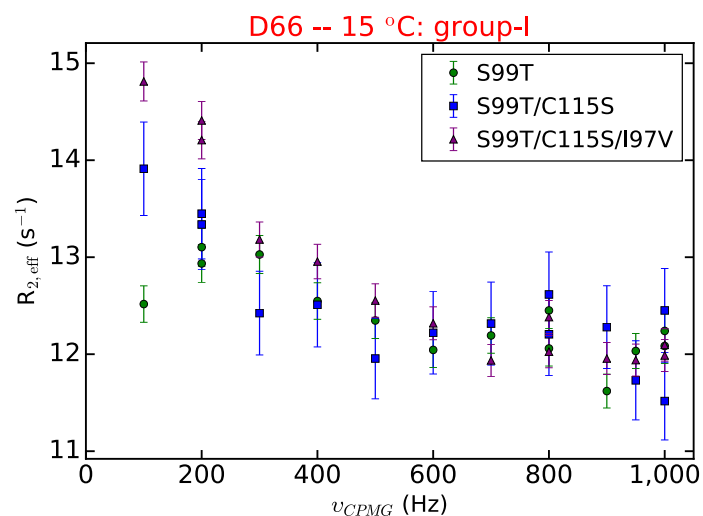

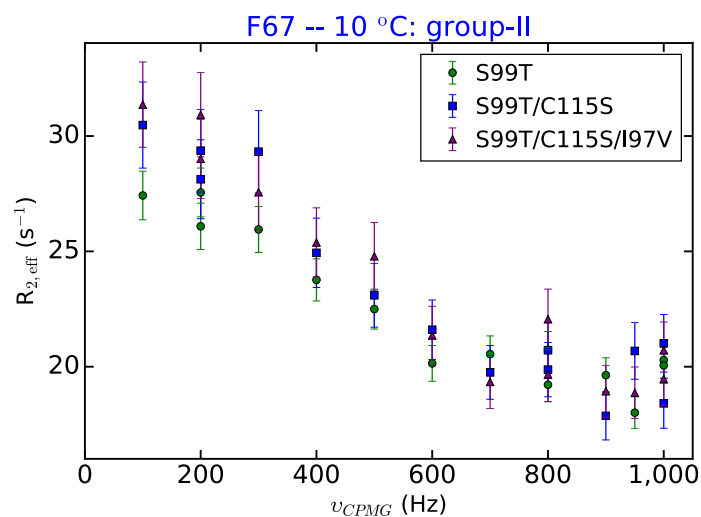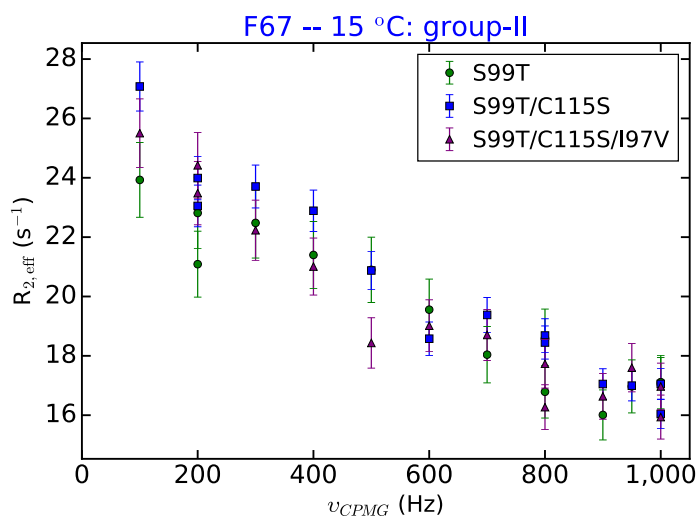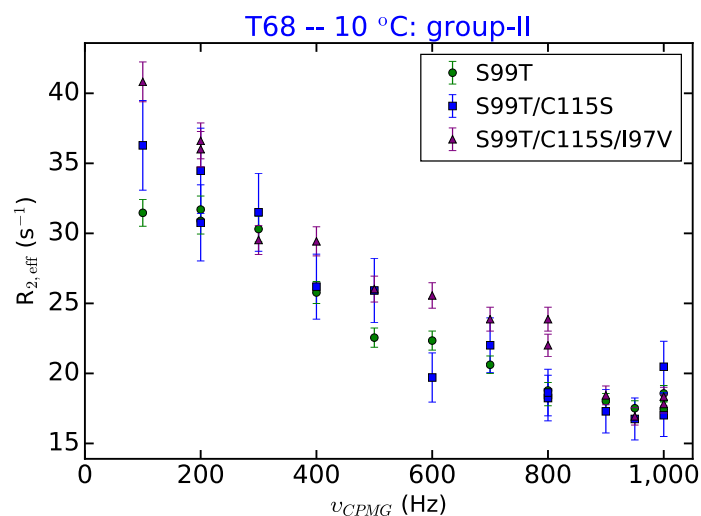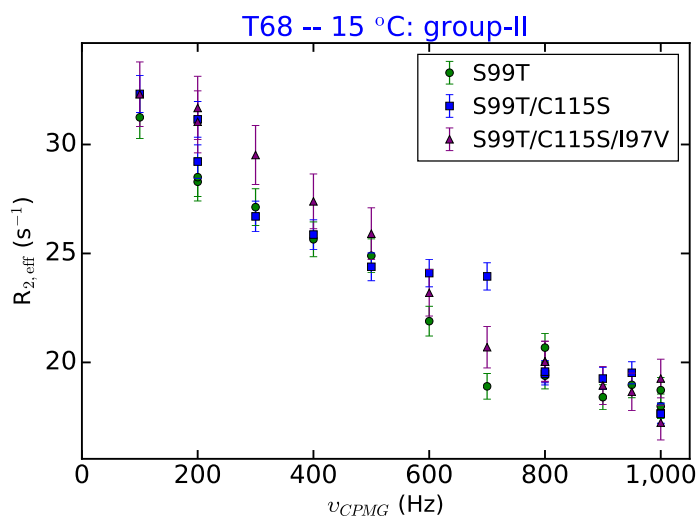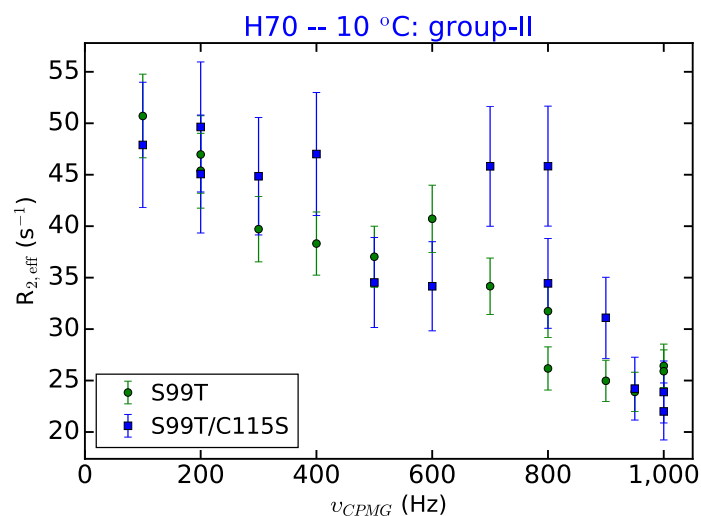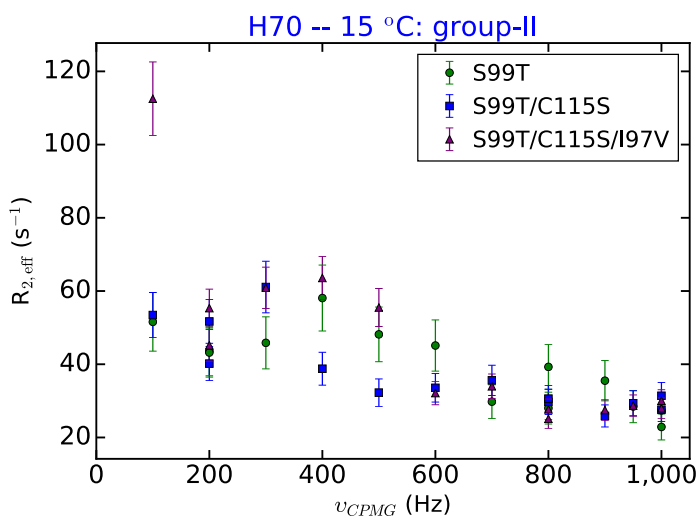

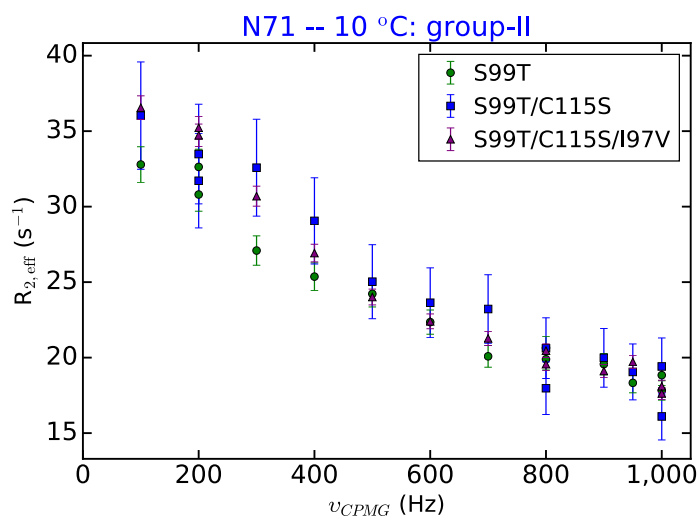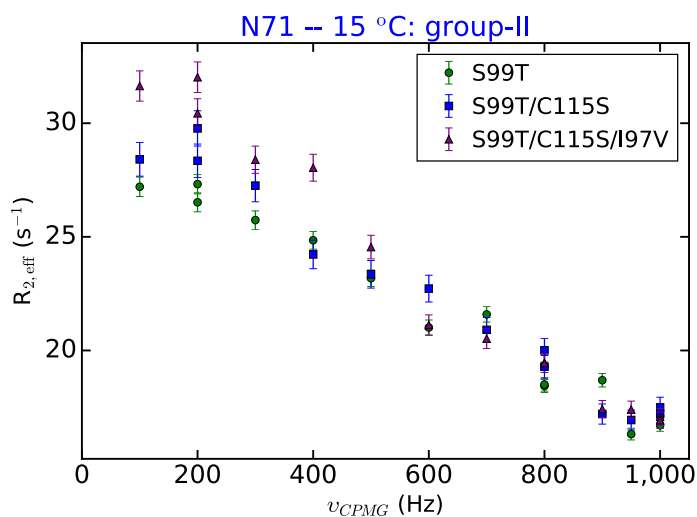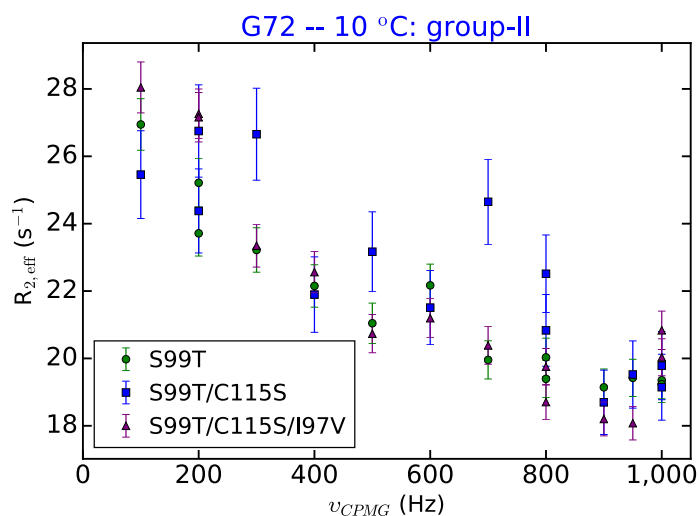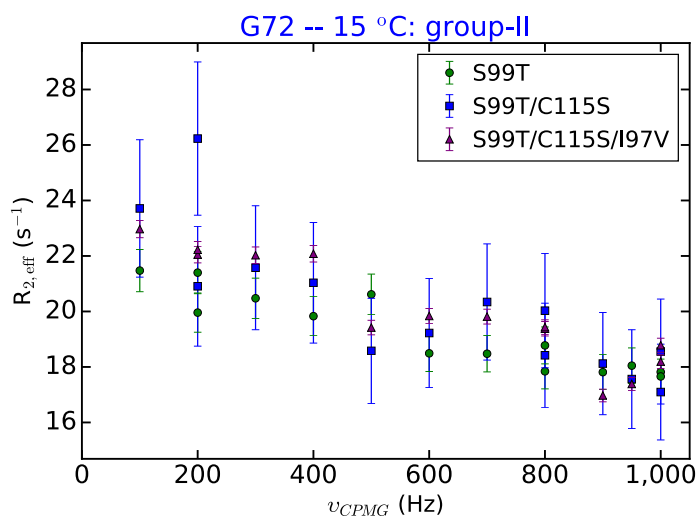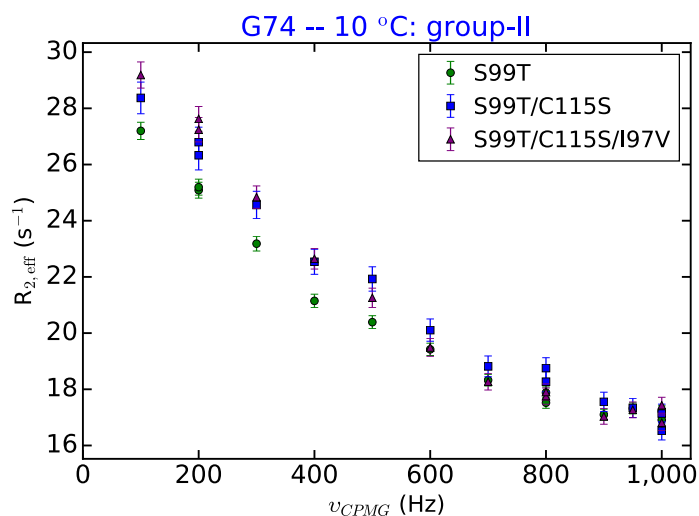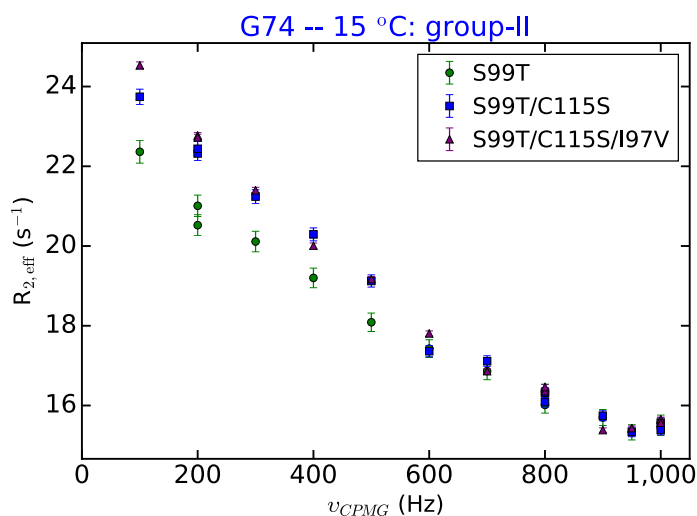

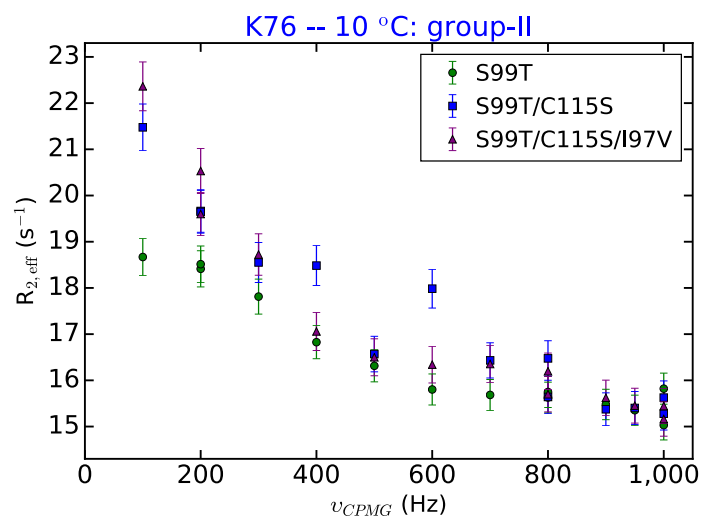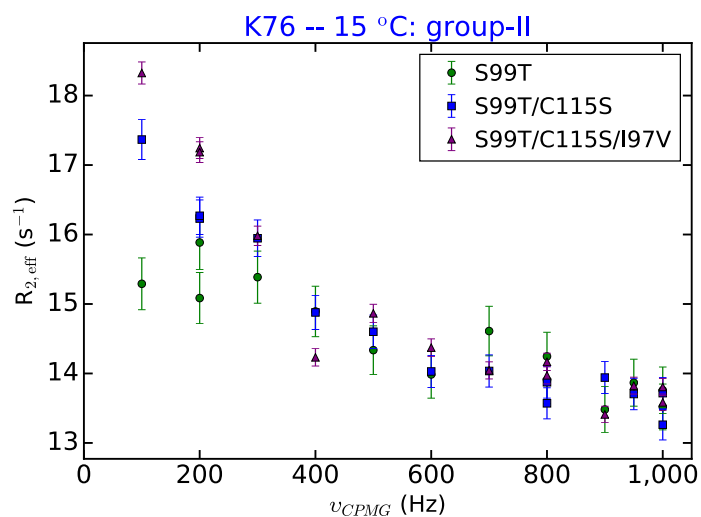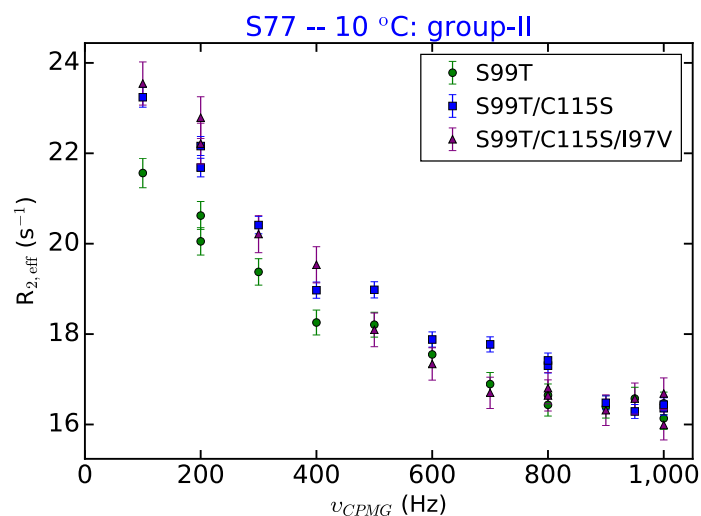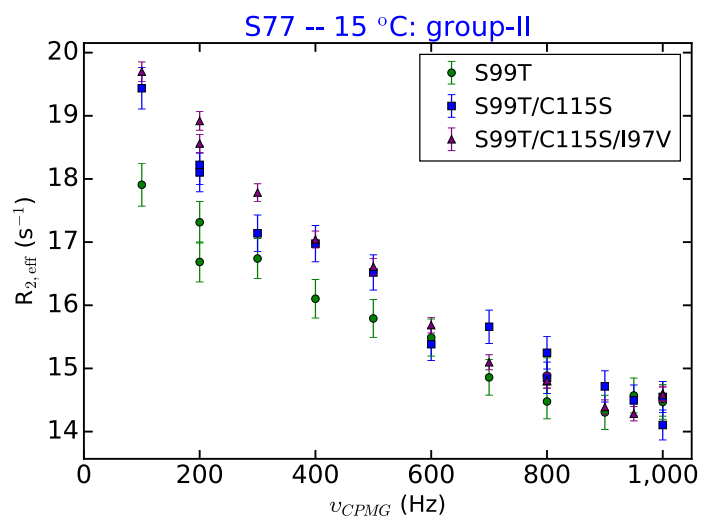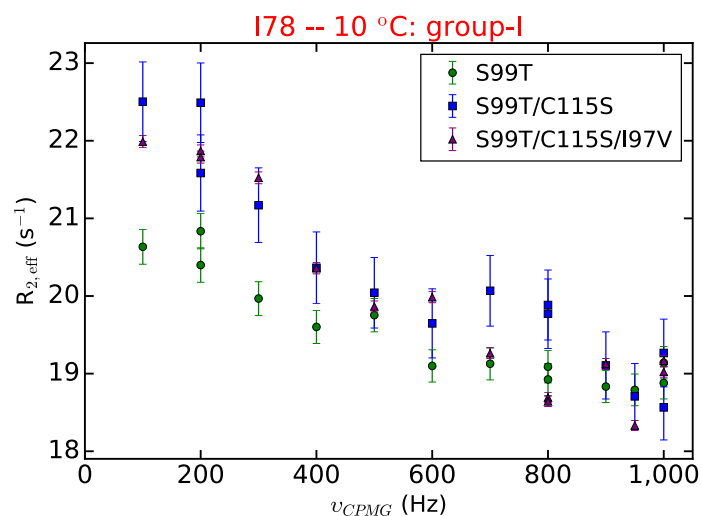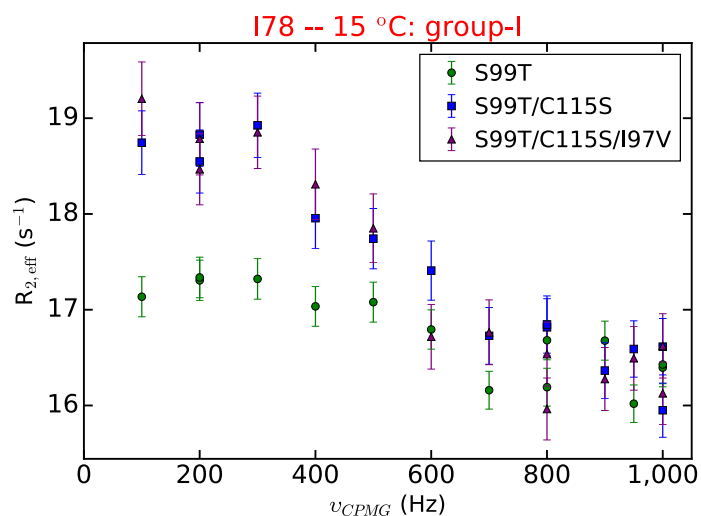

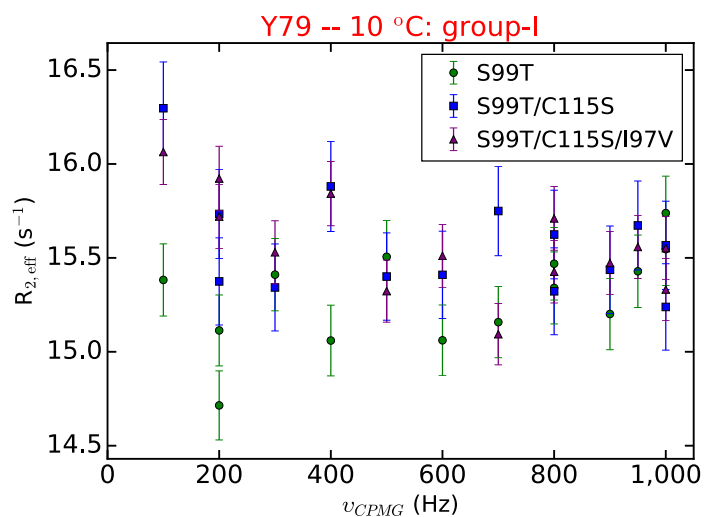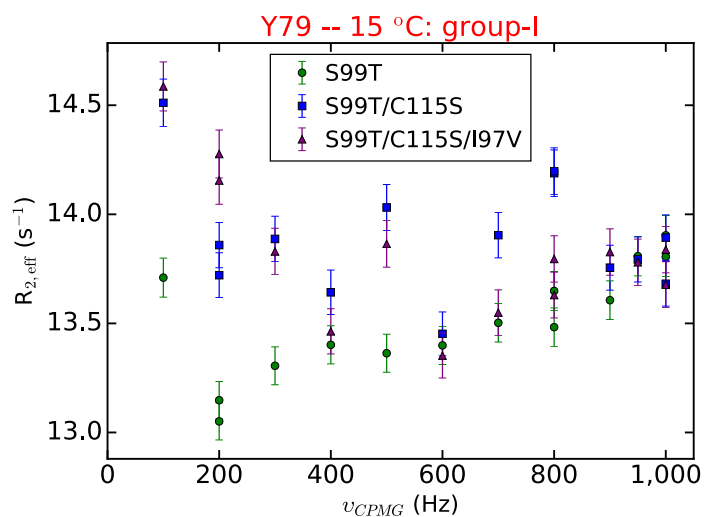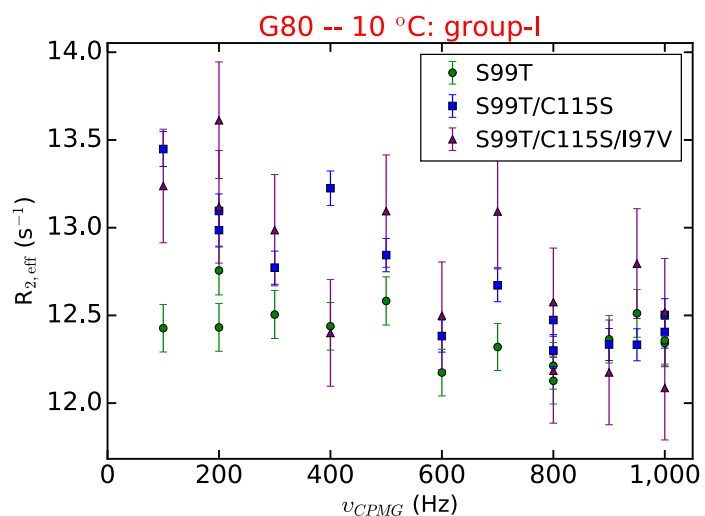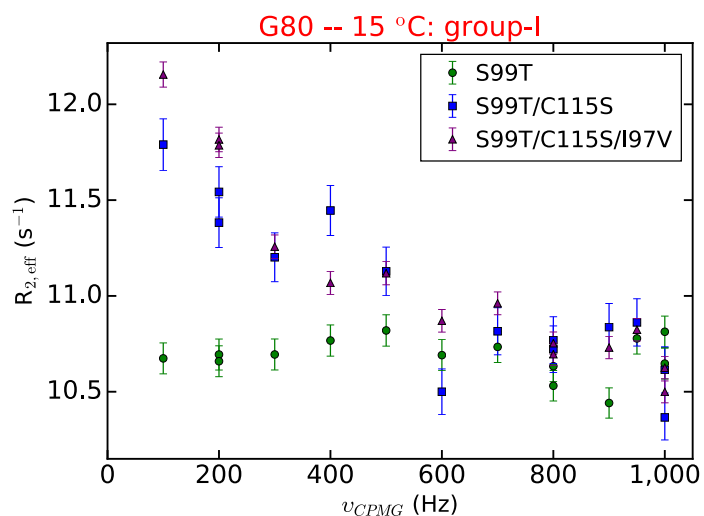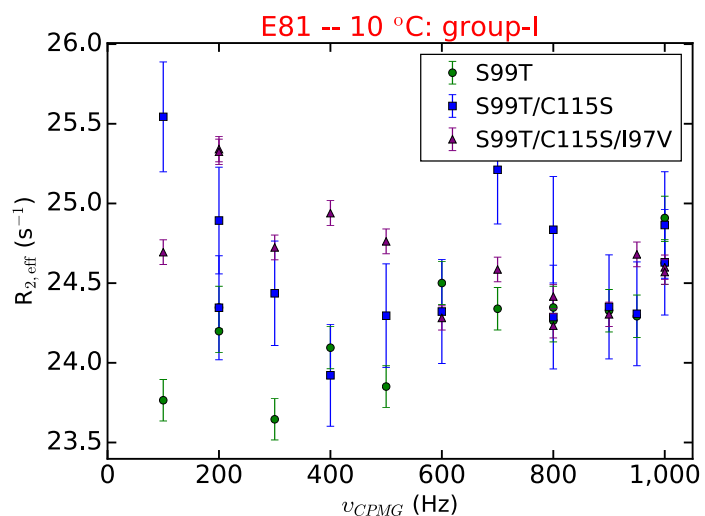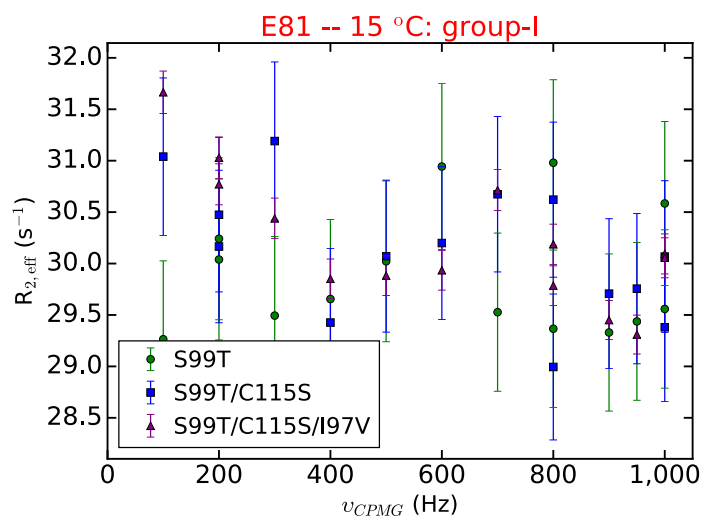

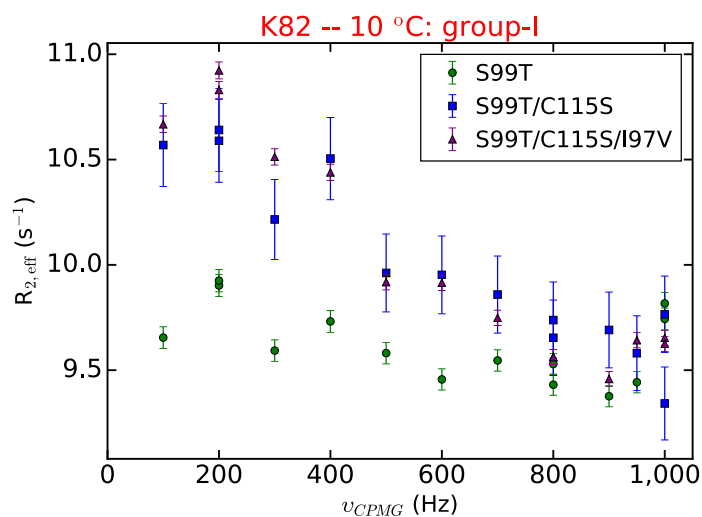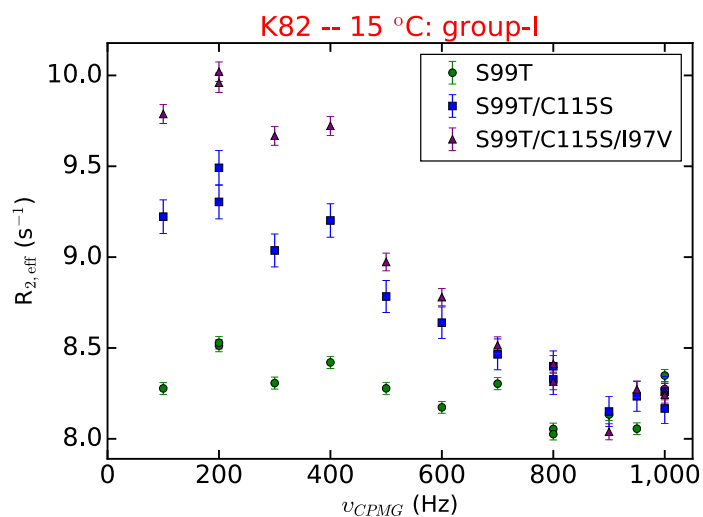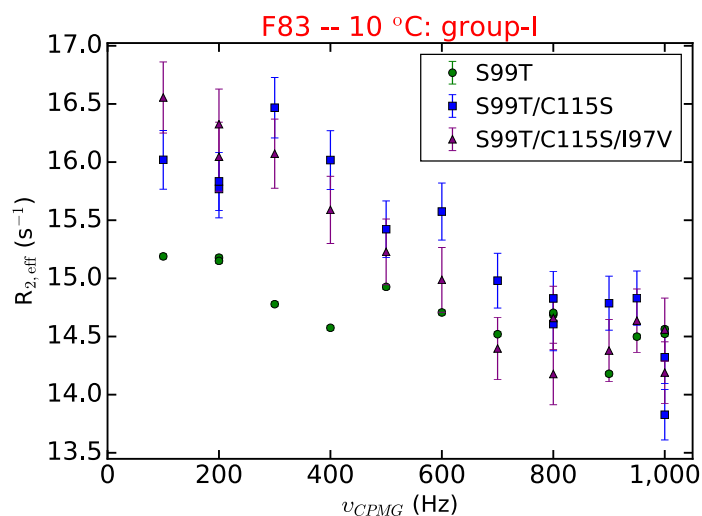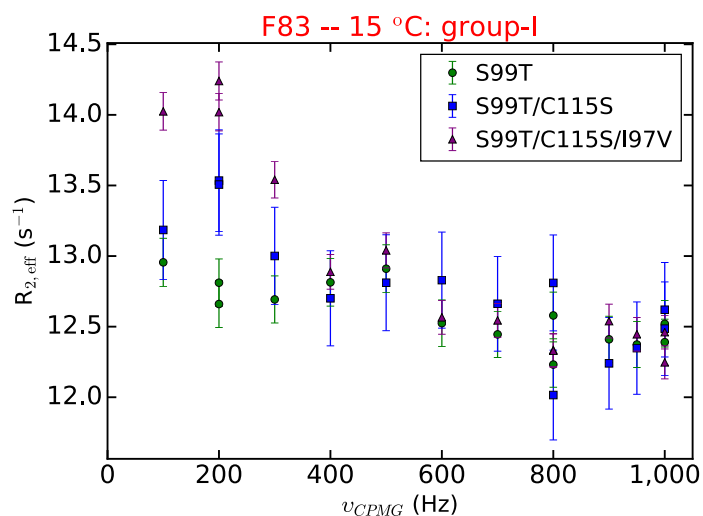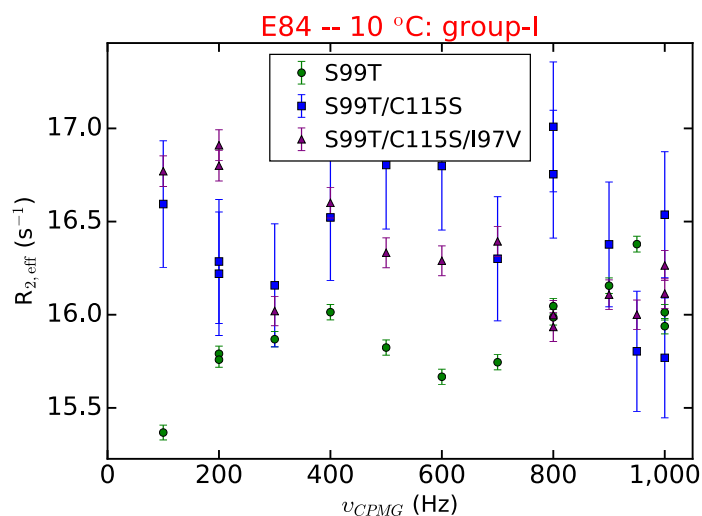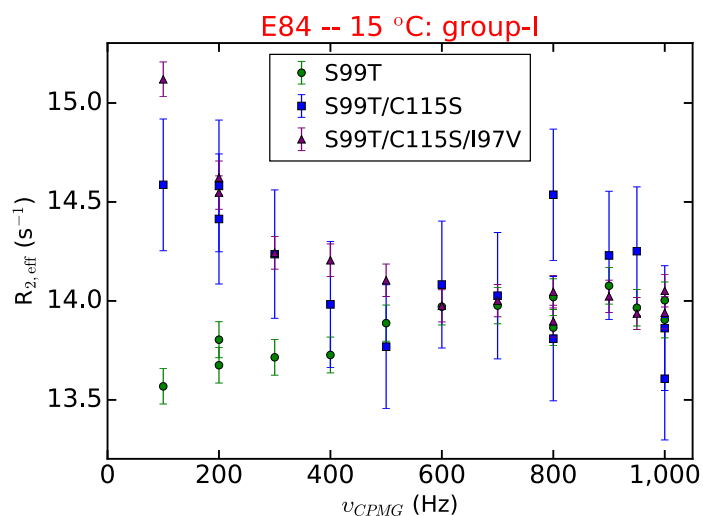

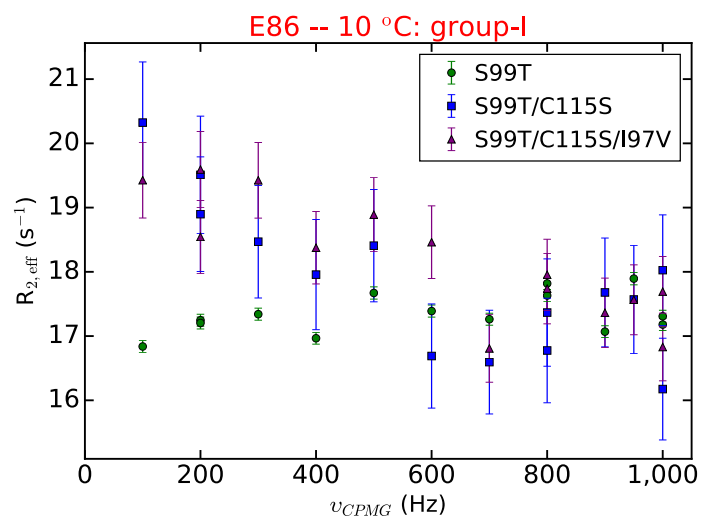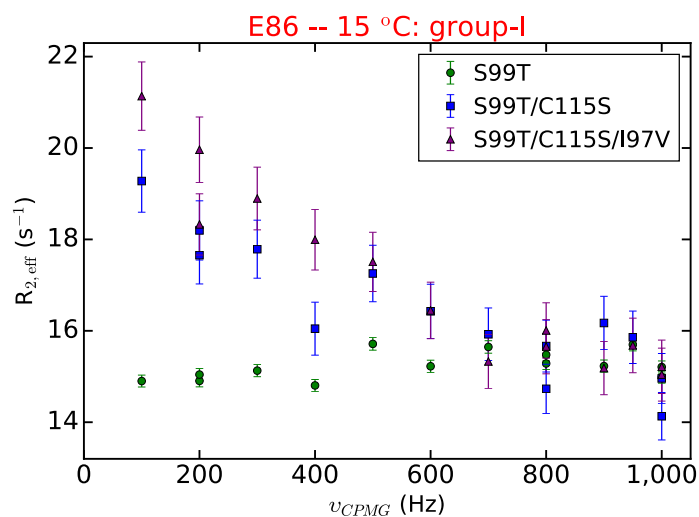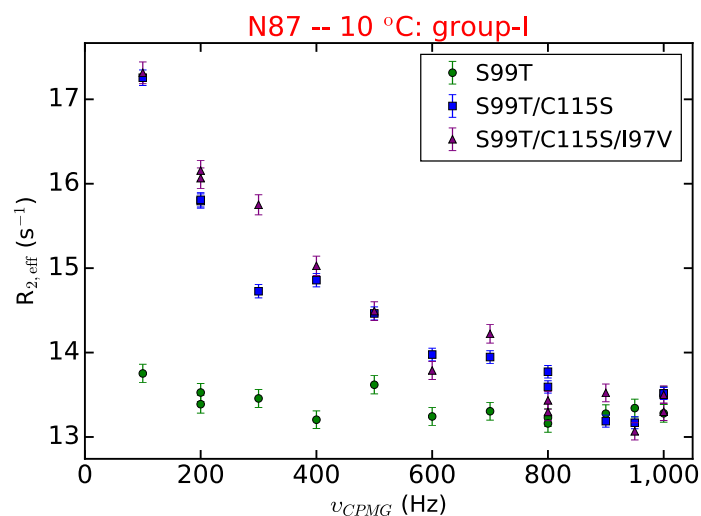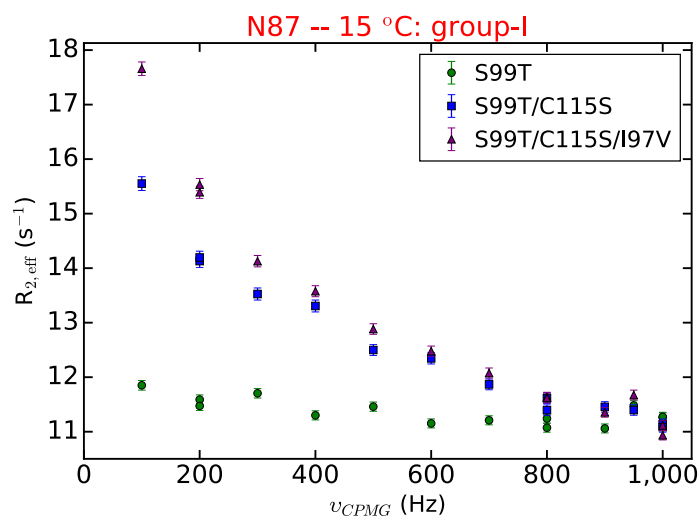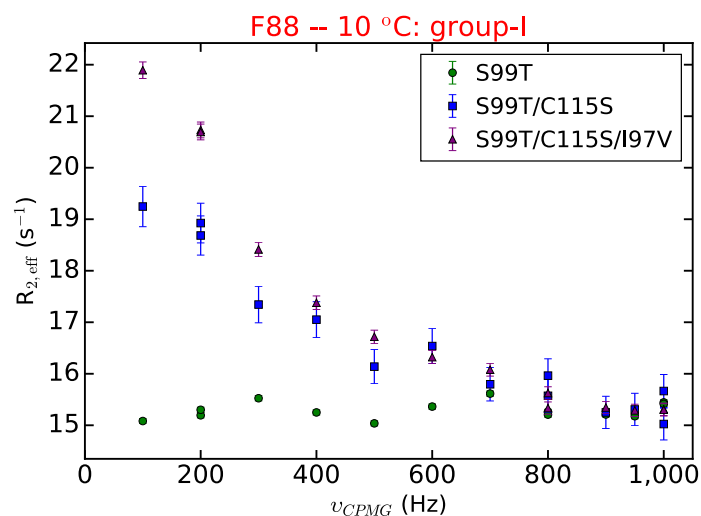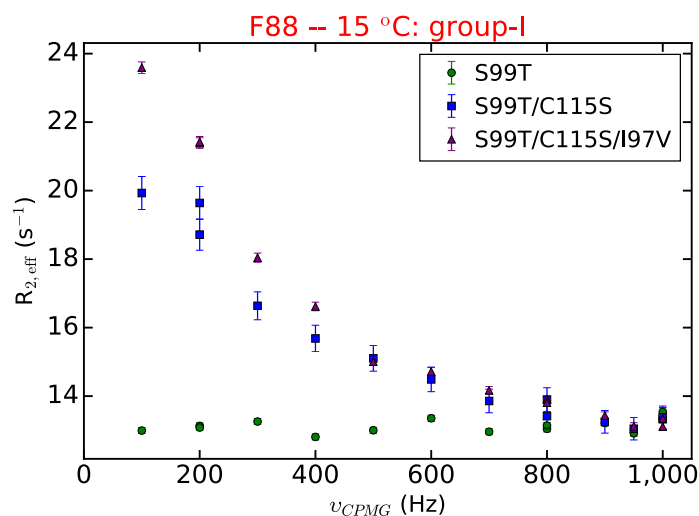

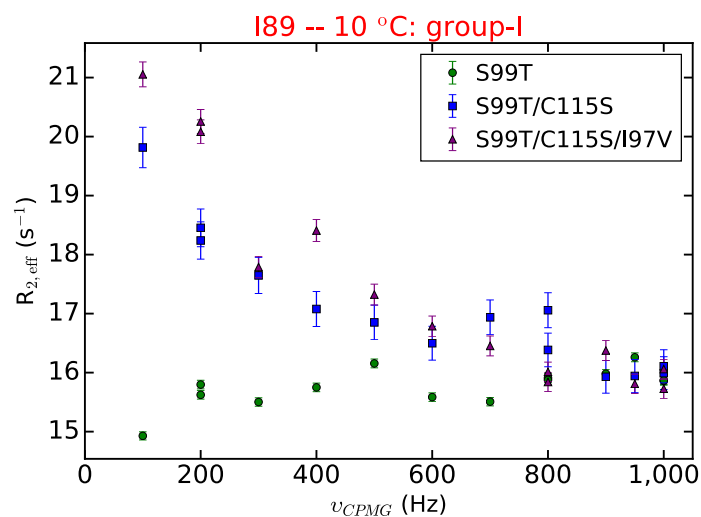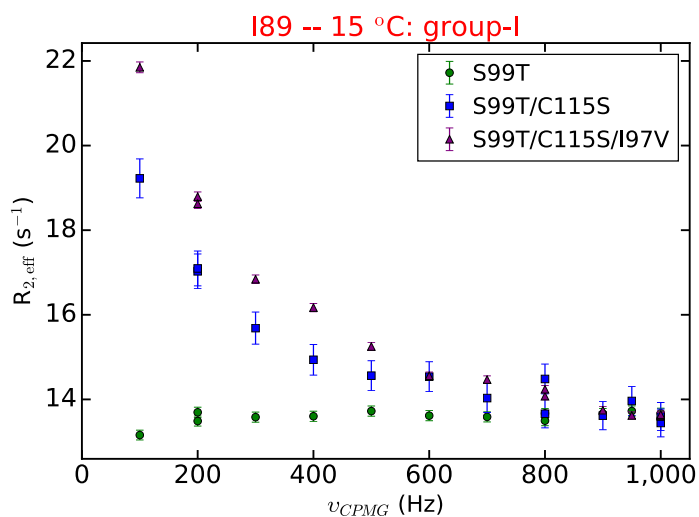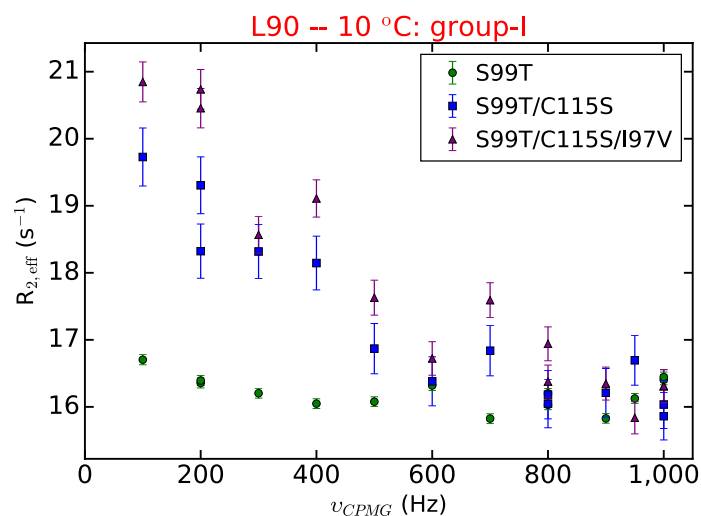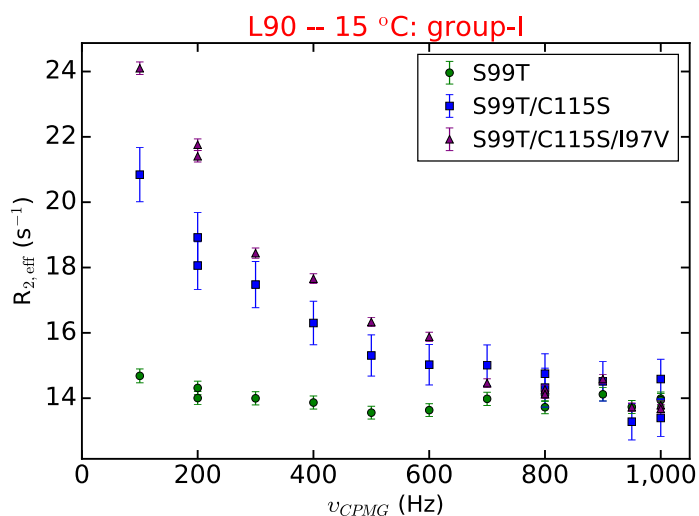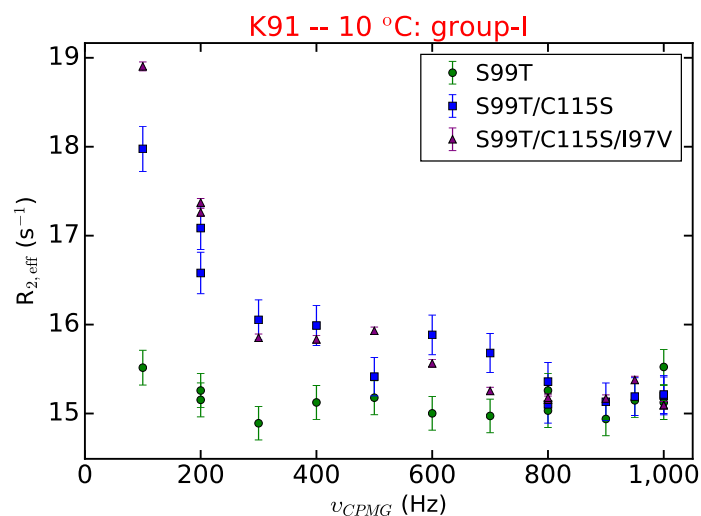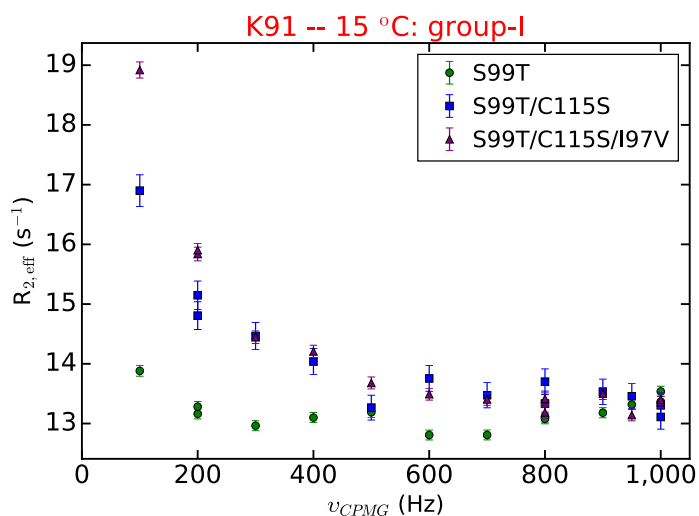

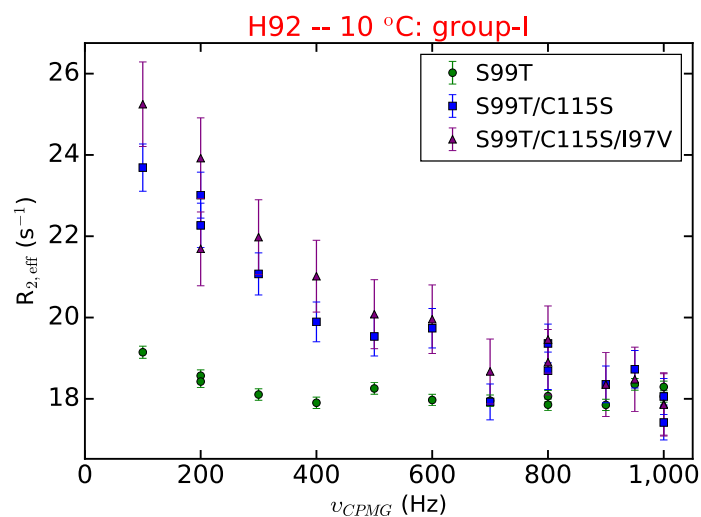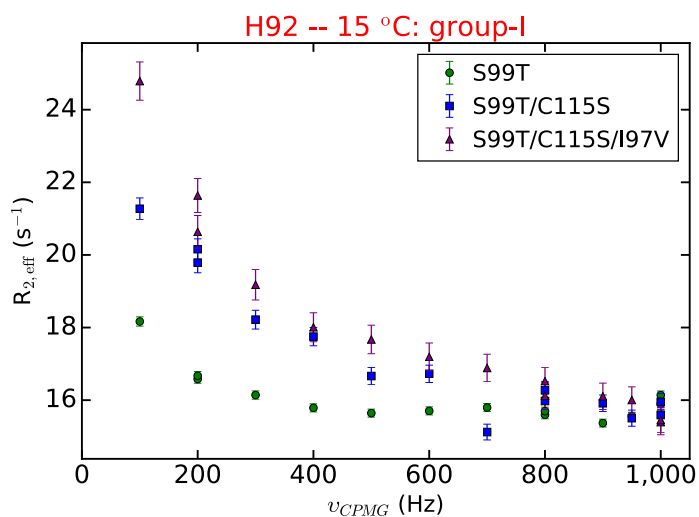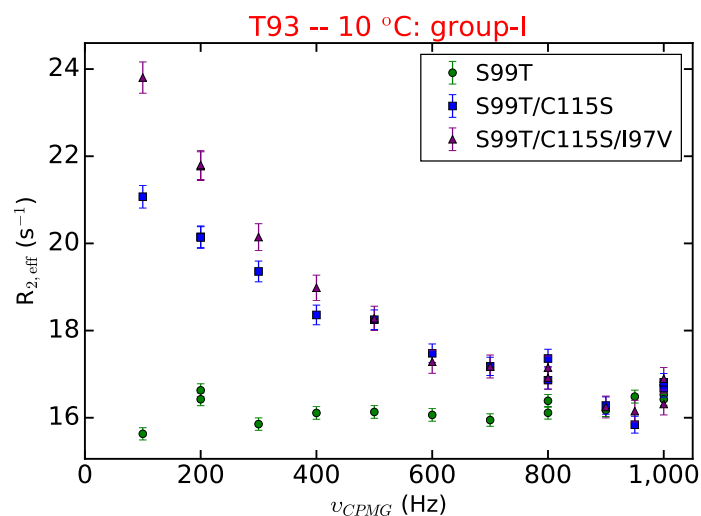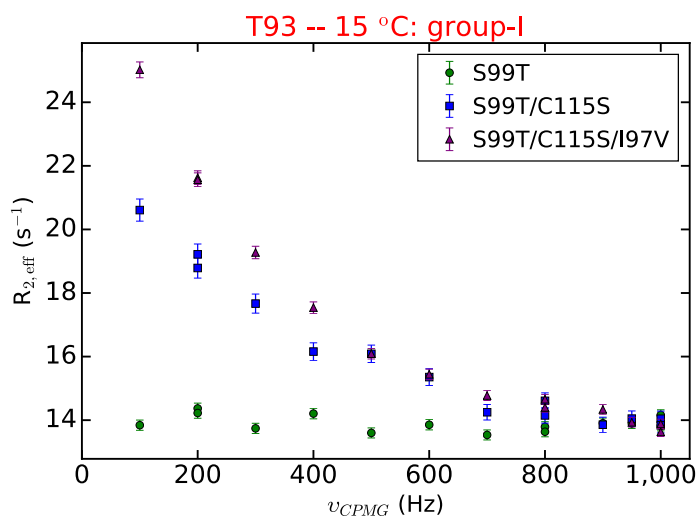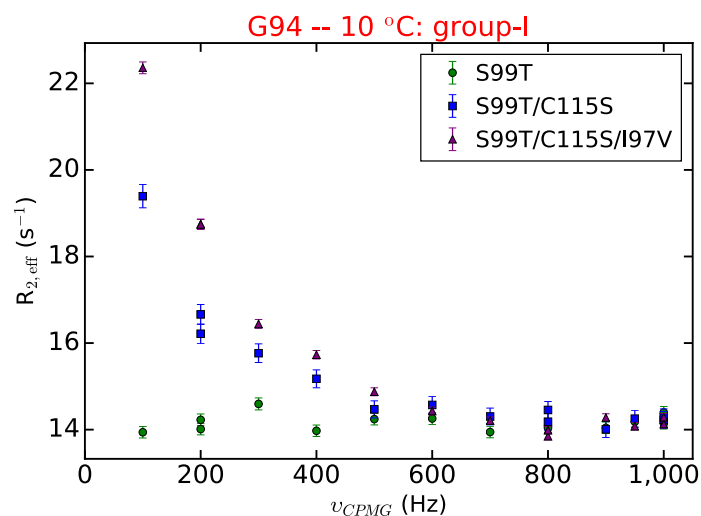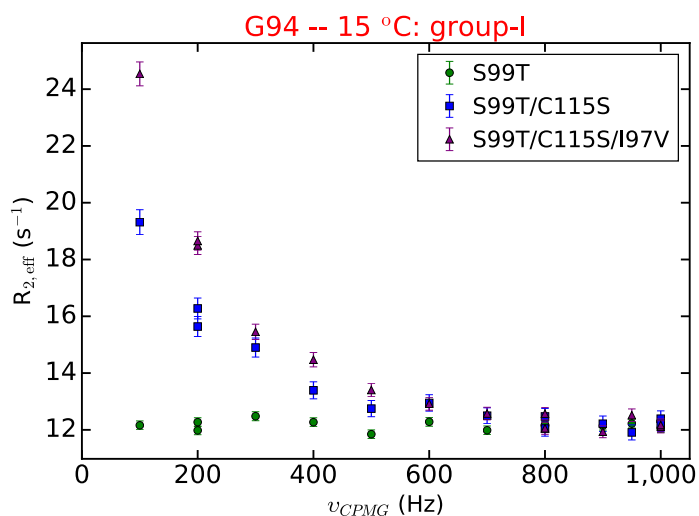

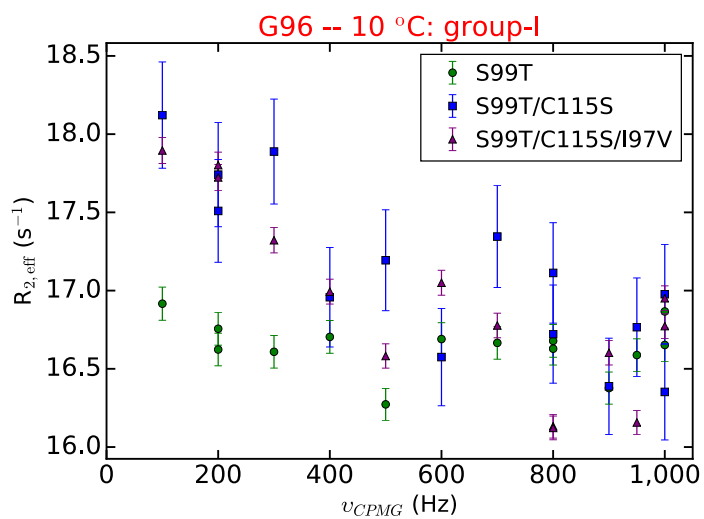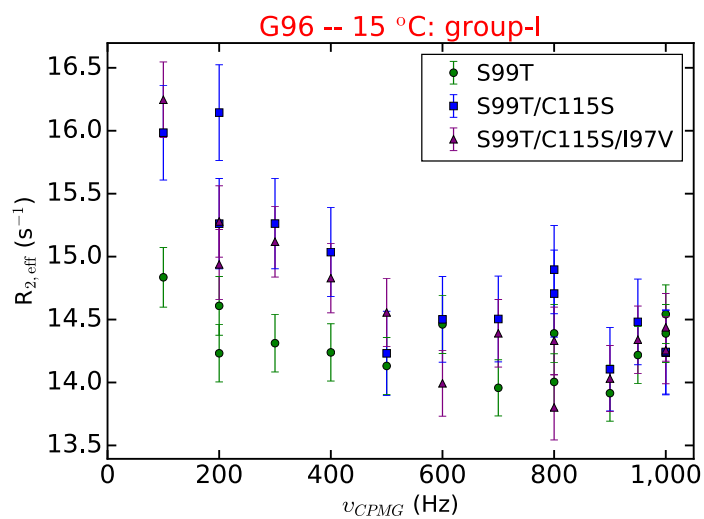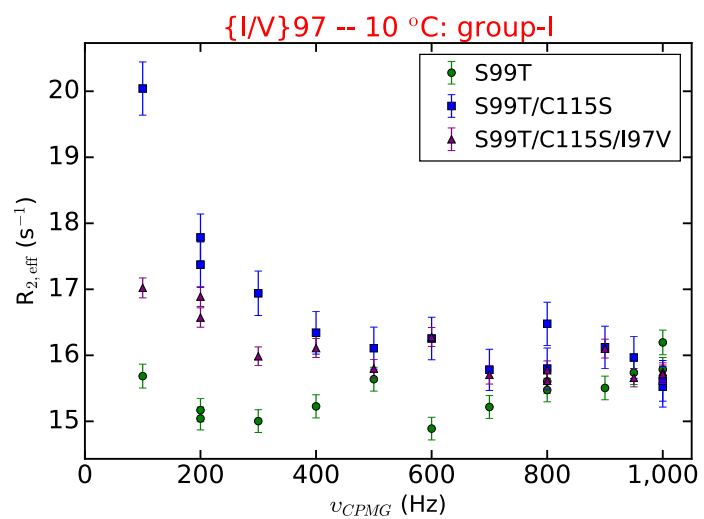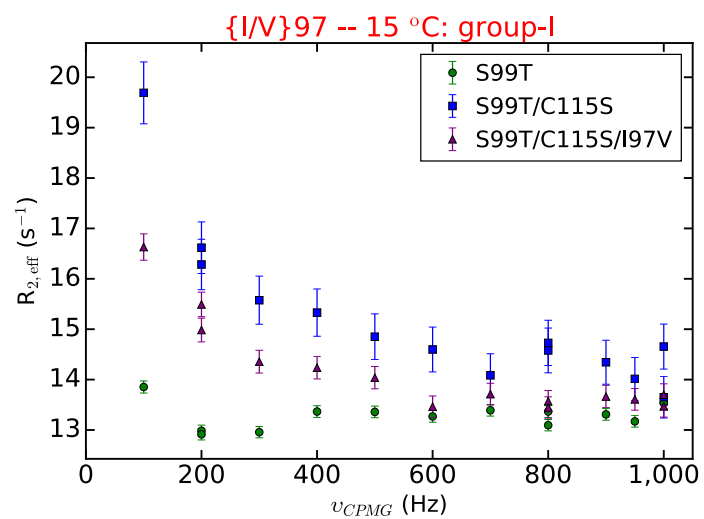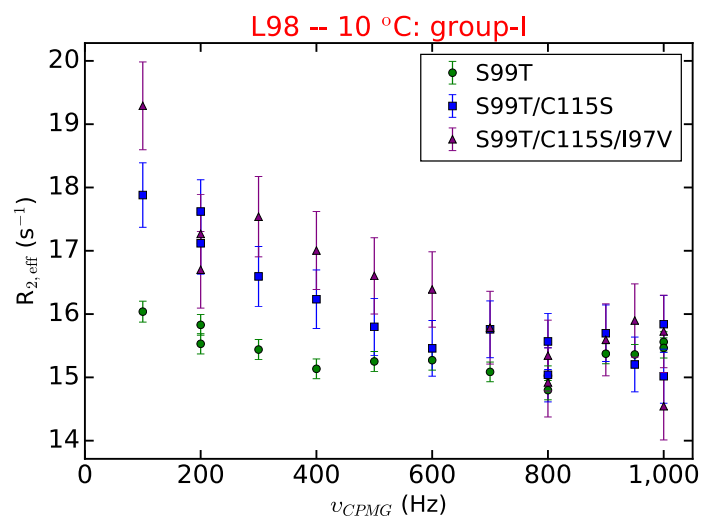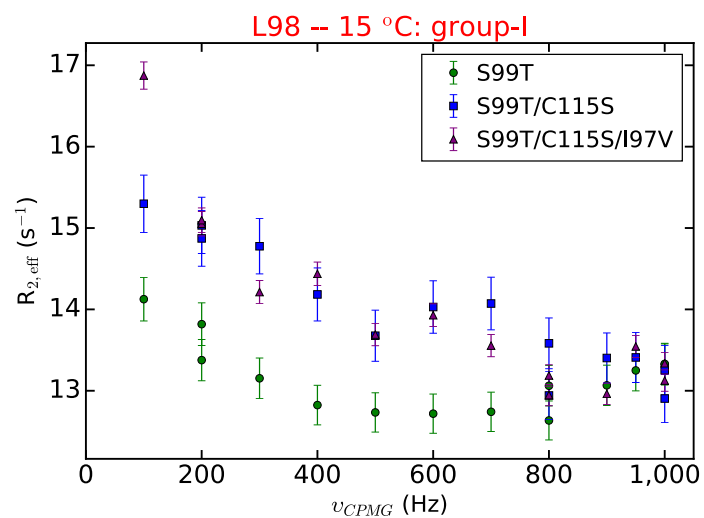

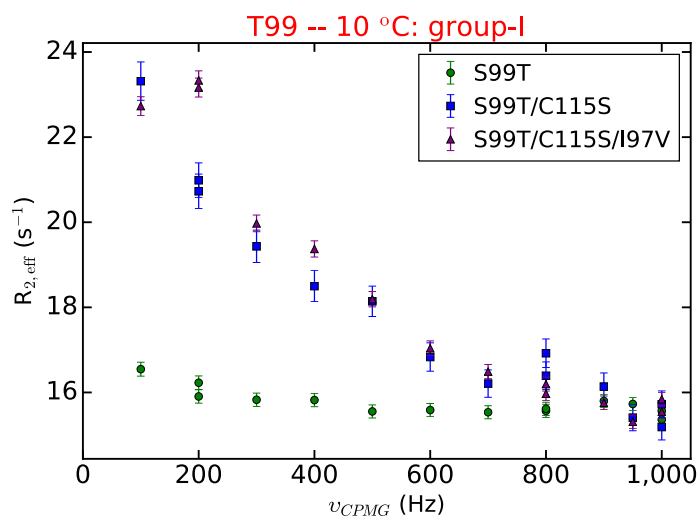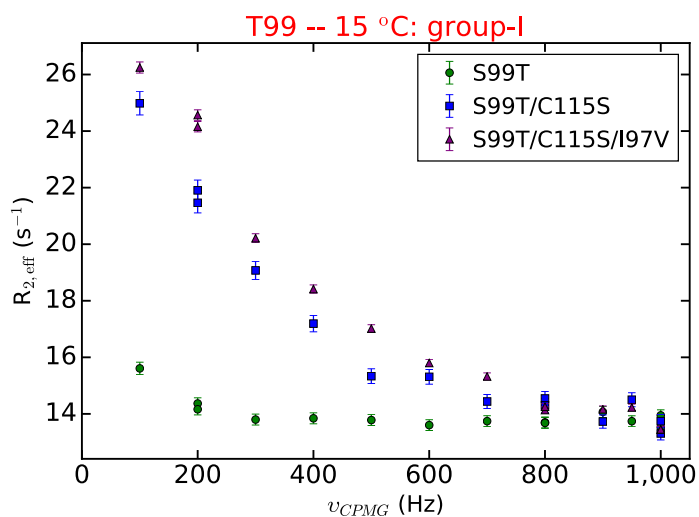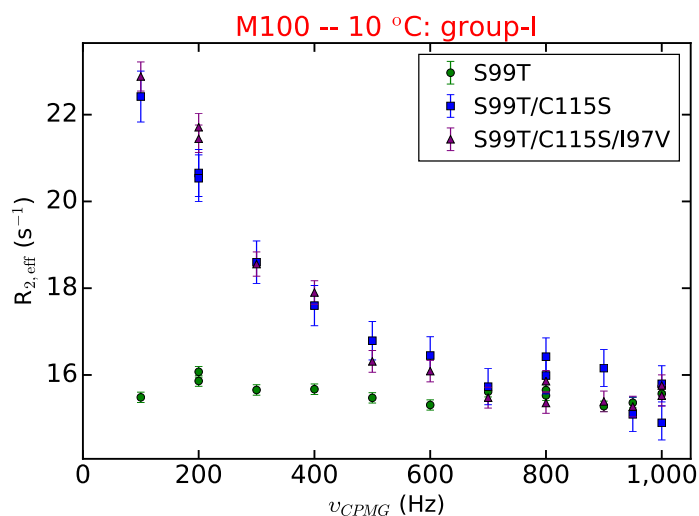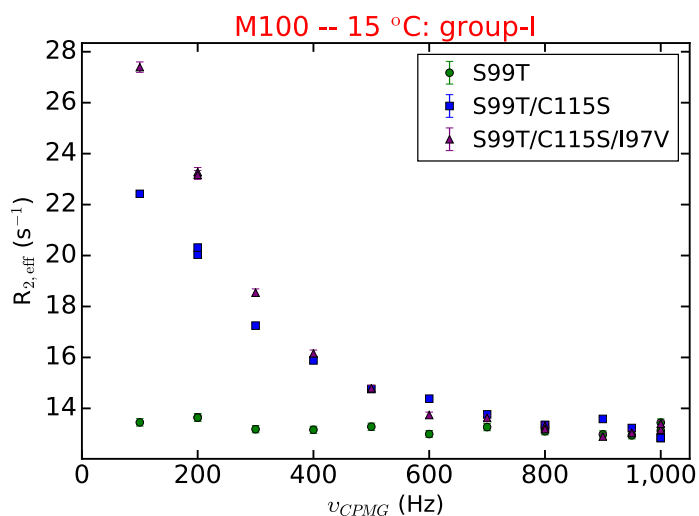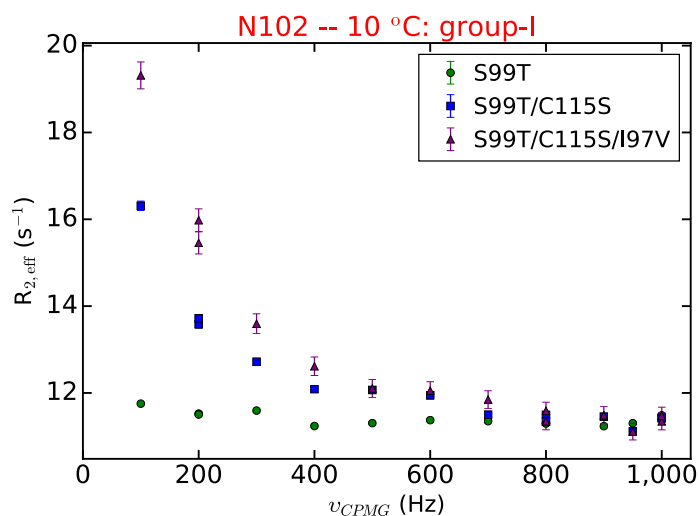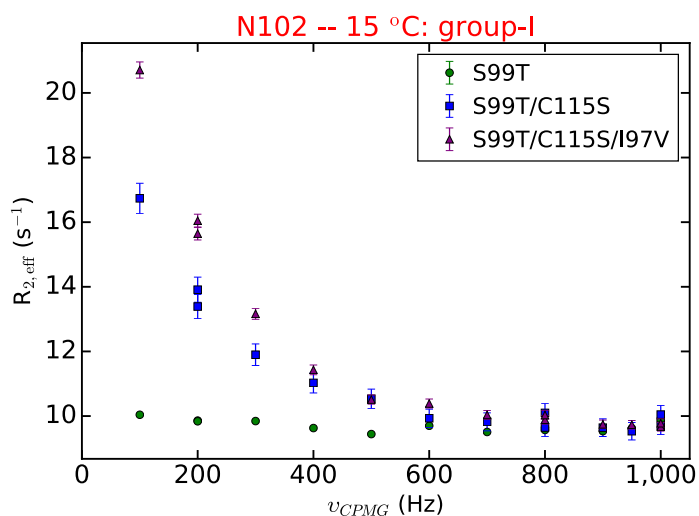

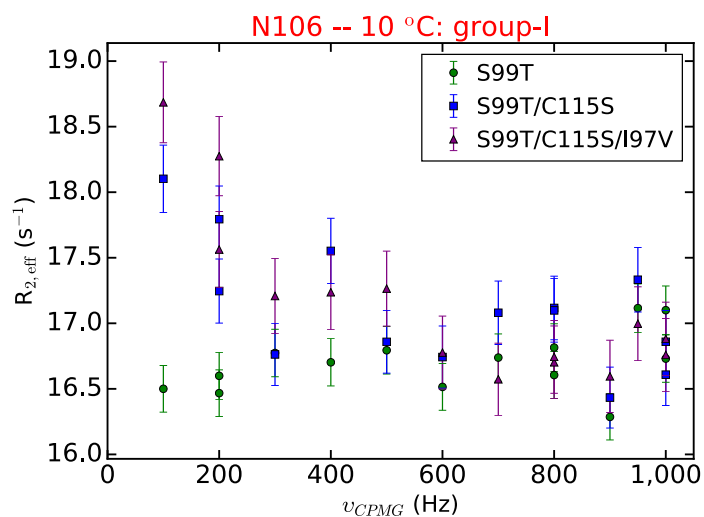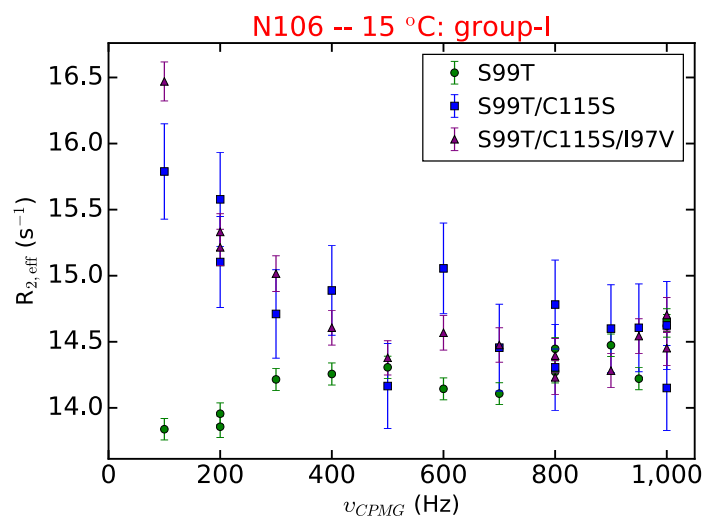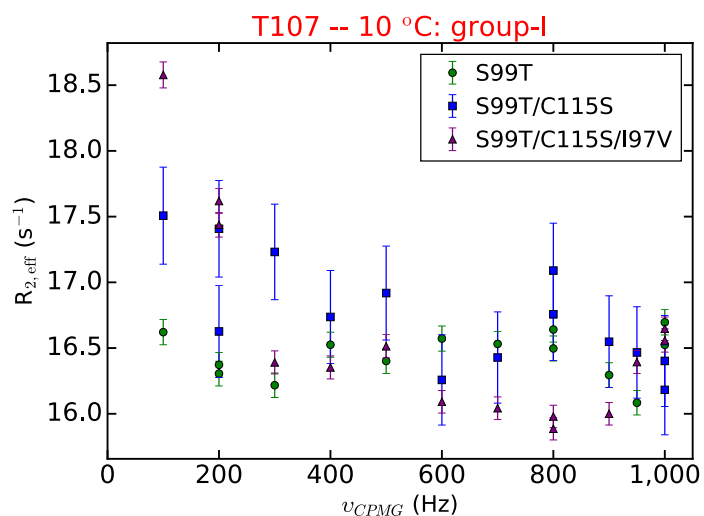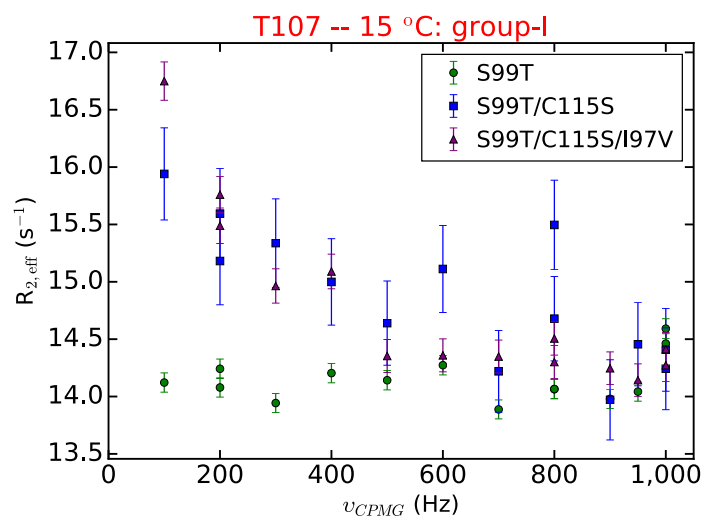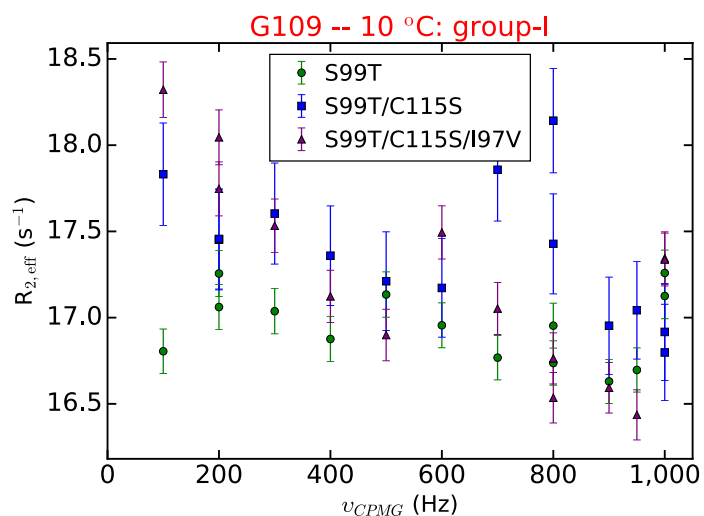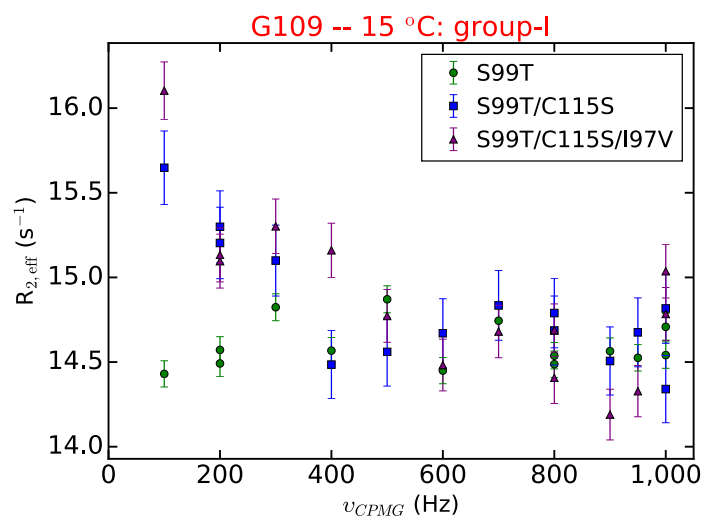

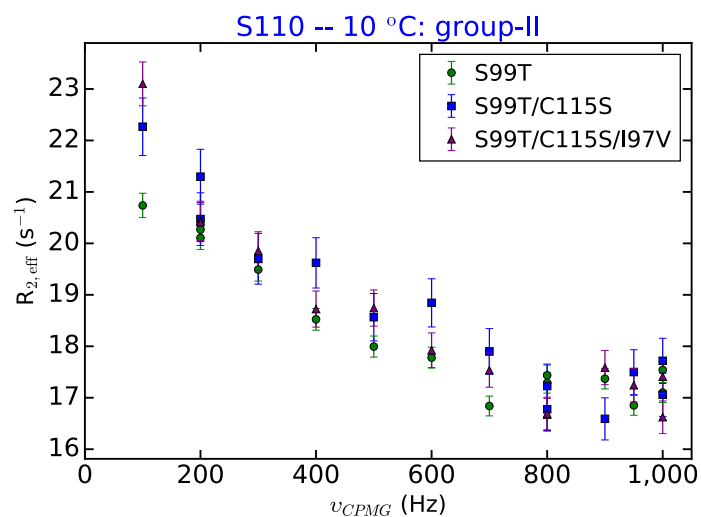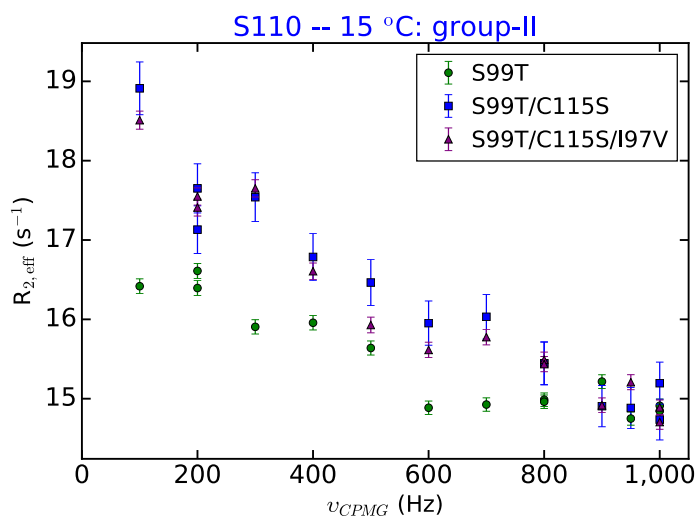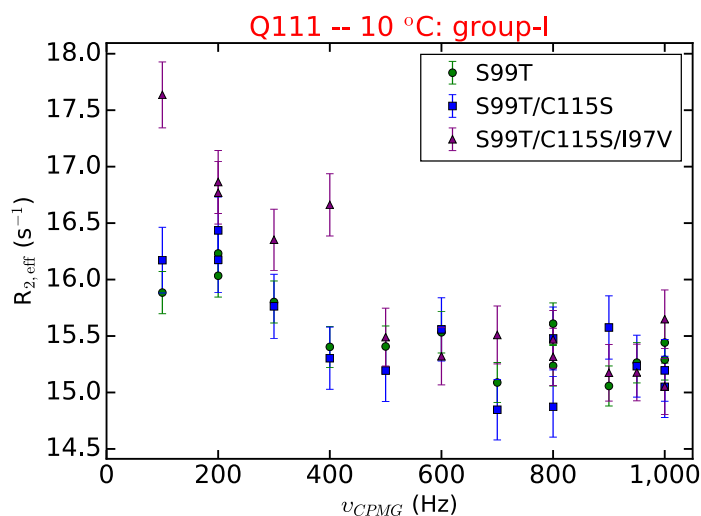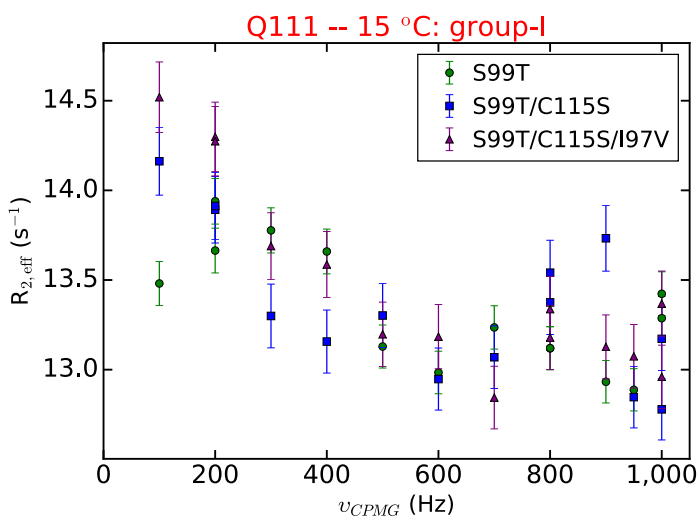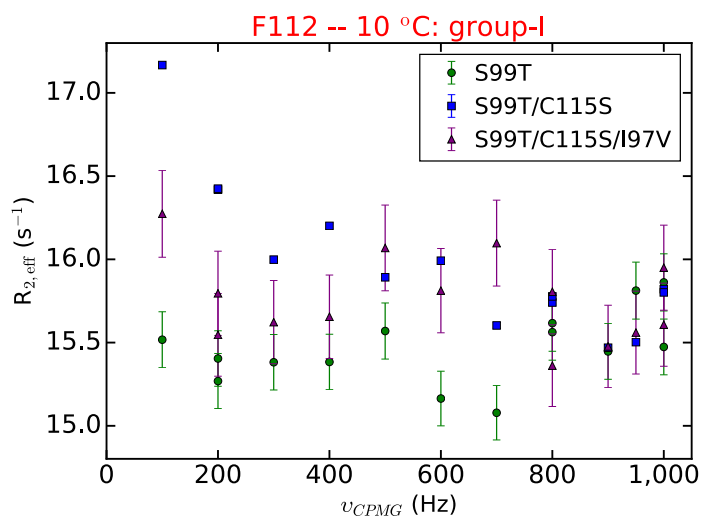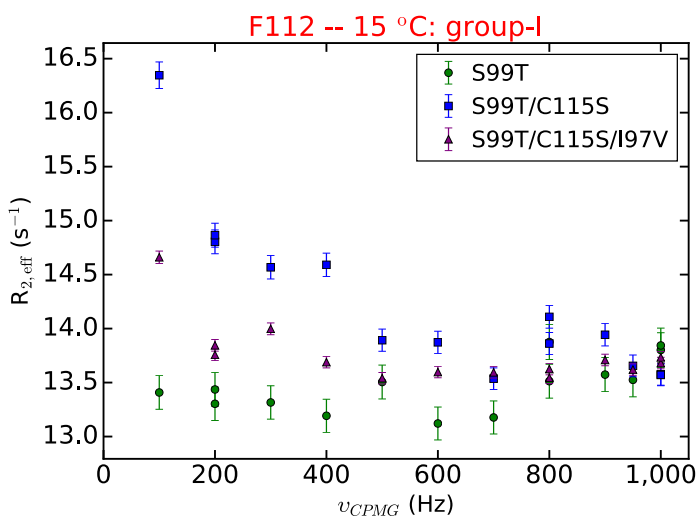

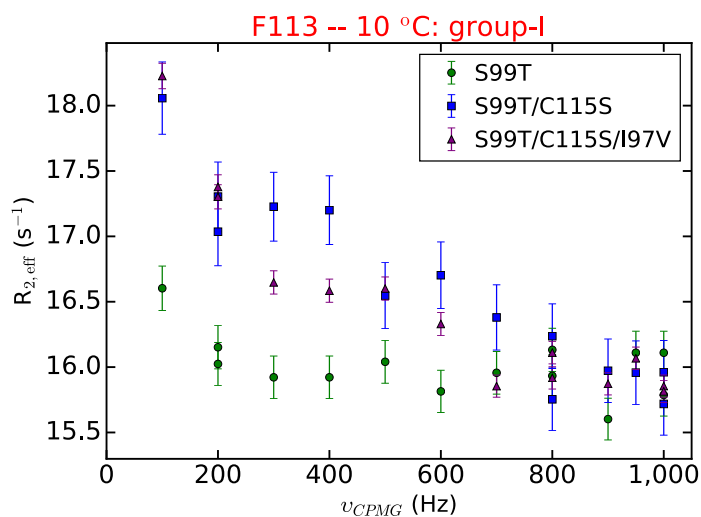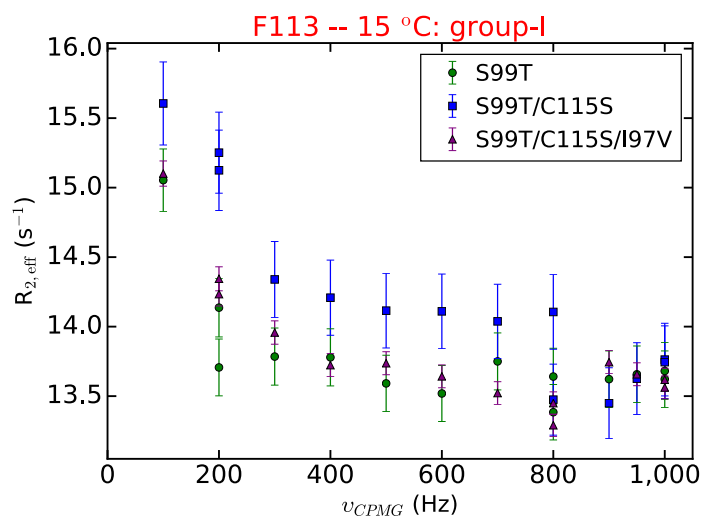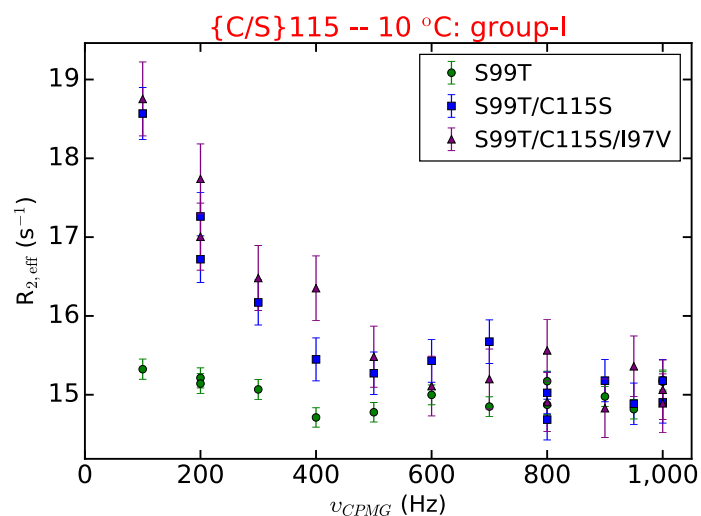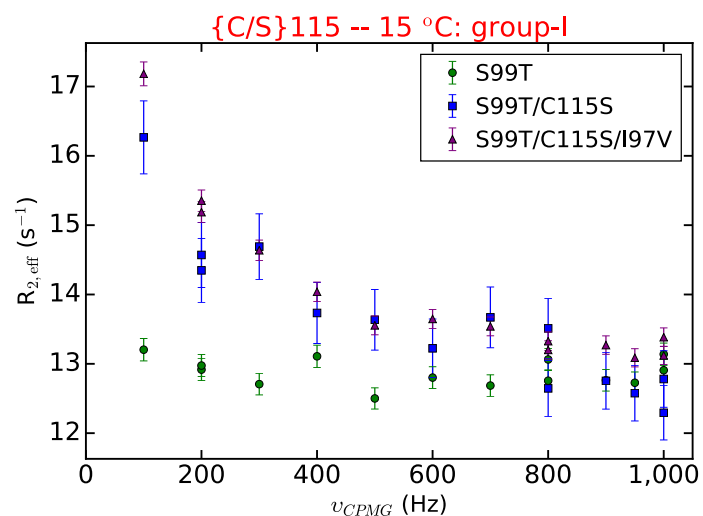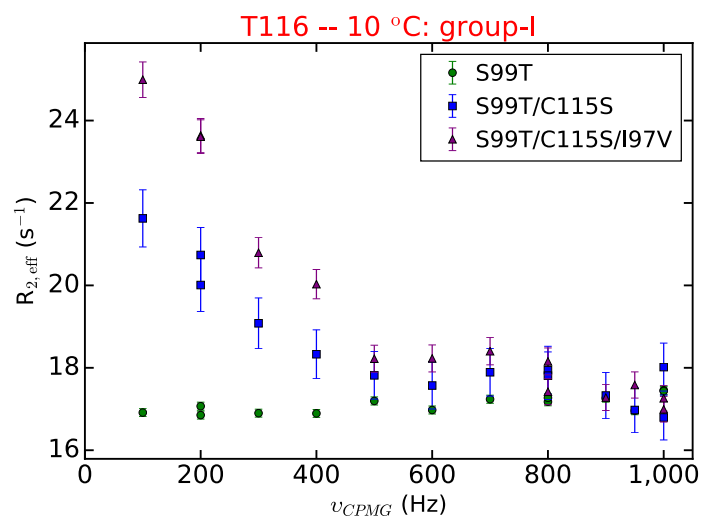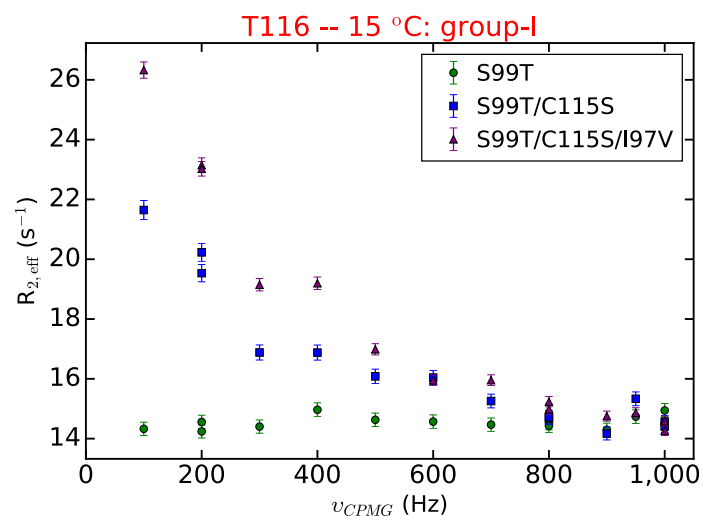

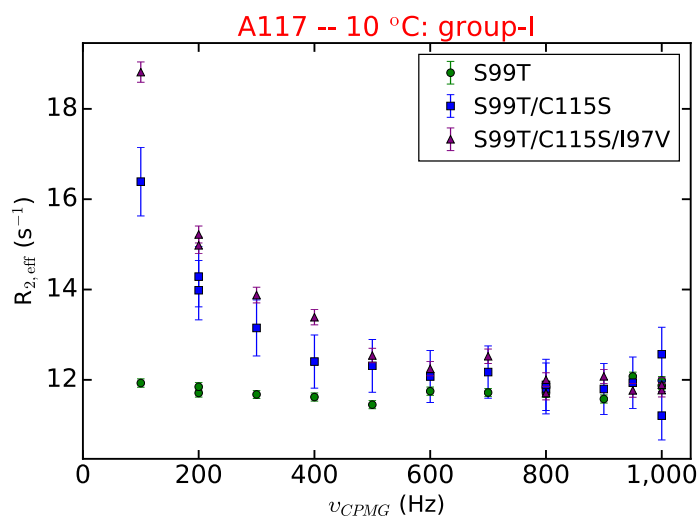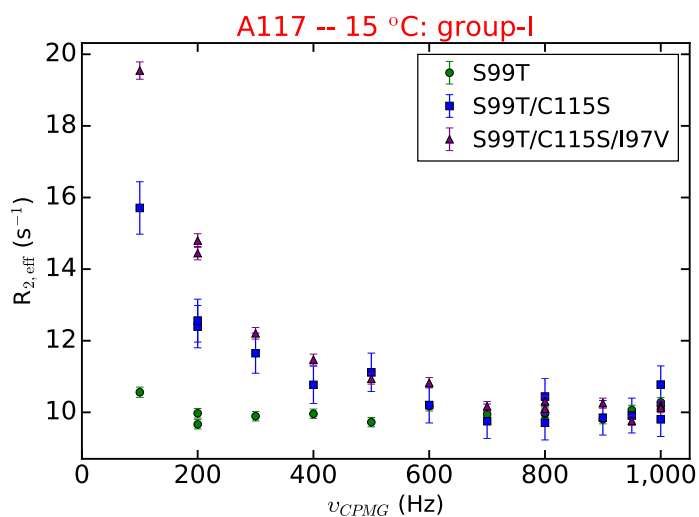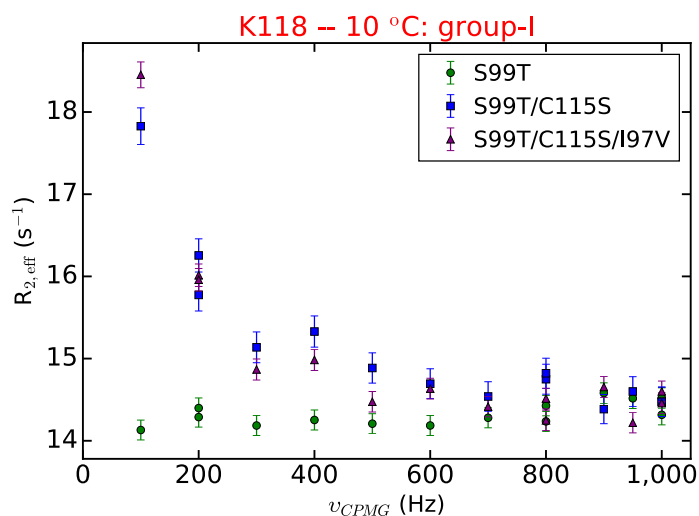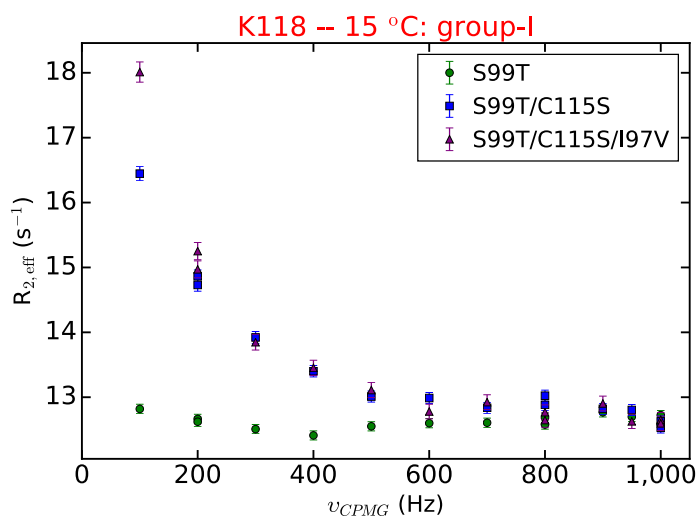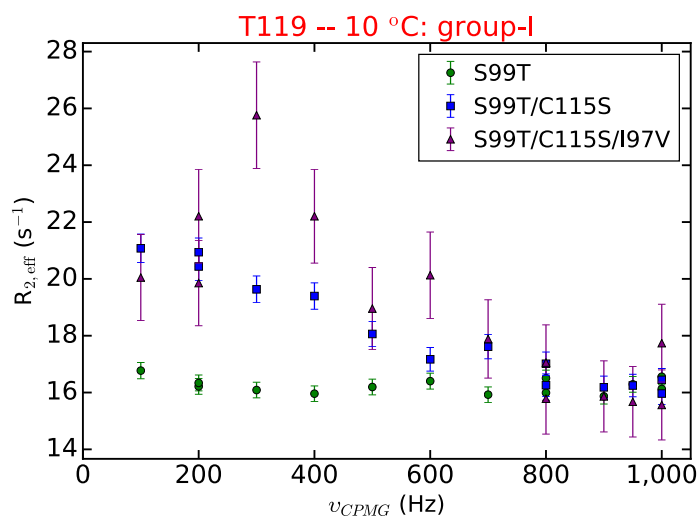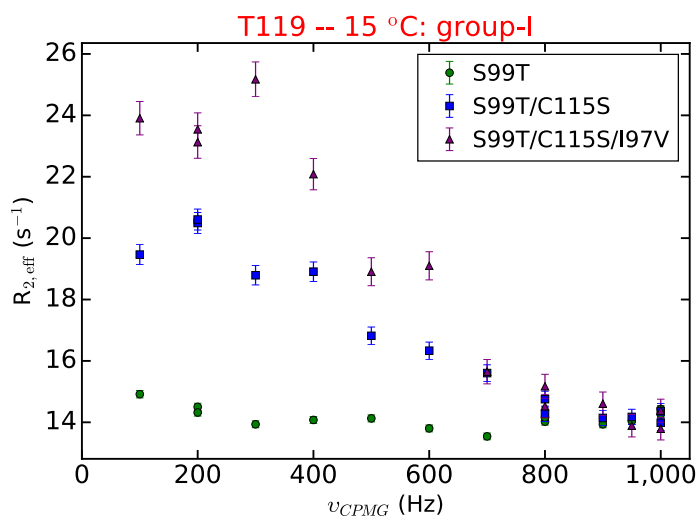

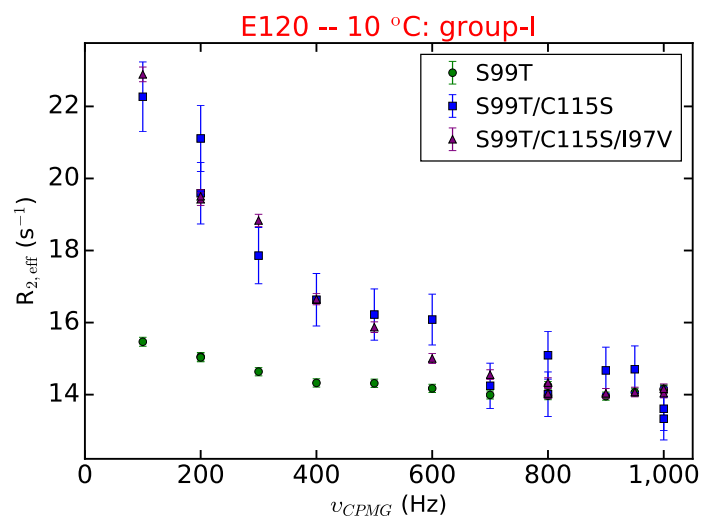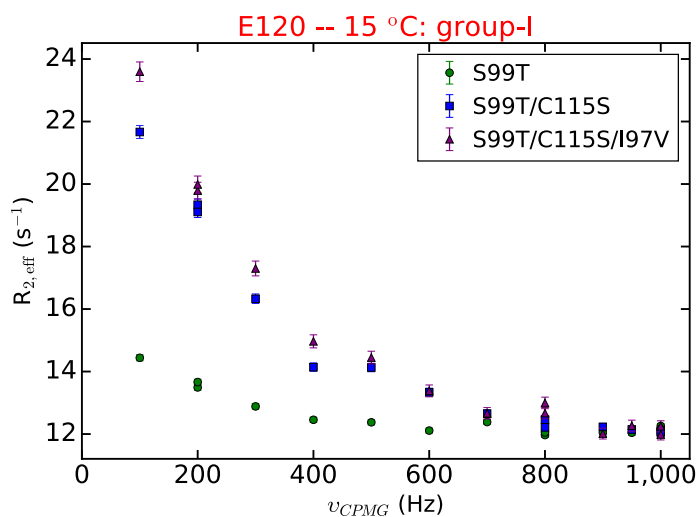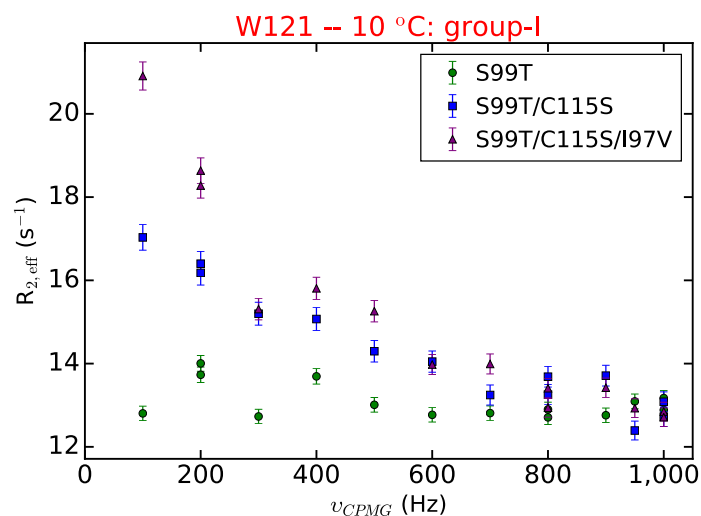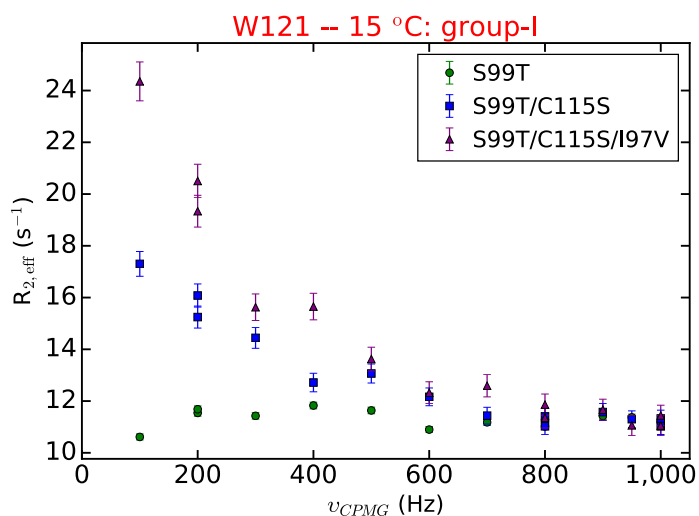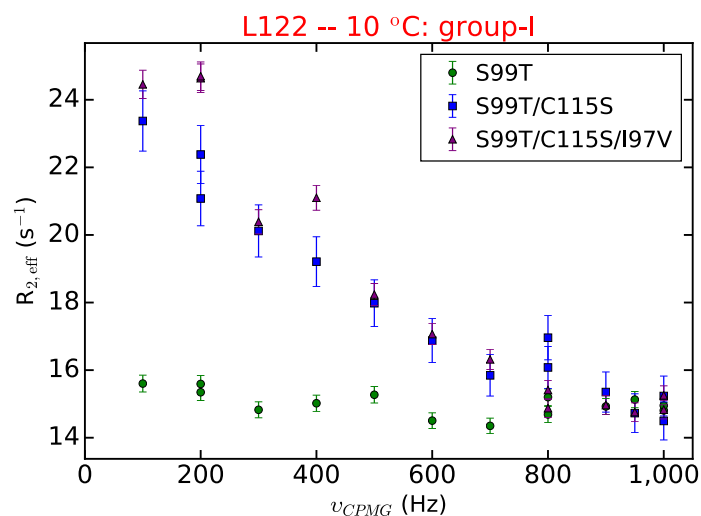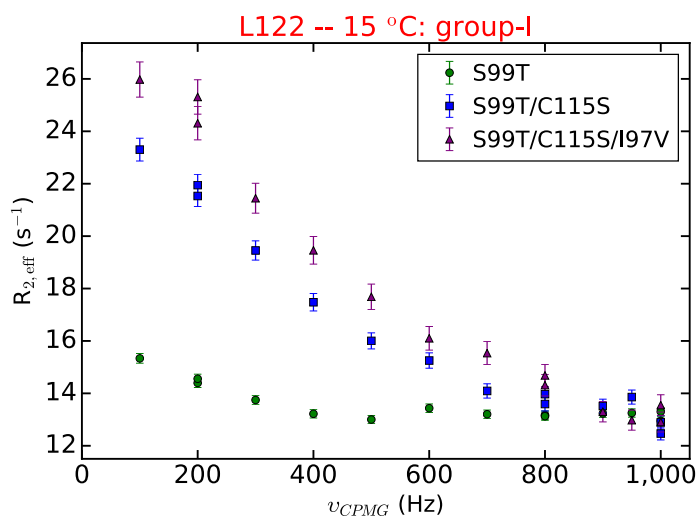

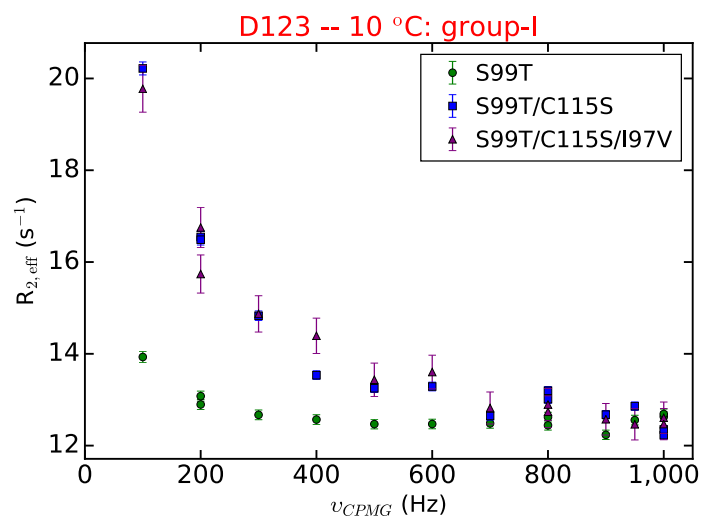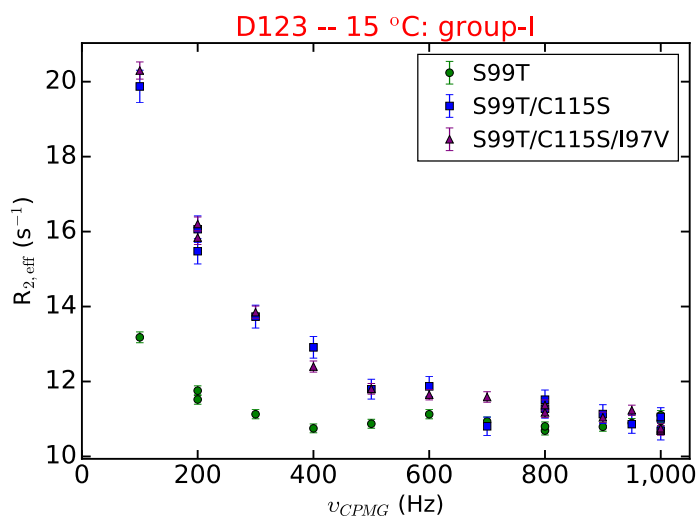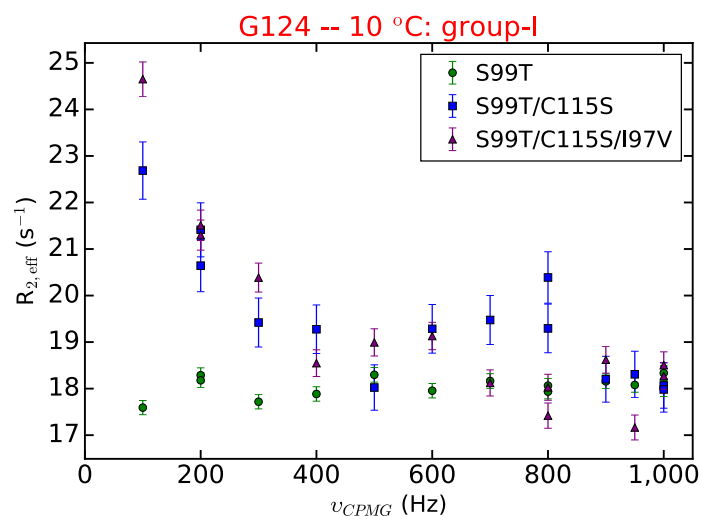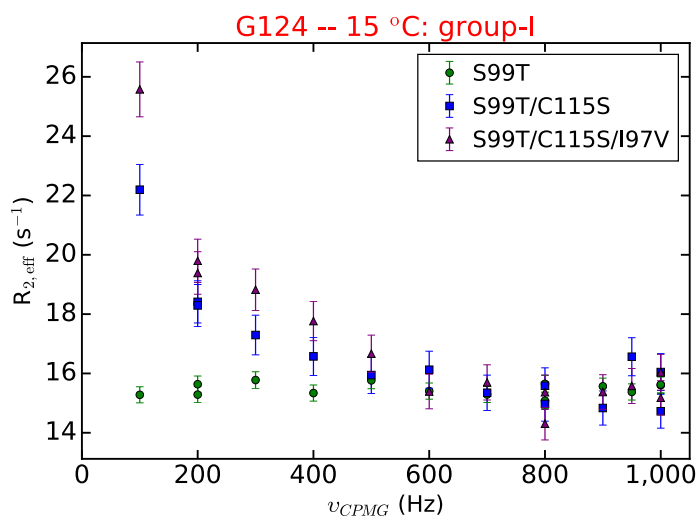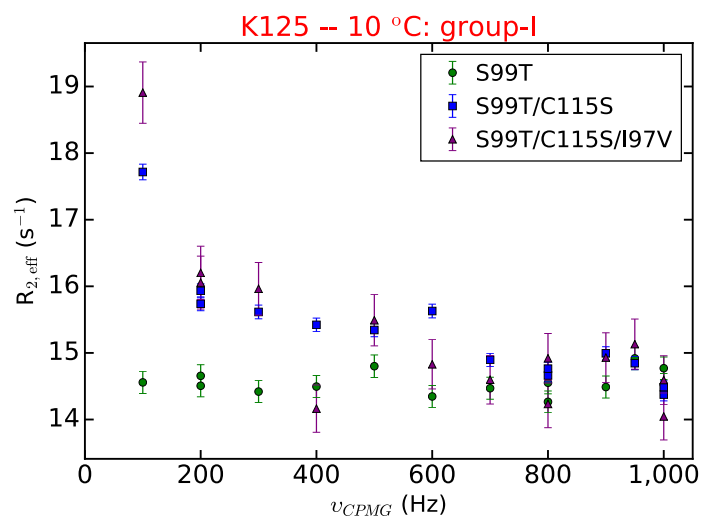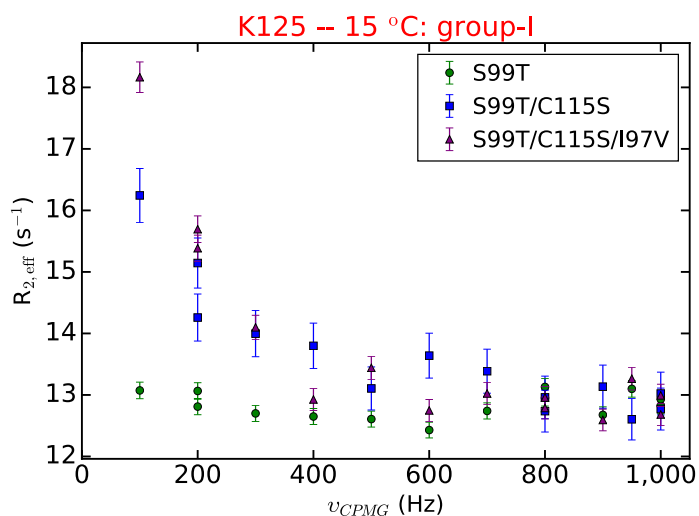

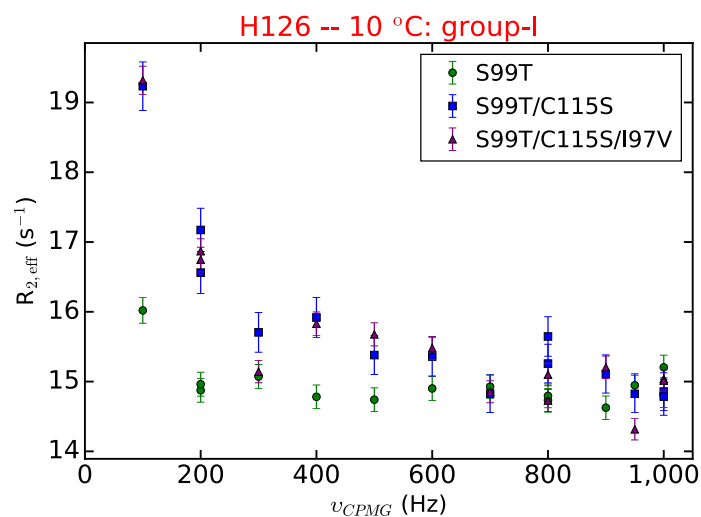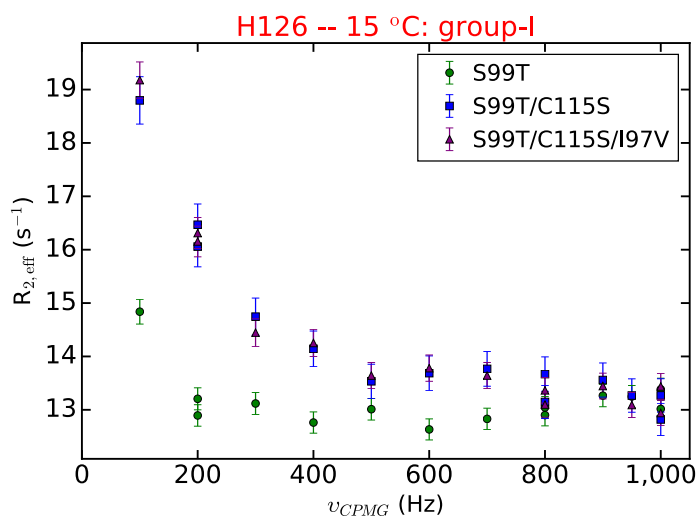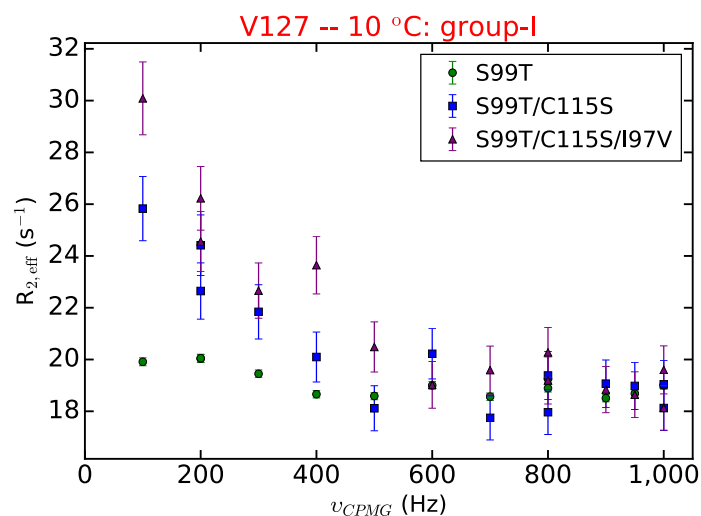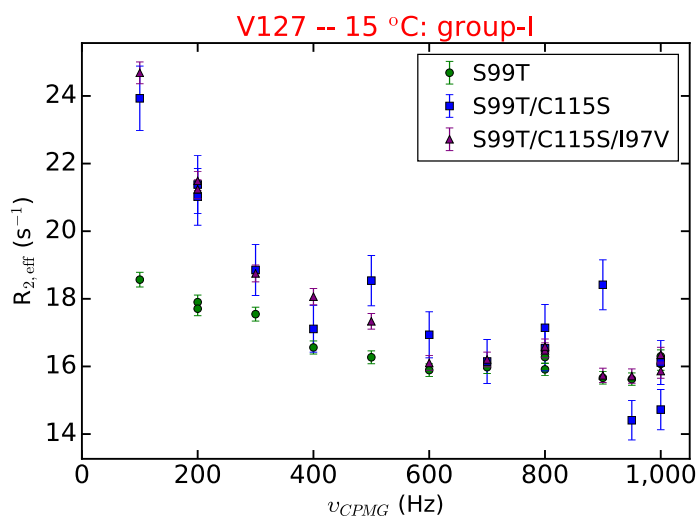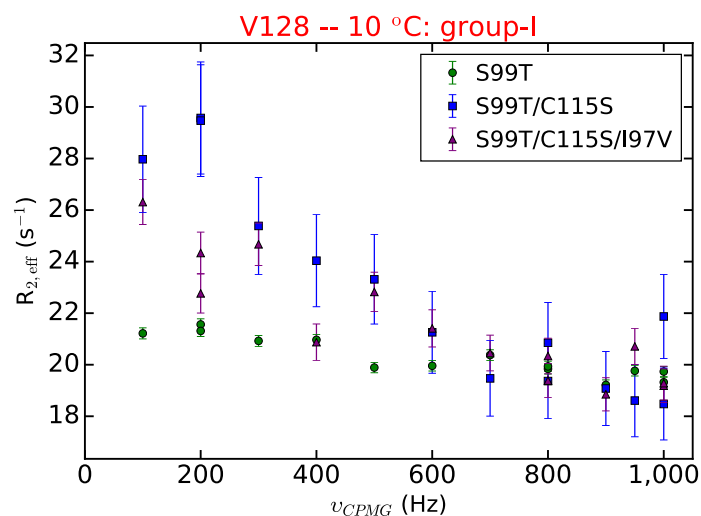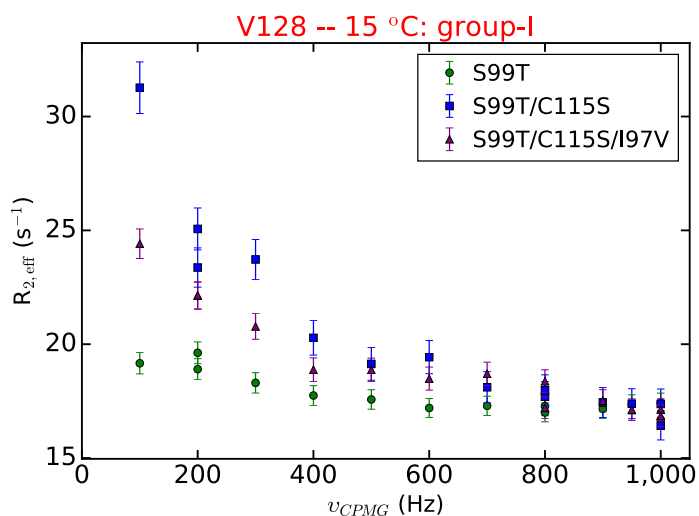

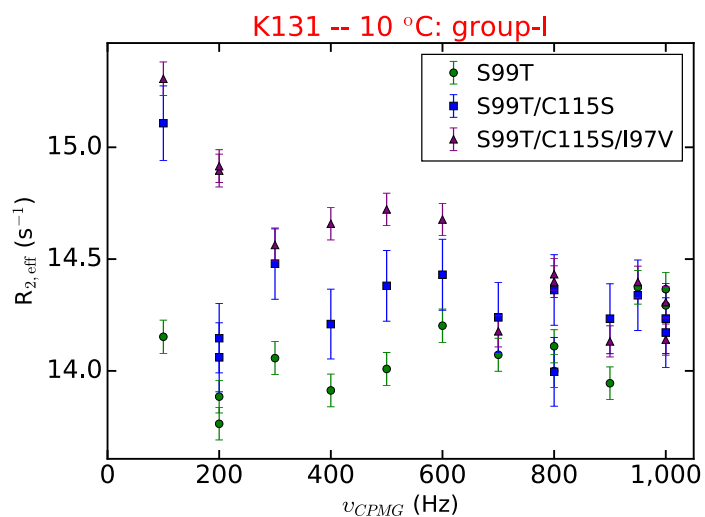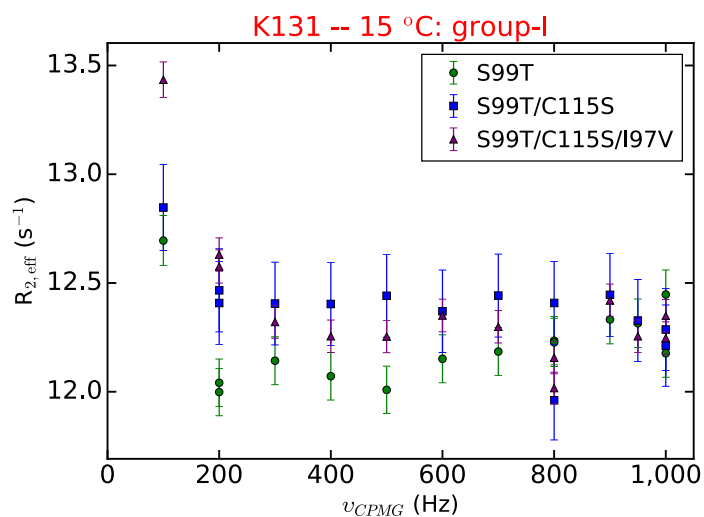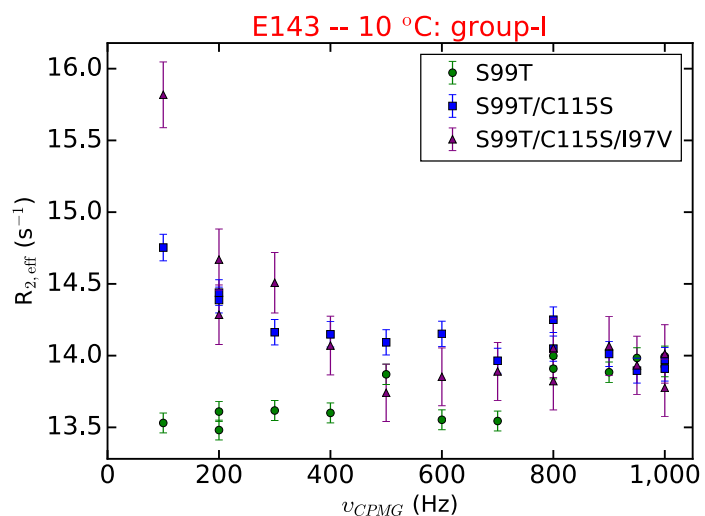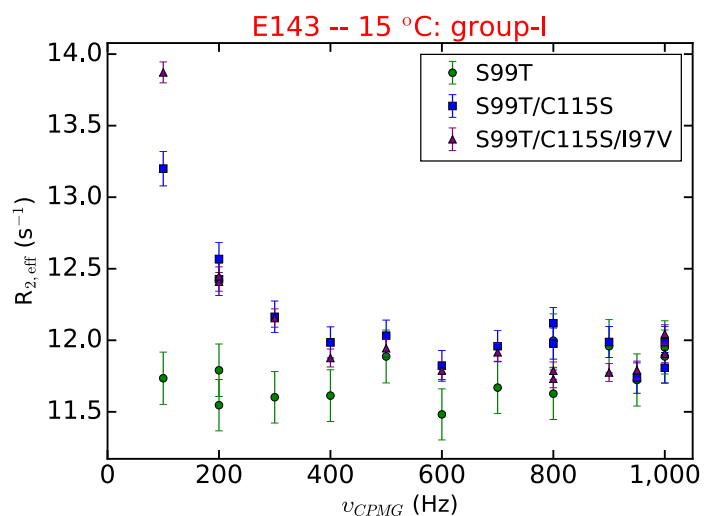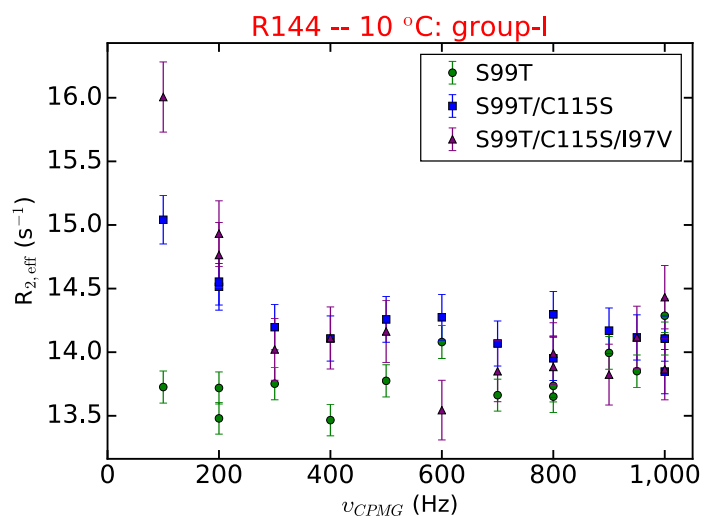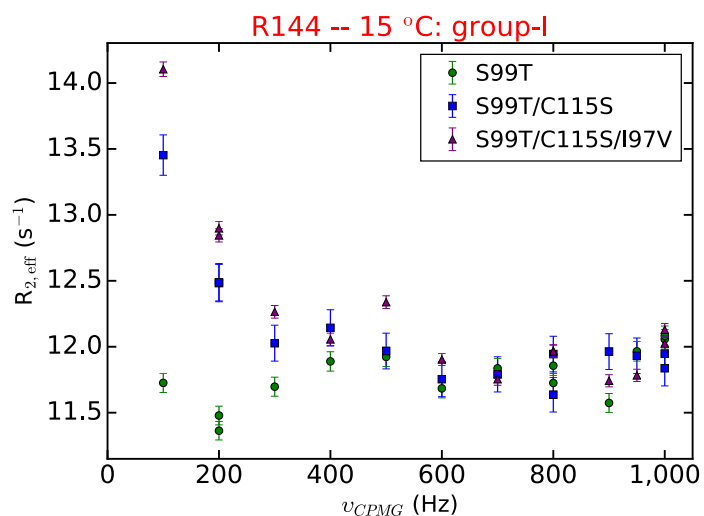

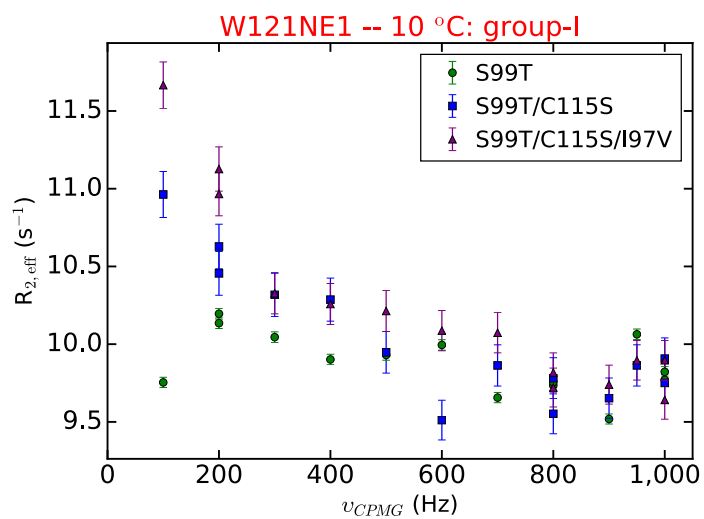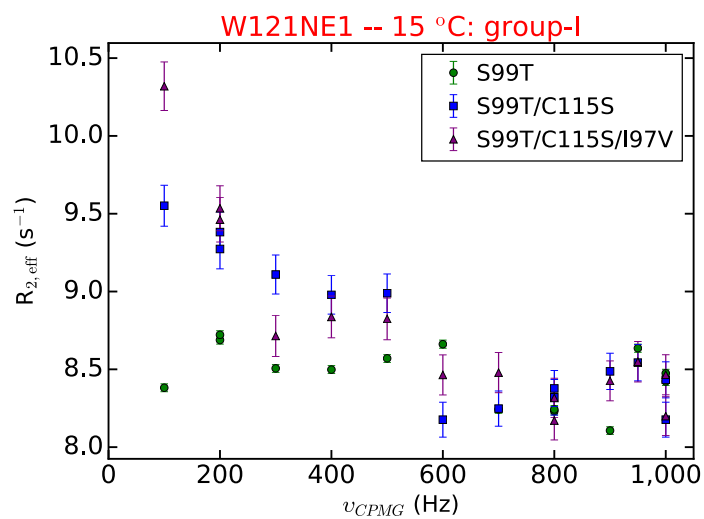

Supplement: Supplementary file 6 — Supplementary Data 3 [file 41467_2018_3562_MOESM6_ESM.pdf]

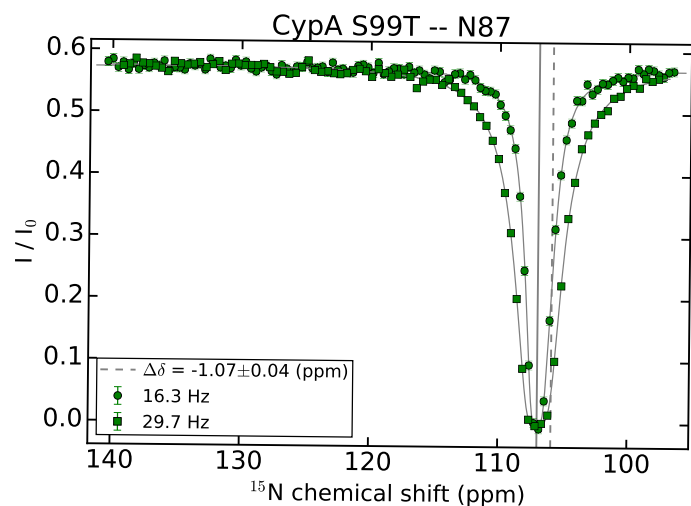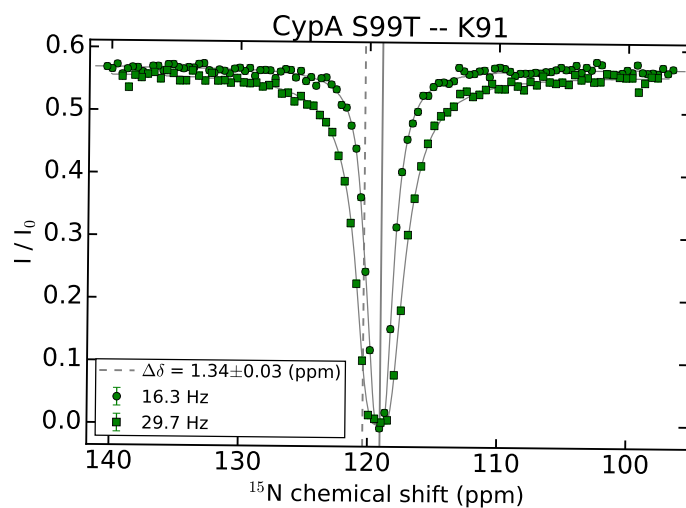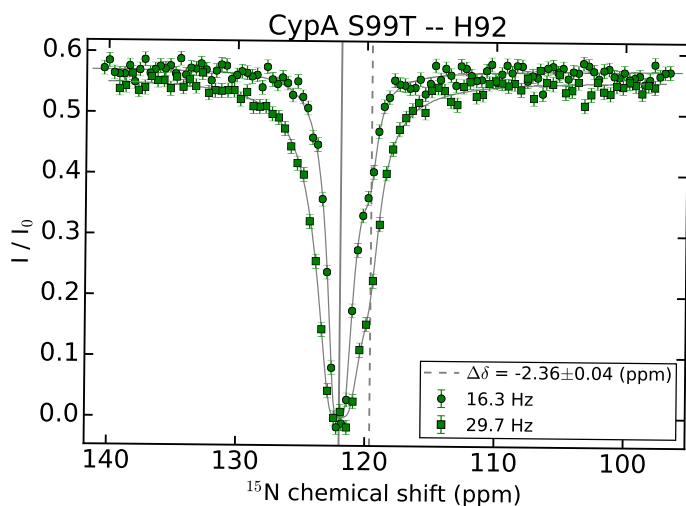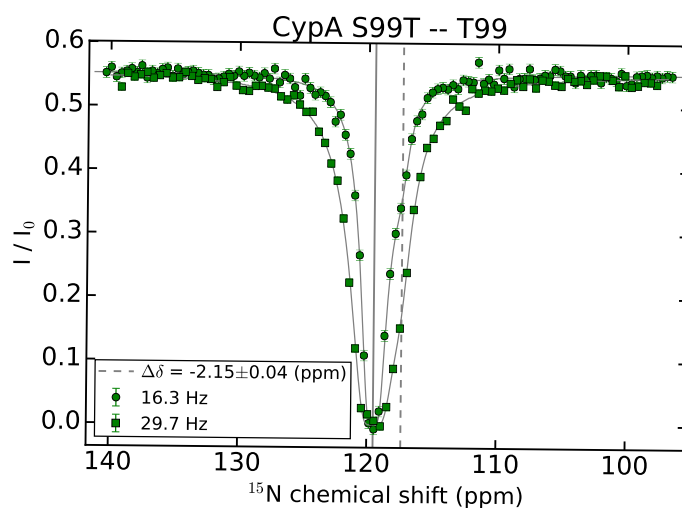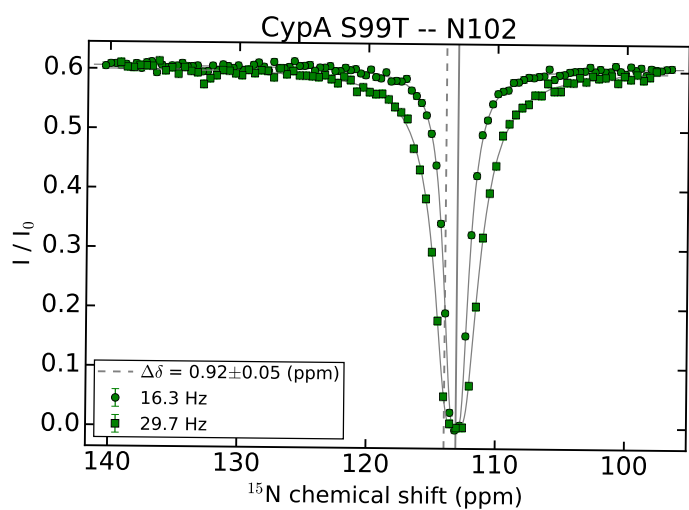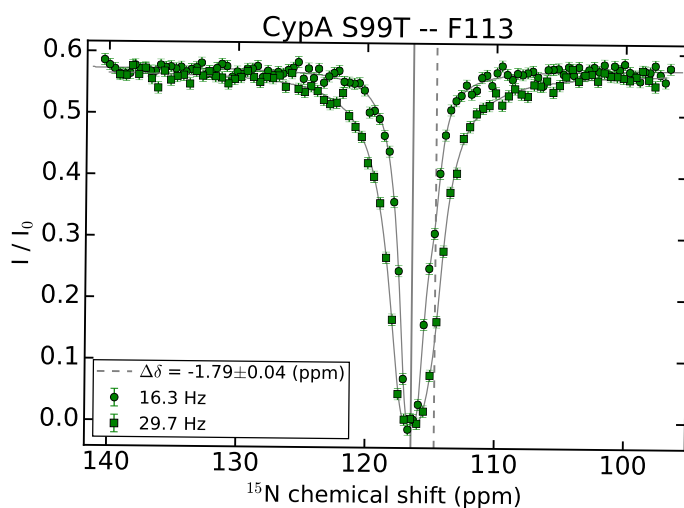

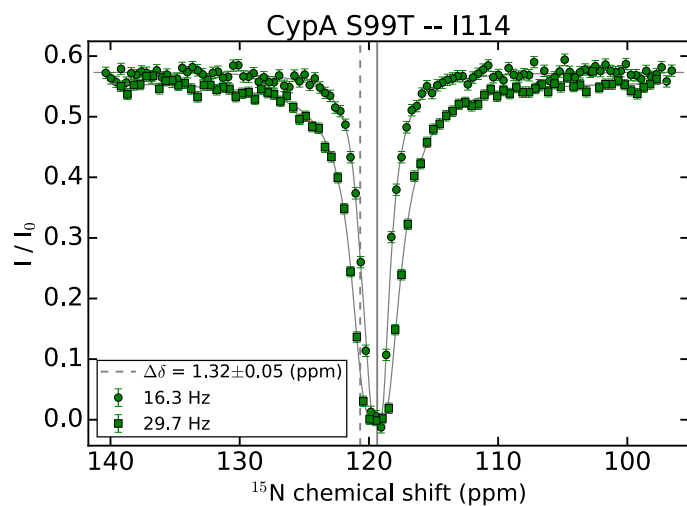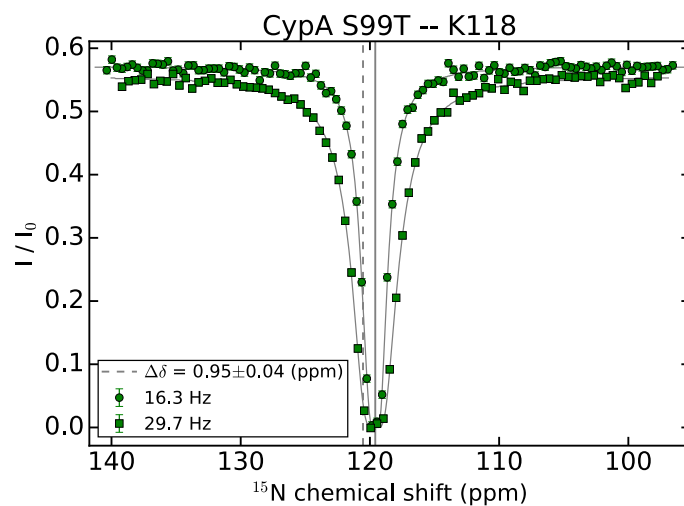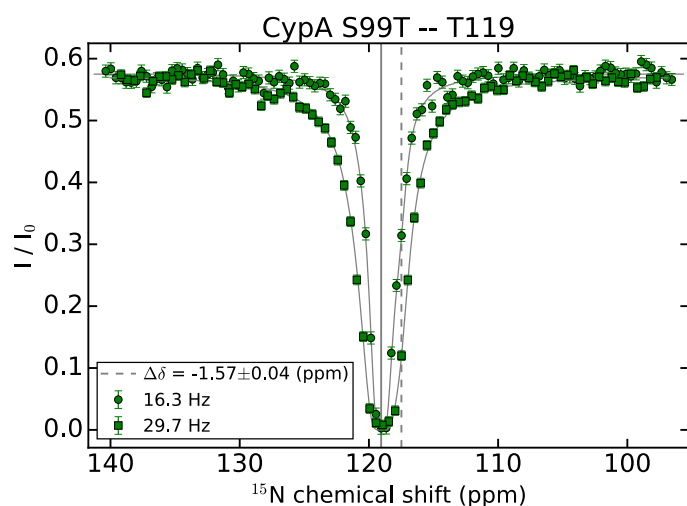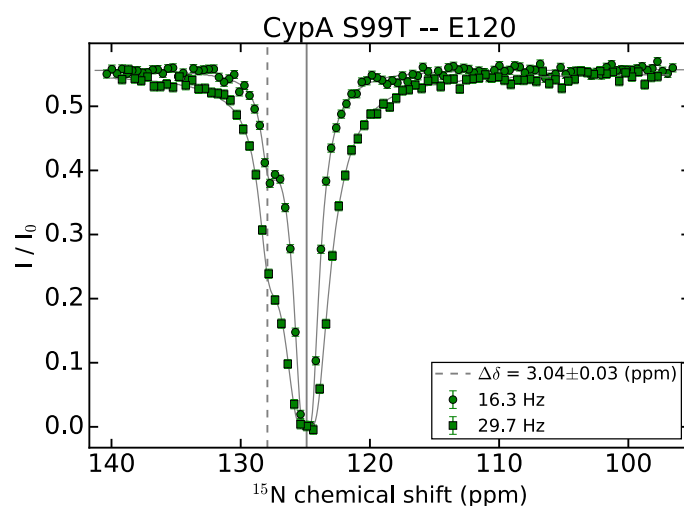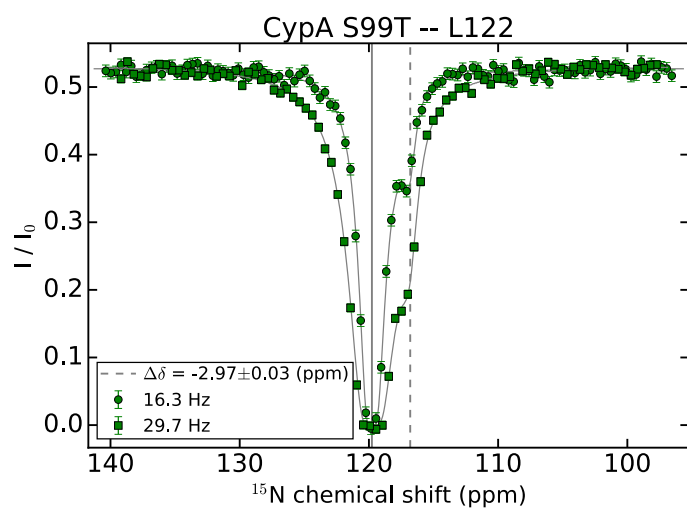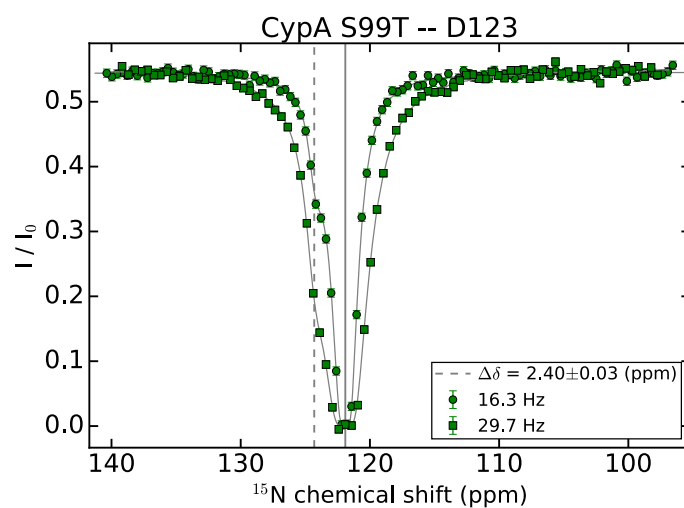

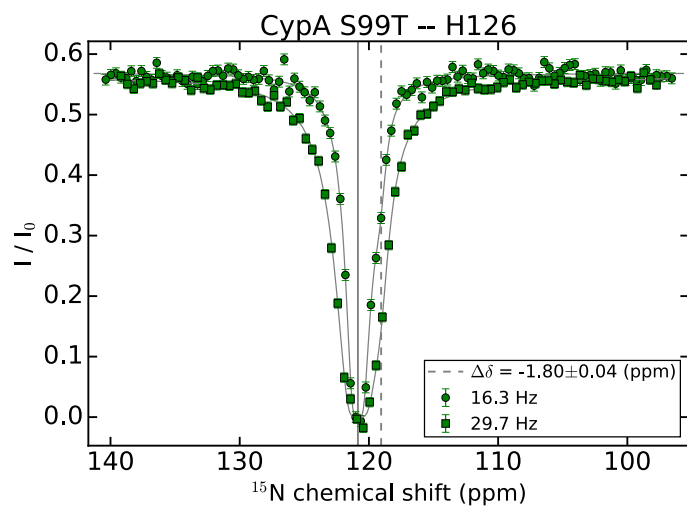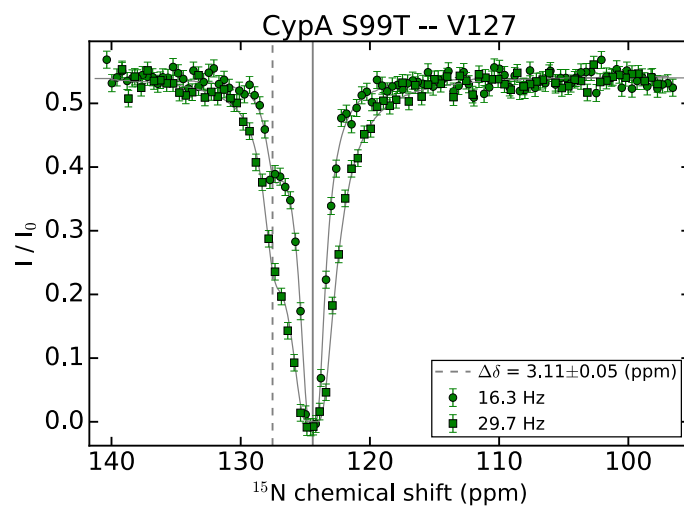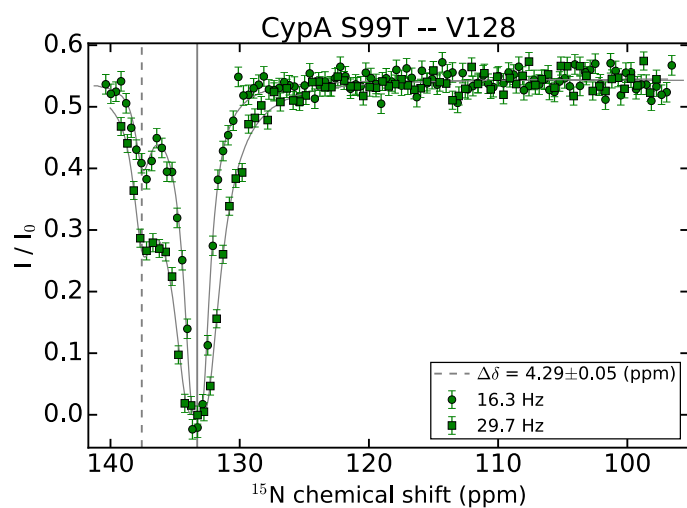

Supplement: Supplementary file 7 — Supplementary Data 4 [file 41467_2018_3562_MOESM7_ESM.pdf]

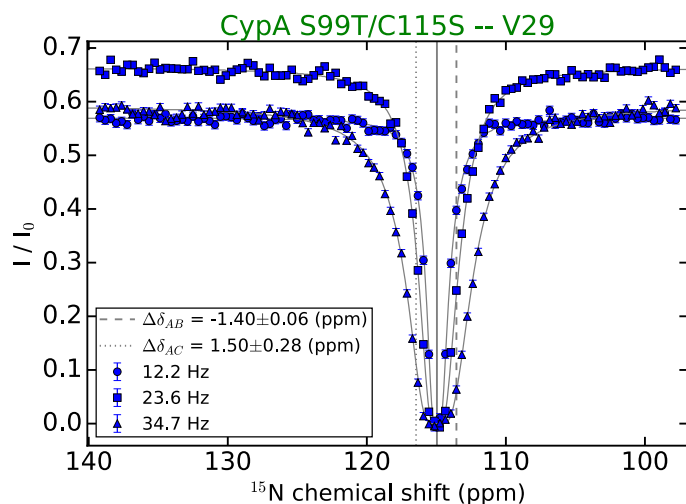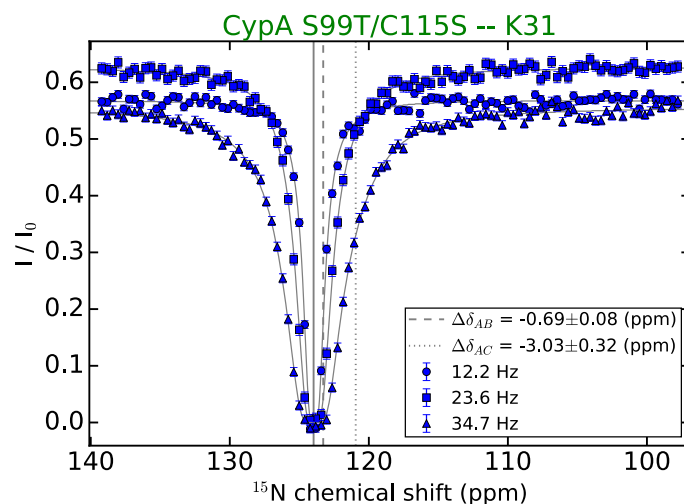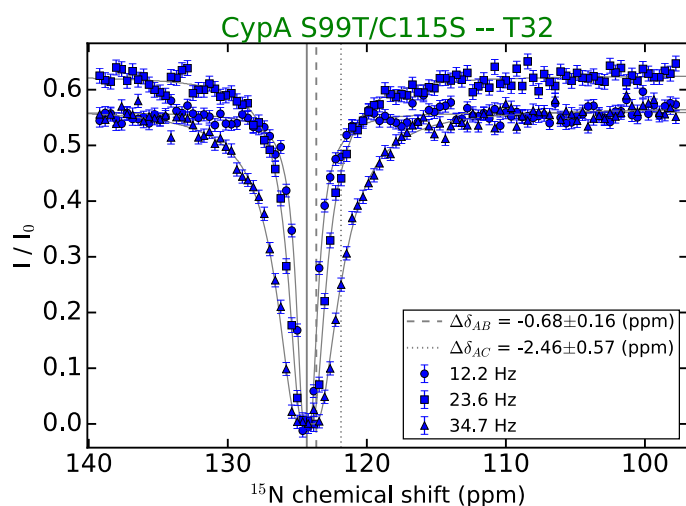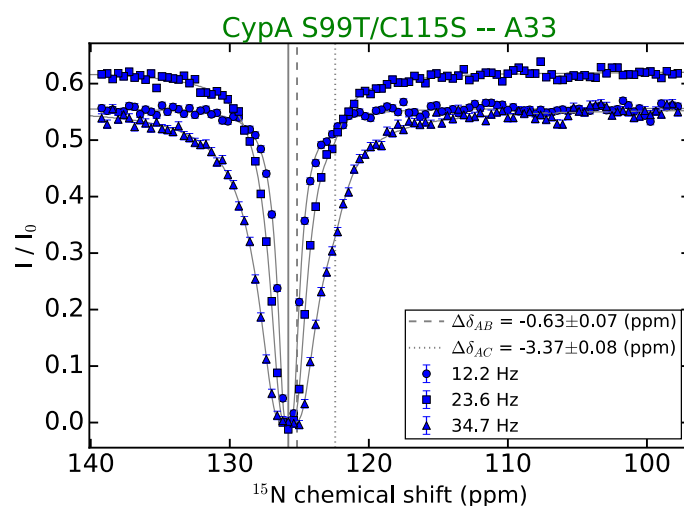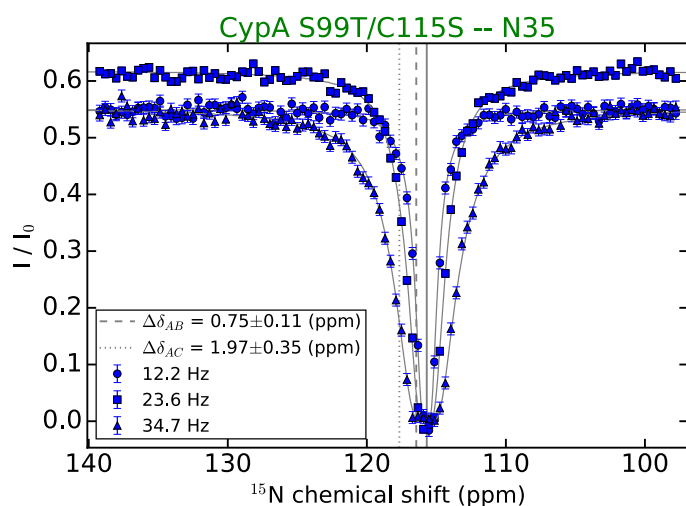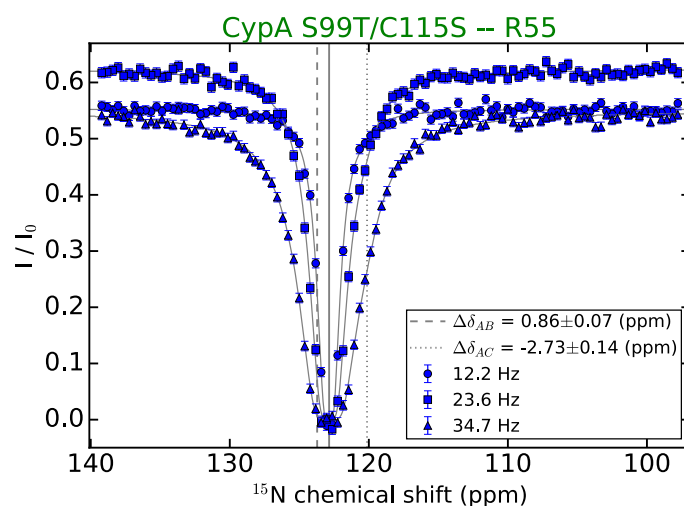

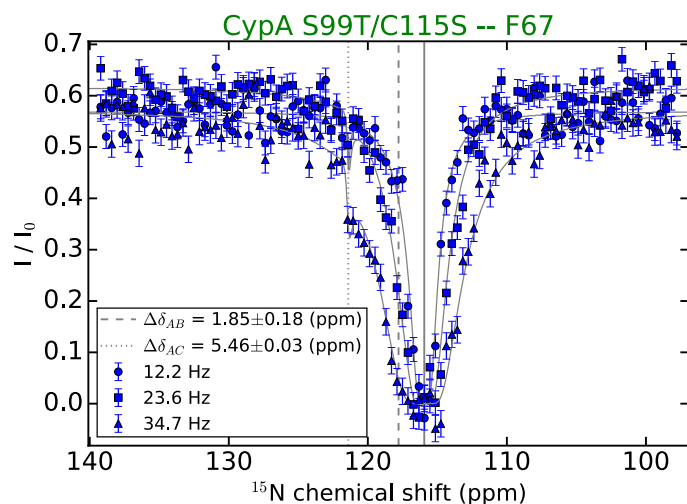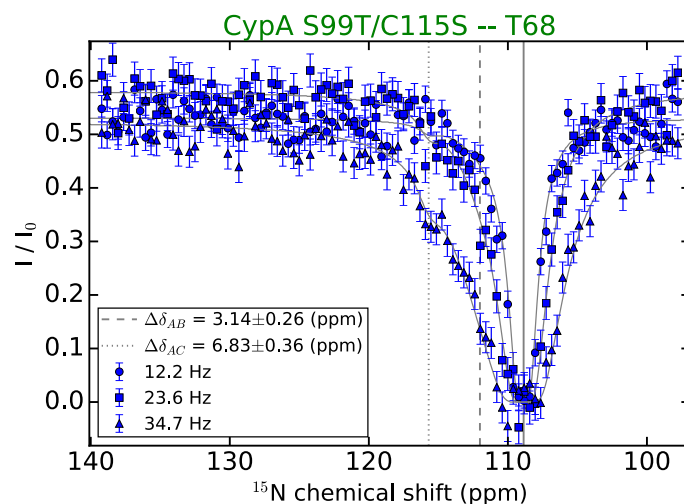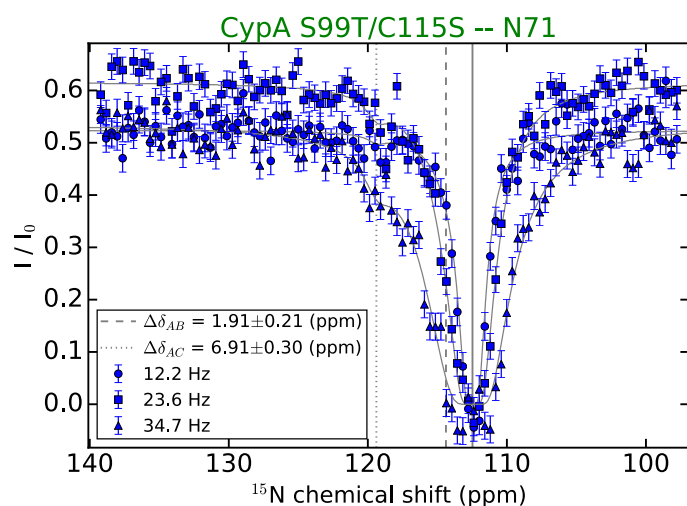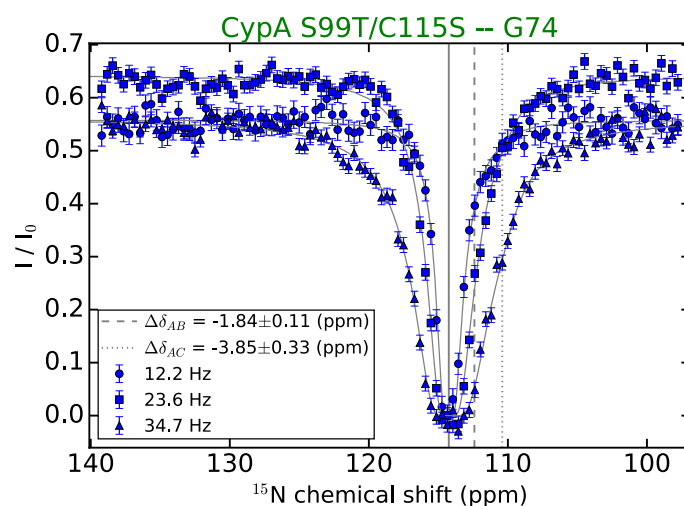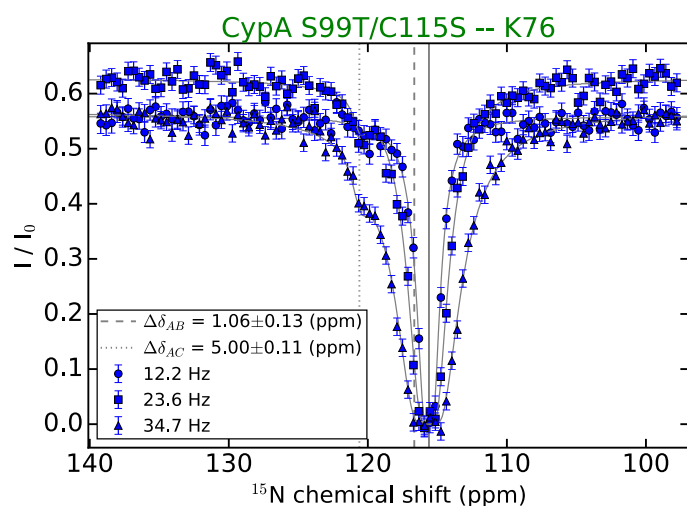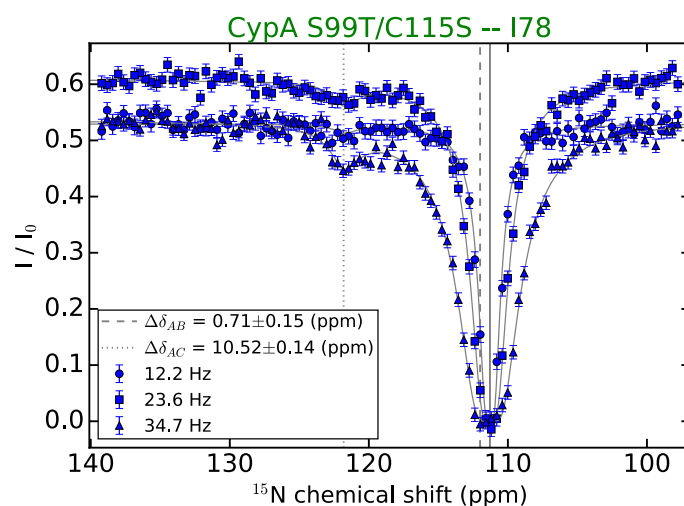

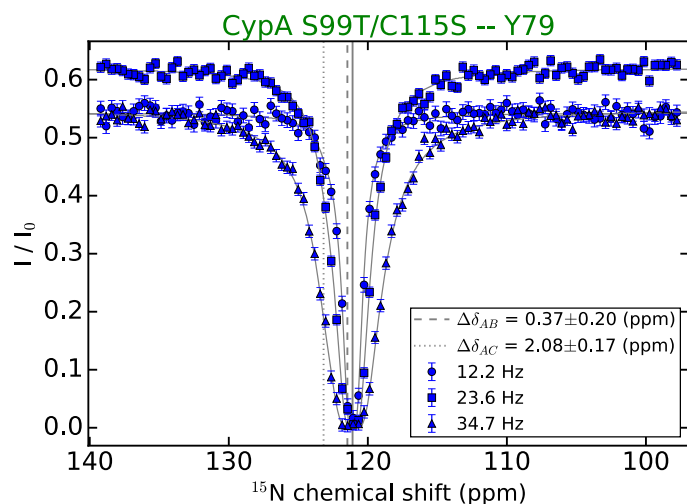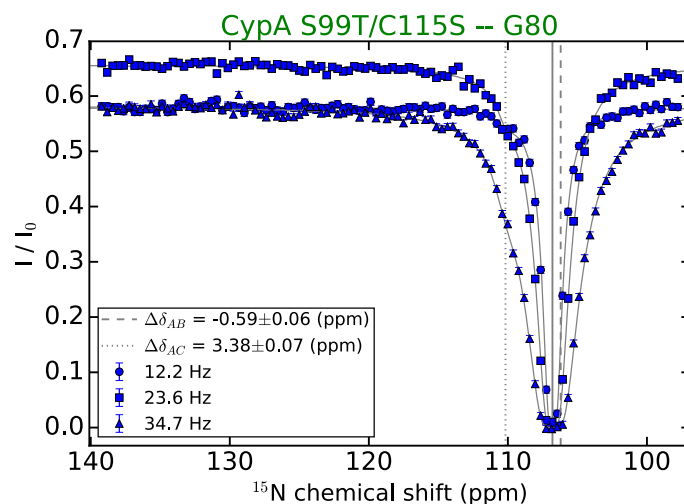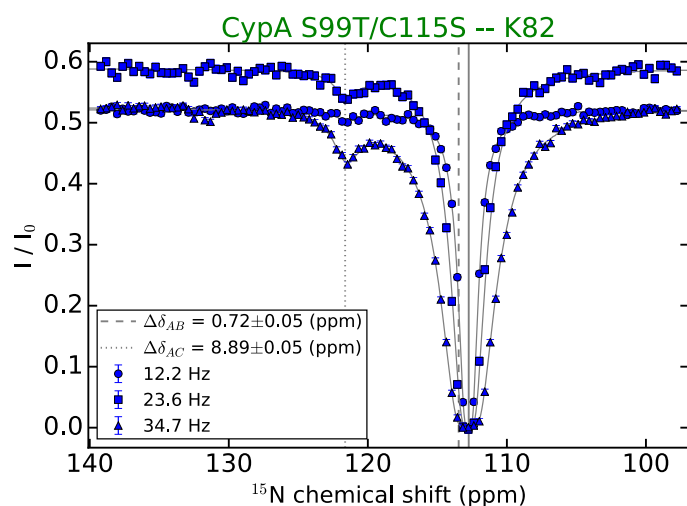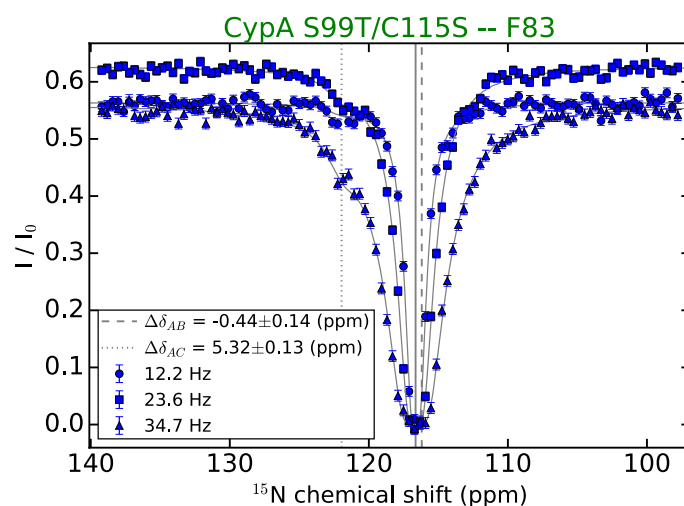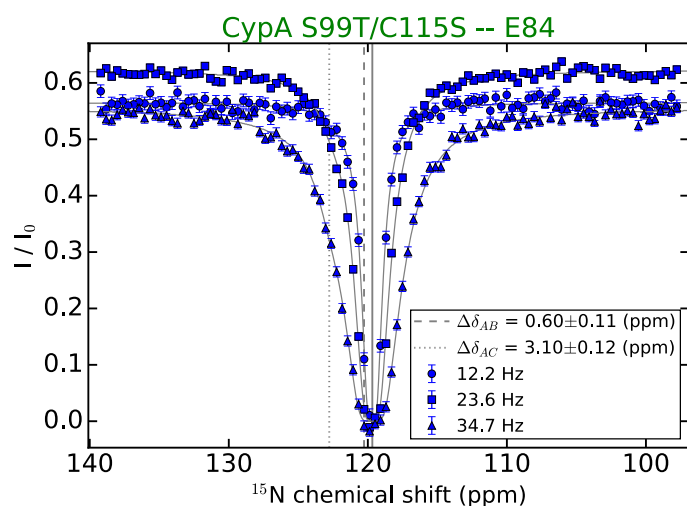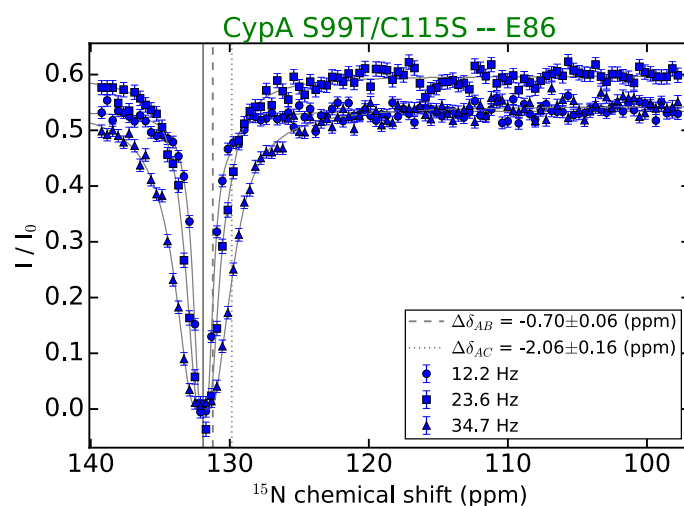

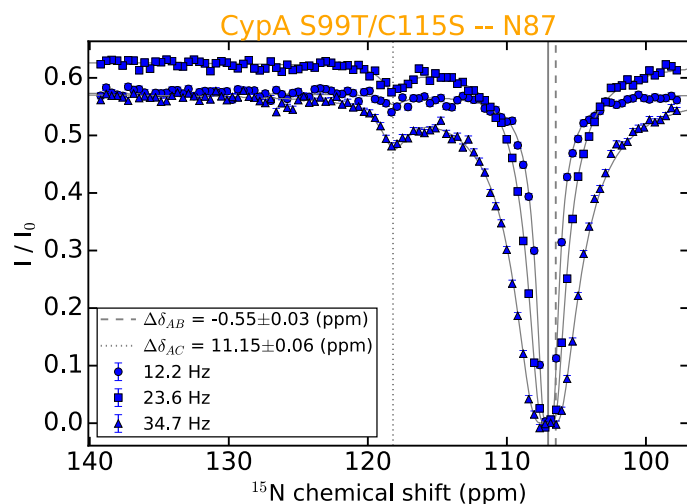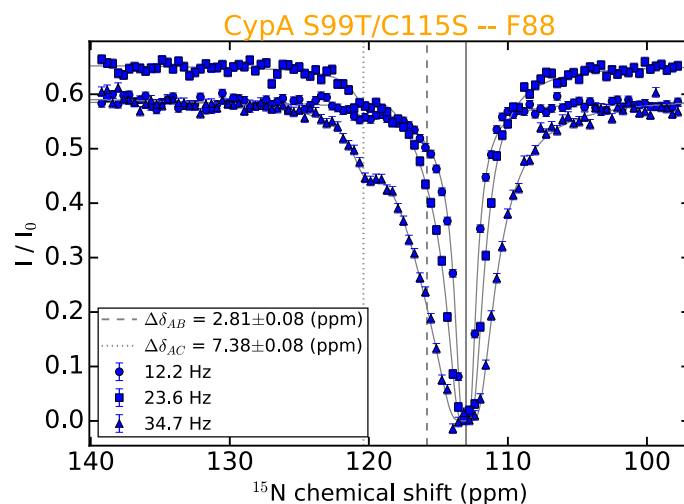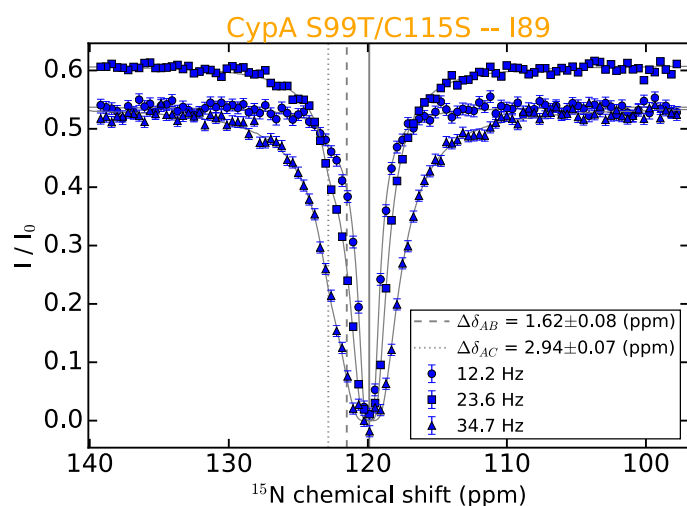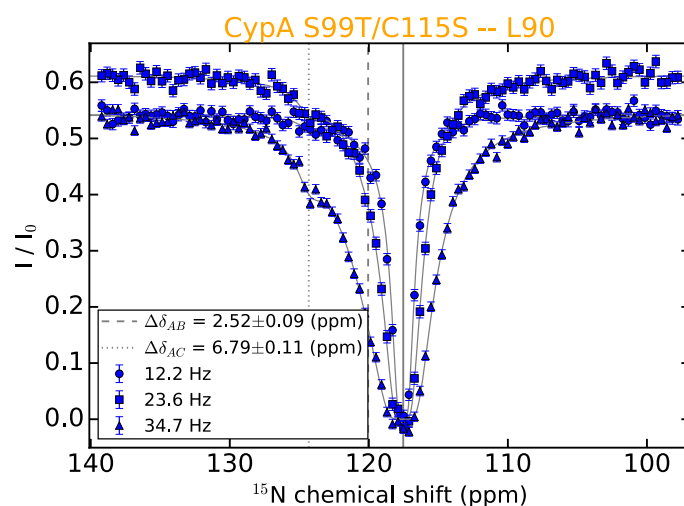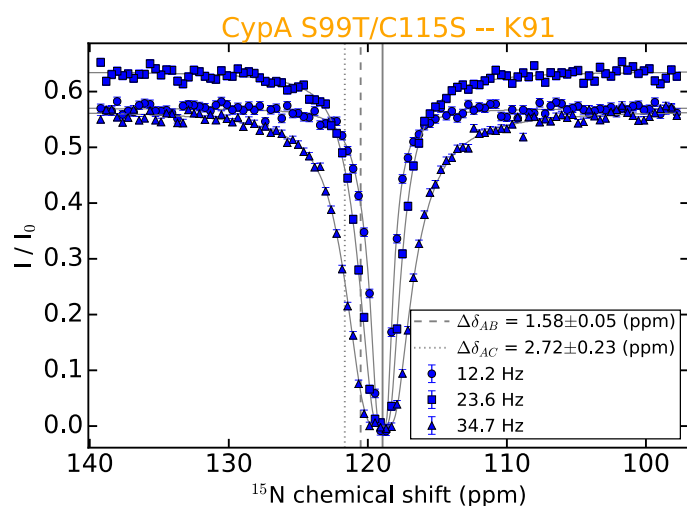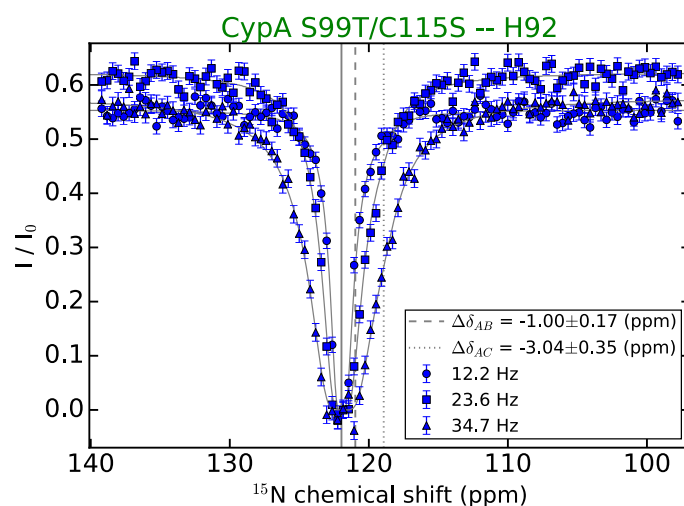

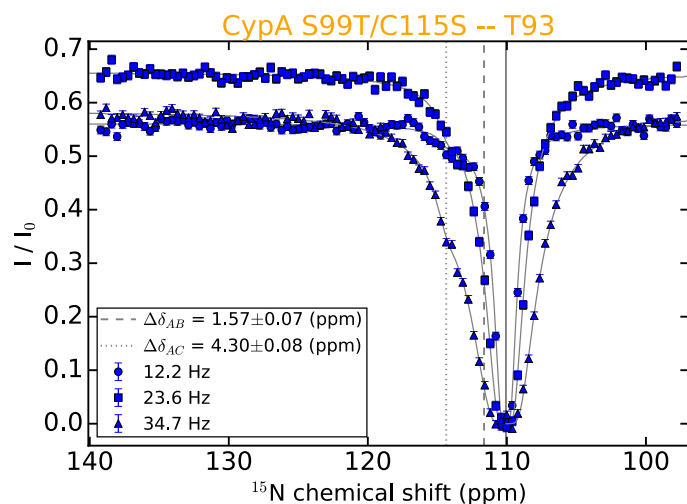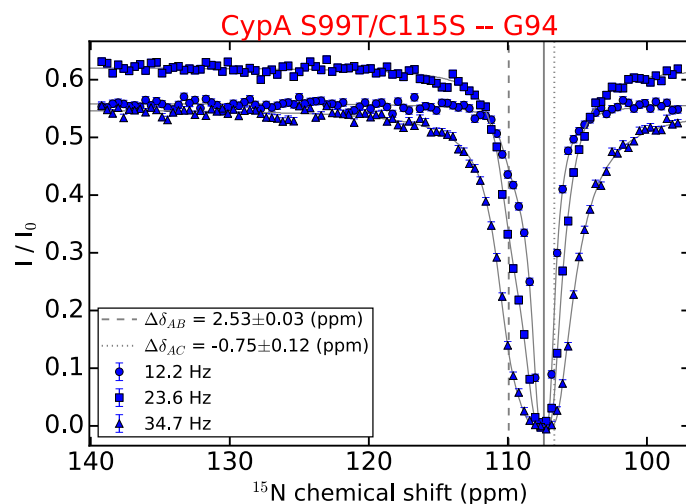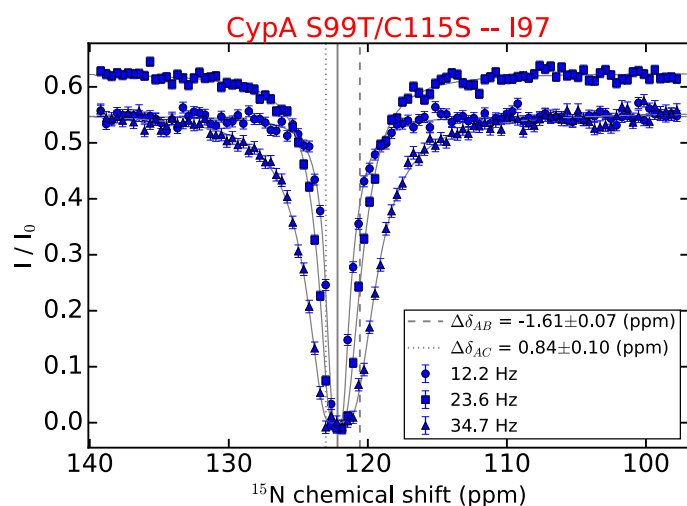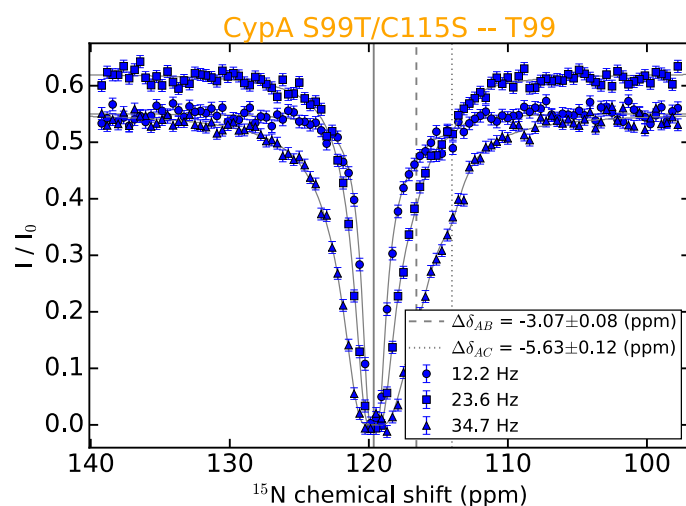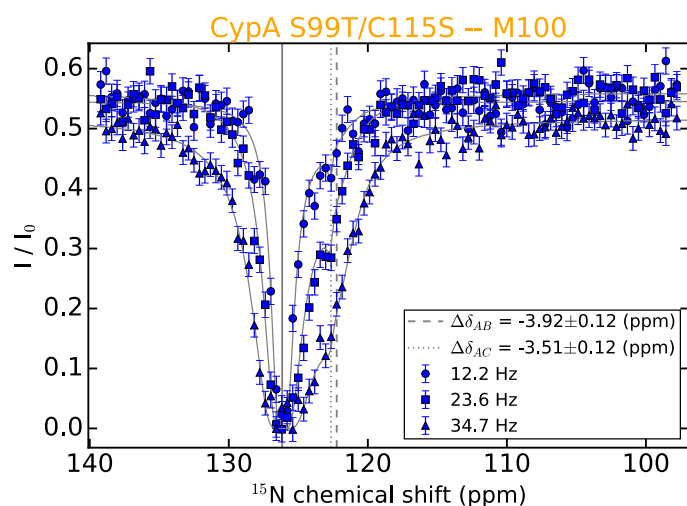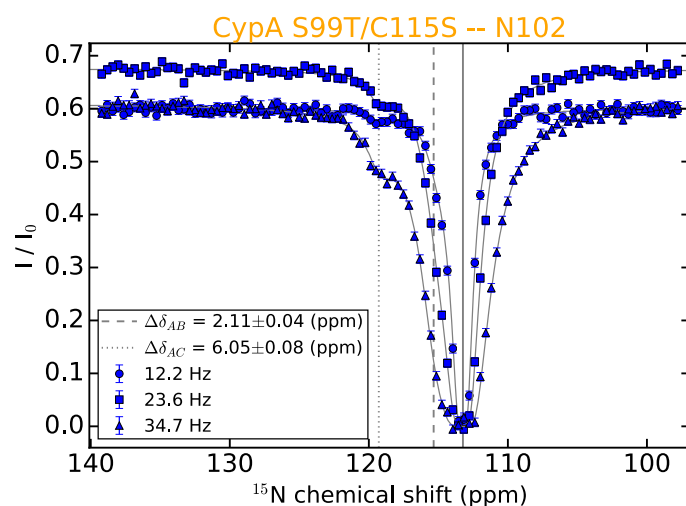

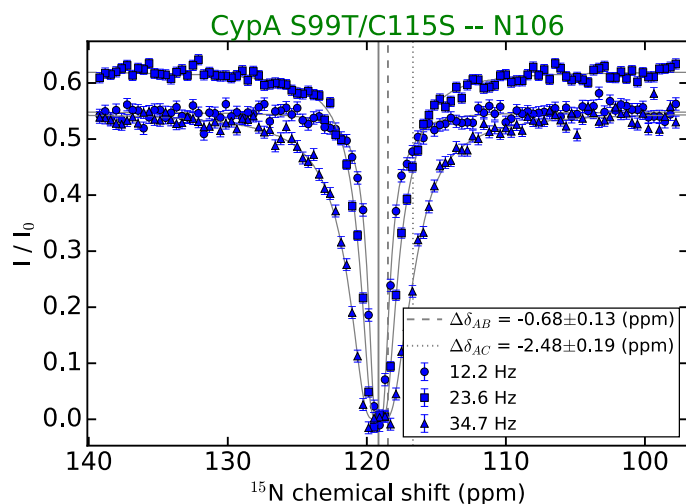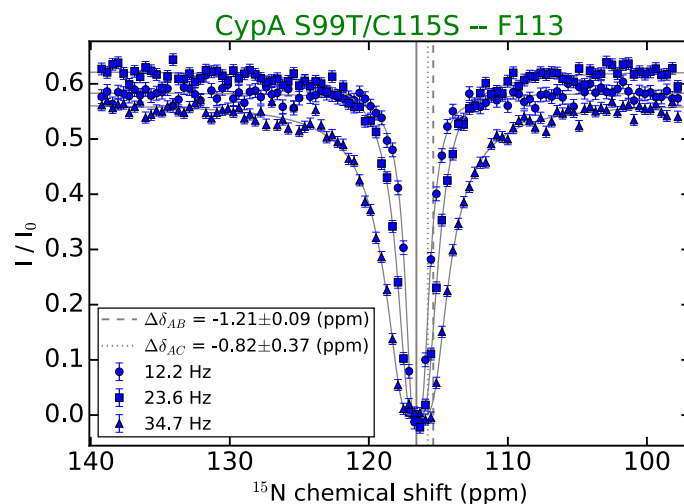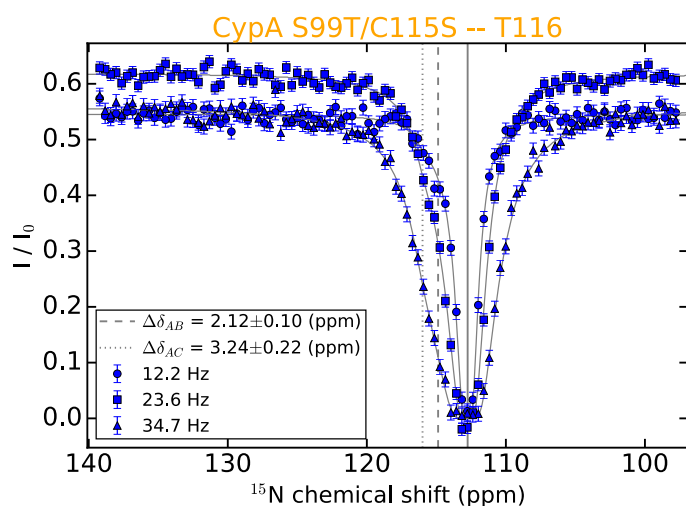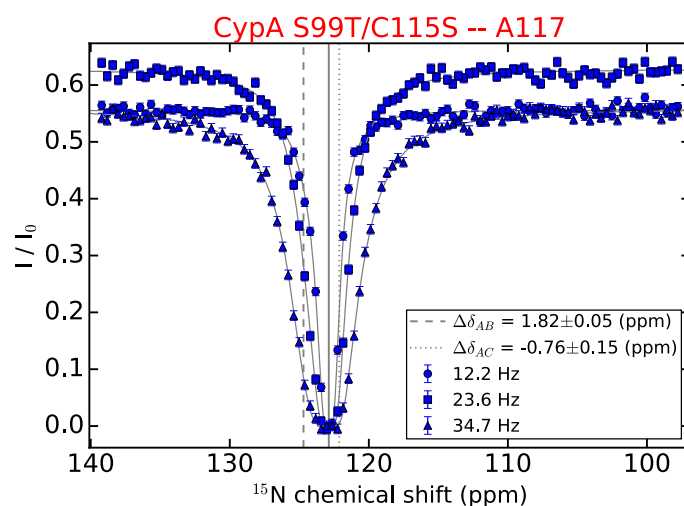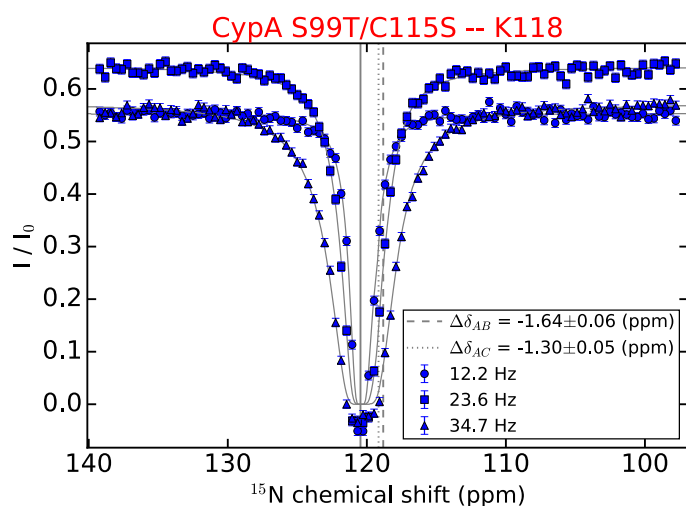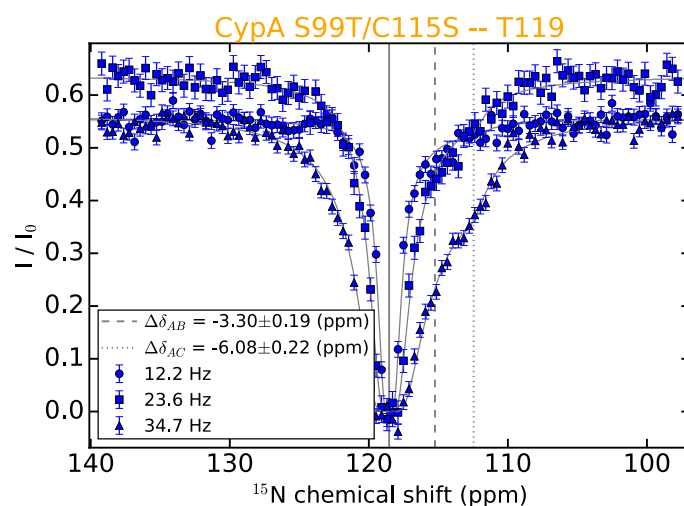

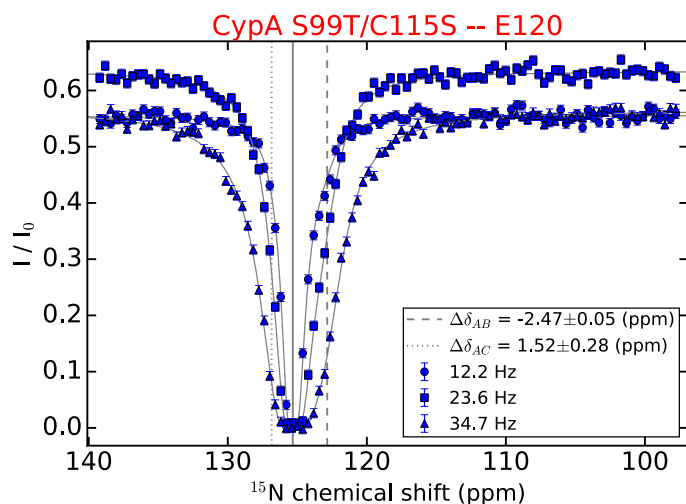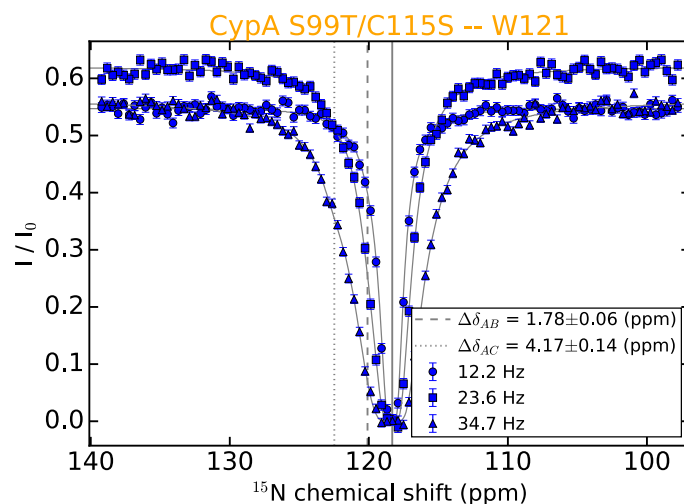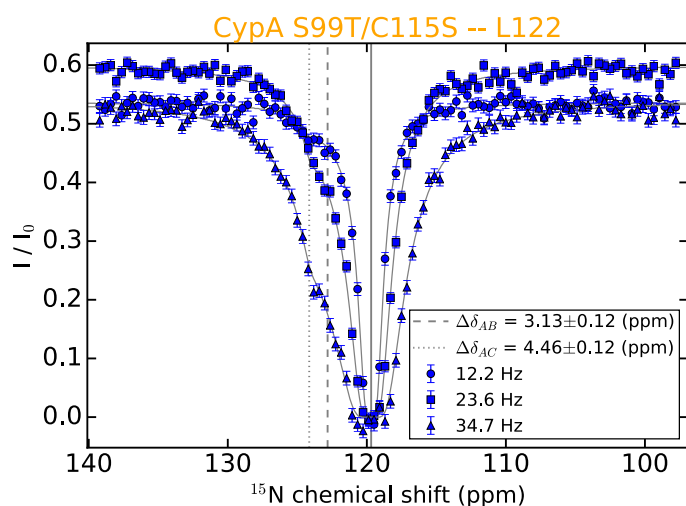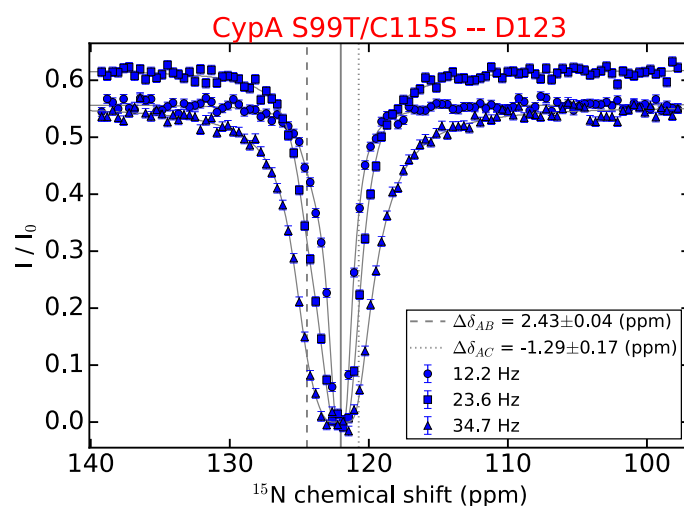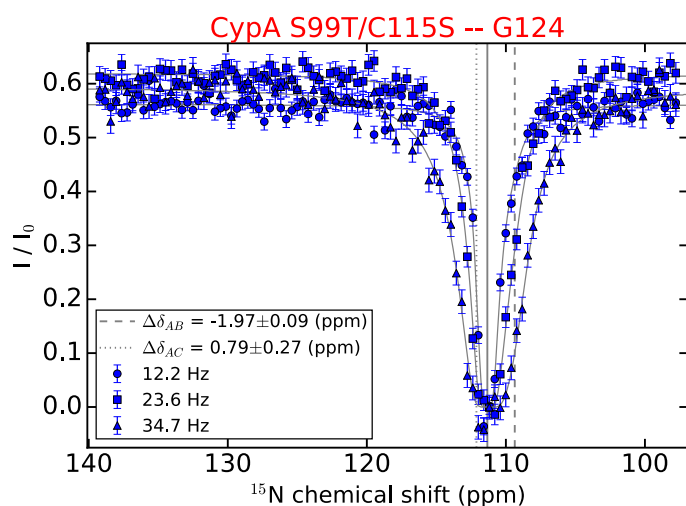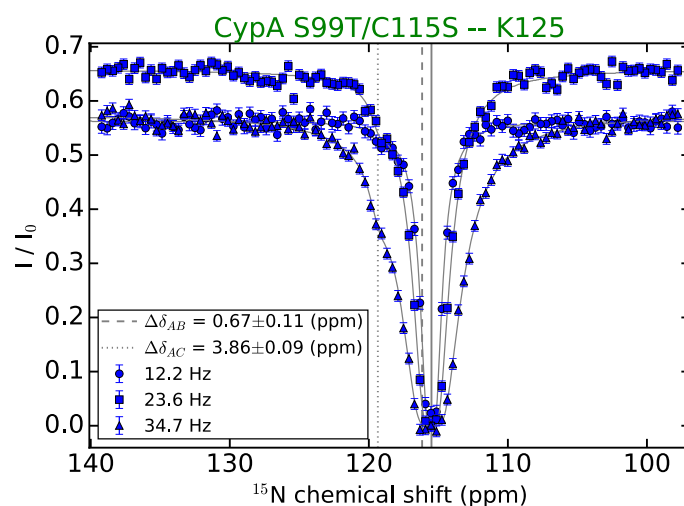

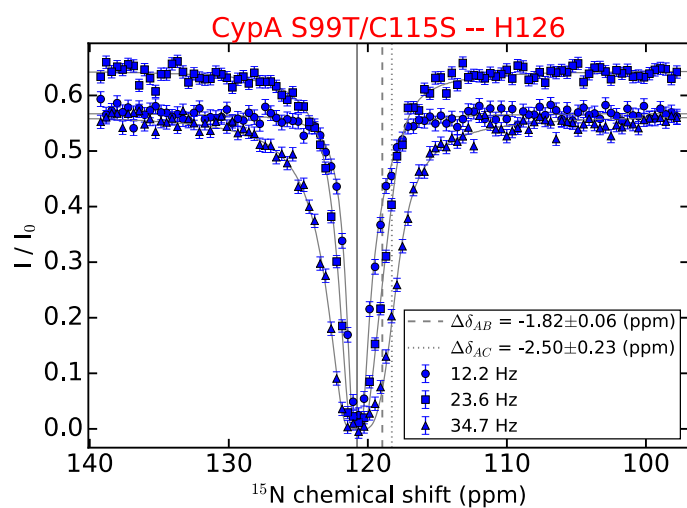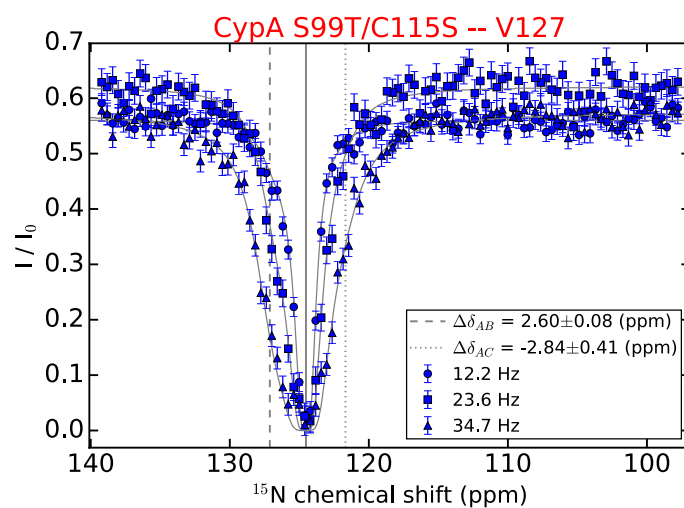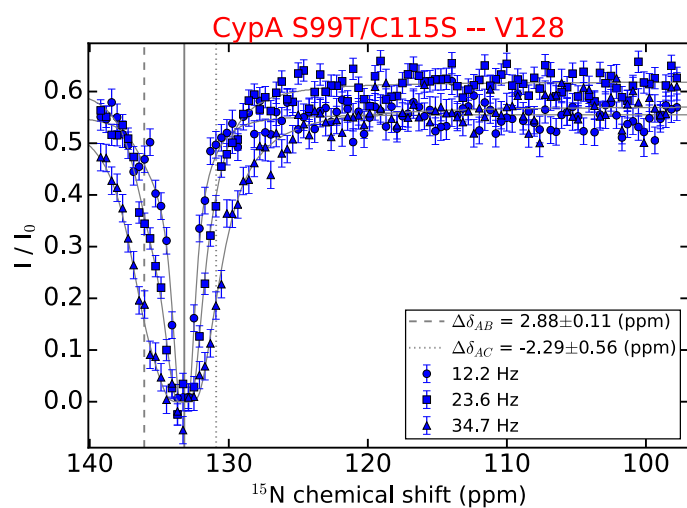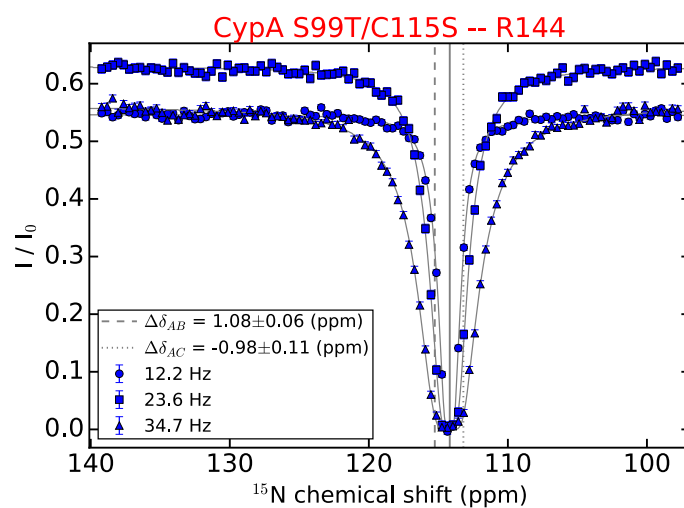

Supplement: Supplementary file 8 — Supplementary Data 5 [file 41467_2018_3562_MOESM8_ESM.pdf]

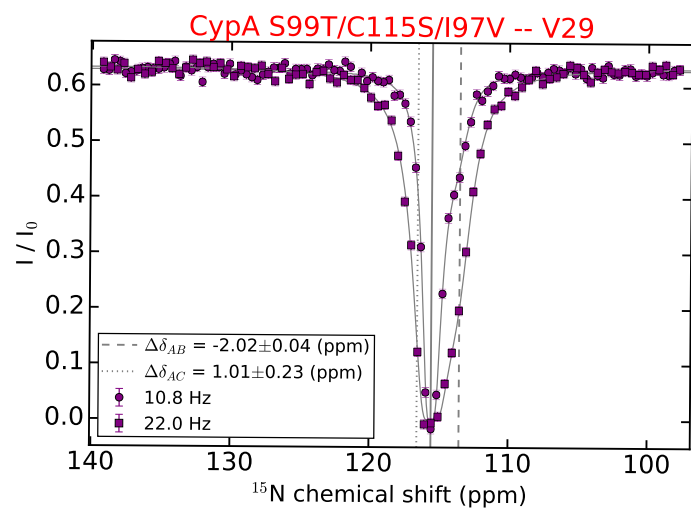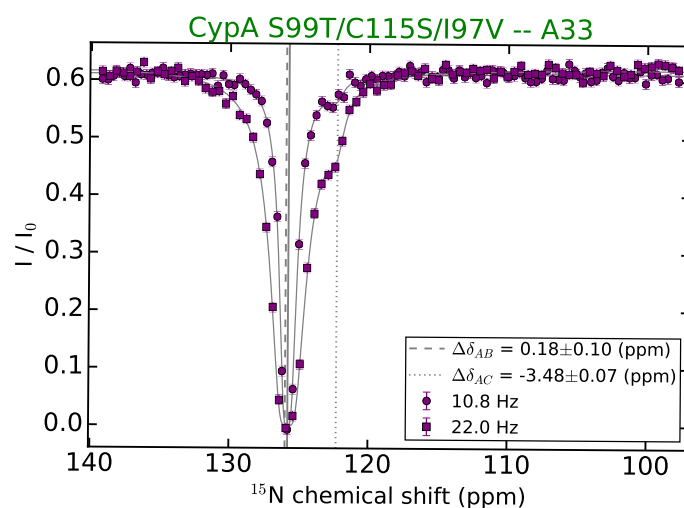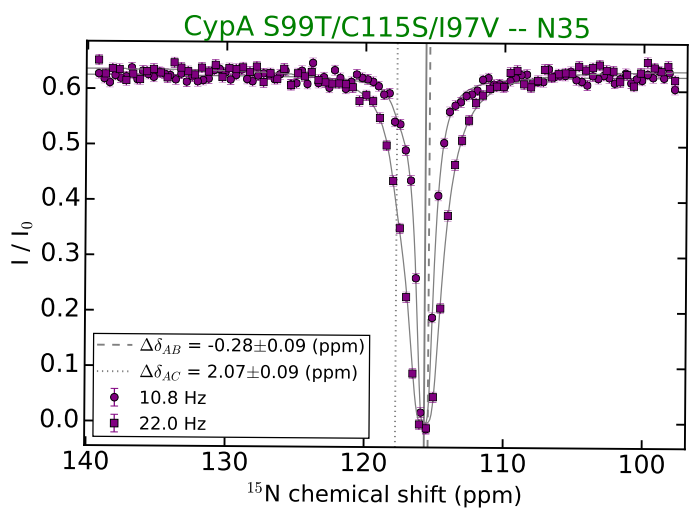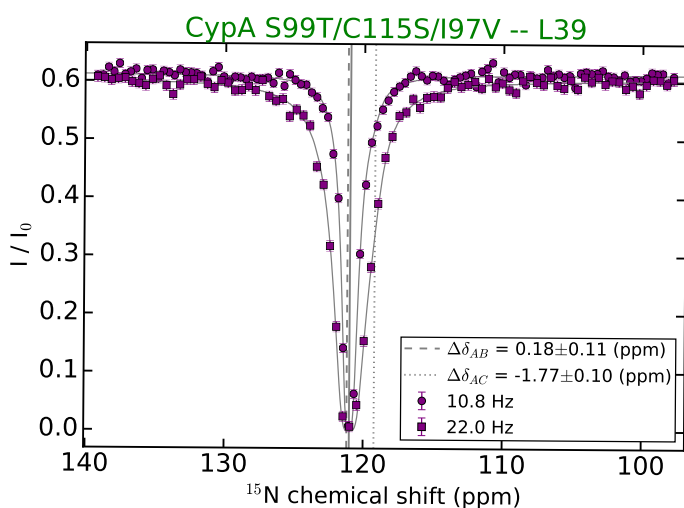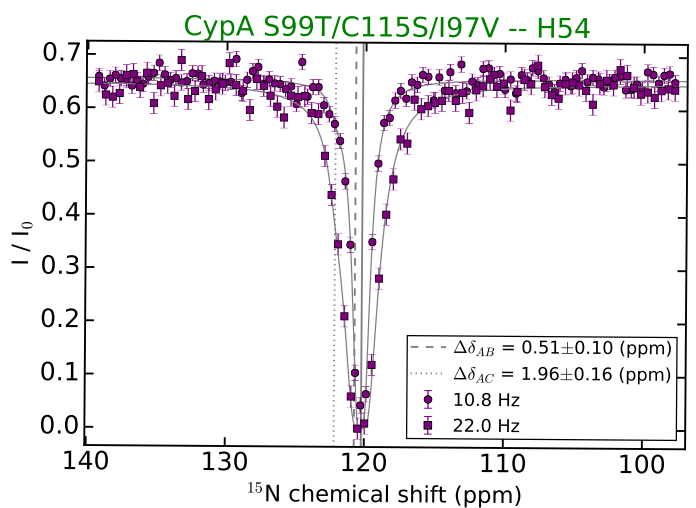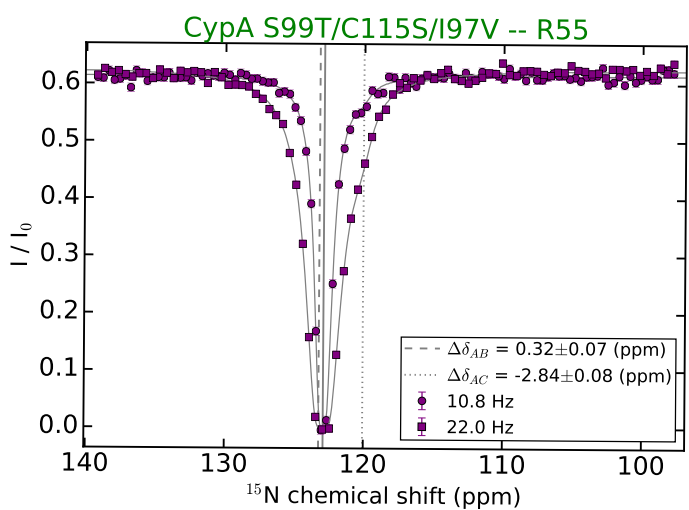

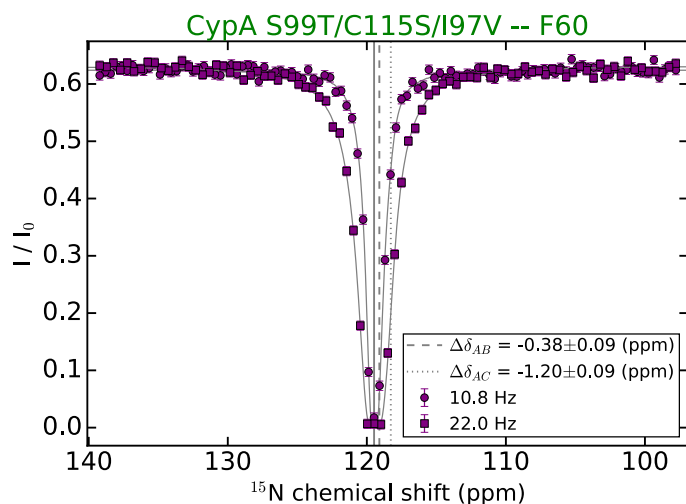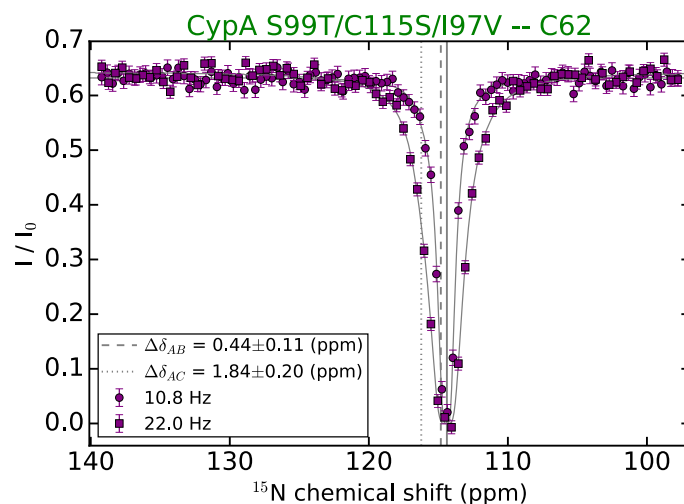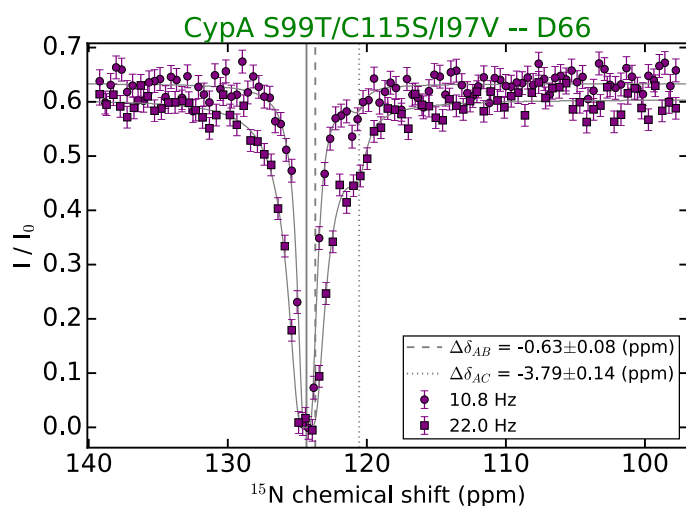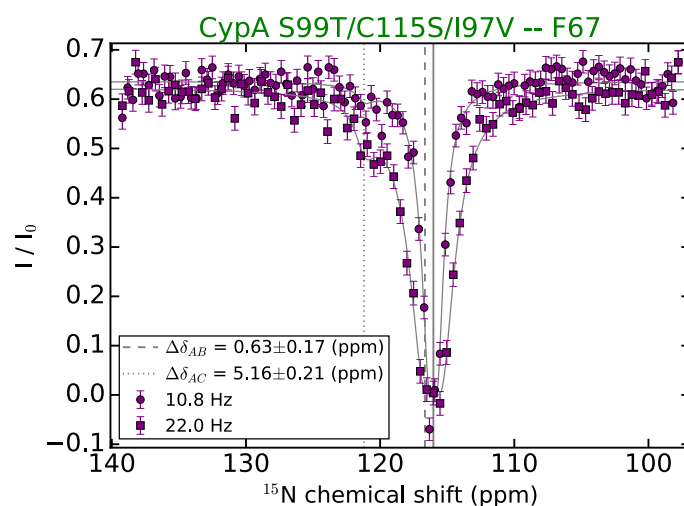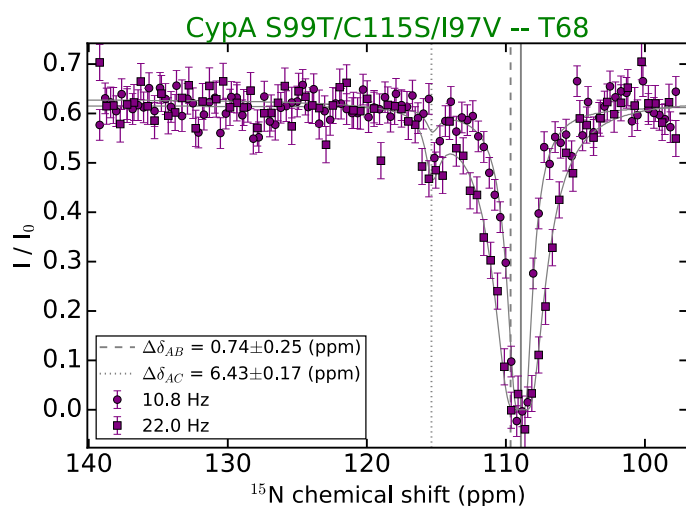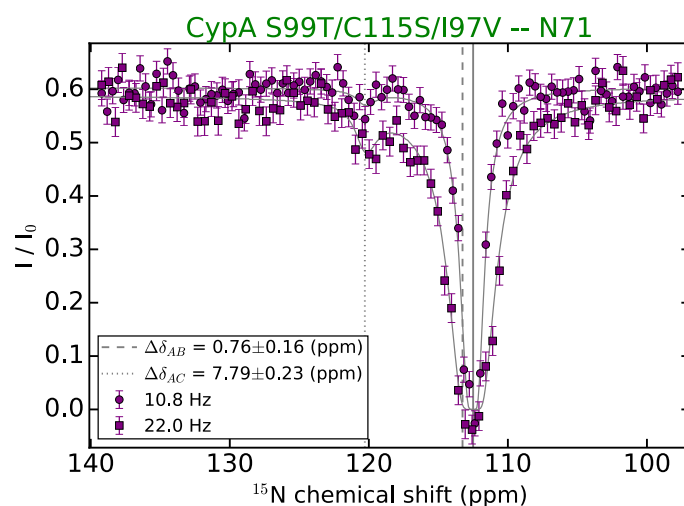

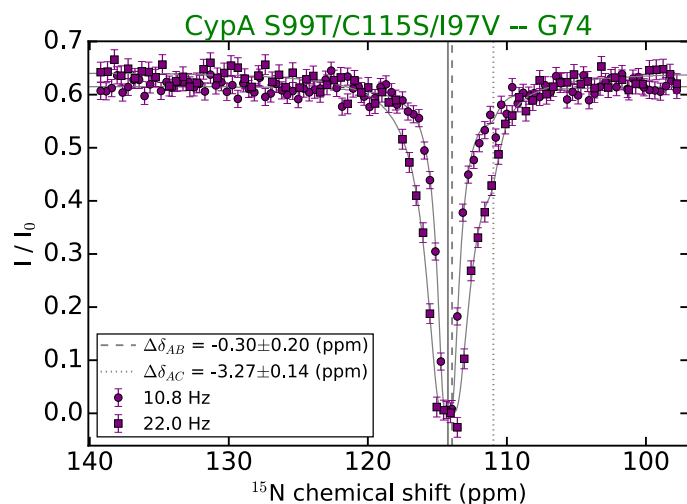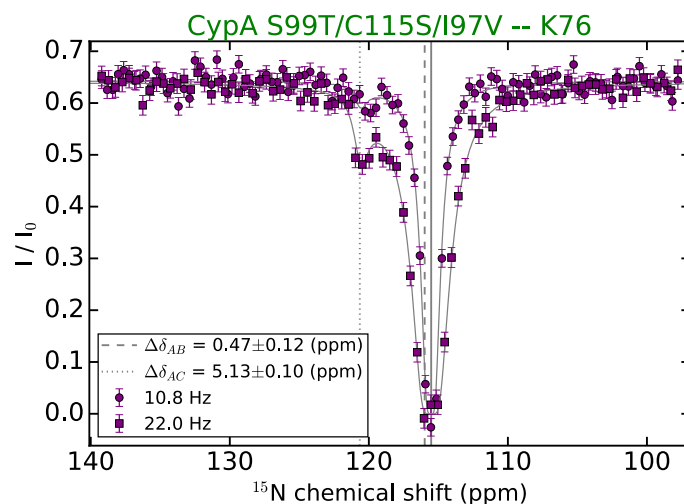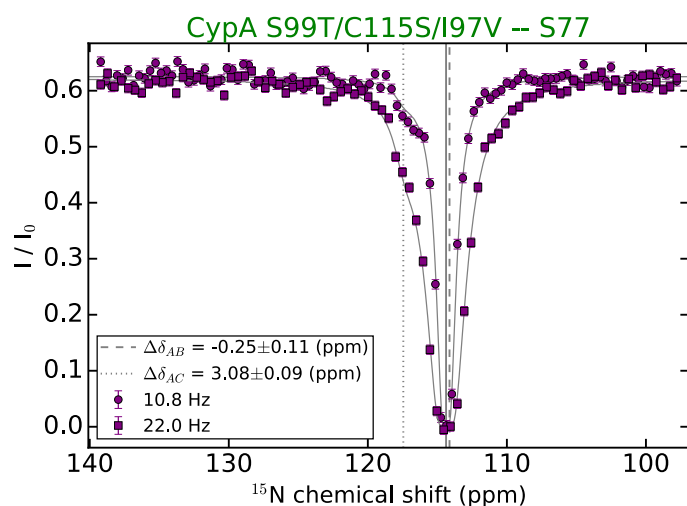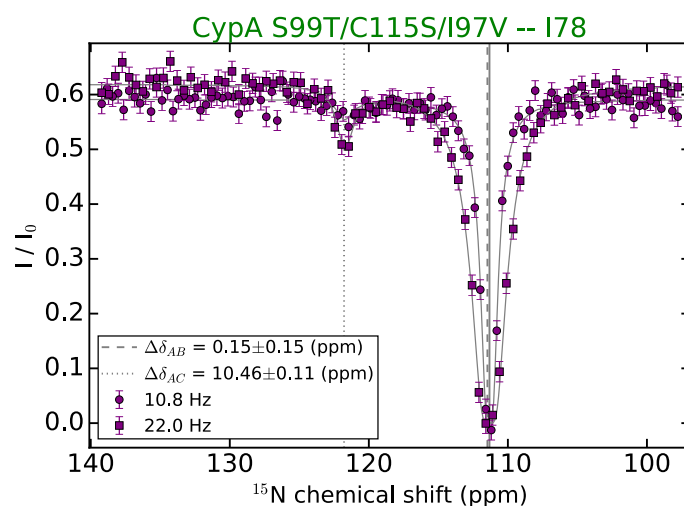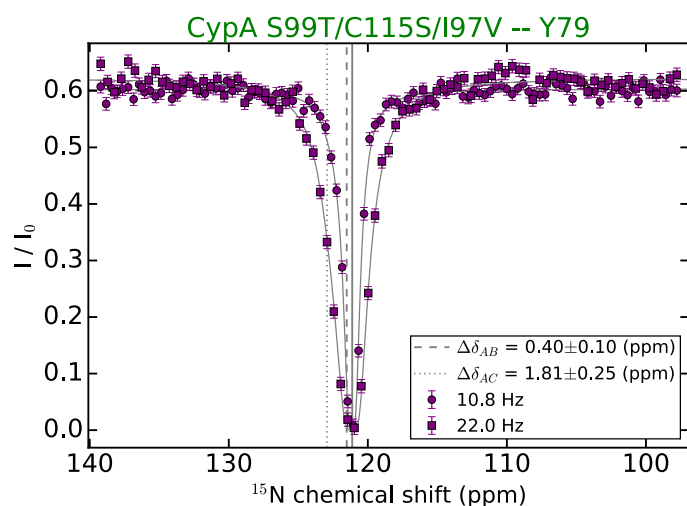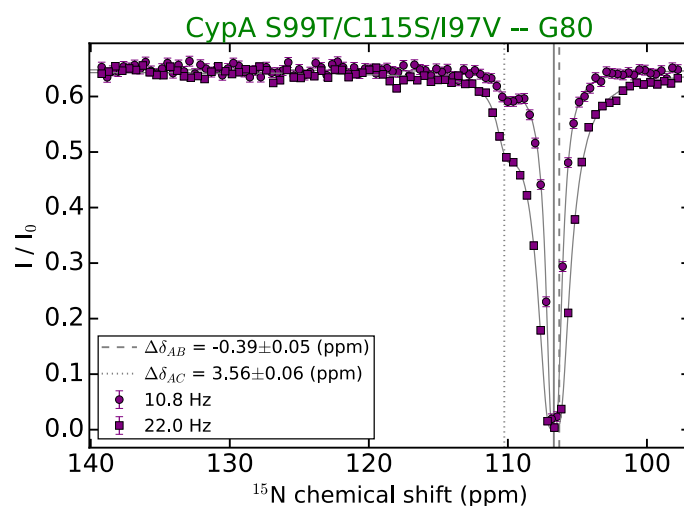

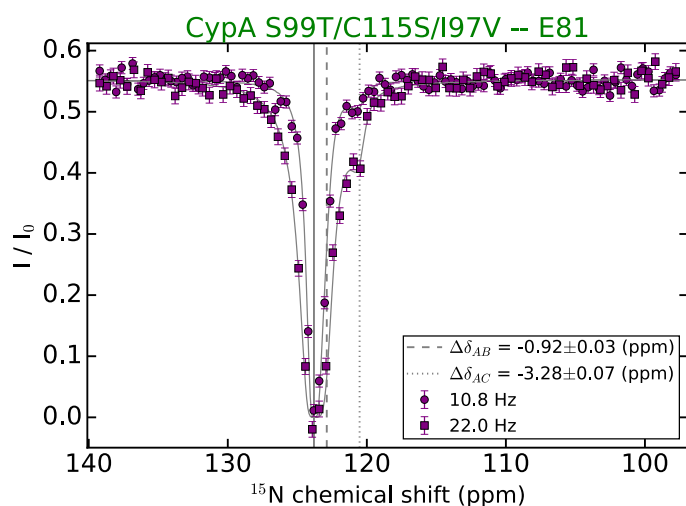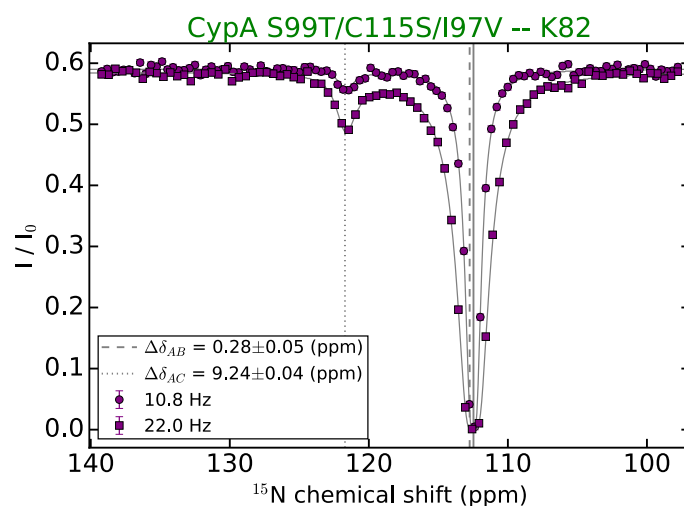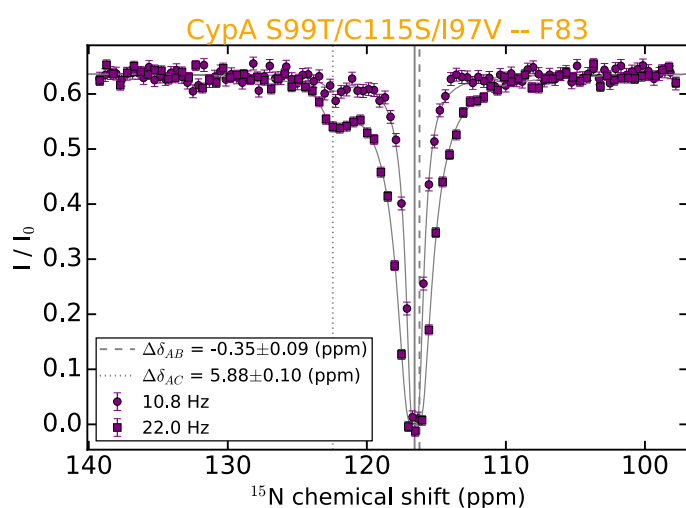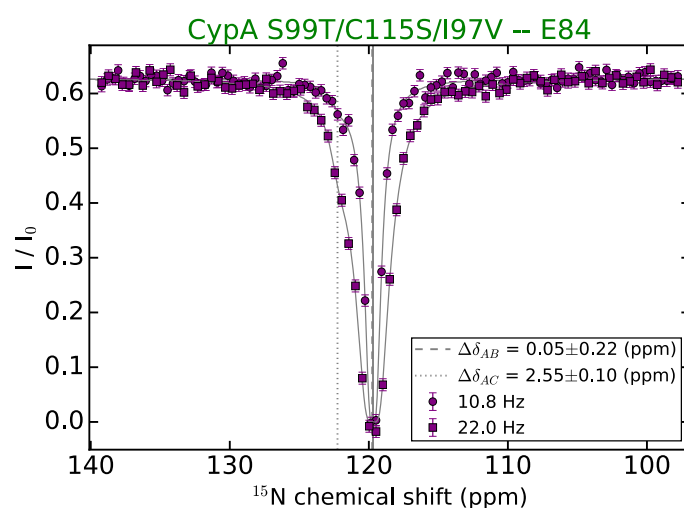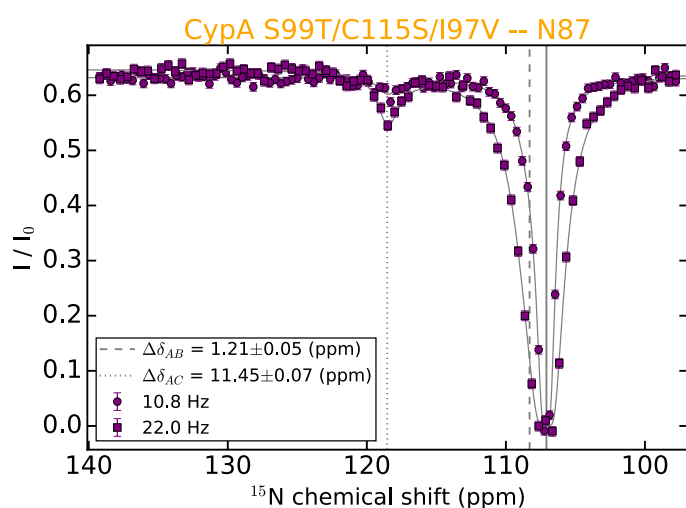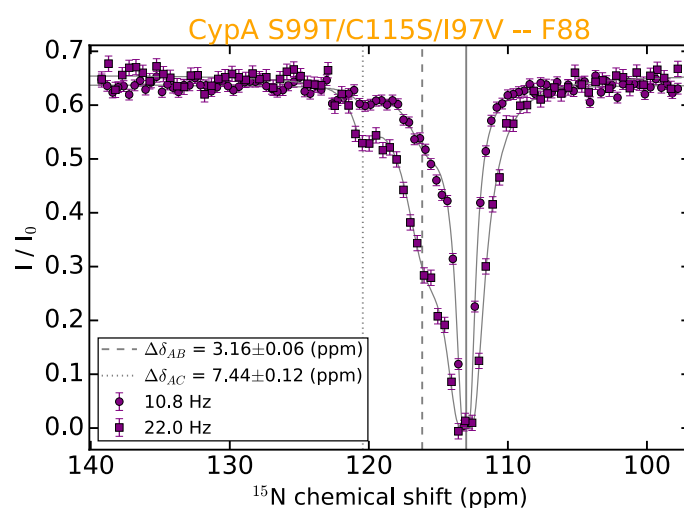

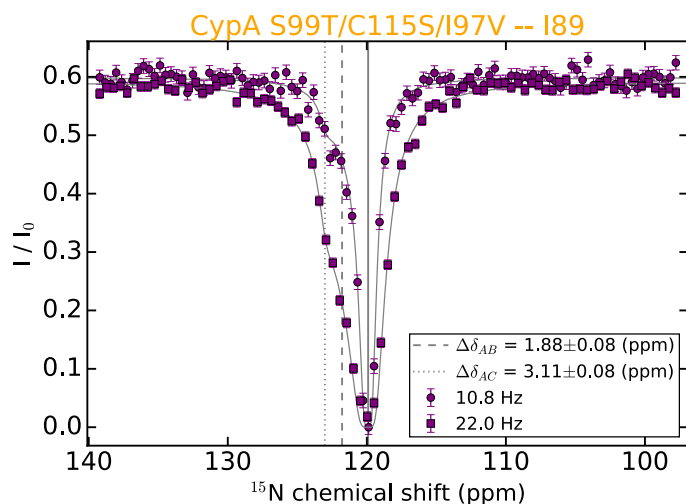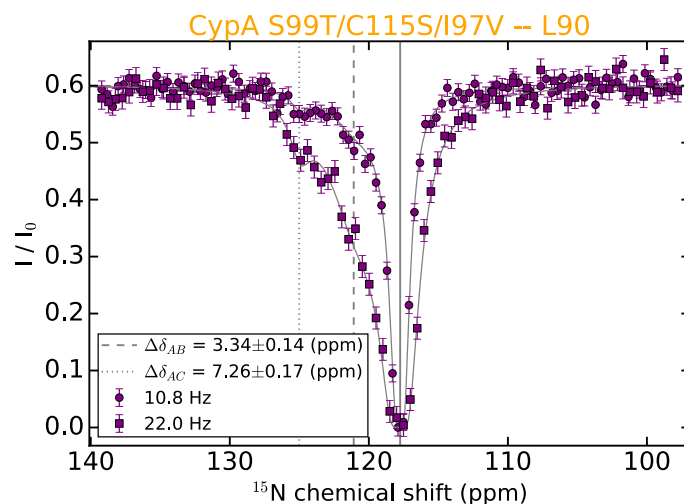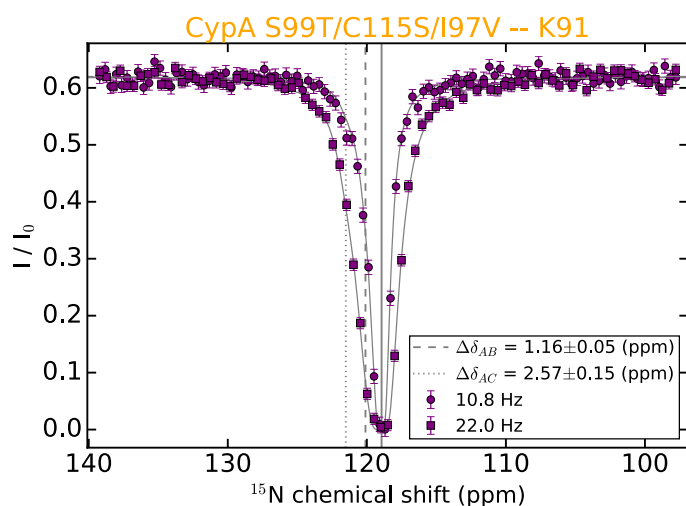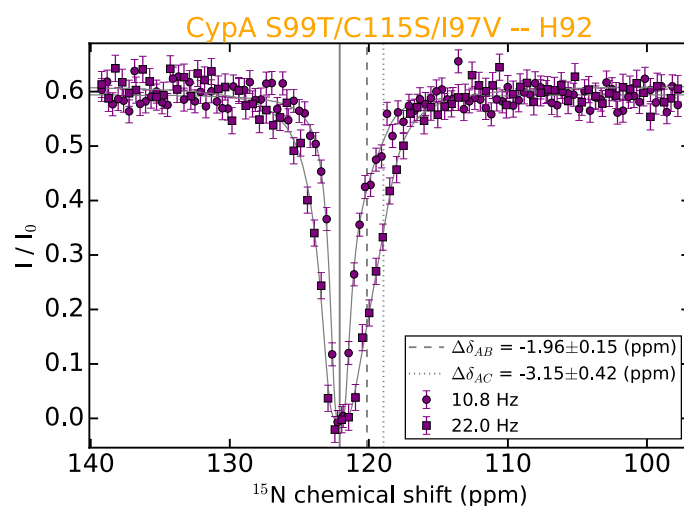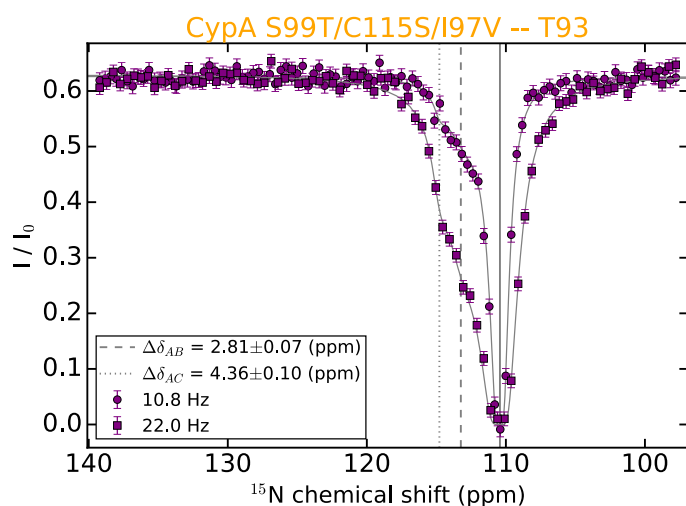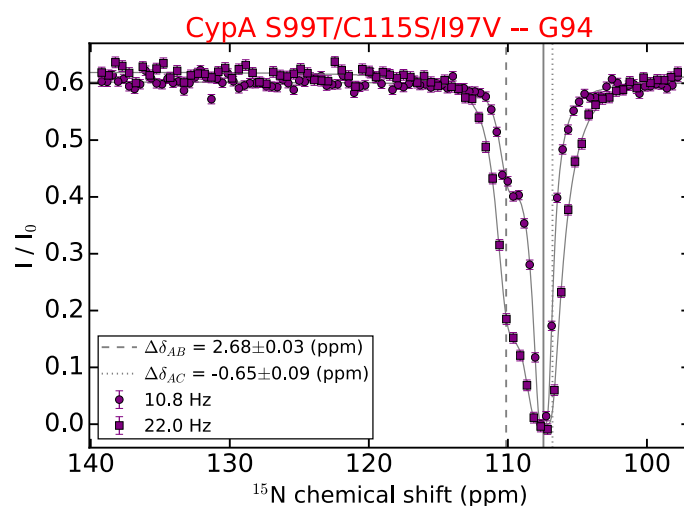

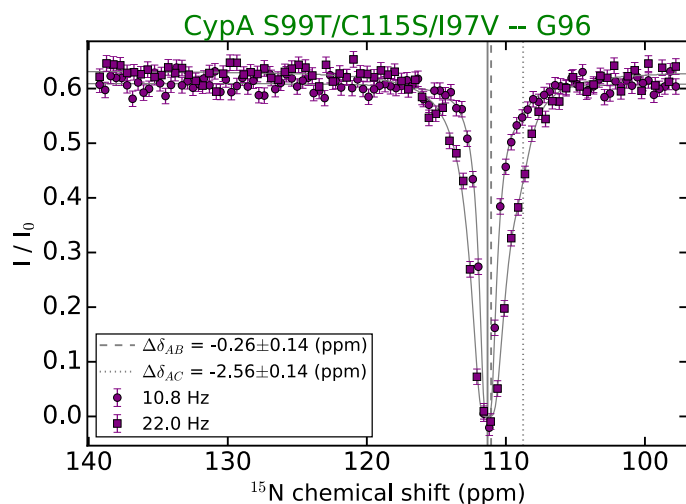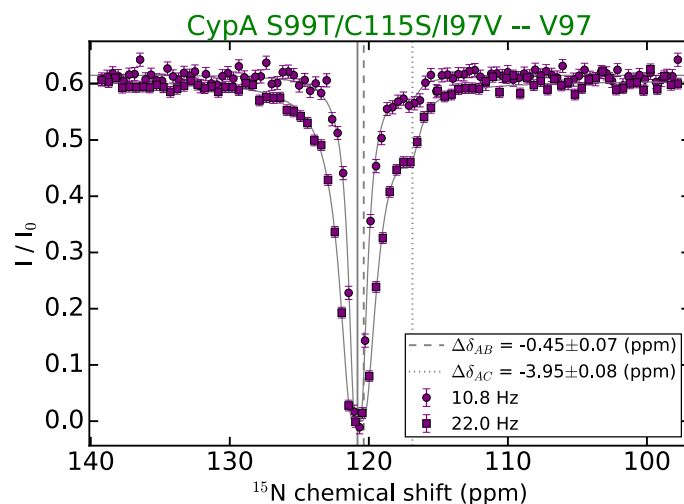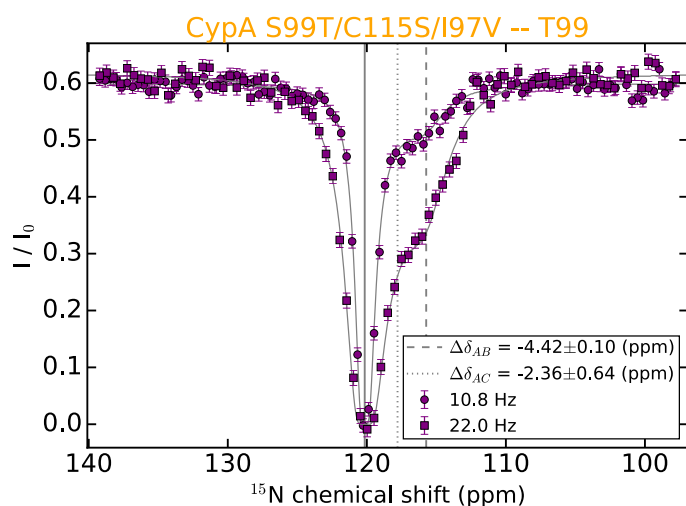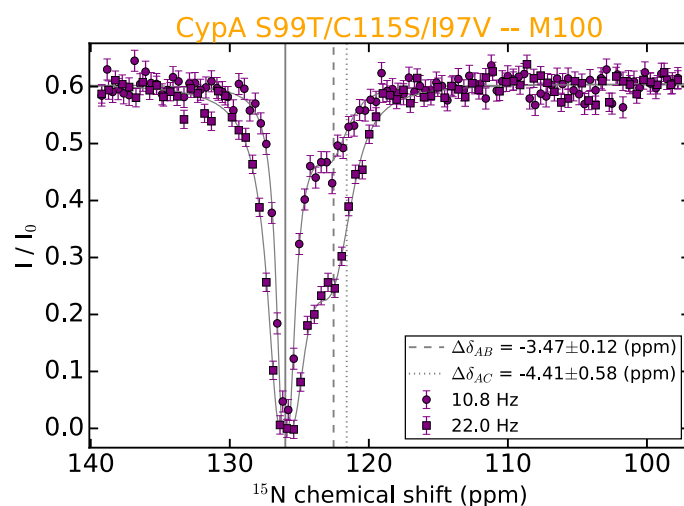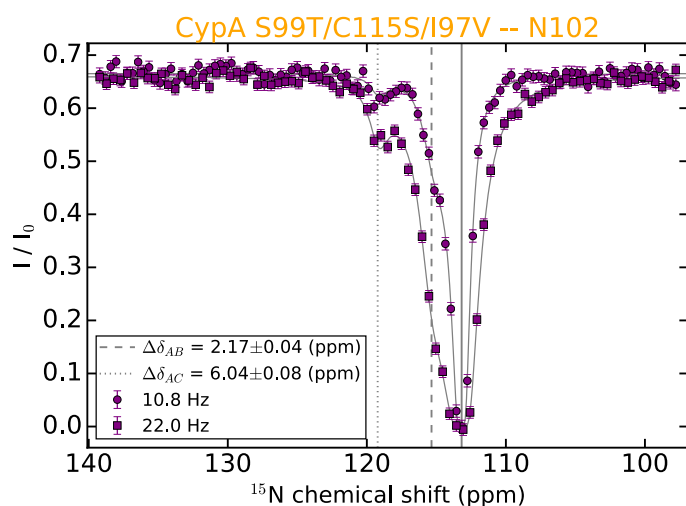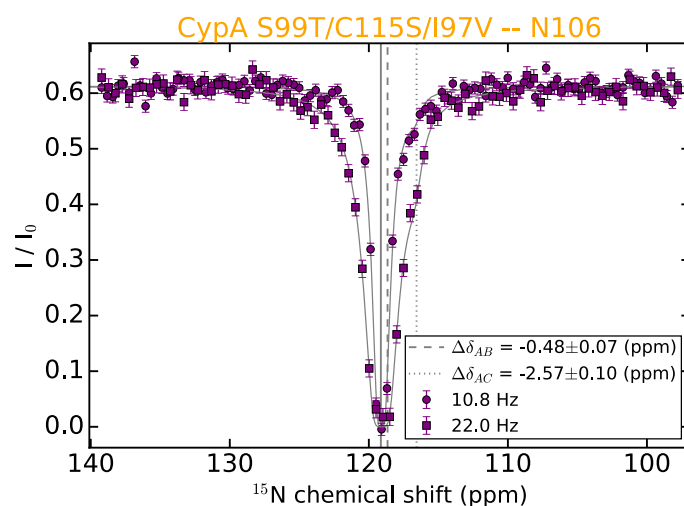

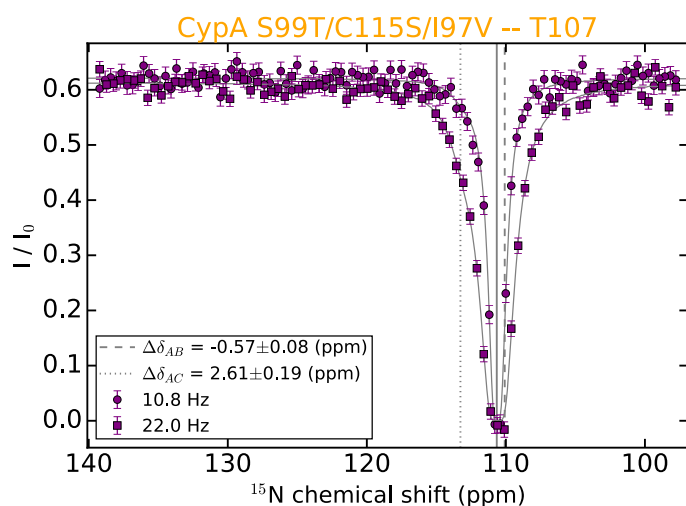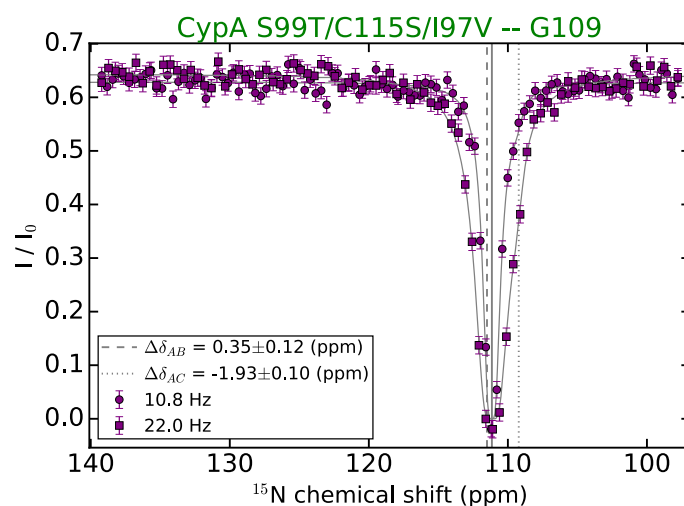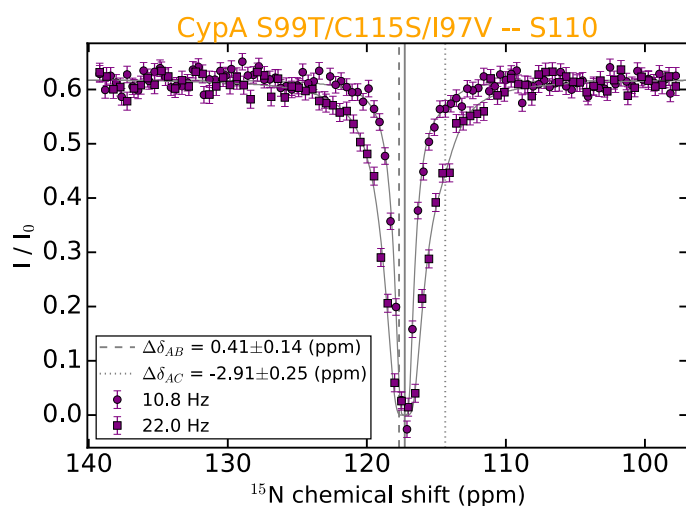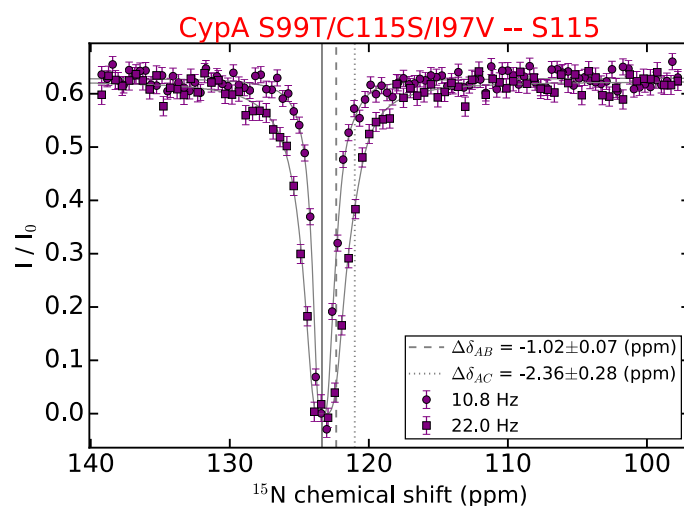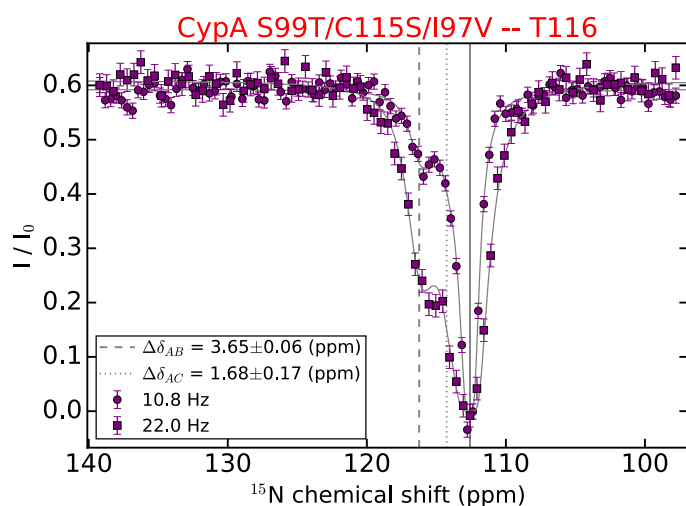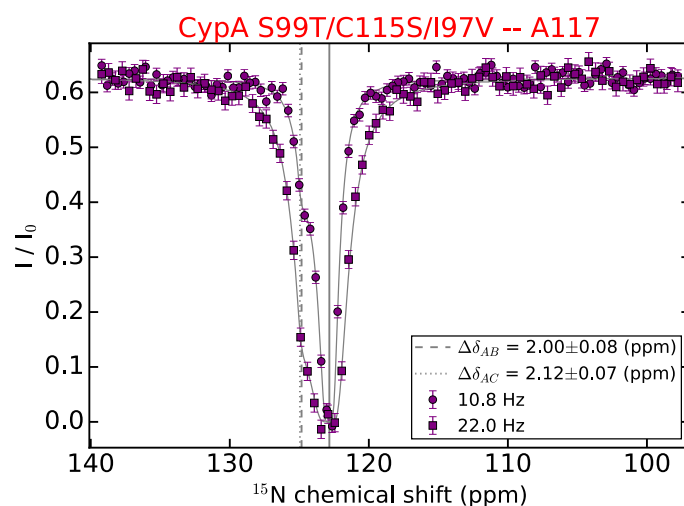

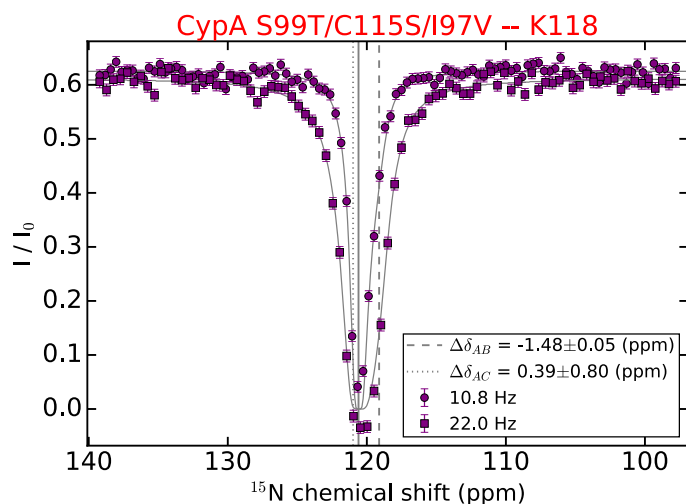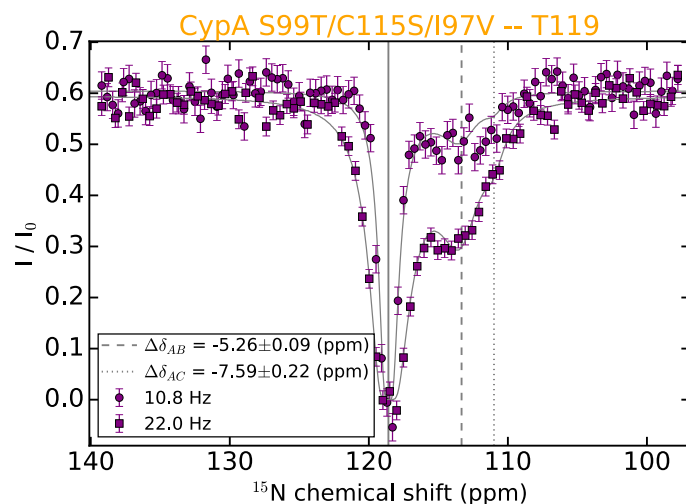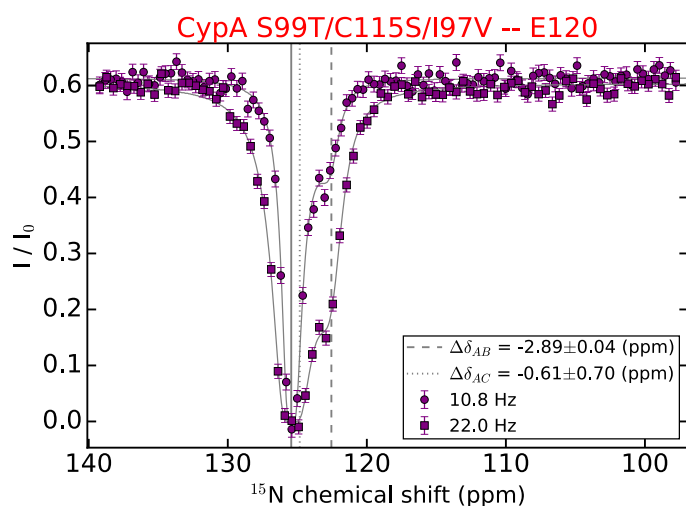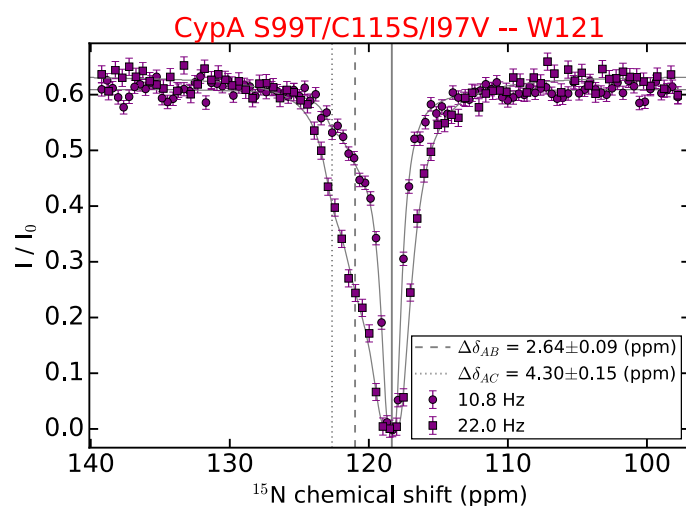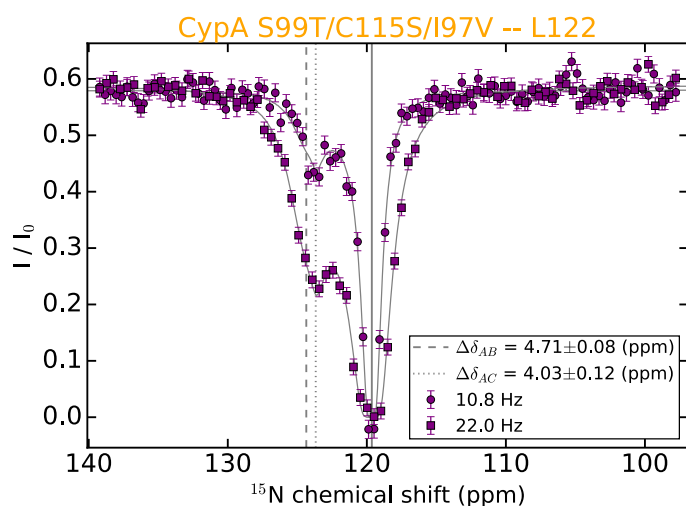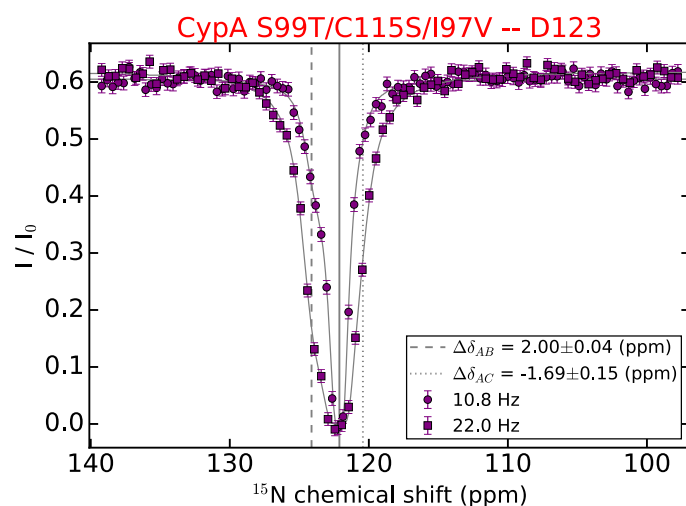

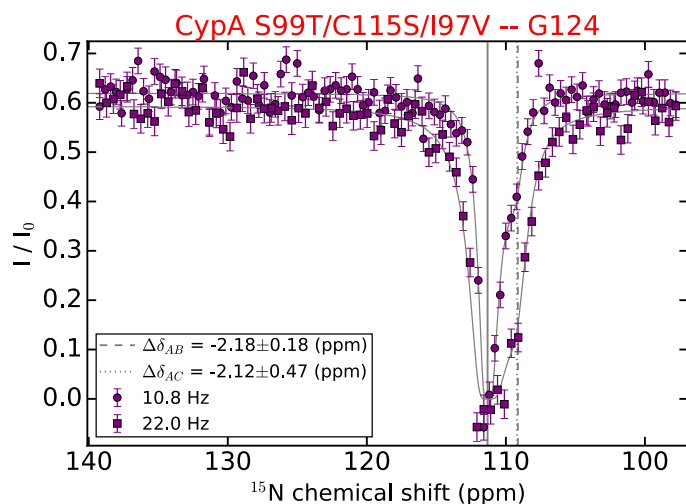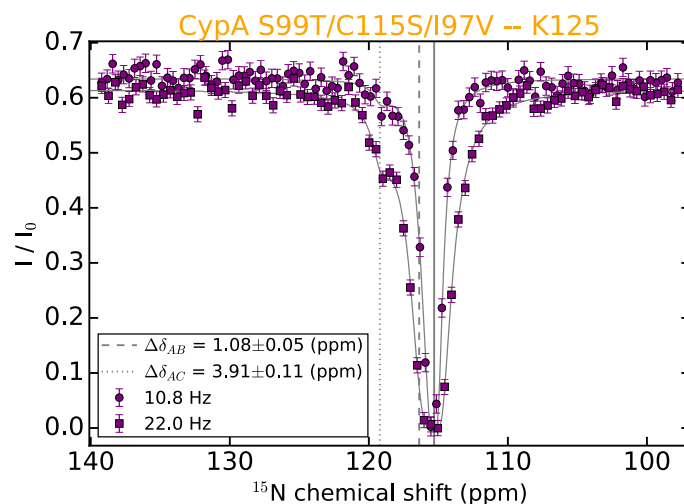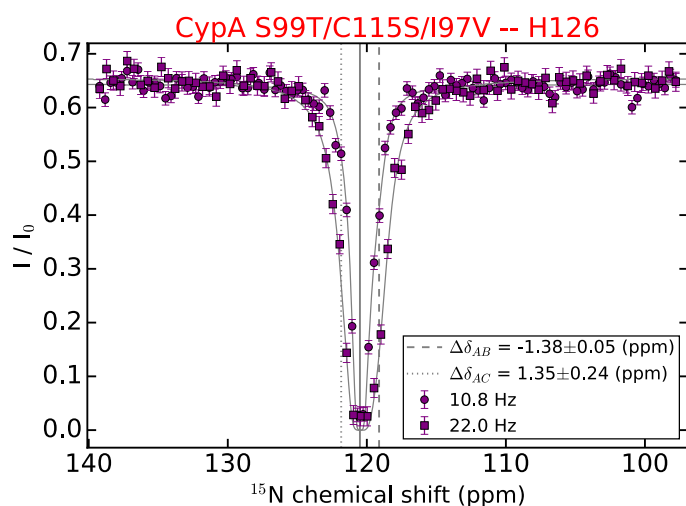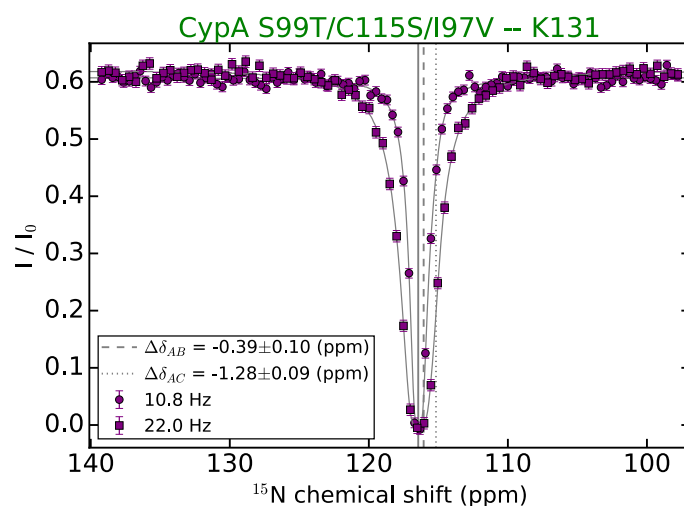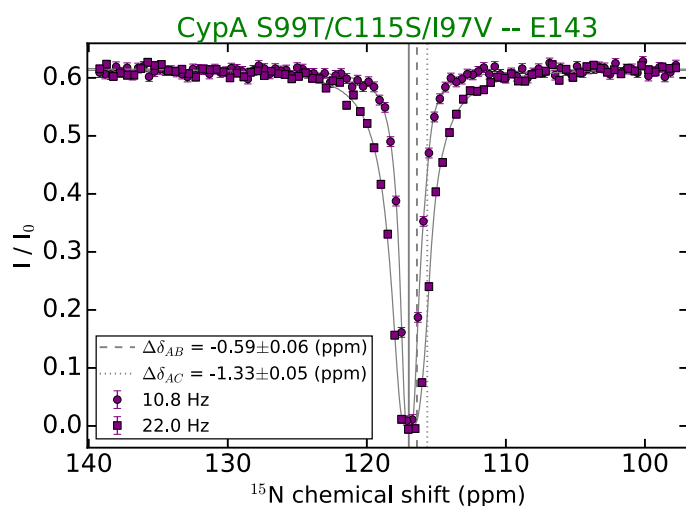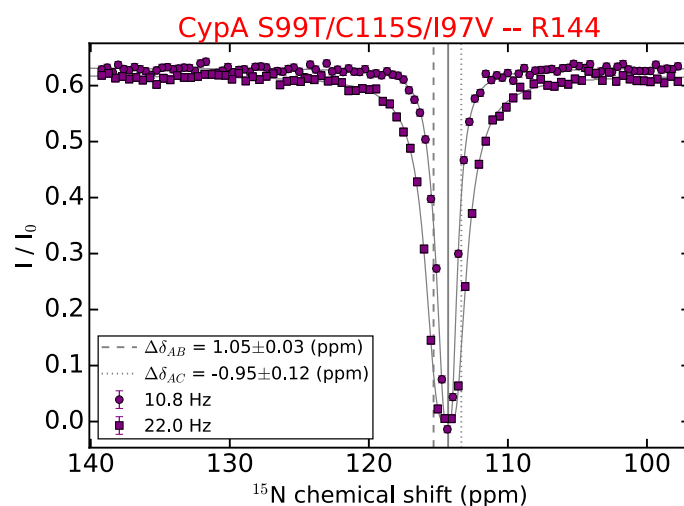

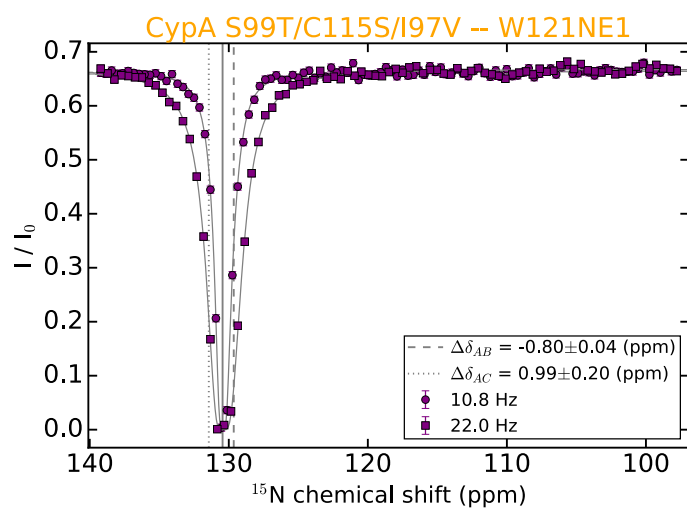

Supplement: Supplementary file 9 — Supplementary Data 6 [file 41467_2018_3562_MOESM9_ESM.pdf]

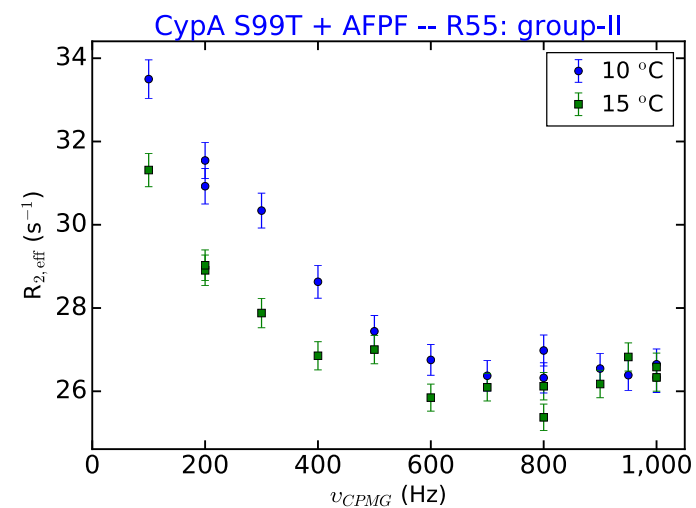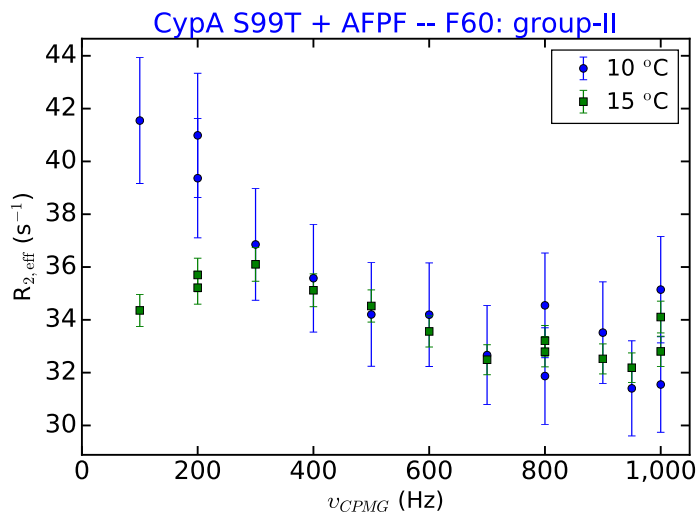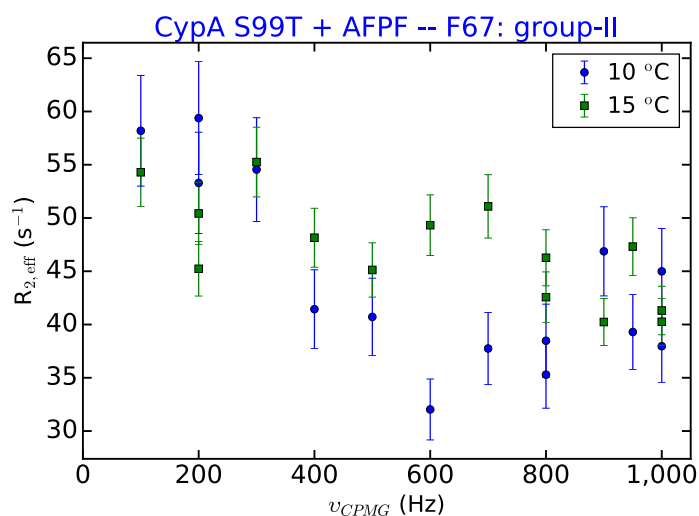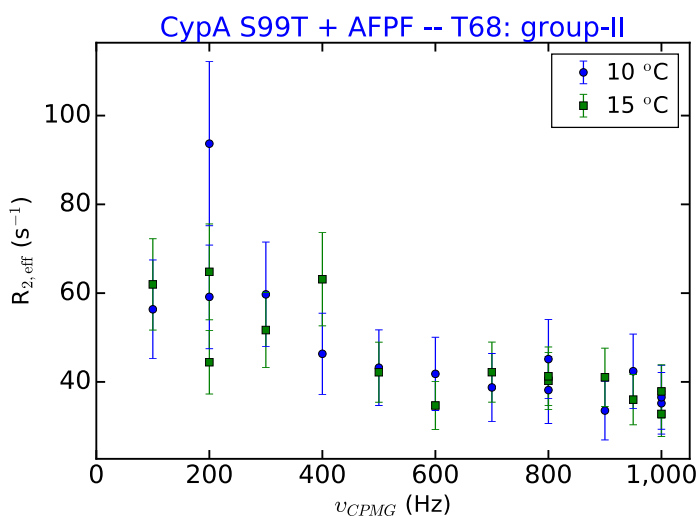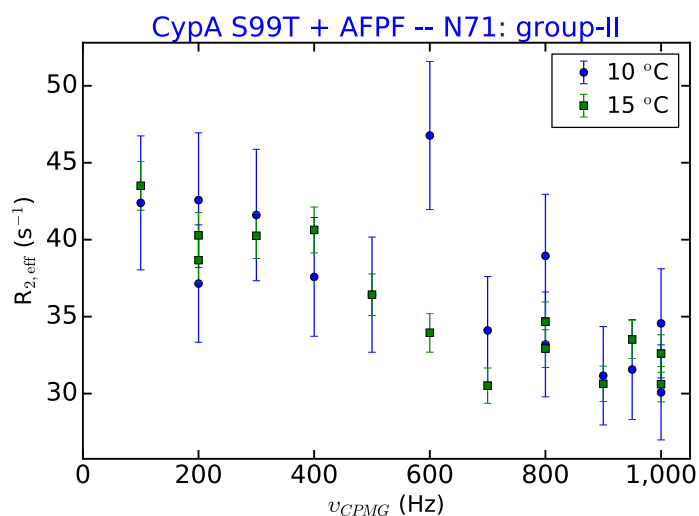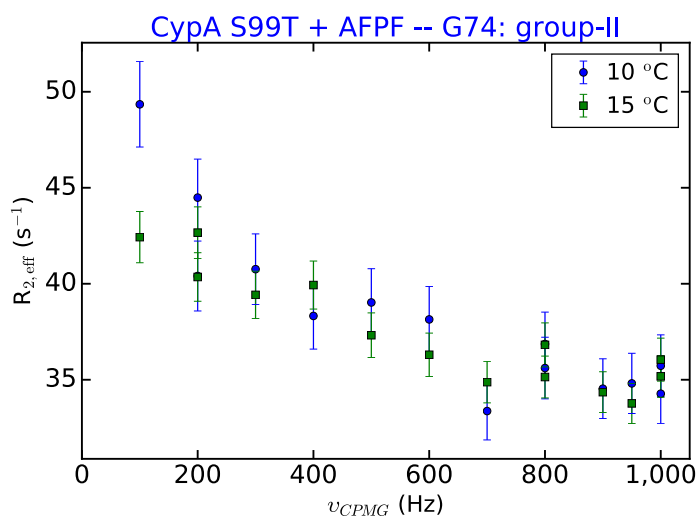

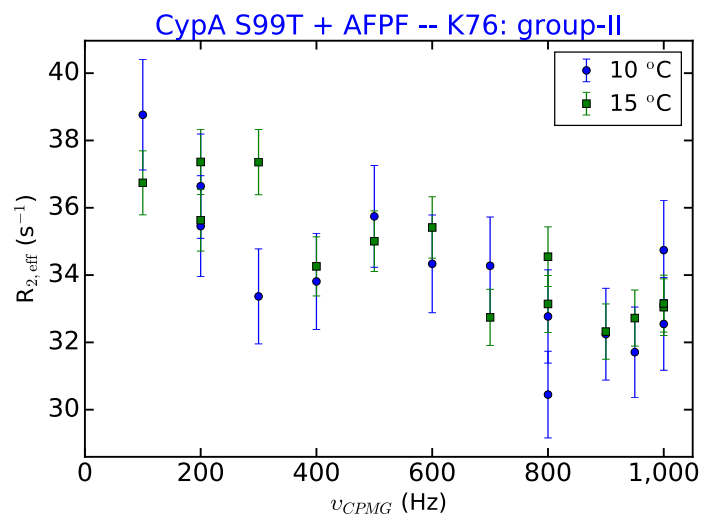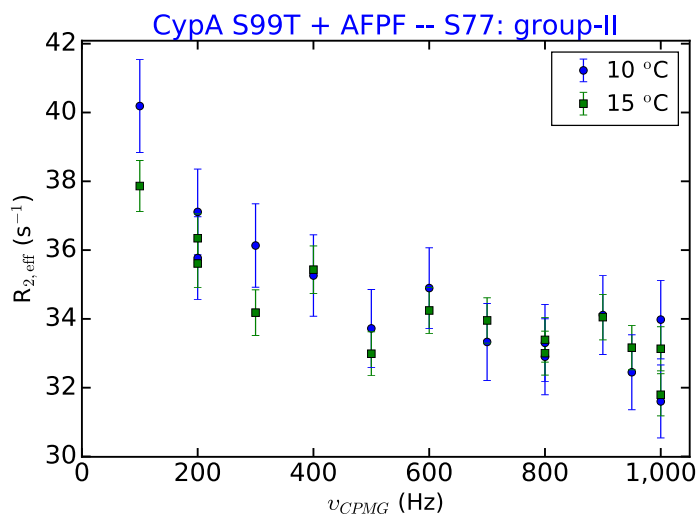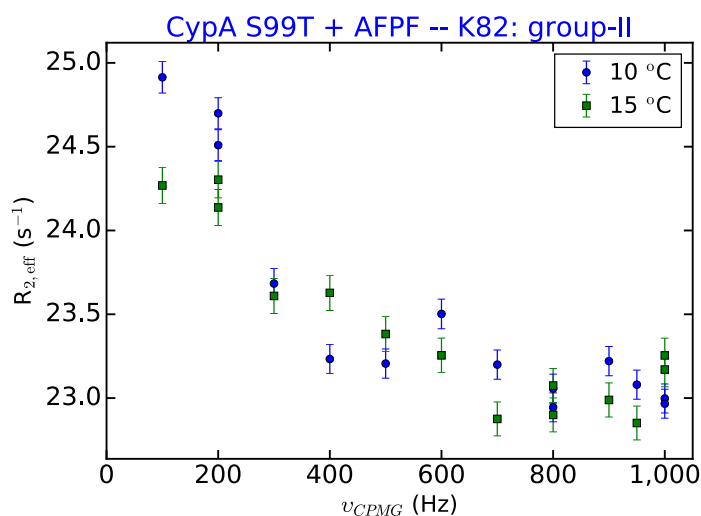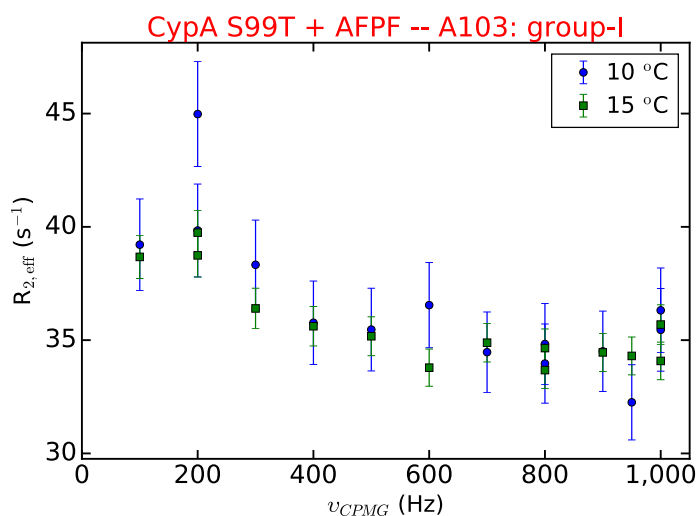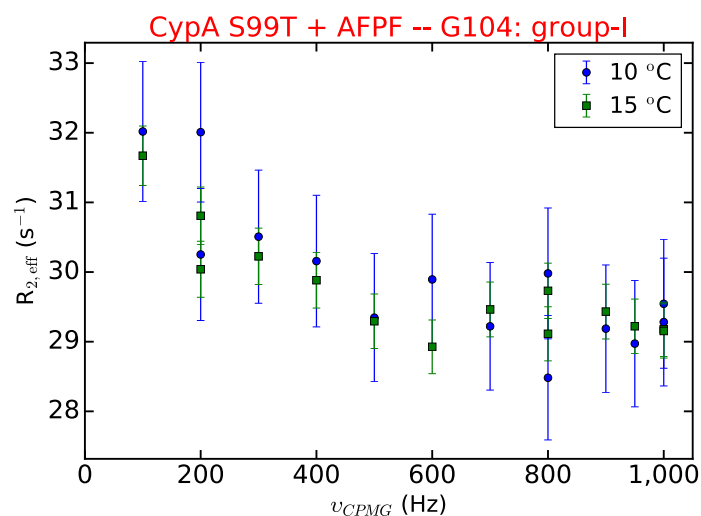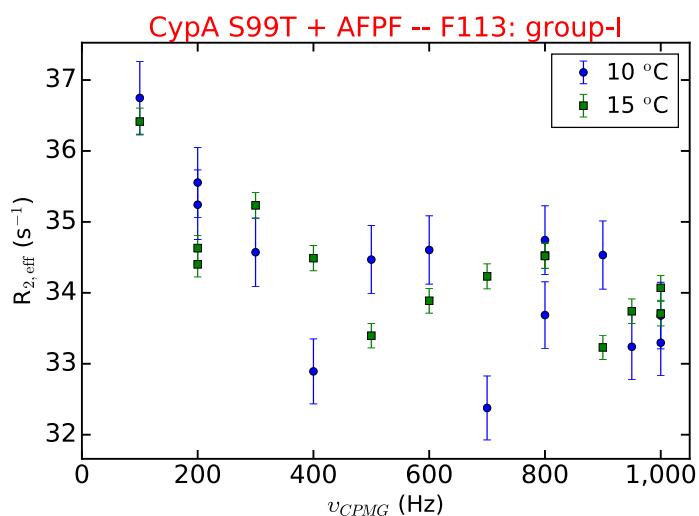

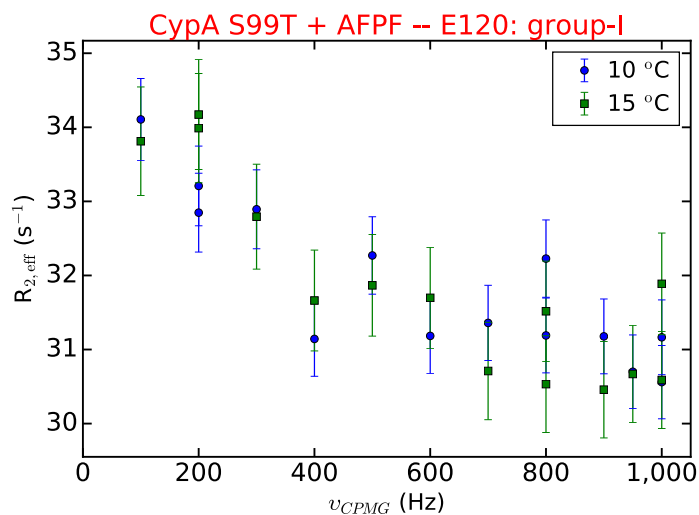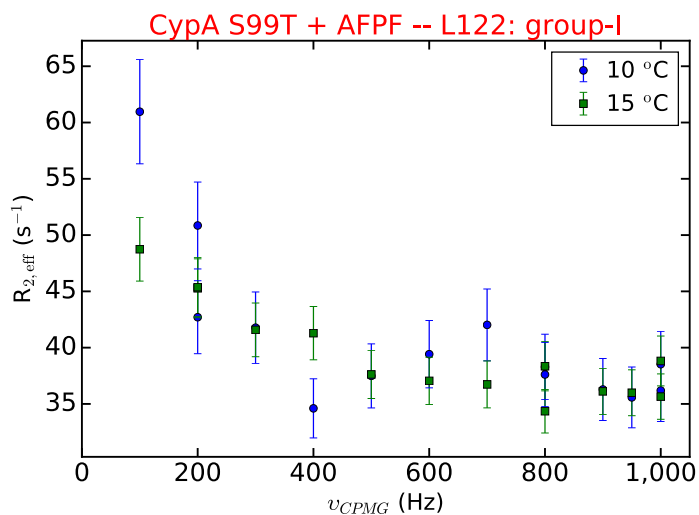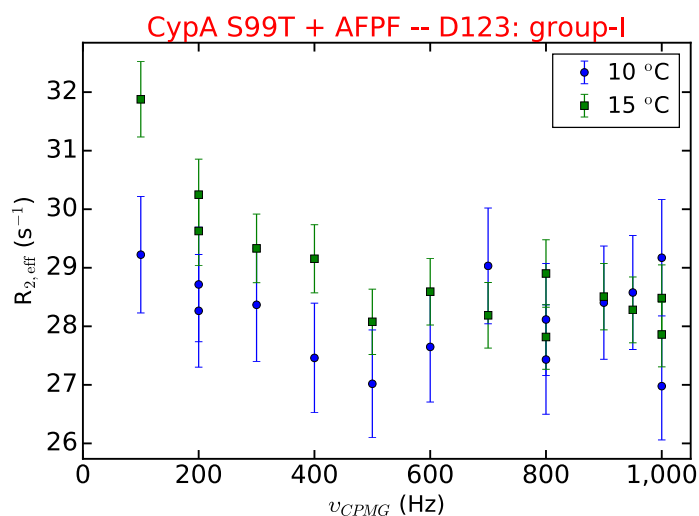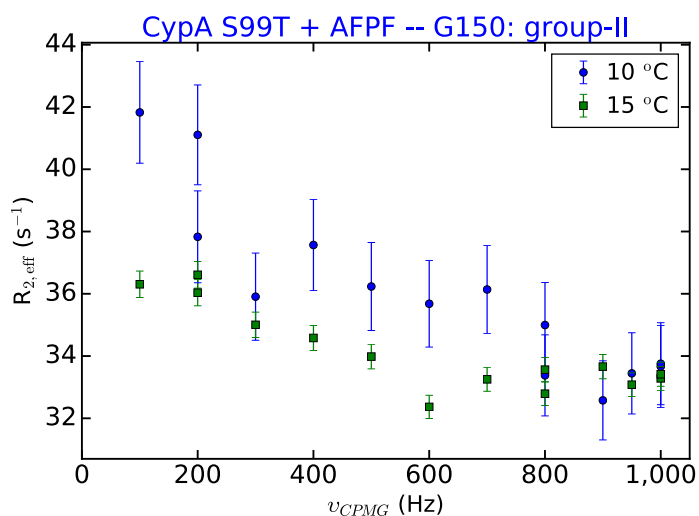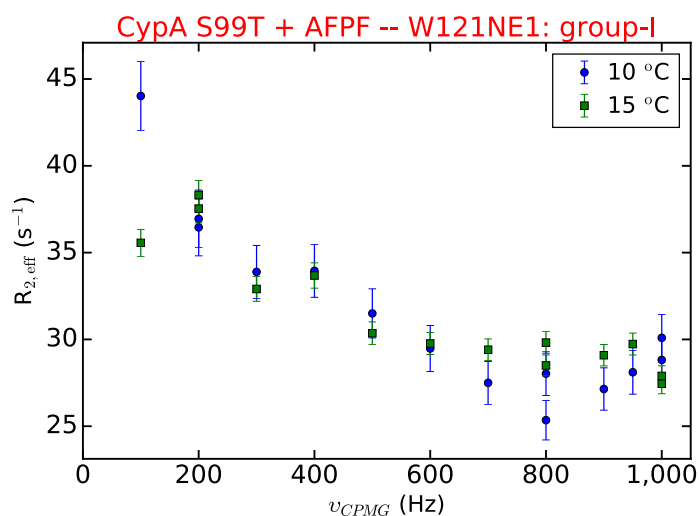

Supplement: Supplementary file 10 — Supplementary Data 7 [file 41467_2018_3562_MOESM10_ESM.pdf]

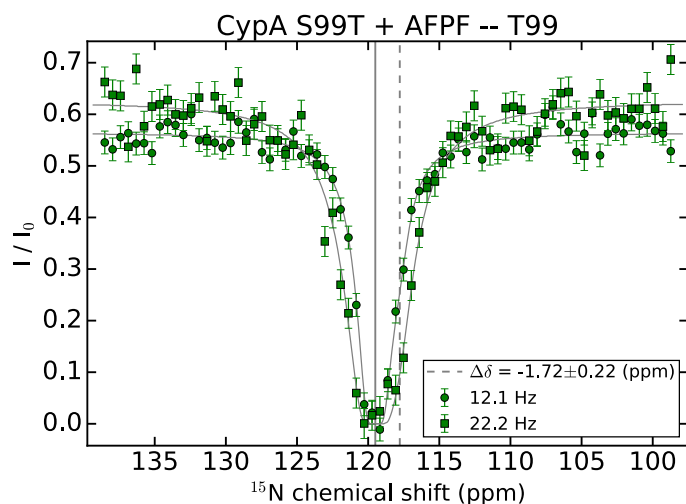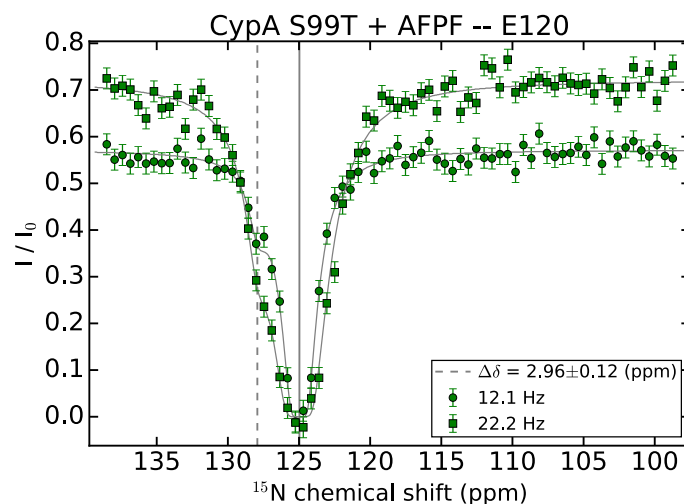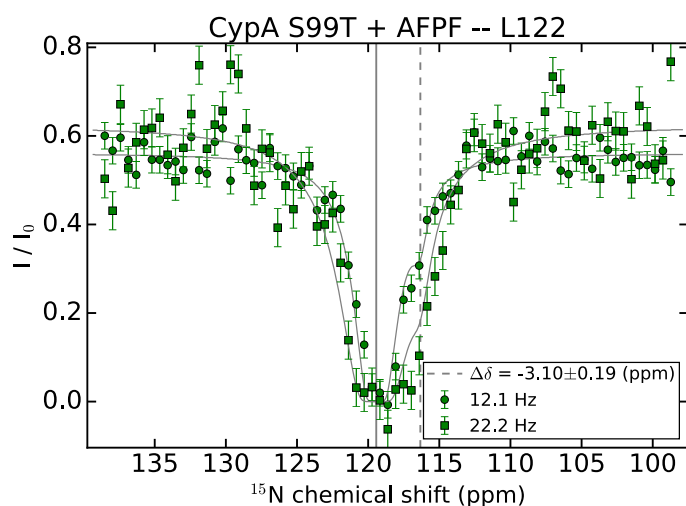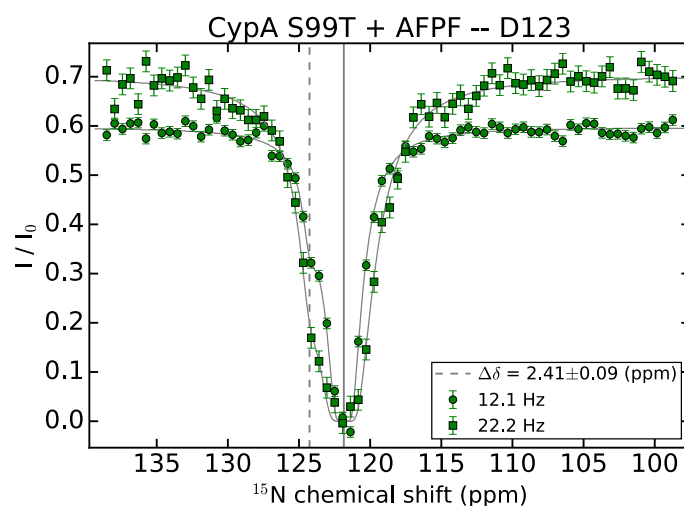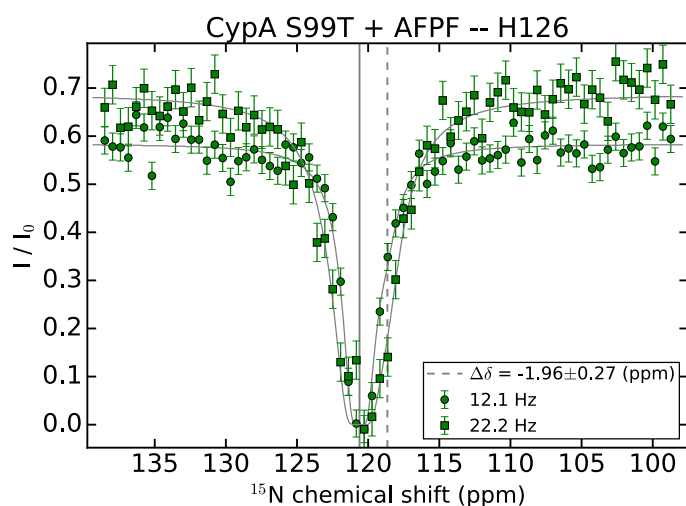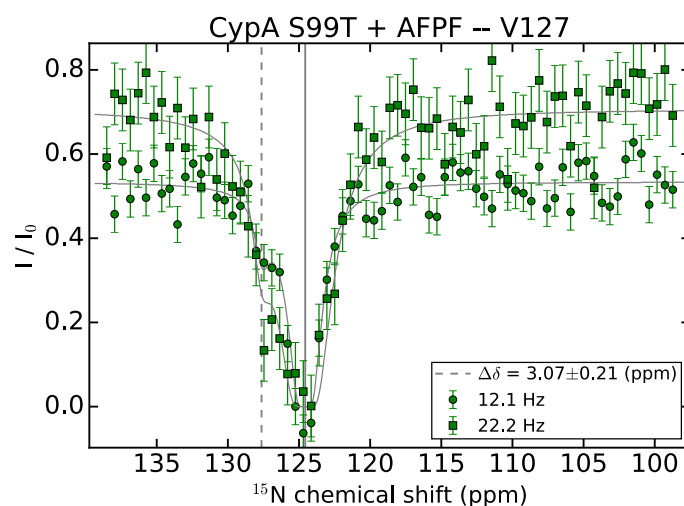

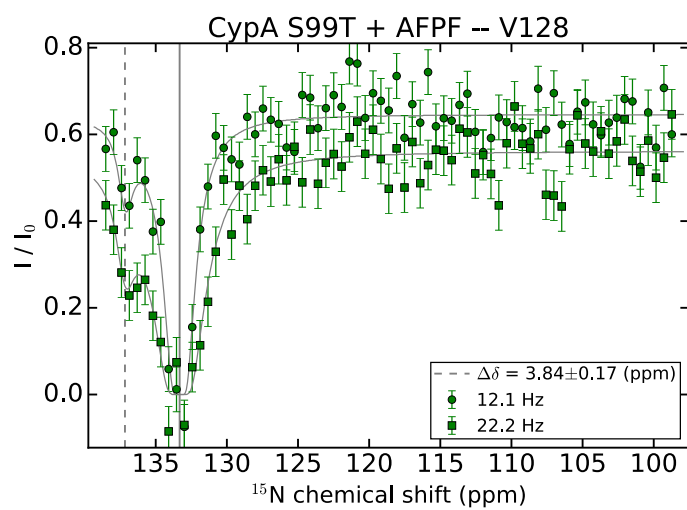

Supplement: Supplementary file 11 — Supplementary Data 8 [file 41467_2018_3562_MOESM11_ESM.pdf]
